# Key anti-freeze genes and pathways of Lanzhou lily (*Lilium davidii*, var. unicolor) during the seedling stage

**DOI:** 10.1371/journal.pone.0299259

**Published:** 2024-03-21

**Authors:** Xuehui Tian, Jianning Li, Sihui Chen

**Affiliations:** 1 Department of Ecological Environment and Engineering, Yangling Vocational and Technical College, Shaanxi, Yangling, China; 2 Gansu Provincial Transportation Planning Survey and Design Institute Limited Liability Company, Lanzhou, Gansu Province, China; Hainan University, CHINA

## Abstract

Temperature is one of the most important environmental factors for plant growth, as low-temperature freezing damage seriously affects the yield and distribution of plants. The Lanzhou lily (*Lilium davidii*, var. unicolor) is a famous ornamental plant with high ornamental value. Using an Illumina HiSeq transcriptome sequencing platform, sequencing was conducted on Lanzhou lilies exposed to two different temperature conditions: a normal temperature treatment at 20°C (A) and a cold treatment at −4°C (C). After being treated for 24 hours, a total of 5848 differentially expressed genes (DEGs) were identified, including 3478 significantly up regulated genes and 2370 significantly down regulated genes, accounting for 10.27% of the total number of DEGs. Quantitative real-time PCR (QRT-PCR) analysis showed that the expression trends of 10 randomly selected DEGs coincided with the results of high-throughput sequencing. In addition, genes responding to low-temperature stress were analyzed using the interaction regulatory network method. The anti-freeze pathway of Lanzhou lily was found to involve the photosynthetic and metabolic pathways, and the key freezing resistance genes were the *OLEO3* gene, 9 *CBF* family genes, and C2H2 transcription factor *c117817_g1* (*ZFP*). This lays the foundation for revealing the underlying mechanism of the molecular anti-freeze mechanism in Lanzhou lily.

## Introduction

Low-temperature stress injury is a common and serious natural disaster in agricultural production that causes huge losses in the world every year, up to hundreds of millions of dollars [1–5]. However, the mo-lecular mechanism of cold resistance in lilies is still poorly understood.

Lily is a world-famous ornamental plant, as well as a homologous plant used as medicine and food. Lily bulbs are rich in nutrition and have multiple functions, such as clearing away lung heat and moisturiz-ing, relieving cough, clearing away heart fire, and enhancing immunity [2, 6, 7]. Studying the molecular mechanism of cold resistance in lily can not only promote the research and development of excellent- quality varieties but also have a wide range of applications to solve the practical problems encountered in lilies production.

Currently, studies on the cold resistance of lilies have mainly focused on Lily cultivation physiology, such as Asiatic lilies [2, 7], Oriental lilies [8, 9], and *Lilium lancifolium* [7, 10]. Research on lily cold resistance mainly focuses on cell membrane permeability and the protective enzyme system. The relative electrical conductivity, the lipid membrane oxidation product malondialdehyde, and the activity of antioxidant en-zymes (superoxide dismutase, catalase, and peroxide dismutase) are usually used as physiological indica-tors of plant cold tolerance [11–14]. Hoshi *et al*. [15] studied the transcriptome of lilies mediated by Agro-bacterium and established a transgenic production system for hybrid Oriental lily varieties. Du [16] se-quenced the transcriptional data of different lily organs and developed simple sequence repeat (SSR) markers. Tang *et al*. [17] investigated the secondary metabolites of different lily bulb extracts and their an-tioxidant capacity. Shafiee-Masouleh *et al*. [6] studied the magnetic and chelating structure of nano-chitosan as a supplementing fertilizer for lilies. Zhao *et al*. [18] compared and analyzed the petal ep-idermal wax components and water loss resistance of five cut flower lily varieties. Chen *et al*. [19] estab-lished a gene transformation system for Lily. In Lanzhou lily current research mainly focuses on cultivation techniques and bulb nutrition. Teng *et al*. [20] examined the effects of different substrates on the growth of lily bulbs. Ling *et al*. [21] investigated the isolation, purification and identification of pathogens from Lanzhou lily. Rao *et al*. [22] studied the influence of different potassium ion concentrations on the accu-mulation of secondary metabolites in the bulbs of Lanzhou lily. Lu *et al*. [23] investigated the nutritional components of the bulbs of Lanzhou lily from different lily-producing areas. Chen *et al*. [24] explored the prevention and control of the rot of Lanzhou lily bulbs during storage. Li *et al*. [25] studied the effect of low-temperature storage on carbohydrate metabolism of Lanzhou lily. Sun *et al*. [26] researched the influ-ence of different temperatures on the germination and growth of Lanzhou lily. However, there have been no the frost resistance of Lanzhou lily at the seedling stage.

Our previous studies have shown that the Lanzhou lily (*Lilium davidii*, var. unicolor) has a strong tolerance to low temperatures, and its growth is unaffected. However, the effect of subzero temperatures on the growth of Lanzhou lily, especially the molecular mechanism, remains to be further studied [27]. In this study, advanced Illumina high-throughput sequencing platform transcriptome sequencing technology was used to sequence the transcriptome mRNA of the leaves of Lanzhou lily growing at normal and sub-zero ultra-low temperatures, and the differentially expressed genes (DEGs) and pathways at normal and sub-zero low-temperatures were compared to investigate the gene expression of Lanzhou lily under ul-tra-low-temperature stress. Gene Ontology (GO) and Kyoto Encyclopedia of Genes and Genomes (KEGG) annotation and concentration analysis were conducted for DEGs to further select genes related to freezing resistance in Lanzhou lily.

## Materials and methods

### Experimental materials and growth conditions

Forty 3-year-old Lanzhou lily bulbs were purchased from a local flower market in Qilihe District (103°54’E, 35°56’N, altitude 2638 m), Lanzhou city, Gansu Province, China, and stored for 110 days in a refrigerator at 2°C. After vernalization, the bulbs were planted in 15-cm-diameter flower pots filled with 2 L of commercial cultivation substrate composed of ½ peat + ½ garden soil (Mengda, Yufeng Co., Ltd., Xianyang, China) and placed in an intelligent artificial climate box (RXZ-0288, Ningbo Jiangnan Instru-ment Factory, Ningbo, China) at the Northwest Agriculture and Forestry University, Yangling, Shaanxi, China. The growth environment temperature was set to 20°C during the day and 15°C at night, the rela-tive air humidity was 50%–65%, the light-dark cycle was 16 light– 8 h dark, and the mean light illumi-nance was (350 ± 20) μmol /(m2 • s). Every 3–5 days, 300 mL of water was poured onto the culture medi-um, and Hoagland nutrient solution was used to provide adequate nutrients. The nutrient solution formula was as follows: 945mg/L calcium nitrate, 607mg/L phosphate nitrate, 115mg/L ammonium phosphate, 493mg/L magnesium sulfate, 2.5ml/L iron salt solution, and 5ml/L trace elements at pH = 6.0.

### Chemicals and treatments

Lily seedlings that had grown to approximately 15 cm in height were placed in an intelligent low-temperature artificial climate chamber for low-temperature treatment at −4°C [26], and the treatments lasted for 24 h [11, 28, 29]. The room temperature was 20°C in the control, and each treatment was repeated three times. Leaves from the same leaf position (the seventh to 10th leaves counted from the top) were sam-pled, immediately frozen in liquid nitrogen, and stored at −80°C until further processing.

### Chemicals and treatments

Lily seedlings that had grown to approximately 15 cm in height were placed in an intelligent low-temperature artificial climate chamber for low-temperature treatment at −4°C [26], and the treatments lasted for 24 h [11, 28, 29]. The room temperature was 20°C in the control, and each treatment was repeated three times. Leaves from the same leaf position (the seventh to 10^th^ leaves counted from the top) were sampled, immediately frozen in liquid nitrogen, and stored at −80°C until further processing.

### Transcription sequencing

The total RNA of three samples of Lanzhou lily leaves was extracted using the TRIzol method [16], and the concentration, purity, and integrity of the RNA were detected. After the samples were qualified, magnetic beads with Oligo (dT) were used to enrich the eukaryotic mRNA [16]. The mRNA was then fragmented into short fragments by adding a fragmentation buffer. These mRNA fragments were then used as templates to synthesize both one strand of cDNA two strands of cDNA. Subsequently, AMPure XP beads were used to purify the synthetic two-stranded cDNA, followed successively by end repair, the addition of polyA tails, the connection of sequencing joints, and selection of fragment size.

The Illumina 4000 high-throughput sequencing platform (HiSeq/MiSeq) was used for sequencing after passing the quality inspection [30–33]. The data sequenced data were termed raw reads or raw data. Then, the transcription results were assembled and spliced, and the gene function annotation and expression levels were analyzed. Finally, polymerase chain reaction (PCR) amplification was performed, and the PCR products were purified to obtain the final library. The clean data were deposited in the Short Read Archive database of the National Center for Biotechnology Information (NCBI) under Accession No. PRJNA565864.

### Real-time fluorescence quantitative PCR (QRT-PCR) validation

Ten differentially expressed genes were randomly selected for QRT-PCR analysis to confirm the reliability of RNA sequencing (RNA-Seq). The leaf RNA of Lanzhou lily was used as material at 20°C and −4°C, and cDNA was synthesized via reverse transcription using the Hi Script II QRT Super Mix for the PCR kit (Nanjing Nuoweizan Biotechnology Co., Ltd., Nanjing, China) (Table 1). The Actin gene was used as the internal reference [34]. Each treatment was repeated three times to calculate the relative expression of the gene.

**Table 1:** Primer sequences for QRT-PCR.

Gene ID	Forward primer (5′→ 3′)	Reverse primer (5′→ 3′)
*c166605_g1*	TGGTCTGGATGTGTTGCG	GTCATAATTGAGTTTGTGGGAG
*c199368_g1*	GCGGGCCAGCTCCTTGTGAA	GCGGGTTCAGCGTGCAGGA
*c171769_g1*	GCAACATCATCAACTCTGG	ATGGAAGCAATCACCTAAGT
*c164813_g2*	TCCAGTCCACCACAGTCT	GCCAGCAGAACAGTCAAG
*c153721_g1*	GTTGAGCCAGCCTACATC	CTAGACTCCGCACTCTGT
*c76799_g1*	TGGAGCAATTAGGCCCGGATGA	CACCAGAGCCAGGCCCAACACT
*c153860_g1*	GGCGCGAGTACGAGCTGGTGA	CGCCTCGTCGGTCTTGTCGG
*c149445_g1*	TGAAAAGGTGAGGTCACCAG	GAACAGAGATGAAAGAAAGTTGG
*c115377_g1*	GCTTGTGGCTGTGTAGATTAAG	TCCACAACCTATTATCACCTCT
*c155535_g1*	GCAAGCAAAGCCTTGGATC	ATTGCCGGAGATCAAACG
*β-Actin*	TGAGCACATTCCAGCAGA	CCATAGACAAAGCCATCG

### Statistical analysis

QRT-PCR assays were analyzed using a t-test at p < 0.01 and < 0.05. The primers for these genes were designed with PRIMER5.0 and are listed in Table 1. The transcriptome data were analyzed by Allwegene Technology Co., Ltd. (Beijing, China).

The DEGs that respond to cold stress were subjected to co-expression network analysis using the weighted gene co-expression network analysis (WGCNA) function in the R package [35–37]. A soft threshold was selected based on scale independence higher than 0.8. Hierarchical clustering trees were built for DEGs within the network using a dissimilarity matrix among genes, which were then assigned into different modules by dynamic shearing, with the default parameter setting of Min Module Size = 30 and Cut Height = 0.25. For each module (namely a group of highly correlated genes), a filtered co-expression network was drawn to highlight hub gene(s) using the Cytoscape 3.8.0 software [38–40].

## Results

### Sequencing results spliced with Trinity

**Fig 1:**
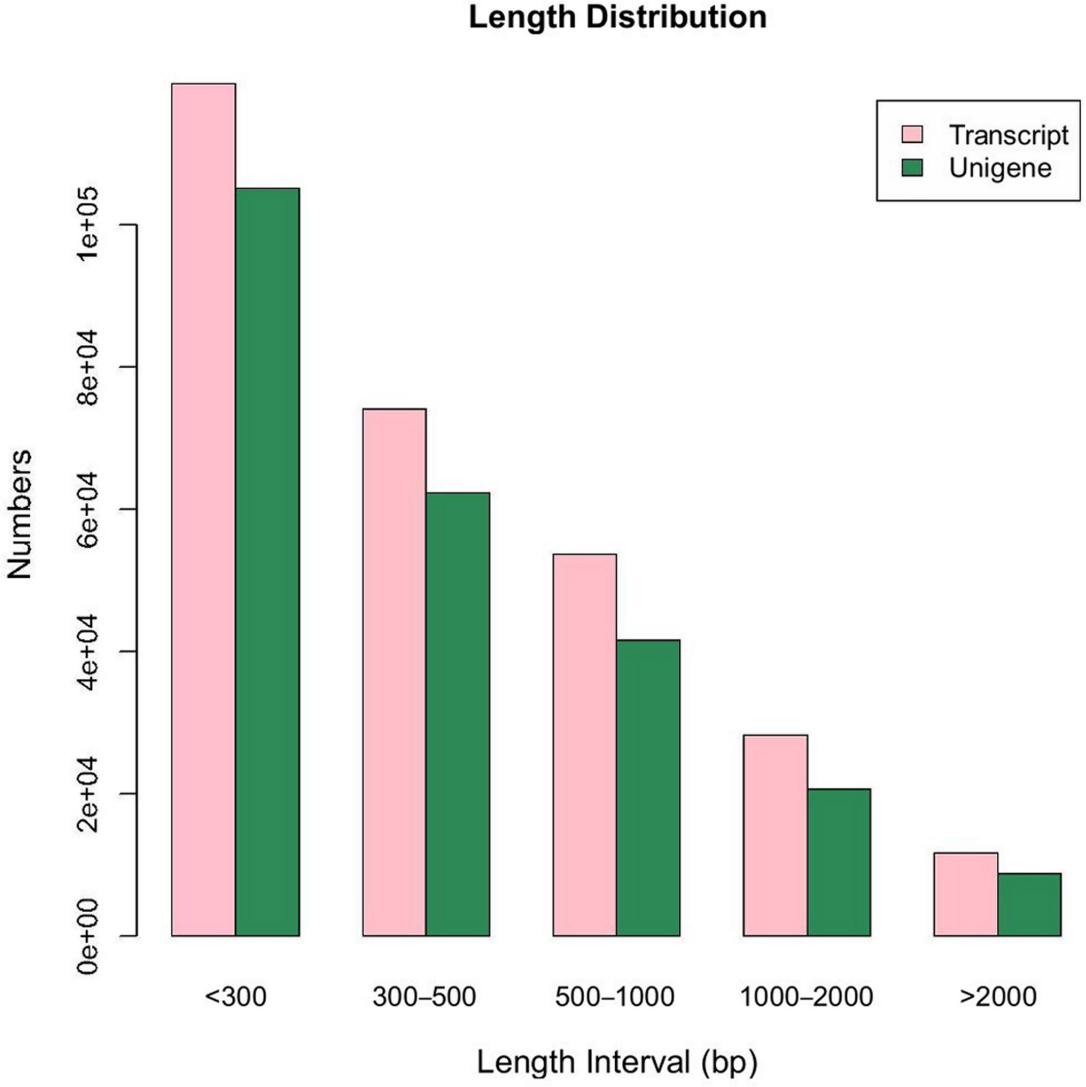
Length distribution of all unigenes. Note: The abscissa in the figure is the length interval of the spliced transcriptor unigene, and the ordinate is the frequency of occurrence of spliced transcripts or unigene of each length.

A total of 38.57 million and 38.66 million raw reads were obtained from the transcriptome libraries of lily leaves under the A(20°C) and C(−4°C) treatments, respectively (S1 Table), with a Q20 value of 94.56%, a Q30 value of 87.32%, and a sequence base GC content of 44.84%. The results indicate that the transcriptome sequencing of Lanzhou lily was of high quality and could provide a good basis for subsequent data assembly and analysis. The results of redundant sequence removal using Trinity transcript splicing and the clustering of similar sequences (CD-HIT) are shown in Table 2. The maximum unigene sequence length was <300 (Fig 1), indicating that transcription splicing was of high quality and conducive to subsequent analysis and research.

**Table 2:** Number of sequences and total base number before and after Trinity transcript splicing and CD-HIT to remove redundant sequences.

-	Shortest	Median	Longest	N50	N75	N90	Total Nucleotides
Transcript	201	341	21,073	829	376	252	168,637,667
Unigene	201	327	21,073	757	350	246	133,179,110

Note: N50, N75, and N90 represent the length of the transcripts of not less than 50, 75, and 90%, respectively; CD-HIT, clustering of similar sequences.

### Gene function annotation results

Database comparison of the obtained transcriptome sequences showed that the number of annotated unigenes was 238,301, and the number of unigenes annotated by the NCBI protein non-redundant database (Nr) was 65,314, accounting for 27.41% of the total number of unigenes. The number of unigenes annotated by the eukaryotic orthologous gene database (KOG) was 42,138, accounting for 17.68% of the total number of unigenes, and the number of unigenes annotated by the GO library was 35,244, accounting for 14.79% of the total number of unigenes. The number of unigenes in the Swiss-Prot annotation was 46,337, accounting for 19.44% of the total number of unigenes, while the number of unigenes in the Pfam annotation was 58,605, accounting for 24.59% of the total number of unigenes (Table 3).

**Table 3:** Annotation statistics for all unigenes.

Database	Number of Unigenes	Percentage (%)
Annotated in NR	65,314	27.41
Annotated in NT	37,916	15.91
Annotated in KO	26,291	11.03
Annotated in SwissProt	46,337	19.44
Annotated in Pfam	58,605	24.59
Annotated in GO	35,244	14.79
Annotated in COG/KOG	42,138	17.68
Annotated in all databases	9695	4.07
Annotated in at least one database	84,559	35.48
Total number of unigenes	238,301	100

Note: NR, non-redundant; NT, Nucleotide Sequence Database; KO, Kyoto Encyclopedia of Genes and Genomes (KEGG) Orthology; SwissProt, Swiss-Prot protein sequence database; Pfam, protein families; GO, Gene Ontology; COG/KOG, the Eukaryotic orthologous gene database.

### DEGs, KEGG analysis, and QRT-PCR verification

**Fig 2:**
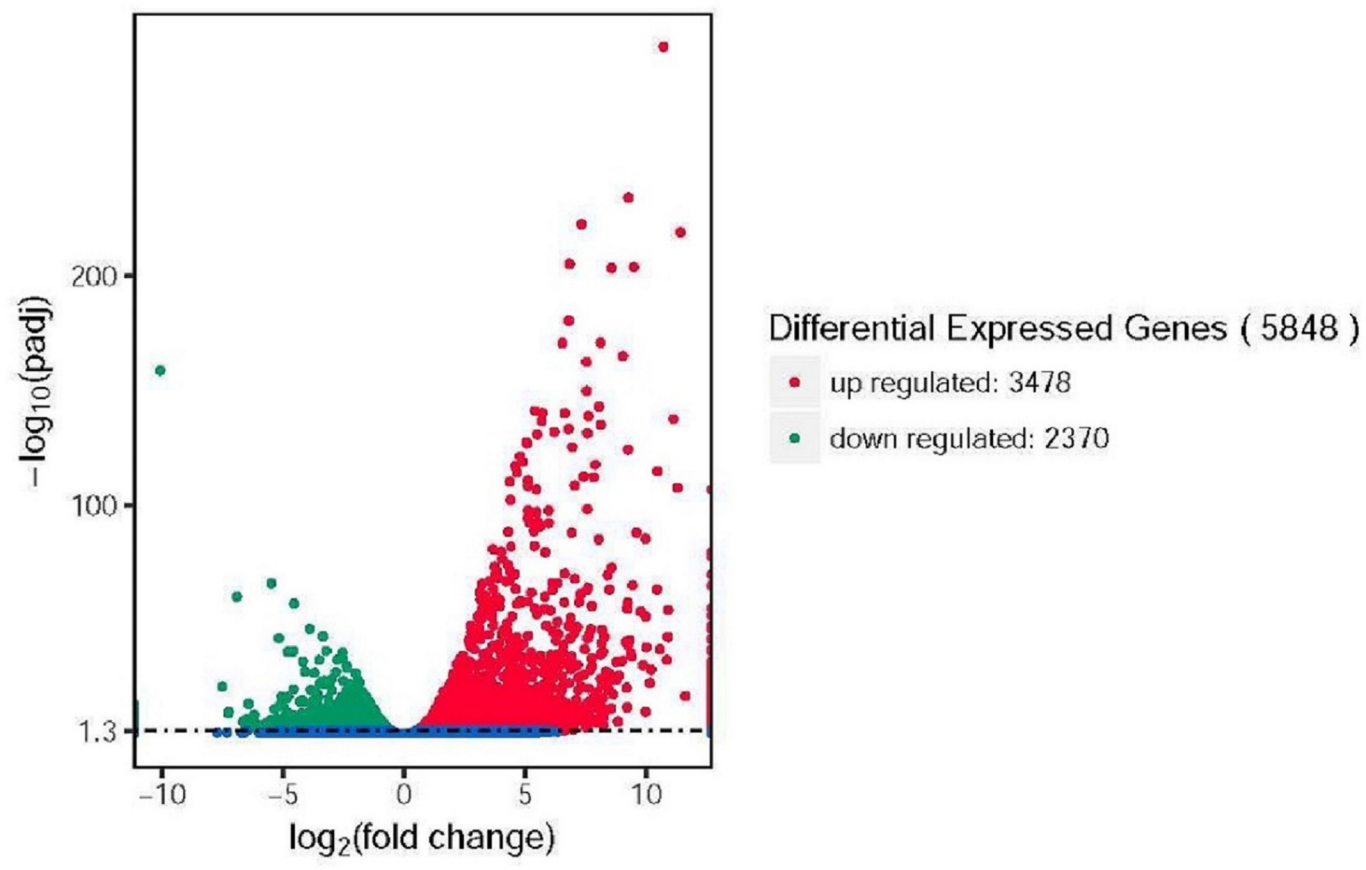
Analysis of differences in gene expression in the leaves of Lanzhou lily exposed to different temperatures between 20°C and −4°C. Note: Significantly differentially expressed up regulated and down regulated genes are indicated by red and green dots, respectively. Genes that are not significantly differentially expressed are indicated by blue dots. The abscissa represents the gene expression fold change in different samples. The ordinate represents the statistical significance of the difference in gene expression.

In this study, 5848 DEGs were identified in the Lanzhou lily transcriptome under subzero treatment (−4°C), among which 3478 DEGs were up regulated and 2370 DEGs were down regulated (Fig 2; S2 and S3 Tables). Using KEGG analysis, significant changes were found in genes related to protein kinase, protein phosphatase, carbon metabolism. Indole-3-glycerophosphate synthase, protein phosphatase, hexokinase, calc-binding protein, chlorophyll a/B-binding protein, and other related genes were significantly up regulated or down regulated.

### KEGG enrichment analysis of DEGs

**Fig 3:**
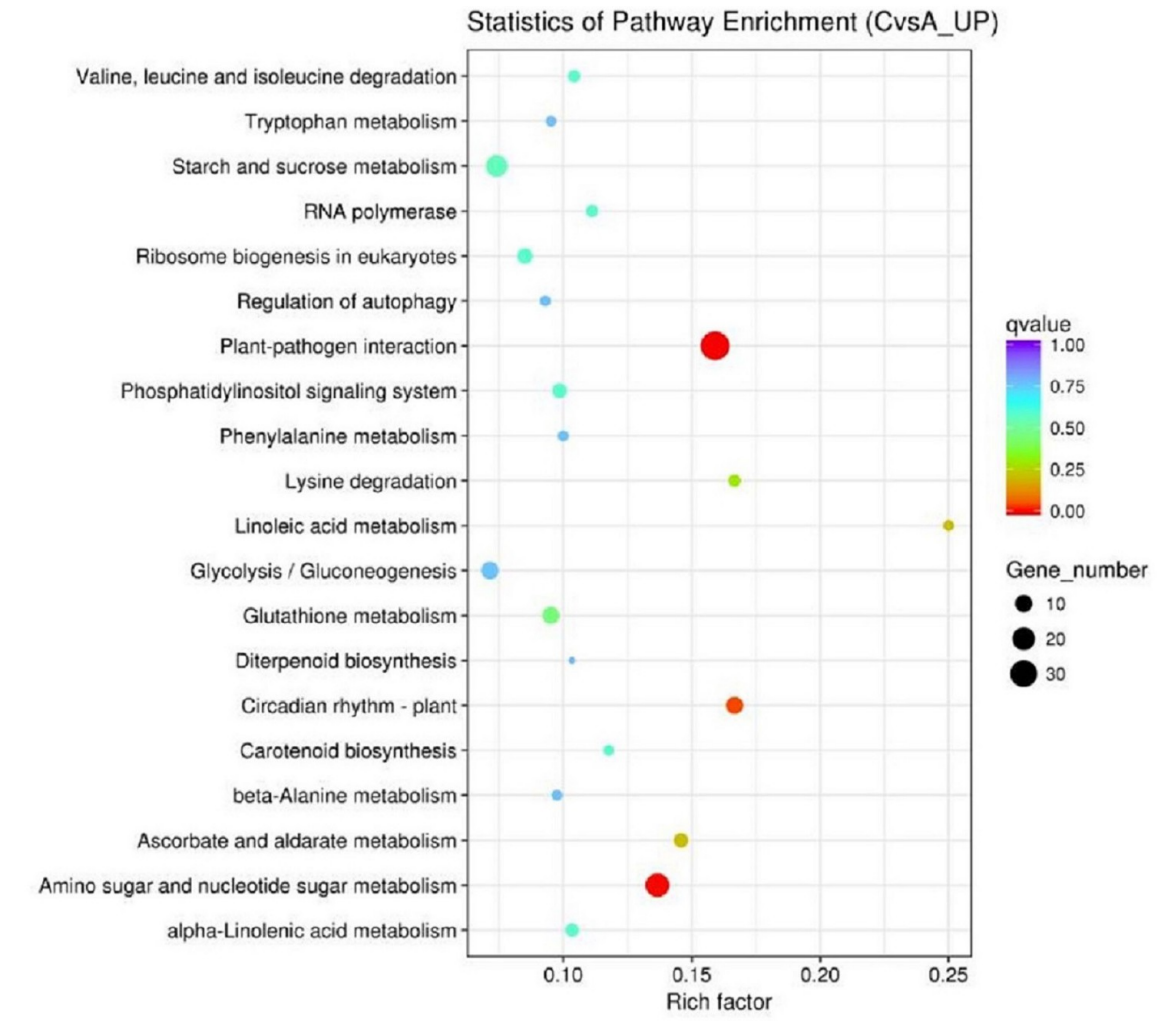
Kyoto Encyclopedia of Genes and Genomes (KEGG) analysis of gene up regulation induced by −4°C treatment in Lanzhou lily. Note: The size of the center point indicates the number of enriched genes; the larger the point, the more enriched genes. The color indicates the credibility of the enrichment, and the larger the q value, the more obvious the enrichment effect.

**Fig 4:**
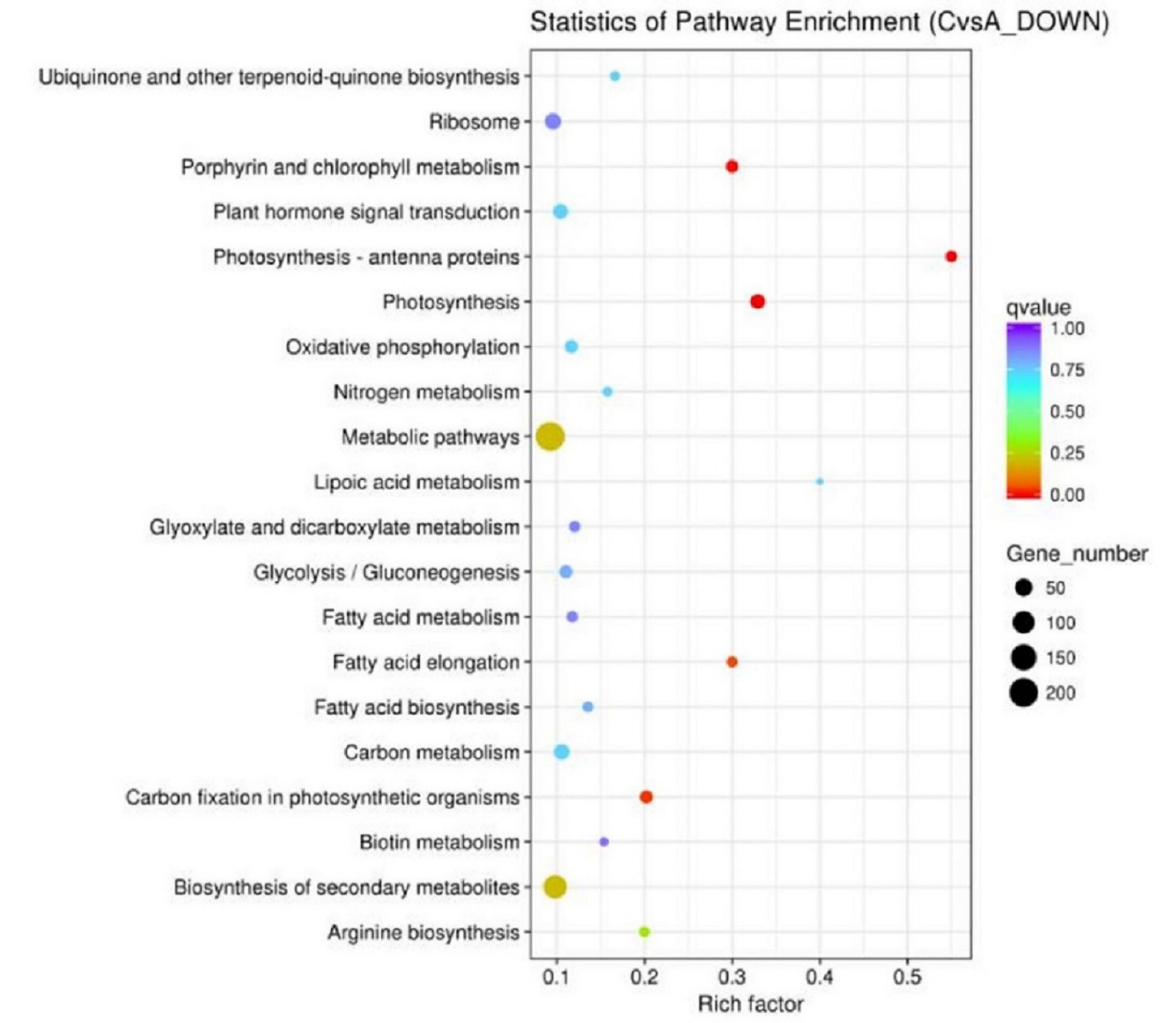
Kyoto Encyclopedia of Genes and Genomes (KEGG) analysis of gene down regulation induced by −4°C treatment in Lanzhou lily. Note: The size of the center point indicates the number of enriched genes; the larger the point, the more enriched genes. The color indicates the credibility of the enrichment, and the larger the q value, the more obvious the enrichment effect.

Pathways in which DEGs were significantly enriched relative to all annotated genes were identified. After pathway enrichment analysis, significant up regulated gene differences were found in significant abdominal muscle pathways, plant–pathogen interaction, amino acid and nucleotide sugar metabolism, plant circadian rhythm, and linoleic acid metabolism (Fig 3, S1 File). These up-regulated differential genes include *c156798_g2*, *c163371_g2* (calcium-dependent protein kinase, EC:2.7.11.1), *c166423_g2*, *c145559_g1* (respiratory burst oxidase, EC:1.6.3.- 1.11.1), *c162064_g1*, *c164545_g2*, *c166855_g1*(3.1027), *c145934_g1*(8.2812), *c101051_g1*(4.9356), *c144017_g1*(Inf), *c144017_g2*(Inf), *c159000_g1*(2.9702), *c140553_g1*(9.9651), *c170503_g1*(Inf), *c140013_g1*(3.7728), *c154815_g1*(Inf), *c173122_g1*(7.8456), *c168962_g1*(Inf), *c163915_g1*(Inf), *c156536_g2*(2.9685), c156536_g1(1.2021) (calmodulin), and *c147877_g2* (mitogen-activated protein kinase kinase 4/5, EC:2.7.12.2). These genes mainly regulate plant-athogen interaction, and the genes related to pathogen interaction are closely related to plant resistance. The significantly enriched pathways of down regulated DEGs included photosynthesis, photosynthesis-antenna proteins, porphyrins and chlorophyll metabolism, carbon fixation in photosynthetic organisms, and fatty acid production (Fig 4, S2 File).

### QRT-PCR verification

**Fig 5:**
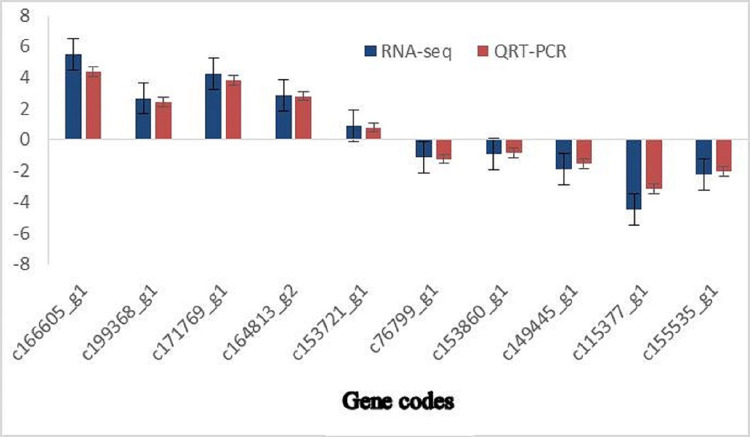
Validation of differentially expressed gene (DEG) data through quantitative real-time PCR (QRT-PCR) and RNA sequencing (RNA-seq). Note: The relative expression levels of the selected genes were normalized to the expression level of the actin gene. The blue and red bars represent the relative expression levels of DEGs in Lanzhou lily exposed to different treatments calculated using the RNA-seq and QRT-PCR methods, respectively. The normal treatment temperature was 20°C; the subzero treatment temperature was −4°C.

To verify the reliability of the RNA-seq results, 10 DEGs were randomly selected. These included five up regulated DEGs (*c166605_g1*, *c199368_g1*, *c171769_g1*, *c164813_g2*, and *c153721_g1*) and five down regulated DEGs (*c76799_g1*, *c153860_g1*, *c149445_g1*, *c115377_g1*, and *c155535_g1*), and Actin was used as the internal parameter for QRT-PCR verification. As shown in Fig 5, the expression degree of 10 genes under low-temperature stress differed, but the expression trend was consistent with the high-throughput sequencing results, indicating that the sequencing results were accurate and reliable (Fig 5).

### Expression levels of genes in response to low-temperature stress

**Fig 6:**
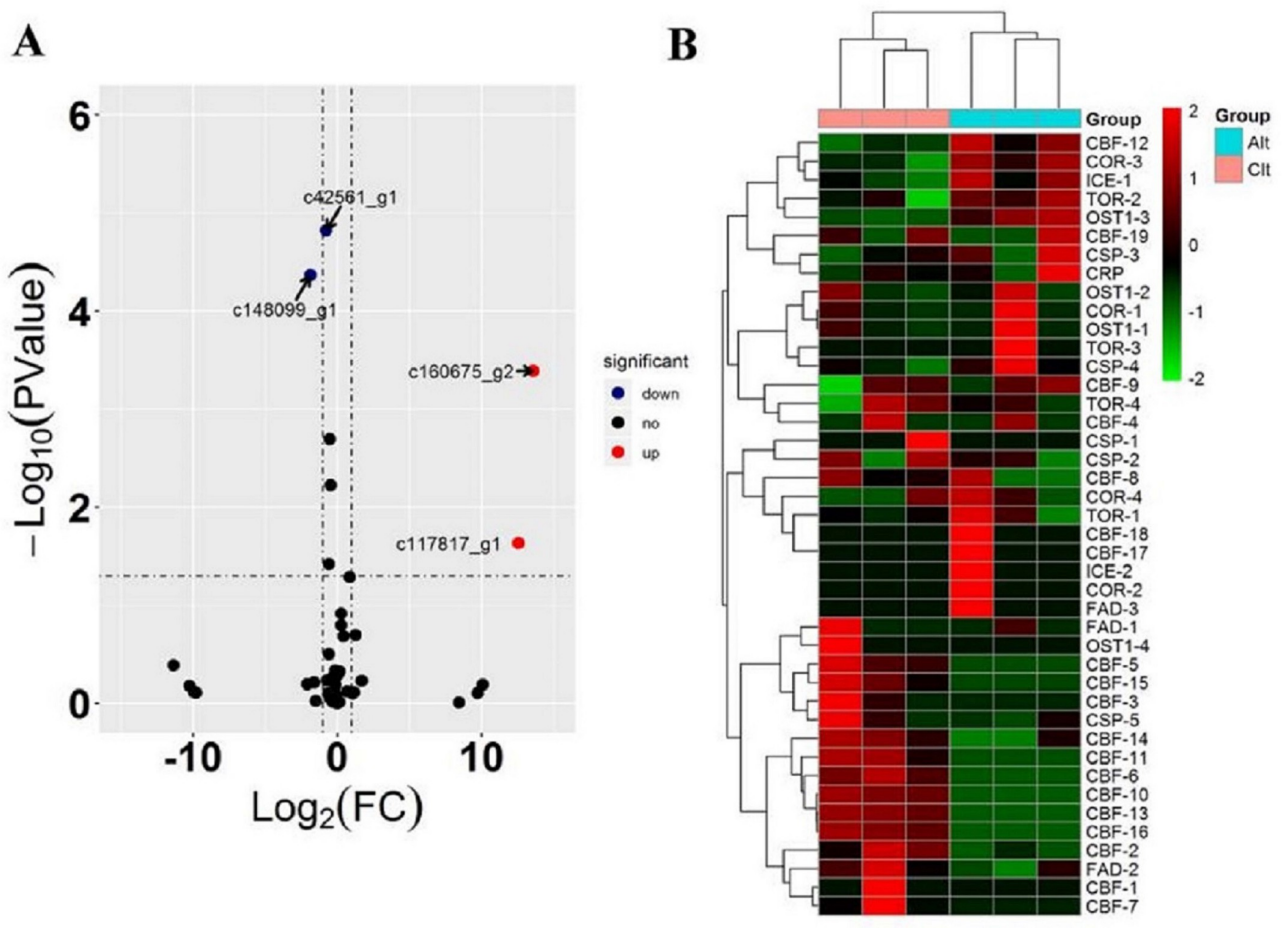
Expression pattern analysis of genes related to the cold stress response. Note: Scatter plot of differentially expressed genes (C vs. A). Red points represent up regulated genes with log2 (fold change) > 1 and adjusted p (q-value) < 0.05 (−log10 (q-value) ≥ 1.3). Green points represent down regulated genes with log2 (fold change) < −1 and q-value < 0.05 (−log10 (q-value) ≥ 1.3). Black points represent genes with no significant differences. Fold change = gene normalized expression of the control group/gene normalized expression of the treatment group. B: Heat map of genes with known functions related to the cold stress response. The up and down regulated genes are shown in red and green, respectively. The FPKM (Z-Score) from transcriptome data represents the gene expression level. FPKM, fragments per kilobase of exon per million reads mapped.

To identify key genes in the process of resistance to low-temperature stress in Lanzhou lily, genes participating in cold response (GO:0009409), cold response (GO:0050826; genes for GO:0009266), cold acclimation (GO:0009631), and cell response to cold (GO:0070417) were screened (Table 4). Analyzing the transcriptional changes of *c148099_g1*, *c42561_g1*, *c117817_g1*, and *c160675_g2* under low-temperature stress revealed that the genes *c148099_g1*, *c42561_g1*, *c117817_g1*, and *c160675_g2* showed significant differences (Fig 6A). The transcription levels of *c148099_g1* and *c42561_g1* decreased by 72% and 42%, respectively, under low-temperature stress. The genes *c117817_g1* and c160675_g2 were activated under low-temperature stress, and their expression levels were significantly up regulated 6010.58 and 12294.41 times, respectively (Table 3). Further annotation in the NR database showed that the genes *c148099_g1*, *c42561_g1*, *c117817_g1*, and *c160675_g2* (*OLEO3*) were stress-induced hydrophobic peptide, F-box protein 7, C2H2-type zinc finger protein, and Oleosin 18.2 kDa-like, respectively (Table 4). Therefore, it is speculate that these four genes can be regarded as the key genes in plant resistance to low-temperature stress. The *c117817_g1* gene is a transcription factor that has the potential function of regulating downstream resistance reactions; therefore, its function in resisting low-temperature stress in Lanzhou lily is of great research value.

**Table 4:** Functional list of differentially expressed genes that respond to cold stress.

Gene_ID	NR_Description	Gene Ontology	Biological Process Description
*c152295_g1*	Hypothetical protein CICLE_v10002973mg	GO:0050826	Response to freezing stress
*c160675_g2*	PREDICTED: oleosin 18.2 kDa-like	GO:0050826	Response to freezing stress
*c155141_g1*	Unnamed protein product	GO:0050826	Response to freezing stress
*c155141_g2*	PREDICTED: protein ESKIMO 1-like isoform X2	GO:0050826	Response to freezing stress
*c171385_g2*	Hypothetical protein POPTR_0010s19480g	GO:0050826	Response to freezing stress
*c163100_g1*	PREDICTED: actin-related protein 6-like isoform X2	GO:0009266	Response to temperature stimulus
*c167898_g3*	PREDICTED: MADS-box protein SVP-like	GO:0009266	Response to temperature stimulus
*c127438_g1*	Histone core	GO:0009266	Response to temperature stimulus
*c169166_g1*	RecName: Full = Molybdenum cofactor sulfurase; Short = MCS; Short = MOS; Short = MoCo sulfurase; AltName: Full = Molybdenum cofactor sulfurase-like protein 3; AltName: Full = Molybdenum cofactor sulfurtransferase	GO:0009266	Response to temperature stimulus
*c165660_g2*	PREDICTED: NADH-cytochrome b5 reductase-like protein-like, partial	GO:0009266	Response to temperature stimulus
*c65163_g1*	FAD/NAD (P) -binding oxidoreductase isoform 2	GO:0009266	Response to temperature stimulus
*c133988_g1*	PREDICTED: alpha-glucan water dikinase, chloroplastic	GO:0009631	Cold acclimation
*c187841_g1*	PREDICTED: alpha-glucan water dikinase, chloroplastic isoform X2	GO:0009631	Cold acclimation
*c230470_g1*	PREDICTED: NADH dehydrogenase [ubiquinone] iron-sulfur protein 4, mitochondrial-like	GO:0009631	Cold acclimation
*c83168_g1*	hypothetical protein MANES_13G110900	GO:0009631	Cold acclimation
*c84961_g1*	PREDICTED: cold shock protein 1-like	GO:0009631	Cold acclimation
*c130601_g1*	PREDICTED: mediator of RNA polymerase II transcription subunit 32	GO:0009631	Cold acclimation
*c28229_g1*	PREDICTED: DEAD-box ATP-dependent RNA helicase 24	GO:0070417	Cellular response to cold stress
*c131748_g1*	PREDICTED: galactinol synthase 1	GO:0070417	Cellular response to cold stress
*c170543_g8*	PREDICTED: probable histidine kinase 5 isoform X1	GO:0070417	Cellular response to cold stress
*c170801_g1*	PREDICTED: bifunctional purine biosynthesis protein purH	GO:0009409	Response to cold stress
*c143212_g1*	PREDICTED: F-box-like/WD repeat-containing protein TBL1XR1 isoform X2	GO:0009409	Response to cold stress
*c183880_g1*	PREDICTED: F-box-like/WD repeat-containing protein TBL1XR1-like	GO:0009409	Response to cold stress
*c241554_g1*	BnaA07g15960D	GO:0009409	Response to cold stress
*c233311_g1*	Arginine decarboxylase	GO:0009409	Response to cold stress
*c182050_g1*	Diacylglycerol kinase 2	GO:0009409	Response to cold stress
*c139694_g1*	CYSB	GO:0009409	Response to cold stress
*c142577_g1*	Cysteine proteinase inhibitor 12	GO:0009409	Response to cold stress
*c117817_g1*	C2H2-type zinc finger protein	GO:0009409	Response to cold stress
*c219654_g1*	Catalase, partial	GO:0009409	Response to cold stress
*c114432_g1*	PREDICTED: probable calcium-binding protein CML14	GO:0009409	Response to cold stress
*c148099_g1*	Stress-induced hydrophobic peptide	GO:0009409	Response to cold stress
*c22858_g2*	PREDICTED: bifunctional purine biosynthesis protein PurH-like isoform X2	GO:0009409	Response to cold stress
*c157461_g1*	PREDICTED: protein GIGANTEA	GO:0009409	Response to cold stress
*c42561_g1*	PREDICTED: F-box protein 7	GO:0009409	Response to cold stress
*c141286_g3*	PREDICTED: ribulose-phosphate 3-epimerase, chloroplastic	GO:0009409	Response to cold stress
*c155247_g1*	PREDICTED: ribulose-phosphate 3-epimerase, chloroplastic	GO:0009409	Response to cold stress
*c127149_g1*	RecName: Full = Hydrophobic protein LTI6A; AltName: Full = Low temperature-induced protein 6A	GO:0009409	Response to cold stress
*c162543_g1*	Predicted protein	GO:0009409	Response to cold stress
*c159054_g1*	Hypothetical protein CISIN_1g010418mg	GO:0009409	Response to cold stress
*c191917_g1*	PREDICTED: acyl-CoA-binding domain-containing protein 1-like isoform X1	GO:0009409	Response to cold stress
*c199362_g1*	PREDICTED: phospholipase D delta-like	GO:0009409	Response to cold stress
*c208592_g1*	PREDICTED: phospholipase D delta	GO:0009409	Response to cold stress
*c199151_g1*	F-box-like/WD repeat-containing protein TBL1XR1	GO:0009409	Response to cold stress
*c92858_g2*	RecName: Full = Ribulose-phosphate 3-epimerase, chloroplastic; AltName: Full = Pentose-5-phosphate 3-epimerase; Short = PPE; AltName: Full = R5P3E; Short = RPE; Flags: Precursor	GO:0009409	Response to cold stress
*c212057_g1*	Hypothetical protein CARUB_v10005847mg	GO:0009409	Response to cold stress
*c167534_g1*	PREDICTED: probable dimethyladenosine transferase isoform X1	GO:0009409	Response to cold stress
*c230248_g1*	Hypothetical protein CICLE_v10020970mg	GO:0009409	Response to cold stress
*c99584_g4*	PREDICTED: ubiquinol oxidase 1a, mitochondrial-like	GO:0009409	Response to cold stress
*c237822_g1*	PREDICTED: F-box-like/WD repeat-containing protein TBL1XR1-like	GO:0009409	Response to cold stress
*c158094_g1*	Pre-mRNA-processing factor 6	GO:0009409	Response to cold stress

In addition, among genes that respond to low-temperature stress, such as *CBF*, *COR*, *ICR*, and *TOR* (Fig 6B; Table 5), the *CBF* family genes were significantly affected in the process of resistance to low-temperature stress in lily. A total of 10 *CBF* family genes showed differential expression when exposed to low-temperature stress. Except for the *c166871_g1* gene, the other nine genes were significantly activated, and their expression levels increased when Lanzhou lily was exposed to low-temperature stress. Therefore, based on the above results, it is speculated that the *CBF* family genes and C2H2 type transcription factor *c117817_g1* play an important regulatory role in the response of Lanzhou lilies to low-temperature stress and that they are widely involved in the response pathway of Lanzhou lilies to low-temperature stress.

**Table 5:** Expression characteristics of the *CBF* gene family under cold stress. p < 0.05 is considered statistically significant.

Gene_ID	NR_Description	ID	log2FoldChange	p-Value
*c123982_g2*	PREDICTED: dehydration-responsive element-binding protein 3-like	*CBF-2*	3.09738	0.014174
*c140567_g1*	PREDICTED: dehydration-responsive element-binding protein 1F-like	*CBF-5*	16.08889	2.38E−11
*c147067_g1*	Dehydration-responsive element-binding protein 1F	*CBF-6*	17.65592	2.17E−49
*c162057_g1*	PREDICTED: dehydration-responsive element-binding protein 1F-like	*CBF-10*	14.53353	2.7E−07
*c164032_g1*	C-repeat/dehydration-responsive element-binding factor 2	*CBF-11*	10.15465	2.02E−25
*c166871_g1*	Dehydration-responsive element-binding protein 3	*CBF-12*	−1.79337	0.002434
*c167721_g2*	Dehydration-responsive element binding transcription factor 1B	*CBF-13*	11.39476	5E−224
*c169240_g5*	PREDICTED: dehydration-responsive element-binding protein 1F	*CBF-14*	2.880757	0.000667
*c170256_g2*	PREDICTED: dehydration-responsive element-binding protein 1B-like	*CBF-15*	9.965905	1.81E−12
*c174313_g1*	Dehydration-responsive element binding transcription factor 1B	*CBF-16*	10.70369	1E−305

### Analysis of the interaction regulatory network of genes responding to low-temperature stress

**Fig 7:**
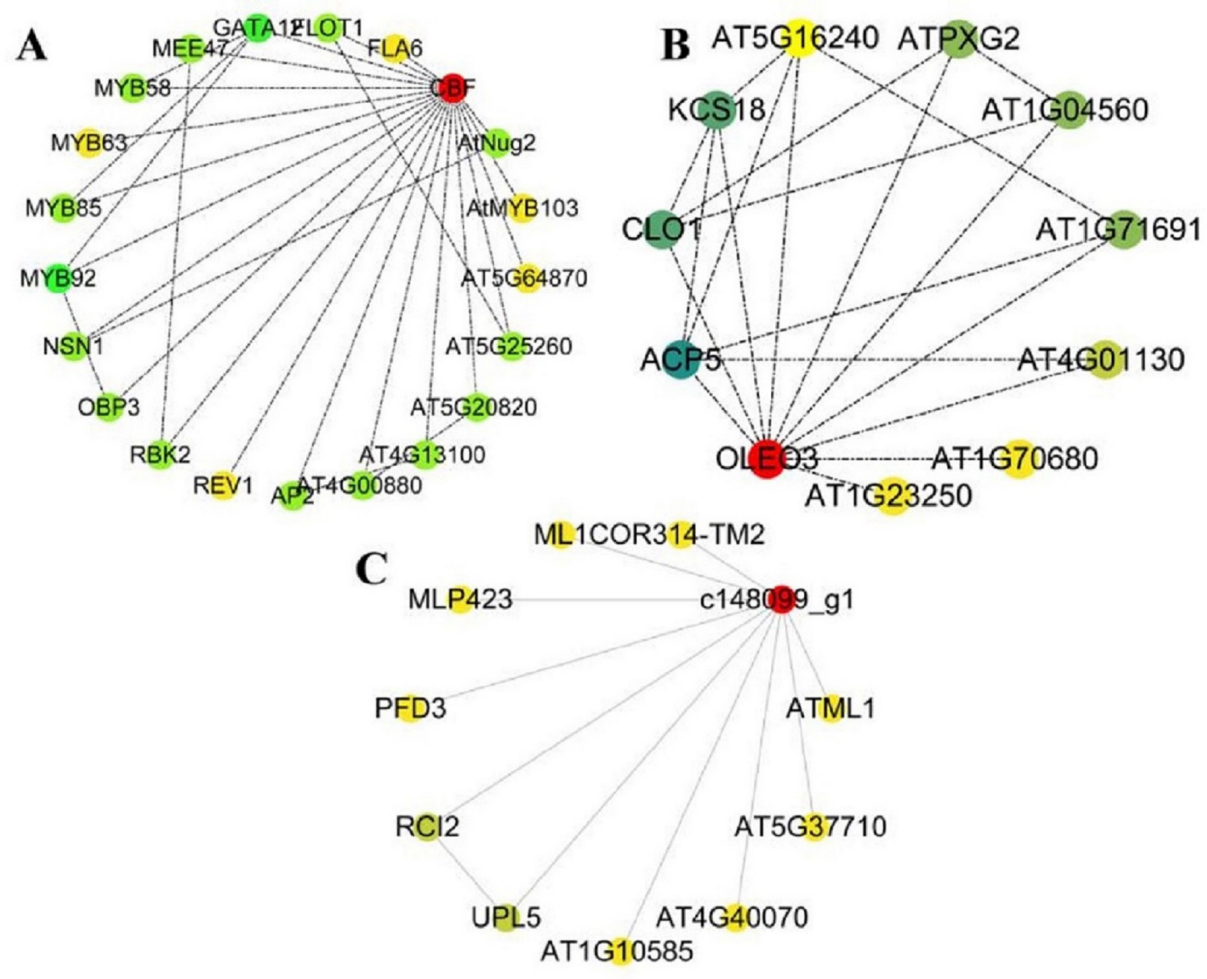
Regulation and interaction network of genes related to cold stress. Note: Each circle represents an individual gene, and the dotted lines between the two genes indicate an interaction between them. The color represents the weight of the corresponding gene in this interaction network, and the red represents the core gene of this interaction network.

To reveal the role of these key genes in the plant response to low-temperature stress, the regulatory interaction network of these genes was further analyzed. Genes in the *CBF* family play an indispensable role in their related interaction networks. Many genes regulated by *CBF* have functions related to plant stress resistance, such as MYB family transcription factors [41] (Table 6). As shown in Fig 7A, *CBF* genes exhibited potential interaction functions with many MYB transcription factors and could regulate each other. Therefore, it is suggested that changes in the *CBF* gene expression level can further affect the expression level of MYB transcription factors and thus play a role in the process of resistance to low-temperature stress in Lanzhou lily.

**Table 6:** GO enrichment analysis of genes involved in the interaction regulation network of the *CBF* gene family with an FDR value > 0.05.

Term ID	Term Description	Observed Gene Count	False Discovery Rate
GO:0005634	Nucleus	7	0.0081
GO:0008134	Transcription factor binding	4	1.97E−05
GO:0003677	DNA binding	6	0.0002
GO:2000652	Regulation of secondary cell wall biogenesis	2	0.0018
GO:0050794	Regulation of cellular process	7	0.0019
GO:0050896	Response to stimulus	6	0.0126
GO:0071554	Cell wall organization or biogenesis	2	0.0391
GO:0009808	Lignin metabolic process	2	0.0019
GO:0009725	Response to hormone	4	0.0046
GO:0009416	Response to light stimulus	3	0.0033

The GO functional enrichment analysis of genes in the regulatory interaction network involved in *CBF* revealed that stress-related functions, including “regulation of secondary cell wall biogenesis”, “regulation of cell processes”, “stimulus response”, “cell wall organization or biogenesis”, “lignin metabolic processes”, “hormone response”, and “photostimulation response”, were significantly enriched. Among them, “cell wall synthesis”, “stress response”, “lignin metabolism”, and “hormone function” played an important role in the plant’s response to low temperatures. It is speculated that these functions protect tissues from low-temperature damage during the response of Lanzhou lily to low temperatures.

The results of the regulatory interaction network of the *c160675_g2* (*OLEO3*) gene [42] showed (Fig 7B) that the functions of genes with potential interaction with this gene were mainly related to “calcium ion binding”, “electron transport chain”, “respiratory electron transport chain”, “lipid metabolism process”, “ATP metabolism process”, “stress response”, and other calcium ions and genes related to energy metabolism (Table 7). In addition, similar to the *CBF* gene family, genes related to membrane structure and hormones, including “cytoplasmic part”, “membrane”, and “linoleic acid cyclooxygenase activity” were affected by the *OLEO3* gene [43] (Table 7). Therefore, it is speculated that the *OLEO3* gene functions by regulating calcium signaling and energy metabolism in plants and helps plants resist low-temperature stress. In addition, the *OLEO3* gene can assist the *CBF* gene [44] to help plants reintegrate cell membrane structures when stressed by low temperatures and further activate downstream reactions by regulating hormone synthesis to enhance plant resistance to low temperatures. However, in the interaction network analysis of the *c148099_g1* gene (Fig 7C), no interaction was found between genes with stress resistance functions and the *c148099_g1* gene.

**Table 7:** GO enrichment analysis of genes involved in the interaction regulation network of *OLEO3* with an FDR value > 0.05.

Term ID	Term Description	Observed Gene Count	False Discovery Rate
GO:0031090	Organelle membrane	13	5.57E−10
GO:0016020	Membrane	15	1.65E−06
GO:0044444	Cytoplasmic part	15	6.02E−06
GO:0071614	Linoleic acid epoxygenase activity	2	2.13E−05
GO:0005509	Calcium ion binding	2	0.039
GO:0022900	Electron transport chain	6	9.78E−08
GO:0022904	Respiratory electron transport chain	4	1.07E−06
GO:0006629	Lipid metabolic process	7	2.33E−05
GO:0046034	ATP metabolic process	3	0.00065
GO:0006950	Response to stress	6	0.0484

According to the above results, we believe that in the face of low-temperature stress, edible lily can stimulate *c160675_g2* (*OLEO3*) and *CBF* family [45] genes, thereby regulating the changes in the cell membrane, energy metabolism, and the synthesis of hormones, lignin, and other secondary metabolites, thereby affecting the signaling pathway of hormones and calcium regulation. Thus, the *c160675_g2* (*OLEO3*) gene effectively activates the downstream transcription factor to protect plants from hypothermia damage.

### Interaction regulatory network and gene function analysis of C2H2 transcription factor c117817_g1 (ZFP)

**Fig 8:**
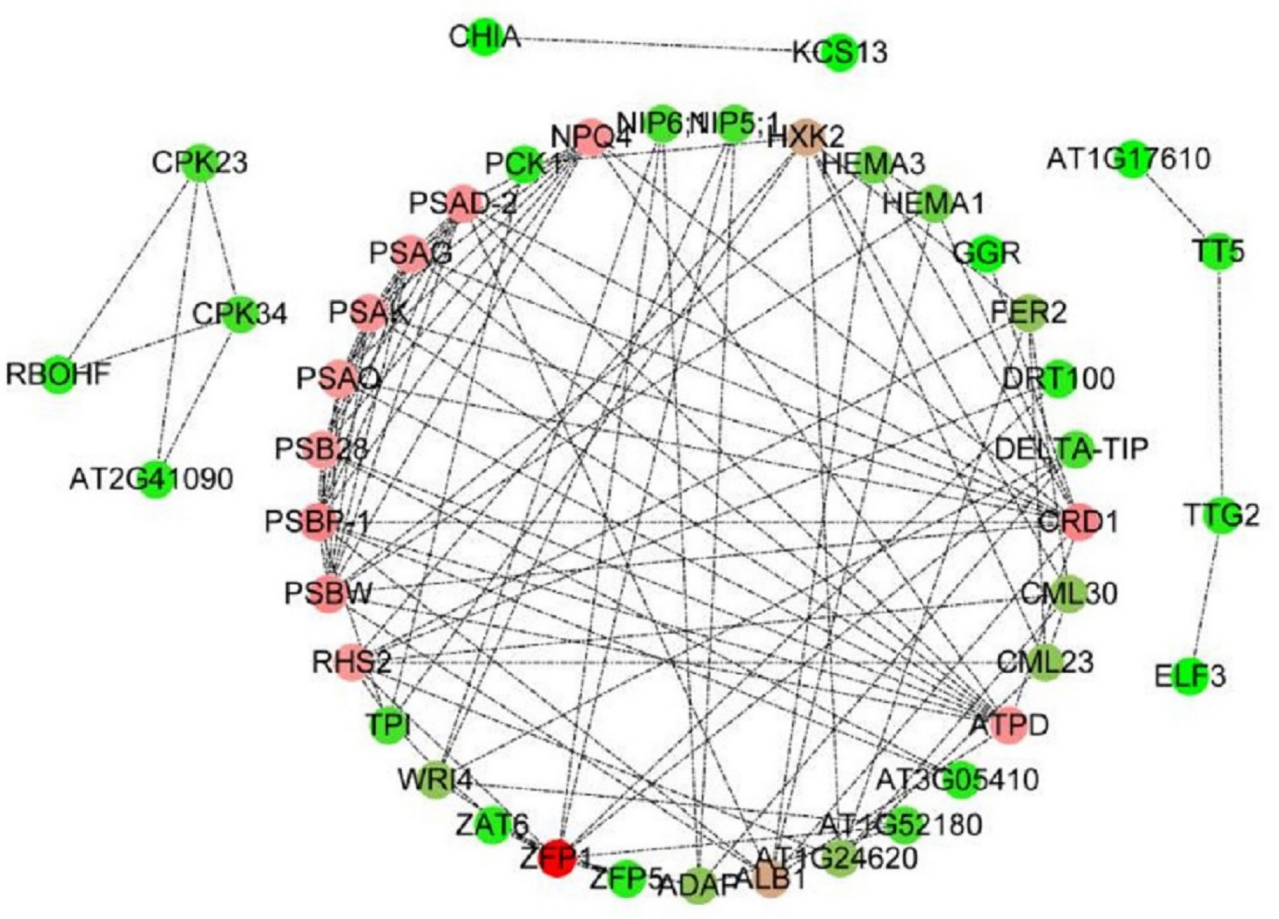
Analysis of the interaction regulation network of C2H2 transcription factor *c117817_g1* (*ZFP*). Note: Each circle represents an individual gene, and the dotted lines between two genes indicate an interaction between them. The color represents the weight of the corresponding gene in this interaction network, and red represents the core gene of this interaction network.

To study the function of C2H2 transcription factor [46] *c117817_g1* (*ZFP*) under low-temperature stress in lily, this work analyzed the interaction regulatory network between the genes related to low-temperature stress in edible lily and the *c117817_g1* (*ZFP*) gene (Fig 8). As shown in Fig 8, the *c117817_g1* (*ZFP*) gene and genes related to the response to low-temperature stress in these plants had a close regulatory interaction. Moreover, the *c117817_g1* (*ZFP*) gene held a core position in this interaction network, and many genes were regulated by the C2H2 transcription factor *c117817_g1* (*ZFP*). Thus, the *c117817_g1* (*ZFP*) gene plays an indispensable role in this plant interaction regulatory network [47].

Further GO functional analysis of the genes involved in this interaction regulatory network revealed that these genes play a role in the photosystem, membrane, calcium ion binding, stress response, hormonal response, cold stress, abiotic stress response, and ABA response (Table 8). Among them, such genes are directly involved in the response to cold stress in Lanzhou lilies. Moreover, genes related to the cell membrane and calcium ion binding appeared in this interaction regulatory network, which was affected by the differential expression of the *c117817_g1* (*ZFP*) gene.

**Table 8:** GO enrichment analysis of genes involved in the interaction regulation network of C2H2-type transcription factor *c117817_g1* (*ZFP*) with an FDR value < 0.05.

Term ID	Term Description	Gene Count	FDR
GO:0009523	Photosystem	9	9.96E−07
GO:0009534	Chloroplast thylakoid	12	6.78E−10
GO:0016020	Membrane	30	5.93E−08
GO:0005509	Calcium ion binding	12	4.58E−12
GO:0004683	Calmodulin-dependent protein kinase activity	2	0.0318
GO:0015979	Photosynthesis	12	1.15E−11
GO:0050896	Response to stimulus	32	4.67E−09
GO:0009725	Response to hormone	16	1.14E−06
GO:0009409	Response to cold stress	8	1.47E−05
GO:0009628	Response to abiotic stimulus	15	1.81E−05
GO:0015995	Chlorophyll biosynthetic process	4	3.58E−05
GO:0009755	Hormone-mediated signaling pathway	10	5.56E−05
GO:0009737	Response to abscisic acid	8	0.00011
GO:1901700	Response to oxygen-containing compound	12	0.00017
GO:0015250	Water channel activity	2	0.0011

Further analysis revealed that water channel activity and genes involved in the ABA reaction were also affected. These results indicate that plants can regulate ABA-related signaling pathways and change the activity of water channels in response to low-temperature stress. Therefore, ABA and water channel changes play an important role in the plant’s response to low-temperature stress. These genes are mainly involved in metabolic pathways such as photosynthesis (Fig 4), plant circadian rhythm, and linoleic acid metabolism (Fig 3). Therefore, it is speculated that C2H2 transcription factor *c117817_g1* (*ZFP*) can enhance the response and resistance of Lanzhou lily to low-temperature stress by regulating the metabolic processes of photosynthesis, circadian rhythm, and linoleic acid metabolism.

## Discussion

Adverse growth environments, especially the stress of temperatures below zero, are a natural disaster that plants often encounter during their growth and development. In this study, an advanced sequencing platform, the Illumina 4000 high-throughput sequencing platform, was used to sequence Lanzhou lily treated at normal (20°C) and low temperatures (−4°C).

According to the results of the KEGG enrichment analysis, 25 DEGs participated in pathogen interaction pathways, 10 participated in amino acid and nucleotide sugar metabolism pathways, eight participated in plant circadian rhythm pathways, 15 participated in porphyrin regulation of chlorophyll metabolism pathways, and 16 participated in photosynthesis pathways. A total of 10 DEGs regulated the photosynthesis-antenna protein pathway, 13 regulated the carbon fixation pathway in photosynthetic organisms, and nine regulated the fatty acid elongation pathway.

Further analysis showed that the *OLEO3* gene, nine *CBF* family genes, and the C2H2 transcription factor *c117817_g1* (*ZFP*) played an indispensable role in the response of edible lilies to low-temperature stress. These genes are mainly involved in the regulation of plant photosynthesis, calcium ion binding, membrane structure formation, and the ABA-dominated signaling pathway, having a function in the process of Lanzhou lily’s resistance to low-temperature stress.

Under low-temperature stress, *CBF* family genes showed significant activation, and their transcription levels were greatly improved. According to interaction network analysis, these genes interacted with *MYB* family genes, regulated the plant secondary cell wall and lignin synthesis, and affected hormone-related responses to enhance plant resistance to stress, such as ultra-low temperatures, and reduce plant tissue damage. Studies have shown that the *CBF* gene can interact with *ATMYB14* in Arabidopsis, thus participating in freezing resistance and playing an important role in the plant response to cold stress [48–50]. In addition, the regulation effect of the typical *R2R3-MY*B transcription factor *MYB88* on plant cold tolerance dependent on the *CBF* gene has been confirmed in apples [51], and the activation of this gene can promote the accumulation of plant anthocyanins and enhance resistance to low-temperature and oxidative stress [52]. This study also found that highly expressed *CBF* family genes interacted with multiple MYB transcription factors in Lanzhou lily, thereby affecting their expression and downstream resistance. This is similar to the findings reported above.

The C2H2 transcription factor *c117817_g1* (*ZFP*) gene also played an important role in the regulation of low-temperature stress in Lanzhou lily. According to the results of this study, this gene mainly interacts with genes involved in processes including photosynthesis, calcium ion binding, membrane structure formation, and ABA-dominated signaling pathways, and other processes in plants. Low-temperature stress research has shown that photosynthesis, ion balance, regulated dehydration, and other stress and hormone regulation pathways in plants are affected when they are subjected to low-temperature stress, enabling plants to resist low-temperature stress [53]. In addition, under low-temperature stress, the imbalance of the light absorption capacity and light dissipation capacity of plants leads to the destruction of Photosystem II (PS II) and the inhibition of photosynthesis [54]. In low-temperature-tolerant crops, such as cucumber [55], tobacco [56], and rice [57], light intensity and ambient temperature can affect chloroplasts and initiate signal transduction at low temperatures, thus coordinating gene expression between protoplasts and nuclei to adapt to changes in ambient temperatures [58]. This result is consistent with the photosynthesis inhibition found in the transcriptome of Lanzhou lily under low-temperature stress in this experiment, indicating that Lanzhou lily can also enhance its resistance to low-temperature stress by inhibiting photosynthesis. Lanzhou lily also resist low-temperature stress by adjusting the ABA signaling pathway to adapt to low-temperature changes. In this regard, it has been shown that cells can jointly maintain the balance of the cell state (i.e., the inflow and outflow are equal) through ABA and osmoregulatory substances (such as soluble sugars and proline) to enable plants to resist a certain degree of low-temperature damage [59]. Moreover, an increase in ABA content at low temperatures can promote the transcriptional level of CBF genes [45]. Although it has been demonstrated that such transcription factors can respond to low-temperature stress in plants [60], research in this area is still insufficient, and further study is needed.

According to the results of this study, The *c160675_g2* (*OLEO3*) gene is mainly regulated by calcium ion binding, electron transport chain, respiratory electron transport chains, lipid metabolic processes, ATP metabolic process, response to stress and energy metabolic processes; all of these regulate the cold resistance of Lanzhou lily. Therefore, the *OLEO3* gene can help Lanzhou lily withstand low-temperature stress by regulating plant calcium signaling and energy metabolism. In addition, under ultra-low temperature stress, the *OLEO3* gene can interact with *CBF* gene to further activate downstream reactions and enhance plant resistance to low temperature by integrating cell membrane structure and regulating hormone synthesis. In previous reports, Marta Gliwicka *et al*. studied the expression of the *Arabidopsis* seed storage gene *OLEO3* in somatic embryogenic culture, and no correlation was reported between *OLEO3* and plant cold resistance [61]. The role of the *c160675_g2* (*OLEO3*) gene in improving the cold resistance of lily can be focused on in subsequent studies.

The above genes related to low-temperature stress in Lanzhou lily are considered targets that can improve plant resistance to low temperatures. The function of these genes in Lanzhou lily under low-temperature stress and the reasons for their differential expression need to be further studied at the molecular level to explore the functions and effects of these genes under low-temperature stress and to provide theoretical support and direction for cultivating low-temperature-resistant Lily.

## Conclusions

In total, 8558 DEGs were obtained by KEGG pathway analysis following transcriptome sequencing of the Lanzhou lily. After enrichment analysis, genes related to protein kinase, protein phosphatase, carbon metabolism, and ABA were identified as the main research objects for studying the cold response mechanism in Lanzhou lily. QRT-PCR analysis showed that the expression trends of 10 randomly selected DEGs were consistent with the high-throughput sequencing results. In addition, the interaction regulatory network, GO enrichment analysis, and gene function analysis showed that the pathways closely related to cold resistance in Lanzhou lily included photosynthetic and metabolic pathways. Key genes related to cold resistance were screened and predicted, namely gene *OLEO3*, nine *CBF* family genes, and C2H2 transcription factor *c117817_g1* (*ZFP*), which occupied a key position in the gene interaction network and interacted with multiple genes to regulate freezing resistance in Lanzhou lily. These findings lay a foundation for revealing the molecular mechanism of freezing resistance in Lanzhou lily. In the follow-up study, we will use gene silencing test to verify whether *OLEO3* gene is the key gene for cold resistance of Lanzhou lily.

## Supporting information

S1 Table Quality evaluation of RNA-seq data. (XLSX)

S2 Table Significantly enriched up-regulated DEGs of CvsA. (XLSX)

S3 Table Significantly enriched down-regulated DEGs of CvsA. (XLSX)

S1 File Up-regulated differential genes and enrichment pathways. (ZIP)

S2 File Down-regulated differential genes and enrichment pathways. (ZIP)
