# Supplementary material for: Key anti-freeze genes and pathways of Lanzhou lily (Lilium davidii, var. unicolor) during the seedling stage
Source: PLoS One. 2024 Mar 21;19(3):e0299259. doi: 10.1371/journal.pone.0299259 (PMC10956819; doi:10.1371/journal.pone.0299259)
Supplement: S1 File — (ZIP) [file pone.0299259.s004.zip › S1 Zip/CvsA_UP.html]

Pathway Enrichment

  

# The most enriched pathway terms

Statistic method: hypergeometric test

FDR correction method: Benjamini and Hochberg

| Term | Sample number | Background number | P-value | Corrected P-value | Gene\_id | KEGG\_ID/KO | Entrez ID | Gene name |
| --- | --- | --- | --- | --- | --- | --- | --- | --- |
| Plant-pathogen interaction | 35 | 220 | 3.86854902142e-09 | 3.48169411928e-07 | c173897\_g7 c162064\_g1 c144017\_g1 c144017\_g2 c159000\_g1 c154815\_g1 c140553\_g1 c173122\_g1 c147918\_g2 c166855\_g1 c145934\_g1 c152058\_g1 c152058\_g2 c145559\_g1 c168165\_g3 c156536\_g2 c156536\_g1 c153491\_g1 c170503\_g1 c156798\_g2 c168962\_g1 c140013\_g1 c153028\_g1 c166423\_g2 c153815\_g1 c164545\_g2 c113196\_g1 c163915\_g1 c101051\_g1 c163371\_g2 c147831\_g1 c152643\_g2 c166605\_g3 c152643\_g1 c147877\_g2 | egu:105051034 egu:105041258 egu:105037682 egu:105037682 egu:105037682 egu:105039372 egu:105037682 egu:105039372 egu:105052456 egu:105057333 egu:105057333 egu:105034665 egu:105034665 egu:105042006 egu:105044995 egu:105046031 egu:105046031 egu:105051034 egu:105037682 egu:105054415 egu:105039372 egu:105038625 egu:105047887 egu:105060351 egu:105045551 egu:105042206 egu:105057654 egu:105039372 egu:105057333 egu:105052035 egu:105045551 egu:105044758 egu:105044758 egu:105044758 egu:105050781 | 105051034 105041258 105037682 105037682 105037682 105039372 105037682 105039372 105052456 105057333 105057333 105034665 105034665 105042006 105044995 105046031 105046031 105051034 105037682 105054415 105039372 105038625 105047887 105060351 105045551 105042206 105057654 105039372 105057333 105052035 105045551 105044758 105044758 105044758 105050781 |  |
| Amino sugar and nucleotide sugar metabolism | 22 | 161 | 2.6788070292e-05 | 0.00120546316314 | c72023\_g1 c132414\_g1 c167554\_g2 c164898\_g1 c125968\_g1 c166880\_g1 c154320\_g1 c163736\_g1 c113669\_g1 c237479\_g1 c161991\_g2 c161991\_g1 c153668\_g1 c153668\_g2 c171764\_g2 c172816\_g3 c172816\_g4 c159575\_g2 c164916\_g2 c122873\_g1 c168684\_g1 c106411\_g1 | egu:105046676 egu:105039562 egu:105055983 egu:105049059 egu:105059690 egu:105045517 egu:105059691 egu:105042572 egu:105047535 egu:105047536 egu:105047535 egu:105047536 egu:105050762 egu:105050762 egu:105059690 egu:105060320 egu:105060220 egu:105047457 egu:105054659 egu:105055983 egu:105045517 egu:105057669 | 105046676 105039562 105055983 105049059 105059690 105045517 105059691 105042572 105047535 105047536 105047535 105047536 105050762 105050762 105059690 105060320 105060220 105047457 105054659 105055983 105045517 105057669 |  |
| Circadian rhythm - plant | 10 | 60 | 0.00113039987769 | 0.0339119963308 | c131089\_g1 c174283\_g3 c156146\_g2 c152959\_g1 c153783\_g1 c151010\_g1 c167910\_g3 c144654\_g1 c171563\_g1 c156146\_g1 | egu:105041005 egu:105041113 egu:105036385 egu:105059424 egu:105054824 egu:105045005 egu:105058499 egu:105041005 egu:105036385 egu:105047639 | 105041005 105041113 105036385 105059424 105054824 105045005 105058499 105041005 105036385 105047639 |  |
| Linoleic acid metabolism | 4 | 16 | 0.011234006123 | 0.206755769714 | c173762\_g1 c170715\_g1 c157926\_g1 c162165\_g1 | egu:105053112 egu:105041807 egu:105053112 egu:105056718 | 105053112 105041807 105053112 105056718 |  |
| Ascorbate and aldarate metabolism | 7 | 48 | 0.0114864316508 | 0.206755769714 | c167554\_g2 c168307\_g1 c148031\_g1 c164787\_g1 c224426\_g1 c146039\_g2 c122873\_g1 | egu:105055983 egu:105038499 egu:105042090 egu:105051810 egu:105045793 egu:105055920 egu:105055983 | 105055983 105038499 105042090 105051810 105045793 105055920 105055983 |  |
| Lysine degradation | 5 | 30 | 0.0196042311123 | 0.294063466684 | c148031\_g1 c159372\_g1 c165534\_g2 c154026\_g1 c164787\_g1 | egu:105042090 egu:105053701 egu:105043130 egu:105056313 egu:105051810 | 105042090 105053701 105043130 105056313 105051810 |  |
| Glutathione metabolism | 10 | 105 | 0.0352670579951 | 0.453433602794 | c173732\_g2 c162564\_g1 c163752\_g1 c145216\_g1 c158909\_g1 c152293\_g1 c160646\_g1 c224426\_g1 c165975\_g1 c213021\_g1 | egu:105037855 egu:105054171 egu:105058473 egu:105044685 egu:105032151 egu:105032151 egu:105056588 egu:105045793 egu:105037855 egu:105043366 | 105037855 105054171 105058473 105044685 105032151 105032151 105056588 105045793 105037855 105043366 |  |
| Starch and sucrose metabolism | 18 | 243 | 0.0495566771629 | 0.557512618083 | c174721\_g1 c167554\_g2 c153668\_g2 c79219\_g1 c173495\_g2 c168535\_g1 c174203\_g1 c144134\_g1 c111106\_g1 c106411\_g1 c122873\_g1 c170749\_g3 c163448\_g1 c132414\_g1 c159060\_g1 c152671\_g2 c152671\_g1 c153668\_g1 | egu:105043800 egu:105055983 egu:105050762 egu:105043204 egu:105060892 egu:105041389 egu:105036866 egu:105041628 egu:105043800 egu:105057669 egu:105055983 egu:105045199 egu:105055201 egu:105039562 egu:105043204 egu:105056861 egu:105056861 egu:105050762 | 105043800 105055983 105050762 105043204 105060892 105041389 105036866 105041628 105043800 105057669 105055983 105045199 105055201 105039562 105043204 105056861 105056861 105050762 |  |
| Phosphatidylinositol signaling system | 7 | 71 | 0.0622403834569 | 0.585161868152 | c166846\_g1 c101051\_g1 c166855\_g1 c145934\_g1 c152208\_g2 c168165\_g3 c148900\_g2 | egu:105038590 egu:105057333 egu:105057333 egu:105057333 egu:105056668 egu:105044995 egu:105054063 | 105038590 105057333 105057333 105057333 105056668 105044995 105054063 |  |
| alpha-Linolenic acid metabolism | 6 | 58 | 0.0684717309997 | 0.585161868152 | c157926\_g1 c173723\_g1 c170715\_g1 c162165\_g1 c156705\_g2 c173762\_g1 | egu:105053112 egu:105038559 egu:105041807 egu:105056718 egu:105059131 egu:105053112 | 105053112 105038559 105041807 105056718 105059131 105053112 |  |
| RNA polymerase | 5 | 45 | 0.0741978075786 | 0.585161868152 | c165193\_g1 c166229\_g1 c171631\_g6 c173969\_g2 c173363\_g9 | egu:12079459 egu:105047342 egu:105034397 egu:12079460 egu:12079461 | 12079459 105047342 105034397 12079460 12079461 |  |
| Valine, leucine and isoleucine degradation | 5 | 48 | 0.0902541808 | 0.585161868152 | c148031\_g1 c149905\_g2 c164787\_g1 c154026\_g1 c156705\_g2 | egu:105042090 egu:105053889 egu:105051810 egu:105056313 egu:105059131 | 105042090 105053889 105051810 105056313 105059131 |  |
| Ribosome biogenesis in eukaryotes | 8 | 94 | 0.0903219640613 | 0.585161868152 | c168781\_g1 c170819\_g1 c152959\_g1 c167910\_g3 c166893\_g1 c165011\_g1 c145725\_g1 c143131\_g1 | egu:105046077 egu:105037954 egu:105059424 egu:105058499 egu:105042999 egu:105032038 egu:105043116 egu:105053596 | 105046077 105037954 105059424 105058499 105042999 105032038 105043116 105053596 |  |
| Carotenoid biosynthesis | 4 | 34 | 0.0910251794903 | 0.585161868152 | c173485\_g1 c173509\_g1 c168117\_g1 c162048\_g1 | egu:105033850 egu:105035937 egu:105046802 egu:105046802 | 105033850 105035937 105046802 105046802 |  |
| Glycolysis / Gluconeogenesis | 11 | 154 | 0.127834246759 | 0.765520878647 | c175256\_g1 c172556\_g1 c156756\_g1 c164787\_g1 c148031\_g1 c224887\_g1 c106411\_g1 c188298\_g1 c151470\_g3 c151470\_g2 c19061\_g1 | egu:105052340 egu:105045855 egu:105042489 egu:105051810 egu:105042090 egu:105038179 egu:105057669 egu:105035292 egu:105046041 egu:105052340 egu:105051363 | 105052340 105045855 105042489 105051810 105042090 105038179 105057669 105035292 105046041 105052340 105051363 |  |
| Phenylalanine metabolism | 4 | 40 | 0.136311972996 | 0.765520878647 | c168304\_g3 c168304\_g2 c168304\_g1 c166080\_g1 | egu:105055673 egu:105055673 egu:105035781 egu:105055673 | 105055673 105055673 105035781 105055673 |  |
| beta-Alanine metabolism | 4 | 41 | 0.144611958017 | 0.765520878647 | c148031\_g1 c149905\_g2 c158241\_g1 c164787\_g1 | egu:105042090 egu:105053889 egu:105035498 egu:105051810 | 105042090 105053889 105035498 105051810 |  |
| Tryptophan metabolism | 4 | 42 | 0.153104175729 | 0.765520878647 | c148031\_g1 c168772\_g1 c154026\_g1 c164787\_g1 | egu:105042090 egu:105037452 egu:105056313 egu:105051810 | 105042090 105037452 105056313 105051810 |  |
| Regulation of autophagy | 4 | 43 | 0.161779574381 | 0.766324299702 | c155868\_g1 c163127\_g1 c173414\_g1 c126406\_g1 | egu:105060190 egu:105047382 egu:105060190 egu:105048117 | 105060190 105047382 105060190 105048117 |  |
| Diterpenoid biosynthesis | 3 | 29 | 0.174446675367 | 0.785010039152 | c166295\_g1 c162153\_g1 c155589\_g1 | egu:105047400 egu:105057308 egu:105032920 | 105047400 105057308 105032920 |  |
| Glycine, serine and threonine metabolism | 6 | 79 | 0.186090479848 | 0.797530627919 | c175256\_g1 c148031\_g1 c134944\_g1 c104726\_g1 c151470\_g3 c151470\_g2 | egu:105052340 egu:105042090 egu:105049537 egu:105046819 egu:105046041 egu:105052340 | 105052340 105042090 105049537 105046819 105046041 105052340 |  |
| Fatty acid degradation | 4 | 48 | 0.207577730639 | 0.822502200234 | c148031\_g1 c164787\_g1 c154026\_g1 c156705\_g2 | egu:105042090 egu:105051810 egu:105056313 egu:105059131 | 105042090 105051810 105056313 105059131 |  |
| Histidine metabolism | 2 | 17 | 0.211205127348 | 0.822502200234 | c148031\_g1 c164787\_g1 | egu:105042090 egu:105051810 | 105042090 105051810 |  |
| Plant hormone signal transduction | 17 | 288 | 0.219333920062 | 0.822502200234 | c149381\_g1 c164663\_g1 c199038\_g1 c167964\_g1 c159509\_g4 c170704\_g1 c171320\_g6 c147612\_g1 c87233\_g1 c164987\_g1 c114134\_g1 c173231\_g1 c96913\_g1 c169341\_g1 c157302\_g2 c157031\_g1 c165097\_g1 | egu:105035319 egu:105052902 egu:105052902 egu:105036306 egu:105043050 egu:105043422 egu:105034824 egu:105043791 egu:105052243 egu:105056702 egu:105058413 egu:105059593 egu:105056896 egu:105057423 egu:105042113 egu:105034824 egu:105038245 | 105035319 105052902 105052902 105036306 105043050 105043422 105034824 105043791 105052243 105056702 105058413 105059593 105056896 105057423 105042113 105034824 105038245 |  |
| Pyruvate metabolism | 7 | 104 | 0.236379697384 | 0.850047579575 | c156756\_g1 c154026\_g1 c164787\_g1 c148031\_g1 c164923\_g2 c224887\_g1 c188298\_g1 | egu:105042489 egu:105056313 egu:105051810 egu:105042090 egu:12079399 egu:105038179 egu:105035292 | 105042489 105056313 105051810 105042090 12079399 105038179 105035292 |  |
| Propanoate metabolism | 3 | 35 | 0.245569300766 | 0.850047579575 | c164923\_g2 c154026\_g1 c149905\_g2 | egu:12079399 egu:105056313 egu:105053889 | 12079399 105056313 105053889 |  |
| Sesquiterpenoid and triterpenoid biosynthesis | 1 | 6 | 0.276684194106 | 0.92228064702 | c121798\_g1 | egu:105034997 | 105034997 |  |
| Glycerophospholipid metabolism | 7 | 112 | 0.292389512107 | 0.926179174137 | c171054\_g2 c165623\_g2 c123356\_g1 c166846\_g1 c165891\_g1 c152491\_g2 c138851\_g1 | egu:105050243 egu:105033970 egu:105057319 egu:105038590 egu:105052345 egu:105061179 egu:105057319 | 105050243 105033970 105057319 105038590 105052345 105061179 105057319 |  |
| Arginine and proline metabolism | 4 | 58 | 0.307495905057 | 0.926179174137 | c158241\_g1 c148031\_g1 c164787\_g1 c157509\_g1 | egu:105035498 egu:105042090 egu:105051810 egu:105050758 | 105035498 105042090 105051810 105050758 |  |
| Glycerolipid metabolism | 5 | 77 | 0.312556255626 | 0.926179174137 | c148031\_g1 c166846\_g1 c123356\_g1 c138851\_g1 c164787\_g1 | egu:105042090 egu:105038590 egu:105057319 egu:105057319 egu:105051810 | 105042090 105038590 105057319 105057319 105051810 |  |
| Metabolic pathways | 107 | 2161 | 0.325666672964 | 0.926179174137 | c144134\_g1 c106411\_g1 c132414\_g1 c159060\_g1 c168403\_g1 c173509\_g1 c157321\_g1 c162165\_g1 c104726\_g1 c166080\_g1 c158038\_g1 c237479\_g1 c166229\_g1 c149545\_g1 c171764\_g2 c166846\_g1 c164923\_g2 c164916\_g2 c174721\_g1 c79219\_g1 c19061\_g1 c134944\_g1 c156756\_g1 c164813\_g2 c160525\_g1 c174640\_g1 c170429\_g1 c138851\_g1 c163448\_g1 c134078\_g1 c113669\_g1 c164787\_g1 c173363\_g9 c168117\_g1 c125968\_g1 c165193\_g1 c144654\_g1 c160444\_g2 c154320\_g1 c168772\_g1 c165623\_g2 c153668\_g2 c173969\_g2 c174249\_g4 c158521\_g1 c152491\_g2 c152671\_g2 c164585\_g12 c167554\_g2 c131089\_g1 c148031\_g1 c172129\_g1 c166724\_g1 c165891\_g1 c121798\_g1 c175256\_g1 c168304\_g3 c168304\_g2 c168304\_g1 c170715\_g1 c173762\_g1 c134112\_g1 c121911\_g1 c162258\_g1 c164898\_g1 c149905\_g2 c157509\_g1 c152671\_g1 c152936\_g1 c163736\_g1 c170749\_g3 c171769\_g1 c111106\_g1 c146039\_g2 c153668\_g1 c172556\_g1 c123356\_g1 c162048\_g1 c172816\_g3 c172816\_g4 c173723\_g1 c169815\_g1 c199368\_g1 c171054\_g2 c224887\_g1 c169051\_g3 c171631\_g6 c157926\_g1 c173495\_g2 c174203\_g1 c156705\_g2 c161872\_g2 c151470\_g3 c151470\_g2 c187575\_g1 c173081\_g3 c188298\_g1 c166365\_g1 c161991\_g2 c161991\_g1 c154969\_g2 c154026\_g1 c168307\_g1 c170890\_g1 c168535\_g1 c166605\_g1 c122873\_g1 | egu:105041628 egu:105057669 egu:105039562 egu:105043204 egu:105036591 egu:105035937 egu:105040157 egu:105056718 egu:105046819 egu:105055673 egu:105059341 egu:105047536 egu:105047342 egu:12079396 egu:105059690 egu:105038590 egu:12079399 egu:105054659 egu:105043800 egu:105043204 egu:105051363 egu:105049537 egu:105042489 egu:12079407 egu:105056077 egu:105043536 egu:105039157 egu:105057319 egu:105055201 egu:105051251 egu:105047535 egu:105051810 egu:12079461 egu:105046802 egu:105059690 egu:12079459 egu:105041005 egu:12079491 egu:105059691 egu:105037452 egu:105033970 egu:105050762 egu:12079460 egu:12079446 egu:105061001 egu:105061179 egu:105056861 egu:12079473 egu:105055983 egu:105041005 egu:105042090 egu:105048962 egu:105036407 egu:105052345 egu:105034997 egu:105052340 egu:105055673 egu:105055673 egu:105035781 egu:105041807 egu:105053112 egu:105040155 egu:12079457 egu:105040082 egu:105049059 egu:105053889 egu:105050758 egu:105056861 egu:105036609 egu:105042572 egu:105045199 egu:12079391 egu:105043800 egu:105055920 egu:105050762 egu:105045855 egu:105057319 egu:105046802 egu:105060320 egu:105060220 egu:105038559 egu:105033943 egu:12079383 egu:105050243 egu:105038179 egu:12079443 egu:105034397 egu:105053112 egu:105060892 egu:105036866 egu:105059131 egu:12079488 egu:105046041 egu:105052340 egu:105057579 egu:12079446 egu:105035292 egu:105040597 egu:105047535 egu:105047536 egu:105044935 egu:105056313 egu:105038499 egu:105034922 egu:105041389 egu:12079476 egu:105055983 | 105041628 105057669 105039562 105043204 105036591 105035937 105040157 105056718 105046819 105055673 105059341 105047536 105047342 12079396 105059690 105038590 12079399 105054659 105043800 105043204 105051363 105049537 105042489 12079407 105056077 105043536 105039157 105057319 105055201 105051251 105047535 105051810 12079461 105046802 105059690 12079459 105041005 12079491 105059691 105037452 105033970 105050762 12079460 12079446 105061001 105061179 105056861 12079473 105055983 105041005 105042090 105048962 105036407 105052345 105034997 105052340 105055673 105055673 105035781 105041807 105053112 105040155 12079457 105040082 105049059 105053889 105050758 105056861 105036609 105042572 105045199 12079391 105043800 105055920 105050762 105045855 105057319 105046802 105060320 105060220 105038559 105033943 12079383 105050243 105038179 12079443 105034397 105053112 105060892 105036866 105059131 12079488 105046041 105052340 105057579 12079446 105035292 105040597 105047535 105047536 105044935 105056313 105038499 105034922 105041389 12079476 105055983 |  |
| Endocytosis | 11 | 197 | 0.340330451625 | 0.926179174137 | c163181\_g5 c173338\_g6 c165623\_g2 c171004\_g1 c163801\_g1 c142610\_g1 c142610\_g2 c158968\_g2 c158968\_g1 c161639\_g1 c173778\_g2 | egu:105045690 egu:105058367 egu:105033970 egu:105038113 egu:105041064 egu:105044044 egu:105044044 egu:105047696 egu:105047696 egu:105045676 egu:105046398 | 105045690 105058367 105033970 105038113 105041064 105044044 105044044 105047696 105047696 105045676 105046398 |  |
| Galactose metabolism | 4 | 62 | 0.34895424957 | 0.926179174137 | c171613\_g1 c167034\_g1 c163736\_g1 c106411\_g1 | egu:105034931 egu:105057305 egu:105042572 egu:105057669 | 105034931 105057305 105042572 105057669 |  |
| Photosynthesis | 5 | 82 | 0.357668815805 | 0.926179174137 | c164813\_g2 c171769\_g1 c121911\_g1 c199368\_g1 c166605\_g1 | egu:12079407 egu:12079391 egu:12079457 egu:12079383 egu:12079476 | 12079407 12079391 12079457 12079383 12079476 |  |
| Purine metabolism | 10 | 181 | 0.363010004919 | 0.926179174137 | c168403\_g1 c171631\_g6 c156756\_g1 c173363\_g9 c224887\_g1 c165193\_g1 c173969\_g2 c188298\_g1 c161748\_g2 c166229\_g1 | egu:105036591 egu:105034397 egu:105042489 egu:12079461 egu:105038179 egu:12079459 egu:12079460 egu:105035292 egu:105057256 egu:105047342 | 105036591 105034397 105042489 12079461 105038179 12079459 12079460 105035292 105057256 105047342 |  |
| Synthesis and degradation of ketone bodies | 1 | 9 | 0.370471669655 | 0.926179174137 | c154026\_g1 | egu:105056313 | 105056313 |  |
| Non-homologous end-joining | 1 | 10 | 0.398953926647 | 0.954449062581 | c160026\_g38 | egu:105049145 | 105049145 |  |
| Pentose and glucuronate interconversions | 5 | 87 | 0.402989604201 | 0.954449062581 | c158038\_g1 c164787\_g1 c122873\_g1 c167554\_g2 c174203\_g1 | egu:105059341 egu:105051810 egu:105055983 egu:105055983 egu:105036866 | 105059341 105051810 105055983 105055983 105036866 |  |
| Fatty acid elongation | 2 | 30 | 0.427847794353 | 0.987341063891 | c163278\_g1 c151794\_g1 | egu:105042699 egu:105040213 | 105042699 105040213 |  |
| Vitamin B6 metabolism | 1 | 12 | 0.452117033843 | 0.999900910513 | c104726\_g1 | egu:105046819 | 105046819 |  |
| Homologous recombination | 3 | 52 | 0.455840742802 | 0.999900910513 | c160026\_g38 c172876\_g4 c146039\_g1 | egu:105049145 egu:105042242 egu:105045532 | 105049145 105042242 105045532 |  |
| Biosynthesis of amino acids | 14 | 290 | 0.510896339093 | 0.999900910513 | c175256\_g1 c134112\_g1 c156756\_g1 c148031\_g1 c162258\_g1 c134944\_g1 c224887\_g1 c154969\_g2 c104726\_g1 c188298\_g1 c151470\_g3 c151470\_g2 c19061\_g1 c152936\_g1 | egu:105052340 egu:105040155 egu:105042489 egu:105042090 egu:105040082 egu:105049537 egu:105038179 egu:105044935 egu:105046819 egu:105035292 egu:105046041 egu:105052340 egu:105051363 egu:105036609 | 105052340 105040155 105042489 105042090 105040082 105049537 105038179 105044935 105046819 105035292 105046041 105052340 105051363 105036609 |  |
| Oxidative phosphorylation | 8 | 163 | 0.511959154834 | 0.999900910513 | c149545\_g1 c173081\_g3 c169815\_g1 c169051\_g3 c174249\_g4 c161872\_g2 c160444\_g2 c121911\_g1 | egu:12079396 egu:12079446 egu:105033943 egu:12079443 egu:12079446 egu:12079488 egu:12079491 egu:12079457 | 12079396 12079446 105033943 12079443 12079446 12079488 12079491 12079457 |  |
| Lysine biosynthesis | 1 | 15 | 0.523189835079 | 0.999900910513 | c148031\_g1 | egu:105042090 | 105042090 |  |
| Phenylpropanoid biosynthesis | 8 | 168 | 0.544998706738 | 0.999900910513 | c157321\_g1 c168304\_g3 c168304\_g2 c168304\_g1 c170749\_g3 c166080\_g1 c158521\_g1 c166365\_g1 | egu:105040157 egu:105055673 egu:105055673 egu:105035781 egu:105045199 egu:105055673 egu:105061001 egu:105040597 | 105040157 105055673 105055673 105035781 105045199 105055673 105061001 105040597 |  |
| Glycosaminoglycan degradation | 1 | 18 | 0.585057047159 | 0.999900910513 | c187575\_g1 | egu:105057579 | 105057579 |  |
| Pyrimidine metabolism | 6 | 134 | 0.611398881239 | 0.999900910513 | c171631\_g6 c173363\_g9 c165193\_g1 c173969\_g2 c161748\_g2 c166229\_g1 | egu:105034397 egu:12079461 egu:12079459 egu:12079460 egu:105057256 egu:105047342 | 105034397 12079461 12079459 12079460 105057256 105047342 |  |
| Fructose and mannose metabolism | 4 | 90 | 0.619832566605 | 0.999900910513 | c164916\_g2 c158038\_g1 c106411\_g1 c170553\_g1 | egu:105054659 egu:105059341 egu:105057669 egu:105046004 | 105054659 105059341 105057669 105046004 |  |
| Cysteine and methionine metabolism | 5 | 114 | 0.629627074173 | 0.999900910513 | c157509\_g1 c174640\_g1 c170890\_g1 c162258\_g1 c154969\_g2 | egu:105050758 egu:105043536 egu:105034922 egu:105040082 egu:105044935 | 105050758 105043536 105034922 105040082 105044935 |  |
| Biosynthesis of secondary metabolites | 54 | 1184 | 0.633086541738 | 0.999900910513 | c175256\_g1 c134112\_g1 c168304\_g3 c168304\_g2 c168304\_g1 c170715\_g1 c224887\_g1 c162048\_g1 c173762\_g1 c106411\_g1 c156756\_g1 c160525\_g1 c174640\_g1 c162165\_g1 c138851\_g1 c163448\_g1 c166295\_g1 c168403\_g1 c173509\_g1 c157321\_g1 c157926\_g1 c164787\_g1 c162153\_g1 c168117\_g1 c154969\_g2 c104726\_g1 c156705\_g2 c144654\_g1 c166080\_g1 c151470\_g3 c151470\_g2 c153721\_g1 c152936\_g1 c164923\_g2 c165623\_g2 c151794\_g1 c170749\_g3 c131089\_g1 c146039\_g2 c188298\_g1 c158521\_g1 c166365\_g1 c19061\_g1 c173723\_g1 c172556\_g1 c123356\_g1 c154026\_g1 c168307\_g1 c148031\_g1 c166846\_g1 c172129\_g1 c121798\_g1 c163278\_g1 c162258\_g1 | egu:105052340 egu:105040155 egu:105055673 egu:105055673 egu:105035781 egu:105041807 egu:105038179 egu:105046802 egu:105053112 egu:105057669 egu:105042489 egu:105056077 egu:105043536 egu:105056718 egu:105057319 egu:105055201 egu:105047400 egu:105036591 egu:105035937 egu:105040157 egu:105053112 egu:105051810 egu:105057308 egu:105046802 egu:105044935 egu:105046819 egu:105059131 egu:105041005 egu:105055673 egu:105046041 egu:105052340 egu:105043024 egu:105036609 egu:12079399 egu:105033970 egu:105040213 egu:105045199 egu:105041005 egu:105055920 egu:105035292 egu:105061001 egu:105040597 egu:105051363 egu:105038559 egu:105045855 egu:105057319 egu:105056313 egu:105038499 egu:105042090 egu:105038590 egu:105048962 egu:105034997 egu:105042699 egu:105040082 | 105052340 105040155 105055673 105055673 105035781 105041807 105038179 105046802 105053112 105057669 105042489 105056077 105043536 105056718 105057319 105055201 105047400 105036591 105035937 105040157 105053112 105051810 105057308 105046802 105044935 105046819 105059131 105041005 105055673 105046041 105052340 105043024 105036609 12079399 105033970 105040213 105045199 105041005 105055920 105035292 105061001 105040597 105051363 105038559 105045855 105057319 105056313 105038499 105042090 105038590 105048962 105034997 105042699 105040082 |  |
| Other glycan degradation | 1 | 21 | 0.638909174272 | 0.999900910513 | c166724\_g1 | egu:105036407 | 105036407 |  |
| Isoquinoline alkaloid biosynthesis | 1 | 21 | 0.638909174272 | 0.999900910513 | c172129\_g1 | egu:105048962 | 105048962 |  |
| Zeatin biosynthesis | 1 | 22 | 0.65526198331 | 0.999900910513 | c166677\_g1 | egu:105043135 | 105043135 |  |
| Butanoate metabolism | 1 | 22 | 0.65526198331 | 0.999900910513 | c154026\_g1 | egu:105056313 | 105056313 |  |
| Carbon metabolism | 14 | 322 | 0.662205558212 | 0.999900910513 | c175256\_g1 c156756\_g1 c154026\_g1 c134944\_g1 c164923\_g2 c224887\_g1 c149905\_g2 c106411\_g1 c188298\_g1 c160525\_g1 c151470\_g3 c151470\_g2 c19061\_g1 c164585\_g12 | egu:105052340 egu:105042489 egu:105056313 egu:105049537 egu:12079399 egu:105038179 egu:105053889 egu:105057669 egu:105035292 egu:105056077 egu:105046041 egu:105052340 egu:105051363 egu:12079473 | 105052340 105042489 105056313 105049537 12079399 105038179 105053889 105057669 105035292 105056077 105046041 105052340 105051363 12079473 |  |
| Glycosylphosphatidylinositol(GPI)-anchor biosynthesis | 1 | 23 | 0.670875467942 | 0.999900910513 | c170429\_g1 | egu:105039157 | 105039157 |  |
| SNARE interactions in vesicular transport | 2 | 52 | 0.708141516817 | 0.999900910513 | c162457\_g1 c167775\_g2 | egu:105059832 egu:105059832 | 105059832 105059832 |  |
| Flavonoid biosynthesis | 2 | 54 | 0.726920634034 | 0.999900910513 | c144654\_g1 c131089\_g1 | egu:105041005 egu:105041005 | 105041005 105041005 |  |
| Cutin, suberine and wax biosynthesis | 1 | 28 | 0.73896971505 | 0.999900910513 | c160204\_g2 | egu:105046261 | 105046261 |  |
| Phenylalanine, tyrosine and tryptophan biosynthesis | 2 | 56 | 0.744667000651 | 0.999900910513 | c134112\_g1 c152936\_g1 | egu:105040155 egu:105036609 | 105040155 105036609 |  |
| Fatty acid metabolism | 3 | 85 | 0.766469407235 | 0.999900910513 | c156705\_g2 c164923\_g2 c154026\_g1 | egu:105059131 egu:12079399 egu:105056313 | 105059131 12079399 105056313 |  |
| DNA replication | 2 | 59 | 0.769428347617 | 0.999900910513 | c31927\_g1 c146039\_g1 | egu:105060234 egu:105045532 | 105060234 105045532 |  |
| Ether lipid metabolism | 1 | 32 | 0.783166833716 | 0.999900910513 | c165623\_g2 | egu:105033970 | 105033970 |  |
| Biosynthesis of unsaturated fatty acids | 1 | 36 | 0.819891542489 | 0.999900910513 | c156705\_g2 | egu:105059131 | 105059131 |  |
| Nitrogen metabolism | 1 | 38 | 0.835854722692 | 0.999900910513 | c140928\_g1 | egu:105048068 | 105048068 |  |
| Tyrosine metabolism | 1 | 39 | 0.843298535444 | 0.999900910513 | c172129\_g1 | egu:105048962 | 105048962 |  |
| Sphingolipid metabolism | 1 | 39 | 0.843298535444 | 0.999900910513 | c166724\_g1 | egu:105036407 | 105036407 |  |
| Inositol phosphate metabolism | 2 | 72 | 0.853966360395 | 0.999900910513 | c152208\_g2 c149905\_g2 | egu:105056668 egu:105053889 | 105056668 105053889 |  |
| Cyanoamino acid metabolism | 1 | 43 | 0.869852696051 | 0.999900910513 | c170749\_g3 | egu:105045199 | 105045199 |  |
| Base excision repair | 1 | 44 | 0.875757102434 | 0.999900910513 | c160071\_g1 | egu:105052529 | 105052529 |  |
| Mismatch repair | 1 | 48 | 0.896818792521 | 0.999900910513 | c146039\_g1 | egu:105045532 | 105045532 |  |
| Glyoxylate and dicarboxylate metabolism | 2 | 83 | 0.902293469328 | 0.999900910513 | c154026\_g1 c164585\_g12 | egu:105056313 egu:12079473 | 105056313 12079473 |  |
| Porphyrin and chlorophyll metabolism | 1 | 50 | 0.90597243464 | 0.999900910513 | c153721\_g1 | egu:105043024 | 105043024 |  |
| RNA transport | 6 | 205 | 0.919990948656 | 0.999900910513 | c149538\_g1 c174078\_g4 c170817\_g1 c169579\_g1 c165981\_g1 c145725\_g1 | egu:105051987 egu:105052955 egu:105051424 egu:105035083 egu:105040546 egu:105043116 | 105051987 105052955 105051424 105035083 105040546 105043116 |  |
| Carbon fixation in photosynthetic organisms | 2 | 89 | 0.921908193227 | 0.999900910513 | c19061\_g1 c164585\_g12 | egu:105051363 egu:12079473 | 105051363 12079473 |  |
| Basal transcription factors | 1 | 54 | 0.921919158235 | 0.999900910513 | c105620\_g1 | egu:105056929 | 105056929 |  |
| N-Glycan biosynthesis | 1 | 55 | 0.925464585947 | 0.999900910513 | c134078\_g1 | egu:105051251 | 105051251 |  |
| Alanine, aspartate and glutamate metabolism | 1 | 57 | 0.93208056583 | 0.999900910513 | c168403\_g1 | egu:105036591 | 105036591 |  |
| Fatty acid biosynthesis | 1 | 59 | 0.93811023721 | 0.999900910513 | c164923\_g2 | egu:12079399 | 12079399 |  |
| Terpenoid backbone biosynthesis | 1 | 61 | 0.943605475 | 0.999900910513 | c154026\_g1 | egu:105056313 | 105056313 |  |
| Spliceosome | 6 | 227 | 0.955601966007 | 0.999900910513 | c173699\_g2 c160522\_g1 c163181\_g5 c161316\_g2 c172537\_g3 c172537\_g1 | egu:105050579 egu:105040405 egu:105045690 egu:105061246 egu:105041295 egu:105048742 | 105050579 105040405 105045690 105061246 105041295 105048742 |  |
| Nucleotide excision repair | 1 | 70 | 0.96289466558 | 0.999900910513 | c146039\_g1 | egu:105045532 | 105045532 |  |
| Pentose phosphate pathway | 1 | 80 | 0.976704188762 | 0.999900910513 | c160525\_g1 | egu:105056077 | 105056077 |  |
| RNA degradation | 2 | 130 | 0.984218838963 | 0.999900910513 | c167403\_g1 c156792\_g1 | egu:105049057 egu:105033956 | 105049057 105033956 |  |
| mRNA surveillance pathway | 2 | 149 | 0.992697986048 | 0.999900910513 | c167848\_g1 c165163\_g1 | egu:105034445 egu:105051857 | 105034445 105051857 |  |
| Protein processing in endoplasmic reticulum | 5 | 257 | 0.992979878964 | 0.999900910513 | c163181\_g5 c159312\_g1 c163791\_g1 c42777\_g1 c141977\_g1 | egu:105045690 egu:105053689 egu:105053788 egu:105053227 egu:105042707 | 105045690 105053689 105053788 105053227 105042707 |  |
| Peroxisome | 1 | 113 | 0.994999971608 | 0.999900910513 | c156705\_g2 | egu:105059131 | 105059131 |  |
| Ubiquitin mediated proteolysis | 2 | 159 | 0.995160127001 | 0.999900910513 | c169528\_g2 c153783\_g1 | egu:105043171 egu:105054824 | 105043171 105054824 |  |
| Phagosome | 1 | 118 | 0.996041274766 | 0.999900910513 | c152208\_g2 | egu:105056668 | 105056668 |  |
| Ribosome | 5 | 377 | 0.999900910513 | 0.999900910513 | c166320\_g6 c131320\_g1 c87612\_g1 c133240\_g1 c167542\_g1 | egu:105055675 egu:12079413 egu:12079506 egu:105037979 egu:12079507 | 105055675 12079413 12079506 105037979 12079507 |  |
