# Supplementary material for: Key anti-freeze genes and pathways of Lanzhou lily (Lilium davidii, var. unicolor) during the seedling stage
Source: PLoS One. 2024 Mar 21;19(3):e0299259. doi: 10.1371/journal.pone.0299259 (PMC10956819; doi:10.1371/journal.pone.0299259)
Supplement: S1 File — (ZIP) [file pone.0299259.s004.zip › S1 Zip/src/egu00010.html]

egu00010


- egu:105051810

- Up regulated genes

c164787\_g1(0.64456)
- egu:105042090

- Up regulated genes

c148031\_g1(0.68114)

- egu:105045855

- Up regulated genes

c172556\_g1(1.6166)

- egu:105045855

- Up regulated genes

c172556\_g1(1.6166)

- egu:105035292

- Up regulated genes

c188298\_g1(2.5083)
- egu:105038179

- Up regulated genes

c224887\_g1(2.5486)
- egu:105042489

- Up regulated genes

c156756\_g1(1.0437)

- egu:105051363

- Up regulated genes

c19061\_g1(3.5186)

- egu:105057669

- Up regulated genes

c106411\_g1(0.71601)

- egu:105057669

- Up regulated genes

c106411\_g1(0.71601)

- egu:105052340

- Up regulated genes

c175256\_g1(1.5585) c151470\_g2(1.6287)
- egu:105046041

- Up regulated genes

c151470\_g3(2.2497)

Close
