# Supplementary material for: Key anti-freeze genes and pathways of Lanzhou lily (Lilium davidii, var. unicolor) during the seedling stage
Source: PLoS One. 2024 Mar 21;19(3):e0299259. doi: 10.1371/journal.pone.0299259 (PMC10956819; doi:10.1371/journal.pone.0299259)
Supplement: S1 File — (ZIP) [file pone.0299259.s004.zip › S1 Zip/src/egu00020.html]

egu00020


- egu:105059758

- Down regulated genes

c169453\_g2(-1.0615)
- egu:105045006

- Down regulated genes

c171099\_g1(-0.60519)

- egu:105059287

- Down regulated genes

c163496\_g1(-0.6363)
- egu:105034969

- Down regulated genes

c113371\_g2(-0.73611)
- egu:105034723

- Down regulated genes

c169294\_g2(-0.85273)

- egu:105059287

- Down regulated genes

c163496\_g1(-0.6363)
- egu:105034969

- Down regulated genes

c113371\_g2(-0.73611)
- egu:105034723

- Down regulated genes

c169294\_g2(-0.85273)

- egu:105054530

- Down regulated genes

c174574\_g3(-3.7395) c104889\_g2(-1.5635)
- egu:105034557

- Down regulated genes

c104889\_g1(-1.7144)

Close
