# Supplementary material for: Key anti-freeze genes and pathways of Lanzhou lily (Lilium davidii, var. unicolor) during the seedling stage
Source: PLoS One. 2024 Mar 21;19(3):e0299259. doi: 10.1371/journal.pone.0299259 (PMC10956819; doi:10.1371/journal.pone.0299259)
Supplement: S1 File — (ZIP) [file pone.0299259.s004.zip › S1 Zip/src/egu00040.html]

egu00040


- egu:105055983

- Up regulated genes

c122873\_g1(1.3716) c167554\_g2(2.5763)

- egu:105036866

- Up regulated genes

c174203\_g1(4.065)

- egu:105059341

- Up regulated genes

c158038\_g1(0.66748)

Close
