# Supplementary material for: Key anti-freeze genes and pathways of Lanzhou lily (Lilium davidii, var. unicolor) during the seedling stage
Source: PLoS One. 2024 Mar 21;19(3):e0299259. doi: 10.1371/journal.pone.0299259 (PMC10956819; doi:10.1371/journal.pone.0299259)
Supplement: S1 File — (ZIP) [file pone.0299259.s004.zip › S1 Zip/src/egu00051.html]

egu00051


- egu:105059341

- Up regulated genes

c158038\_g1(0.66748)

- egu:105046004

- Up regulated genes

c170553\_g1(6.0627)

- egu:105057669

- Up regulated genes

c106411\_g1(0.71601)

- egu:105057669

- Up regulated genes

c106411\_g1(0.71601)

- egu:105054659

- Up regulated genes

c164916\_g2(1.1452)

Close
