# Supplementary material for: Key anti-freeze genes and pathways of Lanzhou lily (Lilium davidii, var. unicolor) during the seedling stage
Source: PLoS One. 2024 Mar 21;19(3):e0299259. doi: 10.1371/journal.pone.0299259 (PMC10956819; doi:10.1371/journal.pone.0299259)
Supplement: S1 File — (ZIP) [file pone.0299259.s004.zip › S1 Zip/src/egu00052.html]

egu00052


- egu:105057669

- Up regulated genes

c106411\_g1(0.71601)

- egu:105042572

- Up regulated genes

c163736\_g1(1.0454)

- egu:105034931

- Up regulated genes

c171613\_g1(2.4003)

- egu:105057305

- Up regulated genes

c167034\_g1(4.4592)

Close
