# Supplementary material for: Key anti-freeze genes and pathways of Lanzhou lily (Lilium davidii, var. unicolor) during the seedling stage
Source: PLoS One. 2024 Mar 21;19(3):e0299259. doi: 10.1371/journal.pone.0299259 (PMC10956819; doi:10.1371/journal.pone.0299259)
Supplement: S1 File — (ZIP) [file pone.0299259.s004.zip › S1 Zip/src/egu00053.html]

egu00053


- egu:105038499

- Up regulated genes

c168307\_g1(1.4006)

- egu:105055983

- Up regulated genes

c167554\_g2(2.5763) c122873\_g1(1.3716)

- egu:105055920

- Up regulated genes

c146039\_g2(Inf)

- egu:105051810

- Up regulated genes

c164787\_g1(0.64456)
- egu:105042090

- Up regulated genes

c148031\_g1(0.68114)

- egu:105045793

- Up regulated genes

c224426\_g1(3.6499)

Close
