# Supplementary material for: Key anti-freeze genes and pathways of Lanzhou lily (Lilium davidii, var. unicolor) during the seedling stage
Source: PLoS One. 2024 Mar 21;19(3):e0299259. doi: 10.1371/journal.pone.0299259 (PMC10956819; doi:10.1371/journal.pone.0299259)
Supplement: S1 File — (ZIP) [file pone.0299259.s004.zip › S1 Zip/src/egu00071.html]

egu00071


- egu:105056313

- Up regulated genes

c154026\_g1(0.58616)

- egu:105059131

- Up regulated genes

c156705\_g2(0.75345)

- egu:105059131

- Up regulated genes

c156705\_g2(0.75345)

- egu:105059131

- Up regulated genes

c156705\_g2(0.75345)

- egu:105059131

- Up regulated genes

c156705\_g2(0.75345)

- egu:105059131

- Up regulated genes

c156705\_g2(0.75345)

- egu:105059131

- Up regulated genes

c156705\_g2(0.75345)

- egu:105056313

- Up regulated genes

c154026\_g1(0.58616)

- egu:105051810

- Up regulated genes

c164787\_g1(0.64456)
- egu:105042090

- Up regulated genes

c148031\_g1(0.68114)

Close
