# Supplementary material for: Key anti-freeze genes and pathways of Lanzhou lily (Lilium davidii, var. unicolor) during the seedling stage
Source: PLoS One. 2024 Mar 21;19(3):e0299259. doi: 10.1371/journal.pone.0299259 (PMC10956819; doi:10.1371/journal.pone.0299259)
Supplement: S1 File — (ZIP) [file pone.0299259.s004.zip › S1 Zip/src/egu00100.html]

egu00100


- egu:105058937

- Down regulated genes

c134136\_g1(-0.64172)

- egu:105058937

- Down regulated genes

c134136\_g1(-0.64172)

- egu:105058937

- Down regulated genes

c134136\_g1(-0.64172)

- egu:105058937

- Down regulated genes

c134136\_g1(-0.64172)

- egu:105058937

- Down regulated genes

c134136\_g1(-0.64172)

- egu:105058937

- Down regulated genes

c134136\_g1(-0.64172)

- egu:105049267

- Down regulated genes

c173904\_g1(-0.61455)

- egu:105058937

- Down regulated genes

c134136\_g1(-0.64172)

- egu:105058937

- Down regulated genes

c134136\_g1(-0.64172)

- egu:105055883

- Down regulated genes

c152294\_g1(-1.1731)

- egu:105058937

- Down regulated genes

c134136\_g1(-0.64172)

Close
