# Supplementary material for: Key anti-freeze genes and pathways of Lanzhou lily (Lilium davidii, var. unicolor) during the seedling stage
Source: PLoS One. 2024 Mar 21;19(3):e0299259. doi: 10.1371/journal.pone.0299259 (PMC10956819; doi:10.1371/journal.pone.0299259)
Supplement: S1 File — (ZIP) [file pone.0299259.s004.zip › S1 Zip/src/egu00130.html]

egu00130


- egu:105061098

- Down regulated genes

c164952\_g6(-0.86221)

- egu:105047162

- Down regulated genes

c154629\_g1(-0.79402)

- egu:105060927

- Down regulated genes

c173971\_g3(-0.86974)

- egu:105061098

- Down regulated genes

c164952\_g6(-0.86221)

- egu:105061098

- Down regulated genes

c164952\_g6(-0.86221)

- egu:105061098

- Down regulated genes

c164952\_g6(-0.86221)

- egu:105040940

- Down regulated genes

c163118\_g1(-1.775)

- egu:105041933

- Down regulated genes

c165685\_g1(-0.89504)

- egu:105041933

- Down regulated genes

c165685\_g1(-0.89504)

- egu:105046456

- Down regulated genes

c159709\_g1(-0.99518)

Close
