# Supplementary material for: Key anti-freeze genes and pathways of Lanzhou lily (Lilium davidii, var. unicolor) during the seedling stage
Source: PLoS One. 2024 Mar 21;19(3):e0299259. doi: 10.1371/journal.pone.0299259 (PMC10956819; doi:10.1371/journal.pone.0299259)
Supplement: S1 File — (ZIP) [file pone.0299259.s004.zip › S1 Zip/src/egu00190.html]

egu00190


- egu:12079488

- Up regulated genes

c161872\_g2(4.6565)

- egu:12079443

- Up regulated genes

c169051\_g3(2.8915)

- egu:12079446

- Up regulated genes

c173081\_g3(2.799) c174249\_g4(2.8336)

- egu:12079491

- Up regulated genes

c160444\_g2(4.5104)

- egu:12079396

- Up regulated genes

c149545\_g1(6.1196)

- egu:12079491

- Up regulated genes

c160444\_g2(4.5104)
- egu:12079488

- Up regulated genes

c161872\_g2(4.6565)
- egu:12079396

- Up regulated genes

c149545\_g1(6.1196)
- egu:12079443

- Up regulated genes

c169051\_g3(2.8915)
- egu:12079446

- Up regulated genes

c173081\_g3(2.799) c174249\_g4(2.8336)

- egu:105033943

- Up regulated genes

c169815\_g1(3.8894)

- egu:12079457

- Up regulated genes

c121911\_g1(2.3206)

- egu:105033943

- Up regulated genes

c169815\_g1(3.8894)

- egu:12079457

- Up regulated genes

c121911\_g1(2.3206)

Close
