# Supplementary material for: Key anti-freeze genes and pathways of Lanzhou lily (Lilium davidii, var. unicolor) during the seedling stage
Source: PLoS One. 2024 Mar 21;19(3):e0299259. doi: 10.1371/journal.pone.0299259 (PMC10956819; doi:10.1371/journal.pone.0299259)
Supplement: S1 File — (ZIP) [file pone.0299259.s004.zip › S1 Zip/src/egu00195.html]

egu00195


- egu:12079457

- Up regulated genes

c121911\_g1(2.3206)

- egu:12079407

- Up regulated genes

c164813\_g2(2.8226)

- egu:12079391

- Up regulated genes

c171769\_g1(4.2114)

- egu:12079383

- Up regulated genes

c199368\_g1(2.6746)

- egu:12079476

- Up regulated genes

c166605\_g1(5.4713)

- egu:12079457

- Up regulated genes

c121911\_g1(2.3206)

Close
