# Supplementary material for: Key anti-freeze genes and pathways of Lanzhou lily (Lilium davidii, var. unicolor) during the seedling stage
Source: PLoS One. 2024 Mar 21;19(3):e0299259. doi: 10.1371/journal.pone.0299259 (PMC10956819; doi:10.1371/journal.pone.0299259)
Supplement: S1 File — (ZIP) [file pone.0299259.s004.zip › S1 Zip/src/egu00196.html]

egu00196


- egu:105058558

- Down regulated genes

c149445\_g1(-1.8714)

- egu:105039517

- Down regulated genes

c152435\_g1(-0.73642)

- egu:105053065

- Down regulated genes

c139230\_g1(-1.5021)
- egu:105032432

- Down regulated genes

c211152\_g1(-0.77913)

- egu:105033408

- Down regulated genes

c149517\_g1(-1.2503)

- egu:105058393

- Down regulated genes

c157047\_g1(-1.0947)

- egu:105051572

- Down regulated genes

c174985\_g1(-1.0535)

- egu:105032321

- Down regulated genes

c155535\_g1(-2.2248)

- egu:105035084

- Down regulated genes

c164577\_g1(-0.89077)

- egu:105032174

- Down regulated genes

c115377\_g1(-4.4924)

- egu:105058244

- Down regulated genes

c153860\_g1(-0.83296)

Close
