# Supplementary material for: Key anti-freeze genes and pathways of Lanzhou lily (Lilium davidii, var. unicolor) during the seedling stage
Source: PLoS One. 2024 Mar 21;19(3):e0299259. doi: 10.1371/journal.pone.0299259 (PMC10956819; doi:10.1371/journal.pone.0299259)
Supplement: S1 File — (ZIP) [file pone.0299259.s004.zip › S1 Zip/src/egu00220.html]

egu00220


- egu:105057795

- Down regulated genes

c158088\_g1(-1.4881)
- egu:105035493

- Down regulated genes

c160412\_g1(-0.71149)

- egu:105043264

- Down regulated genes

c152607\_g1(-0.57758)

- egu:105060929

- Down regulated genes

c132251\_g1(-0.6859)
- egu:105052838

- Down regulated genes

c174151\_g1(-1.0054)

- egu:105058731

- Down regulated genes

c146725\_g1(-1.1138)

- egu:105048107

- Down regulated genes

c159323\_g1(-0.98431)

- egu:105049882

- Down regulated genes

c71483\_g1(-0.84471)

Close
