# Supplementary material for: Key anti-freeze genes and pathways of Lanzhou lily (Lilium davidii, var. unicolor) during the seedling stage
Source: PLoS One. 2024 Mar 21;19(3):e0299259. doi: 10.1371/journal.pone.0299259 (PMC10956819; doi:10.1371/journal.pone.0299259)
Supplement: S1 File — (ZIP) [file pone.0299259.s004.zip › S1 Zip/src/egu00240.html]

egu00240


- egu:12079460

- Up regulated genes

c173969\_g2(1.9211)
- egu:12079461

- Up regulated genes

c173363\_g9(4.3388)
- egu:105047342

- Up regulated genes

c166229\_g1(0.90041)
- egu:105034397

- Up regulated genes

c171631\_g6(0.82337)
- egu:12079459

- Up regulated genes

c165193\_g1(5.1039)

- egu:105057256

- Up regulated genes

c161748\_g2(0.85361)

- egu:105057256

- Up regulated genes

c161748\_g2(0.85361)

- egu:12079460

- Up regulated genes

c173969\_g2(1.9211)
- egu:12079461

- Up regulated genes

c173363\_g9(4.3388)
- egu:105047342

- Up regulated genes

c166229\_g1(0.90041)
- egu:105034397

- Up regulated genes

c171631\_g6(0.82337)
- egu:12079459

- Up regulated genes

c165193\_g1(5.1039)

- egu:105057256

- Up regulated genes

c161748\_g2(0.85361)

- egu:105057256

- Up regulated genes

c161748\_g2(0.85361)

- egu:105057256

- Up regulated genes

c161748\_g2(0.85361)

- egu:105057256

- Up regulated genes

c161748\_g2(0.85361)

Close
