# Supplementary material for: Key anti-freeze genes and pathways of Lanzhou lily (Lilium davidii, var. unicolor) during the seedling stage
Source: PLoS One. 2024 Mar 21;19(3):e0299259. doi: 10.1371/journal.pone.0299259 (PMC10956819; doi:10.1371/journal.pone.0299259)
Supplement: S1 File — (ZIP) [file pone.0299259.s004.zip › S1 Zip/src/egu00260.html]

egu00260


- egu:105042090

- Up regulated genes

c148031\_g1(0.68114)

- egu:105049537

- Up regulated genes

c134944\_g1(1.2712)

- egu:105046819

- Up regulated genes

c104726\_g1(1.0852)

- egu:105046041

- Up regulated genes

c151470\_g3(2.2497)
- egu:105052340

- Up regulated genes

c175256\_g1(1.5585) c151470\_g2(1.6287)

Close
