# Supplementary material for: Key anti-freeze genes and pathways of Lanzhou lily (Lilium davidii, var. unicolor) during the seedling stage
Source: PLoS One. 2024 Mar 21;19(3):e0299259. doi: 10.1371/journal.pone.0299259 (PMC10956819; doi:10.1371/journal.pone.0299259)
Supplement: S1 File — (ZIP) [file pone.0299259.s004.zip › S1 Zip/src/egu00270.html]

egu00270


- egu:105043536

- Up regulated genes

c174640\_g1(7.5505)

- egu:105050758

- Up regulated genes

c157509\_g1(0.97039)

- egu:105040082

- Up regulated genes

c162258\_g1(6.8824)
- egu:105044935

- Up regulated genes

c154969\_g2(2.2867)

- egu:105034922

- Up regulated genes

c170890\_g1(2.1422)

Close
