# Supplementary material for: Key anti-freeze genes and pathways of Lanzhou lily (Lilium davidii, var. unicolor) during the seedling stage
Source: PLoS One. 2024 Mar 21;19(3):e0299259. doi: 10.1371/journal.pone.0299259 (PMC10956819; doi:10.1371/journal.pone.0299259)
Supplement: S1 File — (ZIP) [file pone.0299259.s004.zip › S1 Zip/src/egu00310.html]

egu00310


- egu:105042090

- Up regulated genes

c148031\_g1(0.68114)

- egu:105056313

- Up regulated genes

c154026\_g1(0.58616)

- egu:105053701

- Up regulated genes

c159372\_g1(0.94146)
- egu:105043130

- Up regulated genes

c165534\_g2(2.36)

- egu:105053701

- Up regulated genes

c159372\_g1(0.94146)
- egu:105043130

- Up regulated genes

c165534\_g2(2.36)

- egu:105053701

- Up regulated genes

c159372\_g1(0.94146)
- egu:105043130

- Up regulated genes

c165534\_g2(2.36)

- egu:105051810

- Up regulated genes

c164787\_g1(0.64456)
- egu:105042090

- Up regulated genes

c148031\_g1(0.68114)

Close
