# Supplementary material for: Key anti-freeze genes and pathways of Lanzhou lily (Lilium davidii, var. unicolor) during the seedling stage
Source: PLoS One. 2024 Mar 21;19(3):e0299259. doi: 10.1371/journal.pone.0299259 (PMC10956819; doi:10.1371/journal.pone.0299259)
Supplement: S1 File — (ZIP) [file pone.0299259.s004.zip › S1 Zip/src/egu00480.html]

egu00480


- egu:105056588

- Up regulated genes

c160646\_g1(4.5132)
- egu:105037855

- Up regulated genes

c173732\_g2(1.4553) c165975\_g1(2.9981)
- egu:105058473

- Up regulated genes

c163752\_g1(2.2611)
- egu:105032151

- Up regulated genes

c158909\_g1(5.8846) c152293\_g1(2.6398)
- egu:105044685

- Up regulated genes

c145216\_g1(0.99193)
- egu:105043366

- Up regulated genes

c213021\_g1(3.7709)
- egu:105054171

- Up regulated genes

c162564\_g1(8.2457)

- egu:105045793

- Up regulated genes

c224426\_g1(3.6499)

Close
