# Supplementary material for: Key anti-freeze genes and pathways of Lanzhou lily (Lilium davidii, var. unicolor) during the seedling stage
Source: PLoS One. 2024 Mar 21;19(3):e0299259. doi: 10.1371/journal.pone.0299259 (PMC10956819; doi:10.1371/journal.pone.0299259)
Supplement: S1 File — (ZIP) [file pone.0299259.s004.zip › S1 Zip/src/egu00500.html]

egu00500


- egu:105045199

- Up regulated genes

c170749\_g3(0.97516)

- egu:105045199

- Up regulated genes

c170749\_g3(0.97516)

- egu:105057669

- Up regulated genes

c106411\_g1(0.71601)

- egu:105045199

- Up regulated genes

c170749\_g3(0.97516)

- egu:105041389

- Up regulated genes

c168535\_g1(0.5436)

- egu:105057669

- Up regulated genes

c106411\_g1(0.71601)

- egu:105055201

- Up regulated genes

c163448\_g1(0.70197)

- egu:105041628

- Up regulated genes

c144134\_g1(Inf)
- egu:105056861

- Up regulated genes

c152671\_g2(3.6297) c152671\_g1(2.9975)
- egu:105041389

- Up regulated genes

c168535\_g1(0.5436)

- egu:105043204

- Up regulated genes

c79219\_g1(3.3345) c159060\_g1(3.0963)
- egu:105043800

- Up regulated genes

c174721\_g1(7.0539) c111106\_g1(Inf)

- egu:105060892

- Up regulated genes

c173495\_g2(2.5407)

- egu:105043204

- Up regulated genes

c79219\_g1(3.3345) c159060\_g1(3.0963)
- egu:105043800

- Up regulated genes

c174721\_g1(7.0539) c111106\_g1(Inf)

- egu:105060892

- Up regulated genes

c173495\_g2(2.5407)

Close
