# Supplementary material for: Key anti-freeze genes and pathways of Lanzhou lily (Lilium davidii, var. unicolor) during the seedling stage
Source: PLoS One. 2024 Mar 21;19(3):e0299259. doi: 10.1371/journal.pone.0299259 (PMC10956819; doi:10.1371/journal.pone.0299259)
Supplement: S1 File — (ZIP) [file pone.0299259.s004.zip › S1 Zip/src/egu00520.html]

egu00520


- egu:105059691

- Up regulated genes

c154320\_g1(2.0056)
- egu:105059690

- Up regulated genes

c125968\_g1(5.6972) c171764\_g2(5.6213)
- egu:105060320

- Up regulated genes

c172816\_g3(5.9018)
- egu:105047536

- Up regulated genes

c237479\_g1(3.5347) c161991\_g1(2.2688)
- egu:105060220

- Up regulated genes

c172816\_g4(2.2231)
- egu:105047535

- Up regulated genes

c113669\_g1(5.4648) c161991\_g2(1.9362)

- egu:105057669

- Up regulated genes

c106411\_g1(0.71601)

- egu:105059691

- Up regulated genes

c154320\_g1(2.0056)
- egu:105059690

- Up regulated genes

c125968\_g1(5.6972) c171764\_g2(5.6213)
- egu:105060320

- Up regulated genes

c172816\_g3(5.9018)
- egu:105047536

- Up regulated genes

c237479\_g1(3.5347) c161991\_g1(2.2688)
- egu:105060220

- Up regulated genes

c172816\_g4(2.2231)
- egu:105047535

- Up regulated genes

c113669\_g1(5.4648) c161991\_g2(1.9362)

- egu:105057669

- Up regulated genes

c106411\_g1(0.71601)

- egu:105047457

- Up regulated genes

c159575\_g2(0.86066)

- egu:105042572

- Up regulated genes

c163736\_g1(1.0454)

- egu:105055983

- Up regulated genes

c167554\_g2(2.5763) c122873\_g1(1.3716)

- egu:105049059

- Up regulated genes

c164898\_g1(1.4451)

- egu:105057669

- Up regulated genes

c106411\_g1(0.71601)

- egu:105057669

- Up regulated genes

c106411\_g1(0.71601)

- egu:105054659

- Up regulated genes

c164916\_g2(1.1452)

- egu:105057669

- Up regulated genes

c106411\_g1(0.71601)

- egu:105039562

- Up regulated genes

c132414\_g1(Inf)
- egu:105050762

- Up regulated genes

c153668\_g1(1.32) c153668\_g2(1.1586)

- egu:105045517

- Up regulated genes

c166880\_g1(0.70961) c168684\_g1(0.63357)

- egu:105046676

- Up regulated genes

c72023\_g1(1.7299)

Close
