# Supplementary material for: Key anti-freeze genes and pathways of Lanzhou lily (Lilium davidii, var. unicolor) during the seedling stage
Source: PLoS One. 2024 Mar 21;19(3):e0299259. doi: 10.1371/journal.pone.0299259 (PMC10956819; doi:10.1371/journal.pone.0299259)
Supplement: S1 File — (ZIP) [file pone.0299259.s004.zip › S1 Zip/src/egu00561.html]

egu00561


- egu:105057319

- Up regulated genes

c123356\_g1(4.4974) c138851\_g1(4.0766)

- egu:105051810

- Up regulated genes

c164787\_g1(0.64456)
- egu:105042090

- Up regulated genes

c148031\_g1(0.68114)

- egu:105038590

- Up regulated genes

c166846\_g1(1.2816)

Close
