# Supplementary material for: Key anti-freeze genes and pathways of Lanzhou lily (Lilium davidii, var. unicolor) during the seedling stage
Source: PLoS One. 2024 Mar 21;19(3):e0299259. doi: 10.1371/journal.pone.0299259 (PMC10956819; doi:10.1371/journal.pone.0299259)
Supplement: S1 File — (ZIP) [file pone.0299259.s004.zip › S1 Zip/src/egu00564.html]

egu00564


- egu:105061179

- Up regulated genes

c152491\_g2(1.5581)

- egu:105050243

- Up regulated genes

c171054\_g2(3.047)

- egu:105052345

- Up regulated genes

c165891\_g1(0.75464)

- egu:105050243

- Up regulated genes

c171054\_g2(3.047)

- egu:105038590

- Up regulated genes

c166846\_g1(1.2816)

- egu:105033970

- Up regulated genes

c165623\_g2(2.8532)

- egu:105033970

- Up regulated genes

c165623\_g2(2.8532)

- egu:105057319

- Up regulated genes

c123356\_g1(4.4974) c138851\_g1(4.0766)

- egu:105057319

- Up regulated genes

c123356\_g1(4.4974) c138851\_g1(4.0766)

Close
