# Supplementary material for: Key anti-freeze genes and pathways of Lanzhou lily (Lilium davidii, var. unicolor) during the seedling stage
Source: PLoS One. 2024 Mar 21;19(3):e0299259. doi: 10.1371/journal.pone.0299259 (PMC10956819; doi:10.1371/journal.pone.0299259)
Supplement: S1 File — (ZIP) [file pone.0299259.s004.zip › S1 Zip/src/egu00591.html]

egu00591


- egu:105053112

- Up regulated genes

c173762\_g1(7.7551) c157926\_g1(Inf)
- egu:105056718

- Up regulated genes

c162165\_g1(1.1694)
- egu:105041807

- Up regulated genes

c170715\_g1(Inf)

Close
