# Supplementary material for: Key anti-freeze genes and pathways of Lanzhou lily (Lilium davidii, var. unicolor) during the seedling stage
Source: PLoS One. 2024 Mar 21;19(3):e0299259. doi: 10.1371/journal.pone.0299259 (PMC10956819; doi:10.1371/journal.pone.0299259)
Supplement: S1 File — (ZIP) [file pone.0299259.s004.zip › S1 Zip/src/egu00640.html]

egu00640


- egu:105053889

- Up regulated genes

c149905\_g2(0.71101)

- egu:12079399

- Up regulated genes

c164923\_g2(2.2301)

- egu:105053889

- Up regulated genes

c149905\_g2(0.71101)

- egu:105056313

- Up regulated genes

c154026\_g1(0.58616)

Close
