# Supplementary material for: Key anti-freeze genes and pathways of Lanzhou lily (Lilium davidii, var. unicolor) during the seedling stage
Source: PLoS One. 2024 Mar 21;19(3):e0299259. doi: 10.1371/journal.pone.0299259 (PMC10956819; doi:10.1371/journal.pone.0299259)
Supplement: S1 File — (ZIP) [file pone.0299259.s004.zip › S1 Zip/src/egu00770.html]

egu00770


- egu:105047967

- Down regulated genes

c171119\_g1(-0.79494)

- egu:105040792

- Down regulated genes

c156962\_g1(-0.85102)

Close
