# Supplementary material for: Key anti-freeze genes and pathways of Lanzhou lily (Lilium davidii, var. unicolor) during the seedling stage
Source: PLoS One. 2024 Mar 21;19(3):e0299259. doi: 10.1371/journal.pone.0299259 (PMC10956819; doi:10.1371/journal.pone.0299259)
Supplement: S1 File — (ZIP) [file pone.0299259.s004.zip › S1 Zip/src/egu00780.html]

egu00780


- egu:105051936

- Down regulated genes

c152279\_g1(-0.68831)

- egu:105050202

- Down regulated genes

c153334\_g2(-1.1084) c153334\_g1(-1.4904)

- egu:105051936

- Down regulated genes

c152279\_g1(-0.68831)

- egu:105050202

- Down regulated genes

c153334\_g2(-1.1084) c153334\_g1(-1.4904)

- egu:105053059

- Down regulated genes

c164754\_g1(-1.0312)

- egu:105053059

- Down regulated genes

c164754\_g1(-1.0312)

Close
