# Supplementary material for: Key anti-freeze genes and pathways of Lanzhou lily (Lilium davidii, var. unicolor) during the seedling stage
Source: PLoS One. 2024 Mar 21;19(3):e0299259. doi: 10.1371/journal.pone.0299259 (PMC10956819; doi:10.1371/journal.pone.0299259)
Supplement: S1 File — (ZIP) [file pone.0299259.s004.zip › S1 Zip/src/egu00904.html]

egu00904


- egu:105057308

- Up regulated genes

c162153\_g1(4.6489)
- egu:105047400

- Up regulated genes

c166295\_g1(6.3439)

- egu:105057308

- Up regulated genes

c162153\_g1(4.6489)
- egu:105047400

- Up regulated genes

c166295\_g1(6.3439)

- egu:105057308

- Up regulated genes

c162153\_g1(4.6489)
- egu:105047400

- Up regulated genes

c166295\_g1(6.3439)

- egu:105057308

- Up regulated genes

c162153\_g1(4.6489)
- egu:105047400

- Up regulated genes

c166295\_g1(6.3439)

- egu:105032920

- Up regulated genes

c155589\_g1(Inf)

Close
