# Supplementary material for: Key anti-freeze genes and pathways of Lanzhou lily (Lilium davidii, var. unicolor) during the seedling stage
Source: PLoS One. 2024 Mar 21;19(3):e0299259. doi: 10.1371/journal.pone.0299259 (PMC10956819; doi:10.1371/journal.pone.0299259)
Supplement: S1 File — (ZIP) [file pone.0299259.s004.zip › S1 Zip/src/egu00905.html]

egu00905


- egu:105046233

- Down regulated genes

c163288\_g1(-0.70063)

- egu:105033219

- Down regulated genes

c154790\_g1(-0.8608)

- egu:105033219

- Down regulated genes

c154790\_g1(-0.8608)

Close
