# Supplementary material for: Key anti-freeze genes and pathways of Lanzhou lily (Lilium davidii, var. unicolor) during the seedling stage
Source: PLoS One. 2024 Mar 21;19(3):e0299259. doi: 10.1371/journal.pone.0299259 (PMC10956819; doi:10.1371/journal.pone.0299259)
Supplement: S1 File — (ZIP) [file pone.0299259.s004.zip › S1 Zip/src/egu00906.html]

egu00906


- egu:105033850

- Up regulated genes

c173485\_g1(3.8084)

- egu:105035937

- Up regulated genes

c173509\_g1(0.70985)

- egu:105046802

- Up regulated genes

c168117\_g1(1.6213) c162048\_g1(5.8138)

- egu:105046802

- Up regulated genes

c168117\_g1(1.6213) c162048\_g1(5.8138)

Close
