# Supplementary material for: Key anti-freeze genes and pathways of Lanzhou lily (Lilium davidii, var. unicolor) during the seedling stage
Source: PLoS One. 2024 Mar 21;19(3):e0299259. doi: 10.1371/journal.pone.0299259 (PMC10956819; doi:10.1371/journal.pone.0299259)
Supplement: S1 File — (ZIP) [file pone.0299259.s004.zip › S1 Zip/src/egu00940.html]

egu00940


- egu:105035781

- Up regulated genes

c168304\_g1(1.8865)
- egu:105055673

- Up regulated genes

c168304\_g3(4.9024) c168304\_g2(1.9186) c166080\_g1(3.2133)

- egu:105045199

- Up regulated genes

c170749\_g3(0.97516)

- egu:105040597

- Up regulated genes

c166365\_g1(7.6979)
- egu:105040157

- Up regulated genes

c157321\_g1(3.7997)

- egu:105061001

- Up regulated genes

c158521\_g1(0.97781)

- egu:105061001

- Up regulated genes

c158521\_g1(0.97781)

- egu:105061001

- Up regulated genes

c158521\_g1(0.97781)

- egu:105061001

- Up regulated genes

c158521\_g1(0.97781)

- egu:105061001

- Up regulated genes

c158521\_g1(0.97781)

- egu:105040597

- Up regulated genes

c166365\_g1(7.6979)
- egu:105040157

- Up regulated genes

c157321\_g1(3.7997)

- egu:105040597

- Up regulated genes

c166365\_g1(7.6979)
- egu:105040157

- Up regulated genes

c157321\_g1(3.7997)

- egu:105040597

- Up regulated genes

c166365\_g1(7.6979)
- egu:105040157

- Up regulated genes

c157321\_g1(3.7997)

Close
