# Supplementary material for: Key anti-freeze genes and pathways of Lanzhou lily (Lilium davidii, var. unicolor) during the seedling stage
Source: PLoS One. 2024 Mar 21;19(3):e0299259. doi: 10.1371/journal.pone.0299259 (PMC10956819; doi:10.1371/journal.pone.0299259)
Supplement: S1 File — (ZIP) [file pone.0299259.s004.zip › S1 Zip/src/egu00970.html]

egu00970


- egu:105041858

- Down regulated genes

c169070\_g1(-0.91512)

- egu:105055562

- Down regulated genes

c163858\_g1(-0.82688)

- egu:105039813

- Down regulated genes

c161964\_g1(-1.2291)

- egu:105050113

- Down regulated genes

c162019\_g1(-0.86452)

- egu:105041146

- Down regulated genes

c170522\_g1(-1.1359)

- egu:105052855

- Down regulated genes

c161205\_g1(-0.78676)

- egu:105056567

- Down regulated genes

c163317\_g1(-0.6599)
- egu:105055783

- Down regulated genes

c174800\_g1(-0.65748)
- egu:105034995

- Down regulated genes

c132118\_g1(-0.89593)

- egu:105056567

- Down regulated genes

c163317\_g1(-0.6599)
- egu:105055783

- Down regulated genes

c174800\_g1(-0.65748)
- egu:105034995

- Down regulated genes

c132118\_g1(-0.89593)

Close
