# Supplementary material for: Key anti-freeze genes and pathways of Lanzhou lily (Lilium davidii, var. unicolor) during the seedling stage
Source: PLoS One. 2024 Mar 21;19(3):e0299259. doi: 10.1371/journal.pone.0299259 (PMC10956819; doi:10.1371/journal.pone.0299259)
Supplement: S1 File — (ZIP) [file pone.0299259.s004.zip › S1 Zip/src/egu01110.html]

egu01110


- egu:105043024

- Up regulated genes

c153721\_g1(0.60602)

- egu:105047400

- Up regulated genes

c166295\_g1(6.3439)
- egu:105057308

- Up regulated genes

c162153\_g1(4.6489)

- egu:105047400

- Up regulated genes

c166295\_g1(6.3439)
- egu:105057308

- Up regulated genes

c162153\_g1(4.6489)

- egu:105045199

- Up regulated genes

c170749\_g3(0.97516)

- egu:105055201

- Up regulated genes

c163448\_g1(0.70197)

- egu:105038499

- Up regulated genes

c168307\_g1(1.4006)

- egu:105055920

- Up regulated genes

c146039\_g2(Inf)

- egu:105046819

- Up regulated genes

c104726\_g1(1.0852)

- egu:105046819

- Up regulated genes

c104726\_g1(1.0852)

- egu:105046819

- Up regulated genes

c104726\_g1(1.0852)

- egu:105046819

- Up regulated genes

c104726\_g1(1.0852)

- egu:105046819

- Up regulated genes

c104726\_g1(1.0852)

- egu:105046819

- Up regulated genes

c104726\_g1(1.0852)

- egu:105046819

- Up regulated genes

c104726\_g1(1.0852)

- egu:105046819

- Up regulated genes

c104726\_g1(1.0852)

- egu:105046819

- Up regulated genes

c104726\_g1(1.0852)

- egu:105046819

- Up regulated genes

c104726\_g1(1.0852)

- egu:105046819

- Up regulated genes

c104726\_g1(1.0852)

- egu:105053112

- Up regulated genes

c173762\_g1(7.7551) c157926\_g1(Inf)
- egu:105041807

- Up regulated genes

c170715\_g1(Inf)
- egu:105056718

- Up regulated genes

c162165\_g1(1.1694)

- egu:105053112

- Up regulated genes

c173762\_g1(7.7551) c157926\_g1(Inf)
- egu:105041807

- Up regulated genes

c170715\_g1(Inf)
- egu:105056718

- Up regulated genes

c162165\_g1(1.1694)

- egu:105053112

- Up regulated genes

c173762\_g1(7.7551) c157926\_g1(Inf)
- egu:105041807

- Up regulated genes

c170715\_g1(Inf)
- egu:105056718

- Up regulated genes

c162165\_g1(1.1694)

- egu:105053112

- Up regulated genes

c173762\_g1(7.7551) c157926\_g1(Inf)
- egu:105041807

- Up regulated genes

c170715\_g1(Inf)
- egu:105056718

- Up regulated genes

c162165\_g1(1.1694)

- egu:105053112

- Up regulated genes

c173762\_g1(7.7551) c157926\_g1(Inf)
- egu:105041807

- Up regulated genes

c170715\_g1(Inf)
- egu:105056718

- Up regulated genes

c162165\_g1(1.1694)

- egu:105053112

- Up regulated genes

c173762\_g1(7.7551) c157926\_g1(Inf)
- egu:105041807

- Up regulated genes

c170715\_g1(Inf)
- egu:105056718

- Up regulated genes

c162165\_g1(1.1694)

- egu:105053112

- Up regulated genes

c173762\_g1(7.7551) c157926\_g1(Inf)
- egu:105041807

- Up regulated genes

c170715\_g1(Inf)
- egu:105056718

- Up regulated genes

c162165\_g1(1.1694)

- egu:105053112

- Up regulated genes

c173762\_g1(7.7551) c157926\_g1(Inf)
- egu:105041807

- Up regulated genes

c170715\_g1(Inf)
- egu:105056718

- Up regulated genes

c162165\_g1(1.1694)

- egu:105053112

- Up regulated genes

c173762\_g1(7.7551) c157926\_g1(Inf)
- egu:105041807

- Up regulated genes

c170715\_g1(Inf)
- egu:105056718

- Up regulated genes

c162165\_g1(1.1694)

- egu:105053112

- Up regulated genes

c173762\_g1(7.7551) c157926\_g1(Inf)
- egu:105041807

- Up regulated genes

c170715\_g1(Inf)
- egu:105056718

- Up regulated genes

c162165\_g1(1.1694)

- egu:105053112

- Up regulated genes

c173762\_g1(7.7551) c157926\_g1(Inf)
- egu:105041807

- Up regulated genes

c170715\_g1(Inf)
- egu:105056718

- Up regulated genes

c162165\_g1(1.1694)

- egu:105038559

- Up regulated genes

c173723\_g1(0.83659)

- egu:105059131

- Up regulated genes

c156705\_g2(0.75345)

- egu:105059131

- Up regulated genes

c156705\_g2(0.75345)

- egu:105059131

- Up regulated genes

c156705\_g2(0.75345)

- egu:105053112

- Up regulated genes

c173762\_g1(7.7551) c157926\_g1(Inf)
- egu:105041807

- Up regulated genes

c170715\_g1(Inf)
- egu:105056718

- Up regulated genes

c162165\_g1(1.1694)

- egu:105033970

- Up regulated genes

c165623\_g2(2.8532)

- egu:105033970

- Up regulated genes

c165623\_g2(2.8532)

- egu:105033970

- Up regulated genes

c165623\_g2(2.8532)

- egu:105033970

- Up regulated genes

c165623\_g2(2.8532)

- egu:105033970

- Up regulated genes

c165623\_g2(2.8532)

- egu:105033970

- Up regulated genes

c165623\_g2(2.8532)

- egu:105033970

- Up regulated genes

c165623\_g2(2.8532)

- egu:105033970

- Up regulated genes

c165623\_g2(2.8532)

- egu:105033970

- Up regulated genes

c165623\_g2(2.8532)

- egu:105033970

- Up regulated genes

c165623\_g2(2.8532)

- egu:105033970

- Up regulated genes

c165623\_g2(2.8532)

- egu:105033970

- Up regulated genes

c165623\_g2(2.8532)

- egu:105033970

- Up regulated genes

c165623\_g2(2.8532)

- egu:105033970

- Up regulated genes

c165623\_g2(2.8532)

- egu:105033970

- Up regulated genes

c165623\_g2(2.8532)

- egu:105033970

- Up regulated genes

c165623\_g2(2.8532)

- egu:105033970

- Up regulated genes

c165623\_g2(2.8532)

- egu:105033970

- Up regulated genes

c165623\_g2(2.8532)

- egu:105033970

- Up regulated genes

c165623\_g2(2.8532)

- egu:105033970

- Up regulated genes

c165623\_g2(2.8532)

- egu:105033970

- Up regulated genes

c165623\_g2(2.8532)

- egu:105033970

- Up regulated genes

c165623\_g2(2.8532)

- egu:105033970

- Up regulated genes

c165623\_g2(2.8532)

- egu:105033970

- Up regulated genes

c165623\_g2(2.8532)

- egu:105033970

- Up regulated genes

c165623\_g2(2.8532)

- egu:105033970

- Up regulated genes

c165623\_g2(2.8532)

- egu:105033970

- Up regulated genes

c165623\_g2(2.8532)

- egu:105033970

- Up regulated genes

c165623\_g2(2.8532)

- egu:105033970

- Up regulated genes

c165623\_g2(2.8532)

- egu:105033970

- Up regulated genes

c165623\_g2(2.8532)

- egu:105033970

- Up regulated genes

c165623\_g2(2.8532)

- egu:105033970

- Up regulated genes

c165623\_g2(2.8532)

- egu:105033970

- Up regulated genes

c165623\_g2(2.8532)

- egu:105033970

- Up regulated genes

c165623\_g2(2.8532)

- egu:105033970

- Up regulated genes

c165623\_g2(2.8532)

- egu:105033970

- Up regulated genes

c165623\_g2(2.8532)

- egu:105033970

- Up regulated genes

c165623\_g2(2.8532)

- egu:105033970

- Up regulated genes

c165623\_g2(2.8532)

- egu:105033970

- Up regulated genes

c165623\_g2(2.8532)

- egu:105033970

- Up regulated genes

c165623\_g2(2.8532)

- egu:105033970

- Up regulated genes

c165623\_g2(2.8532)

- egu:105033970

- Up regulated genes

c165623\_g2(2.8532)

- egu:105034997

- Up regulated genes

c121798\_g1(1.3576)

- egu:105034997

- Up regulated genes

c121798\_g1(1.3576)

- egu:105034997

- Up regulated genes

c121798\_g1(1.3576)

- egu:105034997

- Up regulated genes

c121798\_g1(1.3576)

- egu:105034997

- Up regulated genes

c121798\_g1(1.3576)

- egu:105034997

- Up regulated genes

c121798\_g1(1.3576)

- egu:105034997

- Up regulated genes

c121798\_g1(1.3576)

- egu:105034997

- Up regulated genes

c121798\_g1(1.3576)

- egu:105034997

- Up regulated genes

c121798\_g1(1.3576)

- egu:105034997

- Up regulated genes

c121798\_g1(1.3576)

- egu:105034997

- Up regulated genes

c121798\_g1(1.3576)

- egu:105059131

- Up regulated genes

c156705\_g2(0.75345)

- egu:105059131

- Up regulated genes

c156705\_g2(0.75345)

- egu:105059131

- Up regulated genes

c156705\_g2(0.75345)

- egu:105059131

- Up regulated genes

c156705\_g2(0.75345)

- egu:105059131

- Up regulated genes

c156705\_g2(0.75345)

- egu:105059131

- Up regulated genes

c156705\_g2(0.75345)

- egu:105059131

- Up regulated genes

c156705\_g2(0.75345)

- egu:105059131

- Up regulated genes

c156705\_g2(0.75345)

- egu:105059131

- Up regulated genes

c156705\_g2(0.75345)

- egu:105059131

- Up regulated genes

c156705\_g2(0.75345)

- egu:105059131

- Up regulated genes

c156705\_g2(0.75345)

- egu:105041005

- Up regulated genes

c144654\_g1(6.9885) c131089\_g1(Inf)

- egu:105041005

- Up regulated genes

c144654\_g1(6.9885) c131089\_g1(Inf)

- egu:105041005

- Up regulated genes

c144654\_g1(6.9885) c131089\_g1(Inf)

- egu:105041005

- Up regulated genes

c144654\_g1(6.9885) c131089\_g1(Inf)

- egu:105041005

- Up regulated genes

c144654\_g1(6.9885) c131089\_g1(Inf)

- egu:105041005

- Up regulated genes

c144654\_g1(6.9885) c131089\_g1(Inf)

- egu:105041005

- Up regulated genes

c144654\_g1(6.9885) c131089\_g1(Inf)

- egu:105041005

- Up regulated genes

c144654\_g1(6.9885) c131089\_g1(Inf)

- egu:105041005

- Up regulated genes

c144654\_g1(6.9885) c131089\_g1(Inf)

- egu:105041005

- Up regulated genes

c144654\_g1(6.9885) c131089\_g1(Inf)

- egu:105041005

- Up regulated genes

c144654\_g1(6.9885) c131089\_g1(Inf)

- egu:105045855

- Up regulated genes

c172556\_g1(1.6166)

- egu:105042489

- Up regulated genes

c156756\_g1(1.0437)
- egu:105038179

- Up regulated genes

c224887\_g1(2.5486)
- egu:105035292

- Up regulated genes

c188298\_g1(2.5083)

- egu:105042090

- Up regulated genes

c148031\_g1(0.68114)
- egu:105051810

- Up regulated genes

c164787\_g1(0.64456)

- egu:105052340

- Up regulated genes

c175256\_g1(1.5585) c151470\_g2(1.6287)
- egu:105046041

- Up regulated genes

c151470\_g3(2.2497)

- egu:105051363

- Up regulated genes

c19061\_g1(3.5186)

- egu:105056077

- Up regulated genes

c160525\_g1(0.9271)

- egu:105035781

- Up regulated genes

c168304\_g1(1.8865)
- egu:105055673

- Up regulated genes

c168304\_g3(4.9024) c168304\_g2(1.9186) c166080\_g1(3.2133)

- egu:105061001

- Up regulated genes

c158521\_g1(0.97781)

- egu:105040157

- Up regulated genes

c157321\_g1(3.7997)
- egu:105040597

- Up regulated genes

c166365\_g1(7.6979)

- egu:105045199

- Up regulated genes

c170749\_g3(0.97516)

- egu:105061001

- Up regulated genes

c158521\_g1(0.97781)

- egu:105061001

- Up regulated genes

c158521\_g1(0.97781)

- egu:105040157

- Up regulated genes

c157321\_g1(3.7997)
- egu:105040597

- Up regulated genes

c166365\_g1(7.6979)

- egu:105061001

- Up regulated genes

c158521\_g1(0.97781)

- egu:105061001

- Up regulated genes

c158521\_g1(0.97781)

- egu:105040157

- Up regulated genes

c157321\_g1(3.7997)
- egu:105040597

- Up regulated genes

c166365\_g1(7.6979)

- egu:105041005

- Up regulated genes

c144654\_g1(6.9885) c131089\_g1(Inf)

- egu:105041005

- Up regulated genes

c144654\_g1(6.9885) c131089\_g1(Inf)

- egu:105041005

- Up regulated genes

c144654\_g1(6.9885) c131089\_g1(Inf)

- egu:105041005

- Up regulated genes

c144654\_g1(6.9885) c131089\_g1(Inf)

- egu:105041005

- Up regulated genes

c144654\_g1(6.9885) c131089\_g1(Inf)

- egu:105041005

- Up regulated genes

c144654\_g1(6.9885) c131089\_g1(Inf)

- egu:105041005

- Up regulated genes

c144654\_g1(6.9885) c131089\_g1(Inf)

- egu:105041005

- Up regulated genes

c144654\_g1(6.9885) c131089\_g1(Inf)

- egu:105041005

- Up regulated genes

c144654\_g1(6.9885) c131089\_g1(Inf)

- egu:105041005

- Up regulated genes

c144654\_g1(6.9885) c131089\_g1(Inf)

- egu:105041005

- Up regulated genes

c144654\_g1(6.9885) c131089\_g1(Inf)

- egu:105048962

- Up regulated genes

c172129\_g1(3.2843)

- egu:105036591

- Up regulated genes

c168403\_g1(1.038)

- egu:105042090

- Up regulated genes

c148031\_g1(0.68114)

- egu:105042489

- Up regulated genes

c156756\_g1(1.0437)
- egu:105038179

- Up regulated genes

c224887\_g1(2.5486)
- egu:105035292

- Up regulated genes

c188298\_g1(2.5083)

- egu:105040082

- Up regulated genes

c162258\_g1(6.8824)
- egu:105044935

- Up regulated genes

c154969\_g2(2.2867)

- egu:105043536

- Up regulated genes

c174640\_g1(7.5505)

- egu:12079399

- Up regulated genes

c164923\_g2(2.2301)

- egu:12079399

- Up regulated genes

c164923\_g2(2.2301)

- egu:12079399

- Up regulated genes

c164923\_g2(2.2301)

- egu:12079399

- Up regulated genes

c164923\_g2(2.2301)

- egu:12079399

- Up regulated genes

c164923\_g2(2.2301)

- egu:12079399

- Up regulated genes

c164923\_g2(2.2301)

- egu:12079399

- Up regulated genes

c164923\_g2(2.2301)

- egu:12079399

- Up regulated genes

c164923\_g2(2.2301)

- egu:12079399

- Up regulated genes

c164923\_g2(2.2301)

- egu:12079399

- Up regulated genes

c164923\_g2(2.2301)

- egu:12079399

- Up regulated genes

c164923\_g2(2.2301)

- egu:12079399

- Up regulated genes

c164923\_g2(2.2301)

- egu:12079399

- Up regulated genes

c164923\_g2(2.2301)

- egu:12079399

- Up regulated genes

c164923\_g2(2.2301)

- egu:12079399

- Up regulated genes

c164923\_g2(2.2301)

- egu:12079399

- Up regulated genes

c164923\_g2(2.2301)

- egu:12079399

- Up regulated genes

c164923\_g2(2.2301)

- egu:12079399

- Up regulated genes

c164923\_g2(2.2301)

- egu:12079399

- Up regulated genes

c164923\_g2(2.2301)

- egu:12079399

- Up regulated genes

c164923\_g2(2.2301)

- egu:12079399

- Up regulated genes

c164923\_g2(2.2301)

- egu:105057669

- Up regulated genes

c106411\_g1(0.71601)

- egu:105057669

- Up regulated genes

c106411\_g1(0.71601)

- egu:105057669

- Up regulated genes

c106411\_g1(0.71601)

- egu:105057669

- Up regulated genes

c106411\_g1(0.71601)

- egu:105057669

- Up regulated genes

c106411\_g1(0.71601)

- egu:105057669

- Up regulated genes

c106411\_g1(0.71601)

- egu:105057669

- Up regulated genes

c106411\_g1(0.71601)

- egu:105057669

- Up regulated genes

c106411\_g1(0.71601)

- egu:105057669

- Up regulated genes

c106411\_g1(0.71601)

- egu:105057669

- Up regulated genes

c106411\_g1(0.71601)

- egu:105057669

- Up regulated genes

c106411\_g1(0.71601)

- egu:105057669

- Up regulated genes

c106411\_g1(0.71601)

- egu:105042699

- Up regulated genes

c163278\_g1(1.5299)
- egu:105040213

- Up regulated genes

c151794\_g1(0.70598)

- egu:105042699

- Up regulated genes

c163278\_g1(1.5299)
- egu:105040213

- Up regulated genes

c151794\_g1(0.70598)

- egu:105042699

- Up regulated genes

c163278\_g1(1.5299)
- egu:105040213

- Up regulated genes

c151794\_g1(0.70598)

- egu:105042699

- Up regulated genes

c163278\_g1(1.5299)
- egu:105040213

- Up regulated genes

c151794\_g1(0.70598)

- egu:105042699

- Up regulated genes

c163278\_g1(1.5299)
- egu:105040213

- Up regulated genes

c151794\_g1(0.70598)

- egu:105042699

- Up regulated genes

c163278\_g1(1.5299)
- egu:105040213

- Up regulated genes

c151794\_g1(0.70598)

- egu:105042699

- Up regulated genes

c163278\_g1(1.5299)
- egu:105040213

- Up regulated genes

c151794\_g1(0.70598)

- egu:105042699

- Up regulated genes

c163278\_g1(1.5299)
- egu:105040213

- Up regulated genes

c151794\_g1(0.70598)

- egu:105042699

- Up regulated genes

c163278\_g1(1.5299)
- egu:105040213

- Up regulated genes

c151794\_g1(0.70598)

- egu:105042699

- Up regulated genes

c163278\_g1(1.5299)
- egu:105040213

- Up regulated genes

c151794\_g1(0.70598)

- egu:105042699

- Up regulated genes

c163278\_g1(1.5299)
- egu:105040213

- Up regulated genes

c151794\_g1(0.70598)

- egu:105042699

- Up regulated genes

c163278\_g1(1.5299)
- egu:105040213

- Up regulated genes

c151794\_g1(0.70598)

- egu:105046802

- Up regulated genes

c162048\_g1(5.8138) c168117\_g1(1.6213)

- egu:105046802

- Up regulated genes

c162048\_g1(5.8138) c168117\_g1(1.6213)

- egu:105046802

- Up regulated genes

c162048\_g1(5.8138) c168117\_g1(1.6213)

- egu:105046802

- Up regulated genes

c162048\_g1(5.8138) c168117\_g1(1.6213)

- egu:105046802

- Up regulated genes

c162048\_g1(5.8138) c168117\_g1(1.6213)

- egu:105046802

- Up regulated genes

c162048\_g1(5.8138) c168117\_g1(1.6213)

- egu:105046802

- Up regulated genes

c162048\_g1(5.8138) c168117\_g1(1.6213)

- egu:105046802

- Up regulated genes

c162048\_g1(5.8138) c168117\_g1(1.6213)

- egu:105046802

- Up regulated genes

c162048\_g1(5.8138) c168117\_g1(1.6213)

- egu:105046802

- Up regulated genes

c162048\_g1(5.8138) c168117\_g1(1.6213)

- egu:105046802

- Up regulated genes

c162048\_g1(5.8138) c168117\_g1(1.6213)

- egu:105035937

- Up regulated genes

c173509\_g1(0.70985)

- egu:105046802

- Up regulated genes

c162048\_g1(5.8138) c168117\_g1(1.6213)

- egu:105045199

- Up regulated genes

c170749\_g3(0.97516)

- egu:105056313

- Up regulated genes

c154026\_g1(0.58616)

- egu:105040155

- Up regulated genes

c134112\_g1(3.5137)
- egu:105036609

- Up regulated genes

c152936\_g1(3.5543)

- egu:105057319

- Up regulated genes

c138851\_g1(4.0766) c123356\_g1(4.4974)

- egu:105038590

- Up regulated genes

c166846\_g1(1.2816)

- egu:105057319

- Up regulated genes

c138851\_g1(4.0766) c123356\_g1(4.4974)

- egu:105057319

- Up regulated genes

c138851\_g1(4.0766) c123356\_g1(4.4974)

- egu:105057319

- Up regulated genes

c138851\_g1(4.0766) c123356\_g1(4.4974)

- egu:105057319

- Up regulated genes

c138851\_g1(4.0766) c123356\_g1(4.4974)

- egu:105057319

- Up regulated genes

c138851\_g1(4.0766) c123356\_g1(4.4974)

- egu:105057319

- Up regulated genes

c138851\_g1(4.0766) c123356\_g1(4.4974)

- egu:105057319

- Up regulated genes

c138851\_g1(4.0766) c123356\_g1(4.4974)

- egu:105057319

- Up regulated genes

c138851\_g1(4.0766) c123356\_g1(4.4974)

- egu:105057319

- Up regulated genes

c138851\_g1(4.0766) c123356\_g1(4.4974)

- egu:105057319

- Up regulated genes

c138851\_g1(4.0766) c123356\_g1(4.4974)

- egu:105057319

- Up regulated genes

c138851\_g1(4.0766) c123356\_g1(4.4974)

Close
