# Supplementary material for: Key anti-freeze genes and pathways of Lanzhou lily (Lilium davidii, var. unicolor) during the seedling stage
Source: PLoS One. 2024 Mar 21;19(3):e0299259. doi: 10.1371/journal.pone.0299259 (PMC10956819; doi:10.1371/journal.pone.0299259)
Supplement: S1 File — (ZIP) [file pone.0299259.s004.zip › S1 Zip/src/egu02010.html]

egu02010


- egu:105059124

- Down regulated genes

c169285\_g1(-1.2329)
- egu:105034865

- Down regulated genes

c163163\_g1(-0.59206)
- egu:105055560

- Down regulated genes

c169285\_g2(-0.85758)

- egu:105052956

- Down regulated genes

c173719\_g3(-1.147)

Close
