# Supplementary material for: Key anti-freeze genes and pathways of Lanzhou lily (Lilium davidii, var. unicolor) during the seedling stage
Source: PLoS One. 2024 Mar 21;19(3):e0299259. doi: 10.1371/journal.pone.0299259 (PMC10956819; doi:10.1371/journal.pone.0299259)
Supplement: S1 File — (ZIP) [file pone.0299259.s004.zip › S1 Zip/src/egu03008.html]

egu03008


- egu:105043116

- Up regulated genes

c145725\_g1(0.68964)

- egu:105053596

- Up regulated genes

c143131\_g1(0.69887)

- egu:105037954

- Up regulated genes

c170819\_g1(0.77308)

- egu:105032038

- Up regulated genes

c165011\_g1(1.0214)

- egu:105046077

- Up regulated genes

c168781\_g1(1.5583)

- egu:105042999

- Up regulated genes

c166893\_g1(0.97707)

- egu:105058499

- Up regulated genes

c167910\_g3(0.74901)

- egu:105059424

- Up regulated genes

c152959\_g1(4.2291)

Close
