# Supplementary material for: Key anti-freeze genes and pathways of Lanzhou lily (Lilium davidii, var. unicolor) during the seedling stage
Source: PLoS One. 2024 Mar 21;19(3):e0299259. doi: 10.1371/journal.pone.0299259 (PMC10956819; doi:10.1371/journal.pone.0299259)
Supplement: S1 File — (ZIP) [file pone.0299259.s004.zip › S1 Zip/src/egu03010.html]

egu03010


- egu:105037979

- Up regulated genes

c133240\_g1(0.94516)

- egu:12079507

- Up regulated genes

c167542\_g1(1.9511)

- egu:105055675

- Up regulated genes

c166320\_g6(2.4552)

- egu:12079413

- Up regulated genes

c131320\_g1(1.2771)

- egu:12079506

- Up regulated genes

c87612\_g1(4.2849)

Close
