# Supplementary material for: Key anti-freeze genes and pathways of Lanzhou lily (Lilium davidii, var. unicolor) during the seedling stage
Source: PLoS One. 2024 Mar 21;19(3):e0299259. doi: 10.1371/journal.pone.0299259 (PMC10956819; doi:10.1371/journal.pone.0299259)
Supplement: S1 File — (ZIP) [file pone.0299259.s004.zip › S1 Zip/src/egu03013.html]

egu03013


- egu:105043116

- Up regulated genes

c145725\_g1(0.68964)

- egu:105043116

- Up regulated genes

c145725\_g1(0.68964)

- egu:105051424

- Up regulated genes

c170817\_g1(4.9526)

- egu:105035083

- Up regulated genes

c169579\_g1(0.74414)

- egu:105052955

- Up regulated genes

c174078\_g4(0.79245)

- egu:105043116

- Up regulated genes

c145725\_g1(0.68964)

- egu:105043116

- Up regulated genes

c145725\_g1(0.68964)

- egu:105051987

- Up regulated genes

c149538\_g1(0.52883)

- egu:105040546

- Up regulated genes

c165981\_g1(0.88444)

Close
