# Supplementary material for: Key anti-freeze genes and pathways of Lanzhou lily (Lilium davidii, var. unicolor) during the seedling stage
Source: PLoS One. 2024 Mar 21;19(3):e0299259. doi: 10.1371/journal.pone.0299259 (PMC10956819; doi:10.1371/journal.pone.0299259)
Supplement: S1 File — (ZIP) [file pone.0299259.s004.zip › S1 Zip/src/egu03018.html]

egu03018


- egu:105033956

- Up regulated genes

c156792\_g1(4.6612)

- egu:105049057

- Up regulated genes

c167403\_g1(0.77339)

Close
