# Supplementary figures and images for: Key anti-freeze genes and pathways of Lanzhou lily (Lilium davidii, var. unicolor) during the seedling stage (part 2 of 2)
Source: PLoS One. 2024 Mar 21;19(3):e0299259. doi: 10.1371/journal.pone.0299259 (PMC10956819; doi:10.1371/journal.pone.0299259)

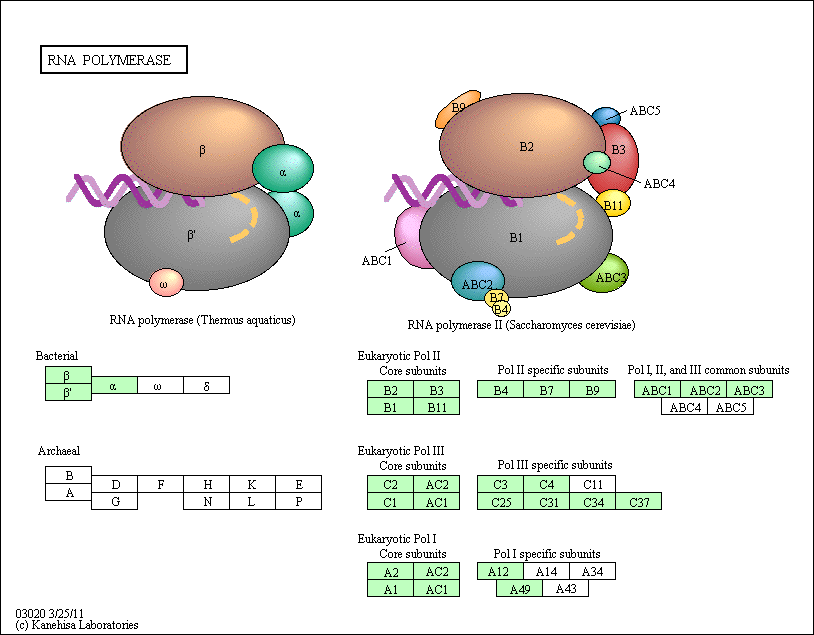

Supplement: S1 File — (ZIP) [file pone.0299259.s004.zip › S1 Zip/src/egu03020.png]

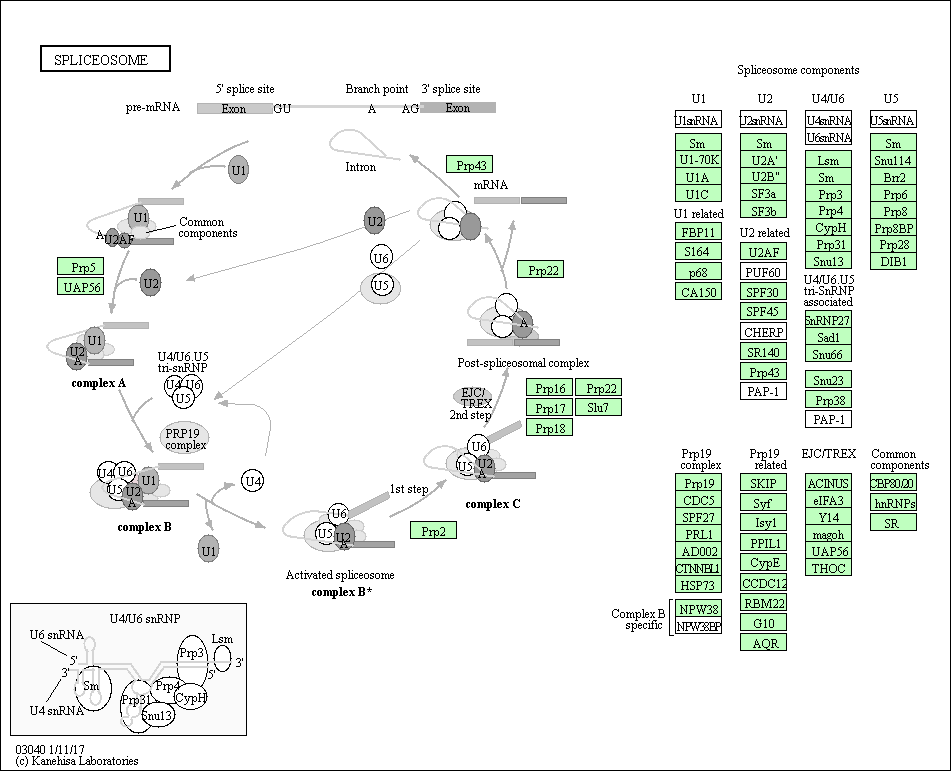

Supplement: S2 File — (ZIP) [file pone.0299259.s005.zip › S2 Zip/src/egu03040.png]

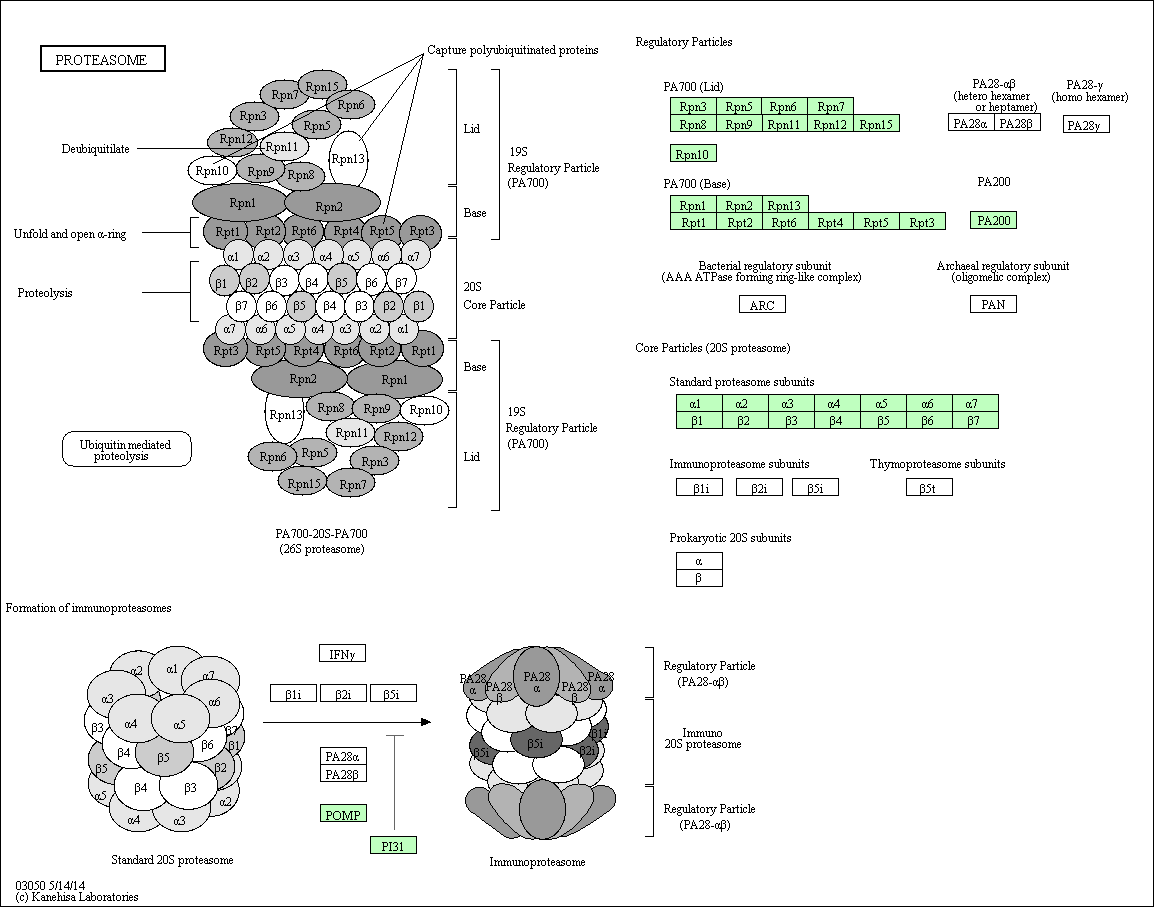

Supplement: S2 File — (ZIP) [file pone.0299259.s005.zip › S2 Zip/src/egu03050.png]

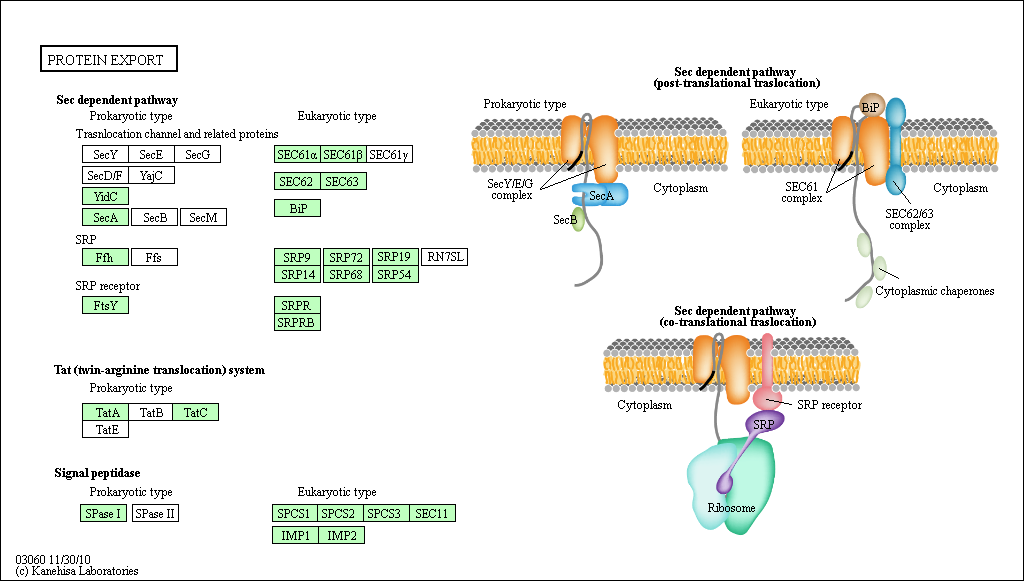

Supplement: S2 File — (ZIP) [file pone.0299259.s005.zip › S2 Zip/src/egu03060.png]

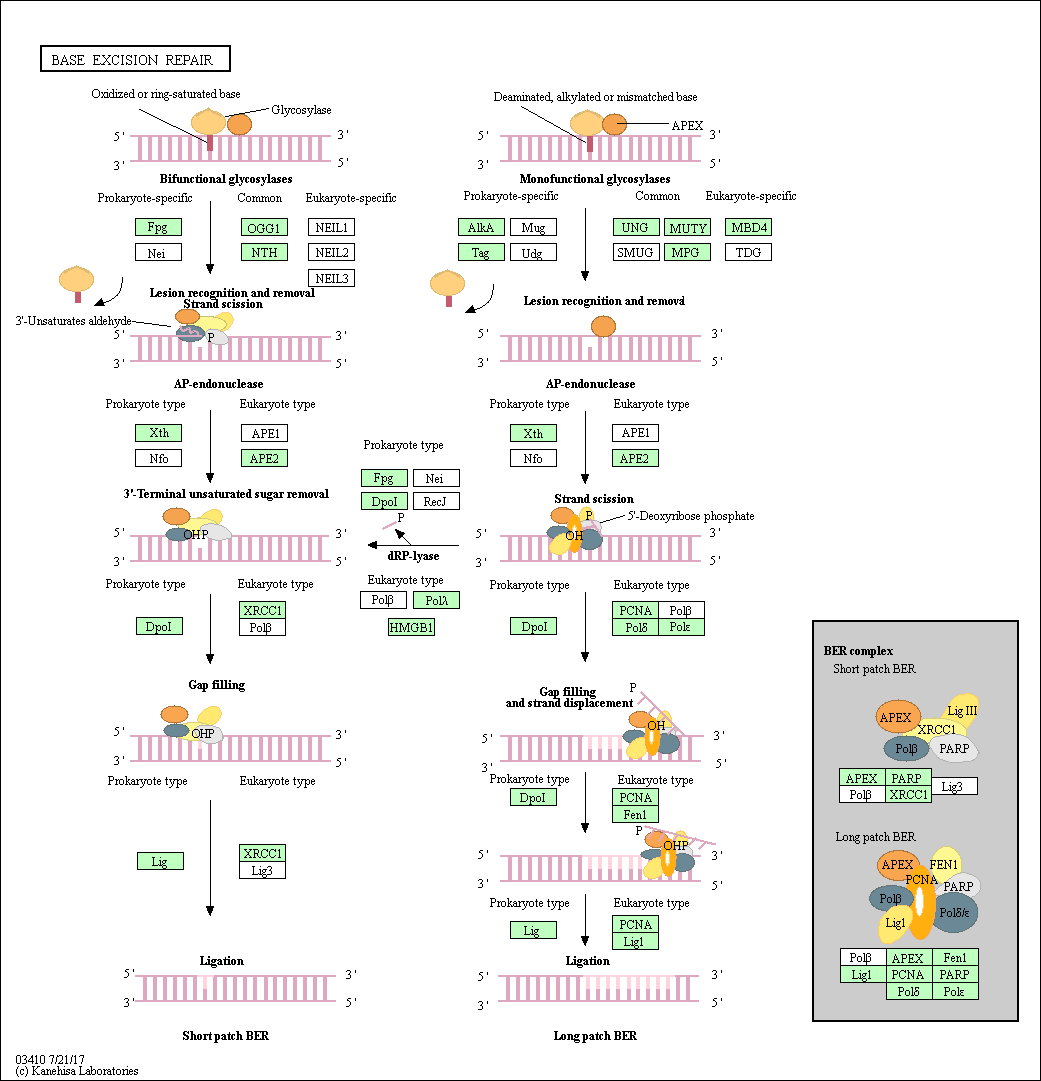

Supplement: S2 File — (ZIP) [file pone.0299259.s005.zip › S2 Zip/src/egu03410.png]

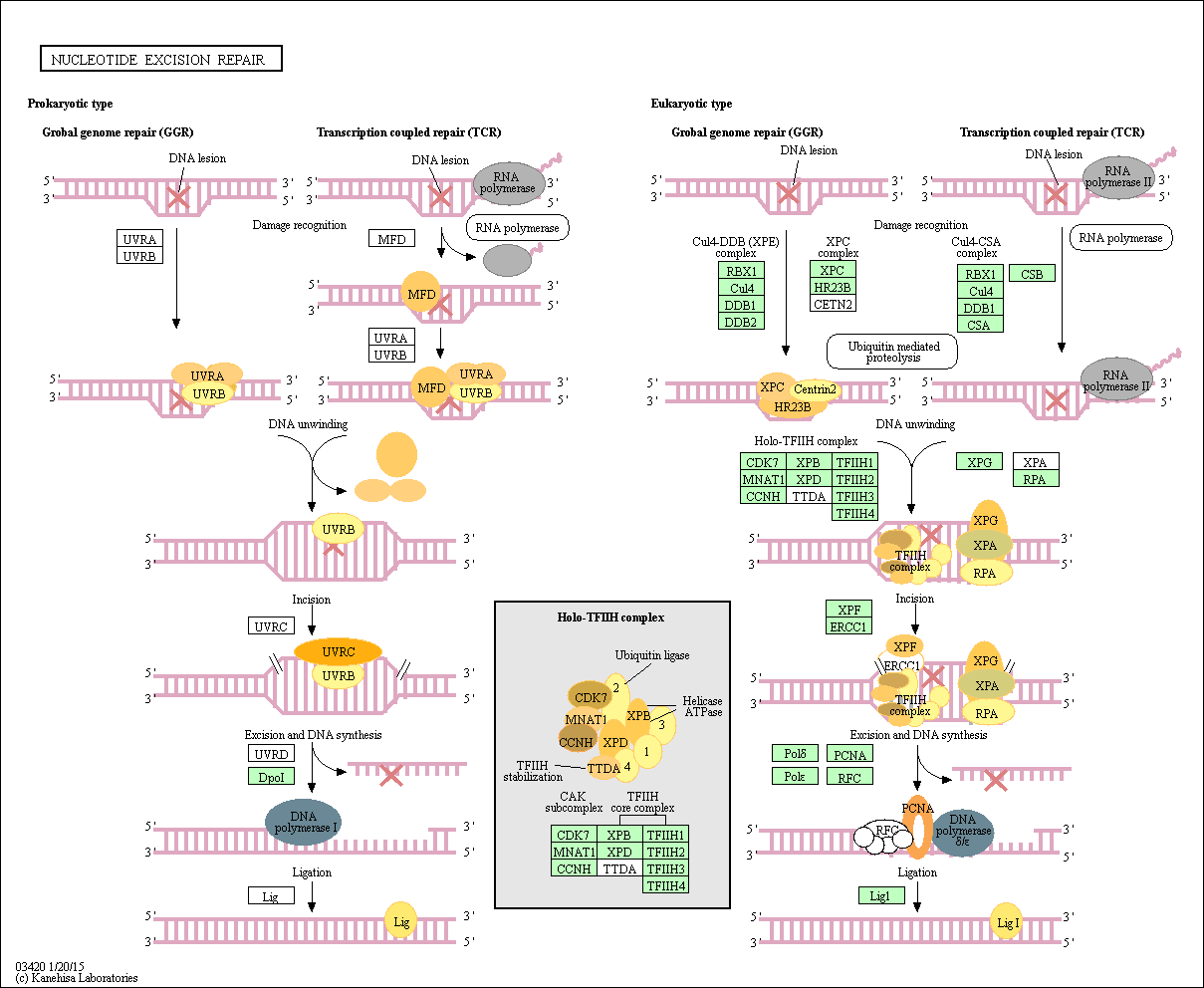

Supplement: S2 File — (ZIP) [file pone.0299259.s005.zip › S2 Zip/src/egu03420.png]

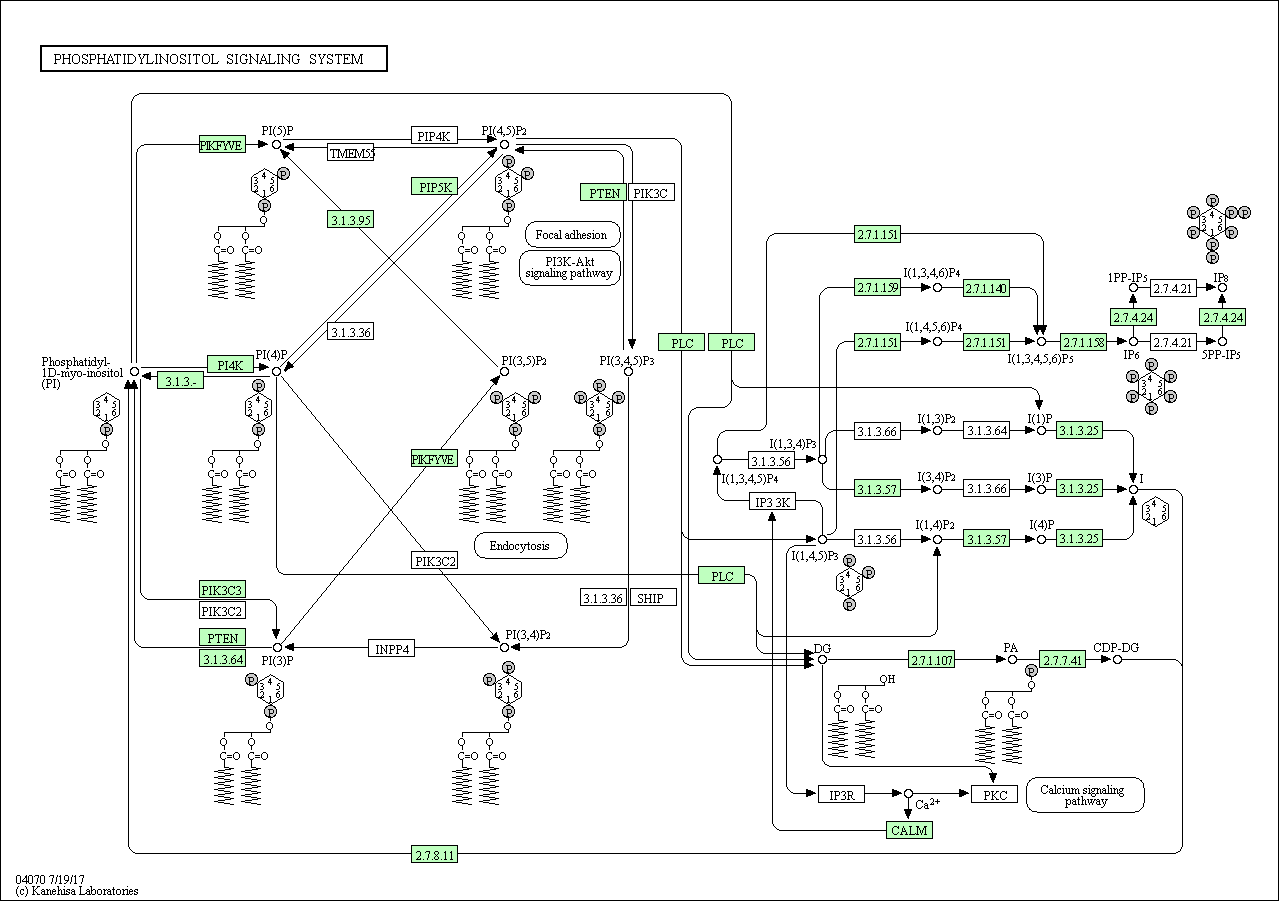

Supplement: S2 File — (ZIP) [file pone.0299259.s005.zip › S2 Zip/src/egu04070.png]

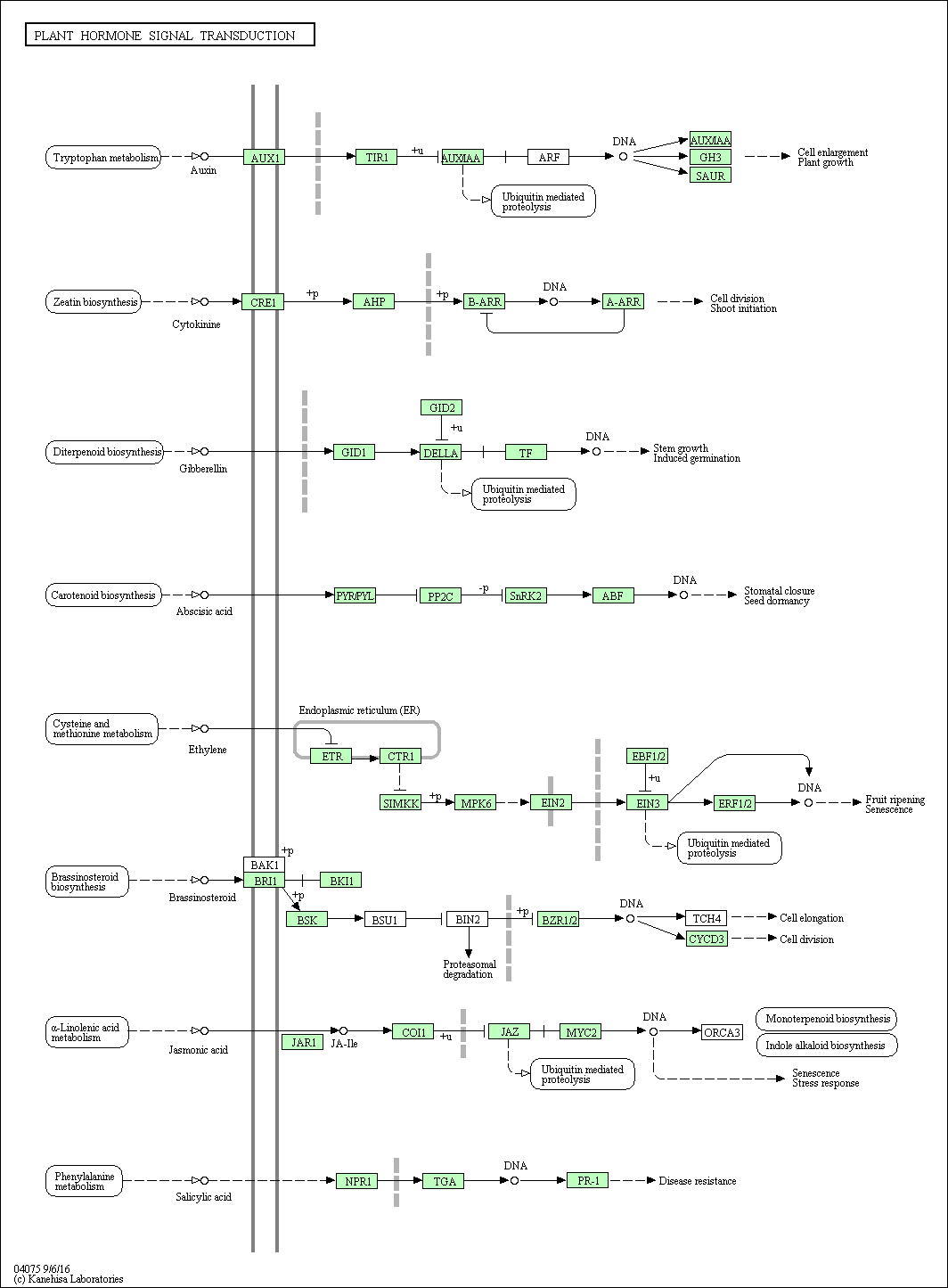

Supplement: S2 File — (ZIP) [file pone.0299259.s005.zip › S2 Zip/src/egu04075.png]

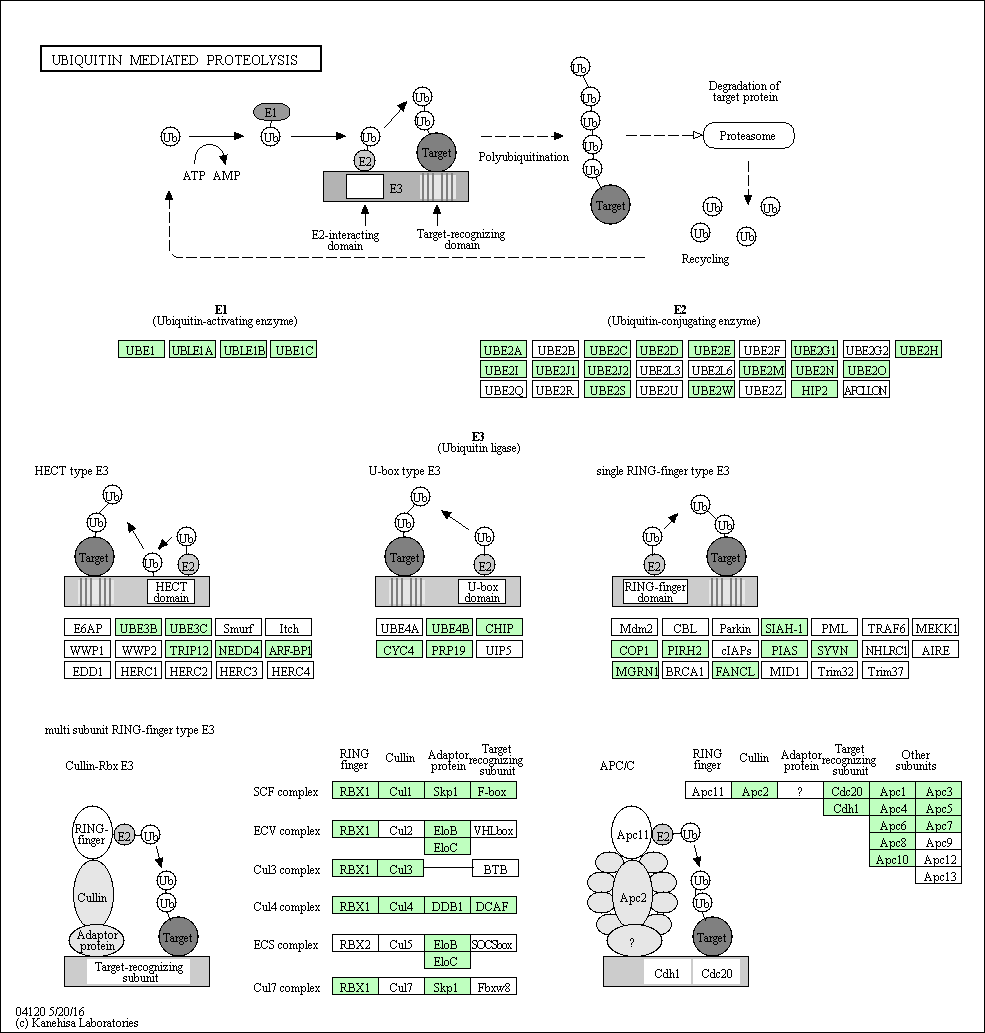

Supplement: S2 File — (ZIP) [file pone.0299259.s005.zip › S2 Zip/src/egu04120.png]

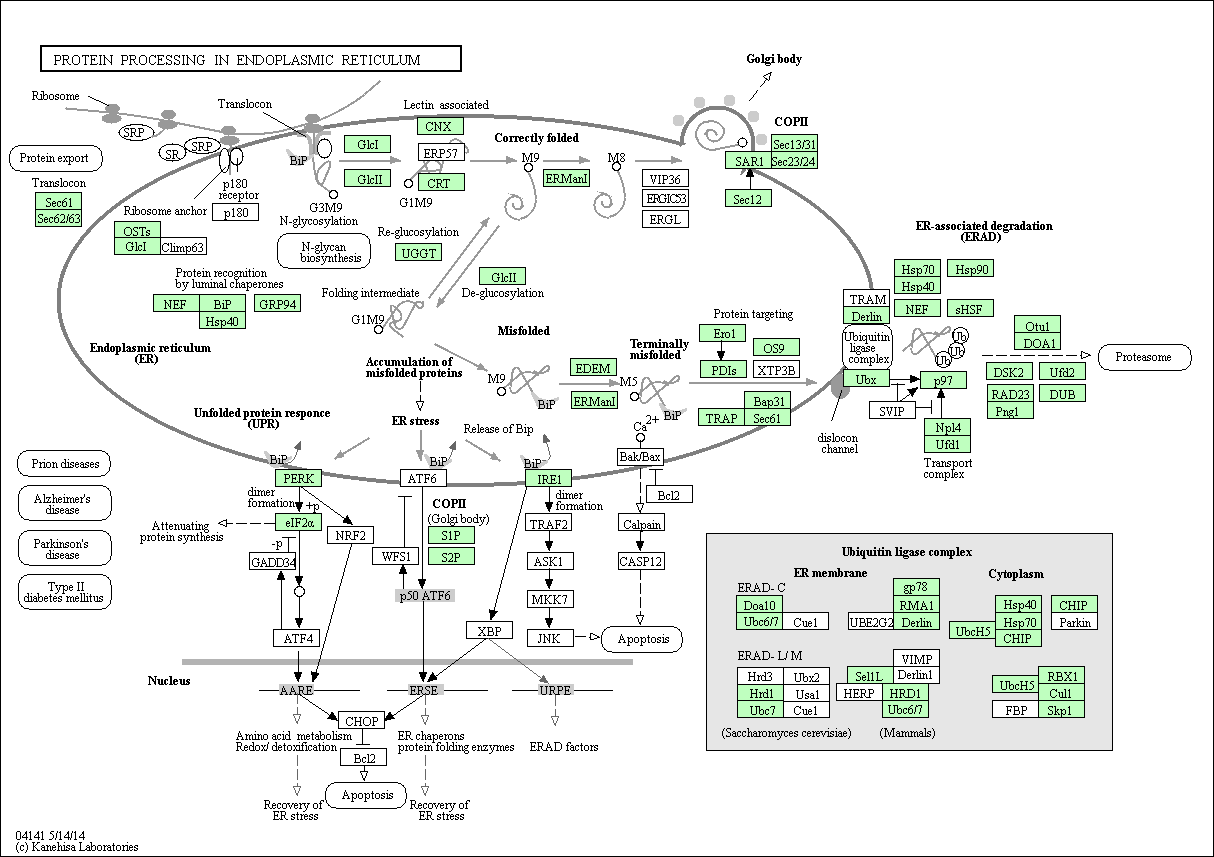

Supplement: S2 File — (ZIP) [file pone.0299259.s005.zip › S2 Zip/src/egu04141.png]
