# Supplementary figures and images for: Key anti-freeze genes and pathways of Lanzhou lily (Lilium davidii, var. unicolor) during the seedling stage (part 1 of 2)
Source: PLoS One. 2024 Mar 21;19(3):e0299259. doi: 10.1371/journal.pone.0299259 (PMC10956819; doi:10.1371/journal.pone.0299259)

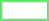

Supplement: S1 File — (ZIP) [file pone.0299259.s004.zip › S1 Zip/src/bg_green.png]

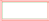

Supplement: S1 File — (ZIP) [file pone.0299259.s004.zip › S1 Zip/src/bg_pink.png]

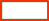

Supplement: S1 File — (ZIP) [file pone.0299259.s004.zip › S1 Zip/src/bg_red.png]

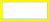

Supplement: S1 File — (ZIP) [file pone.0299259.s004.zip › S1 Zip/src/bg_yellow.png]

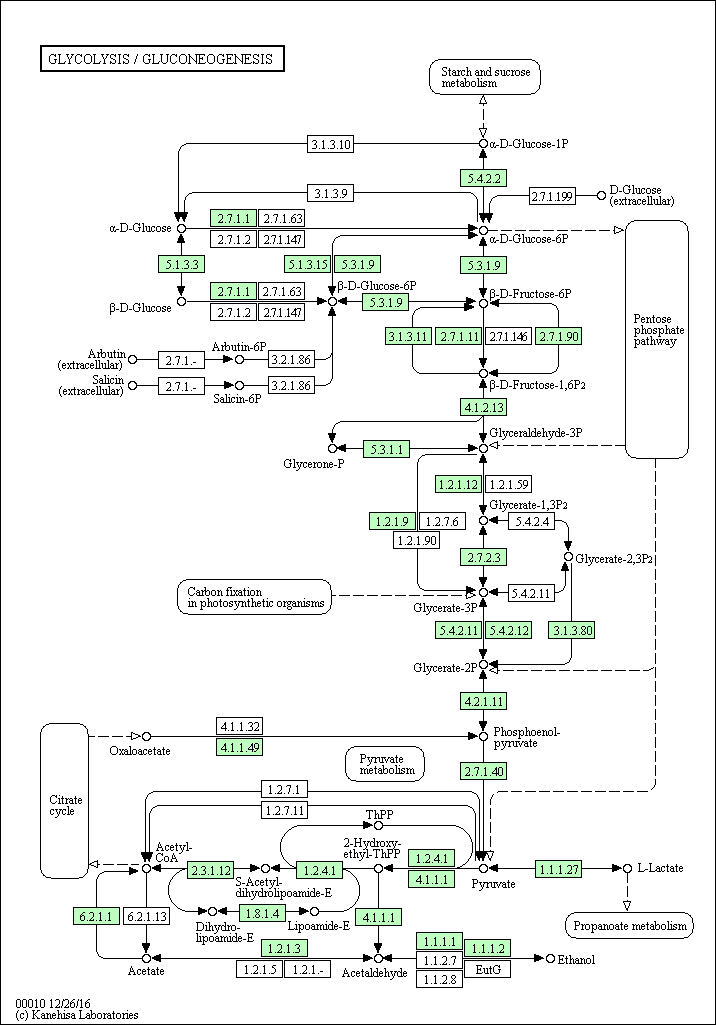

Supplement: S1 File — (ZIP) [file pone.0299259.s004.zip › S1 Zip/src/egu00010.png]

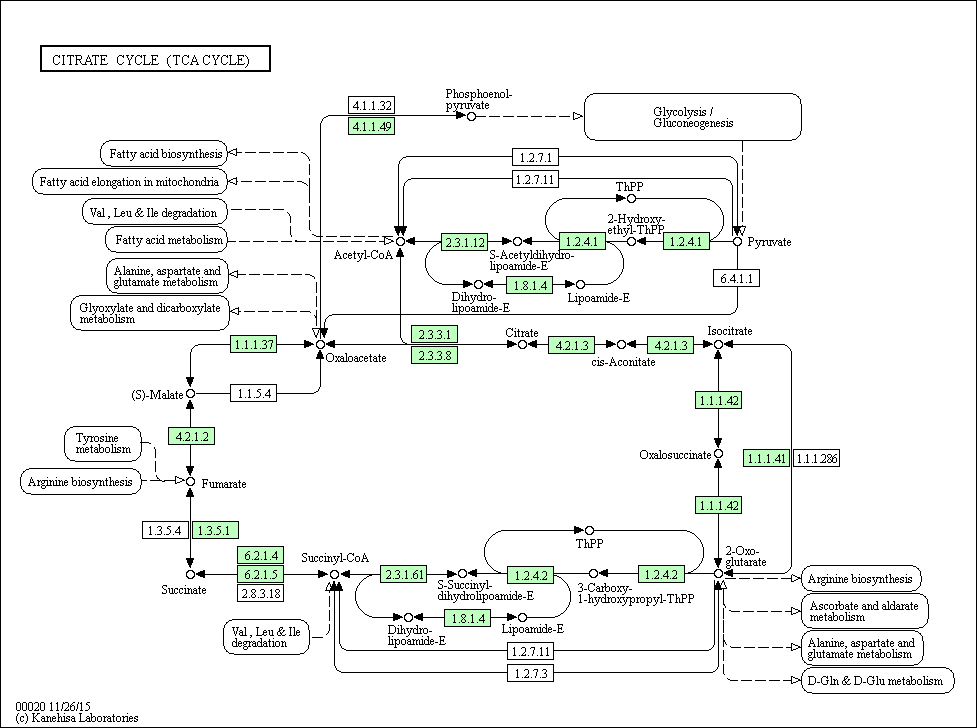

Supplement: S1 File — (ZIP) [file pone.0299259.s004.zip › S1 Zip/src/egu00020.png]

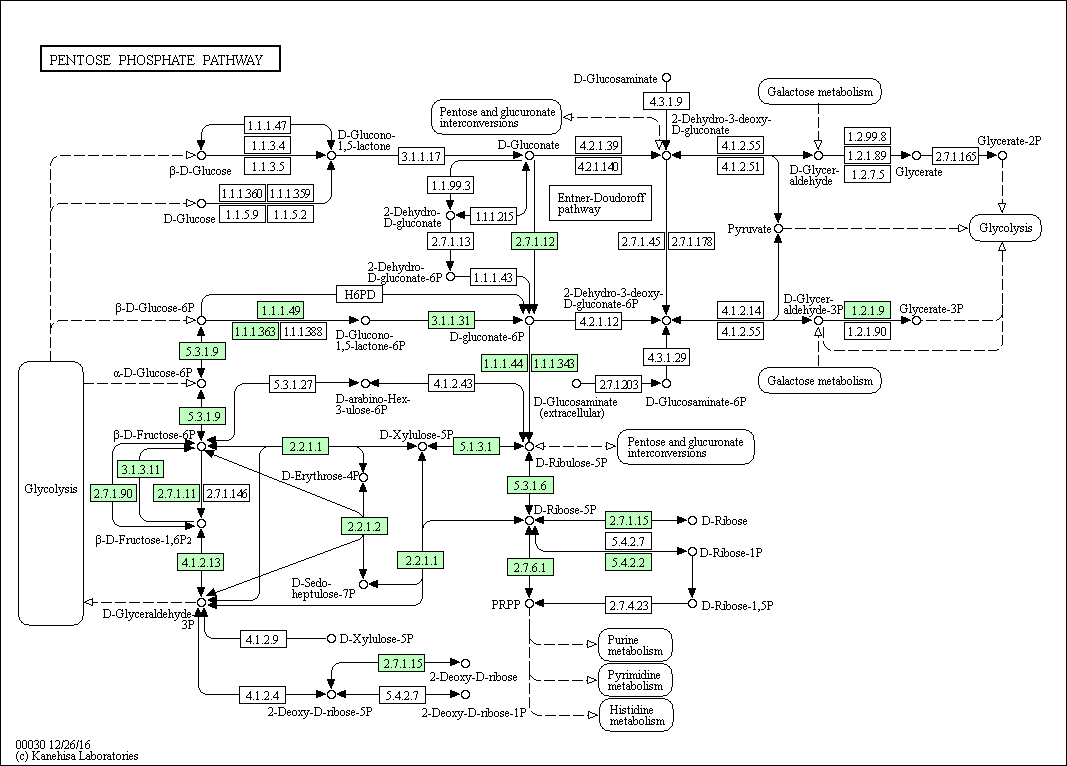

Supplement: S1 File — (ZIP) [file pone.0299259.s004.zip › S1 Zip/src/egu00030.png]

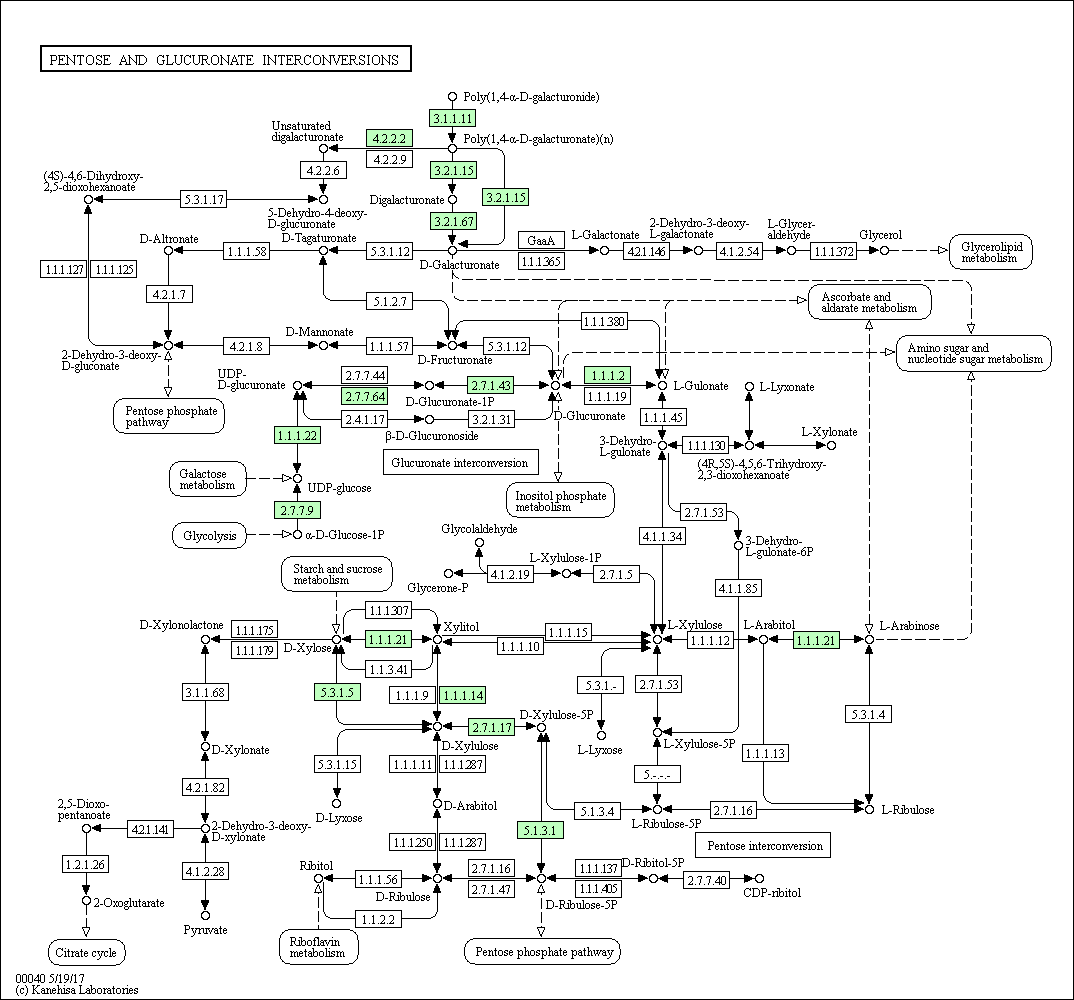

Supplement: S1 File — (ZIP) [file pone.0299259.s004.zip › S1 Zip/src/egu00040.png]

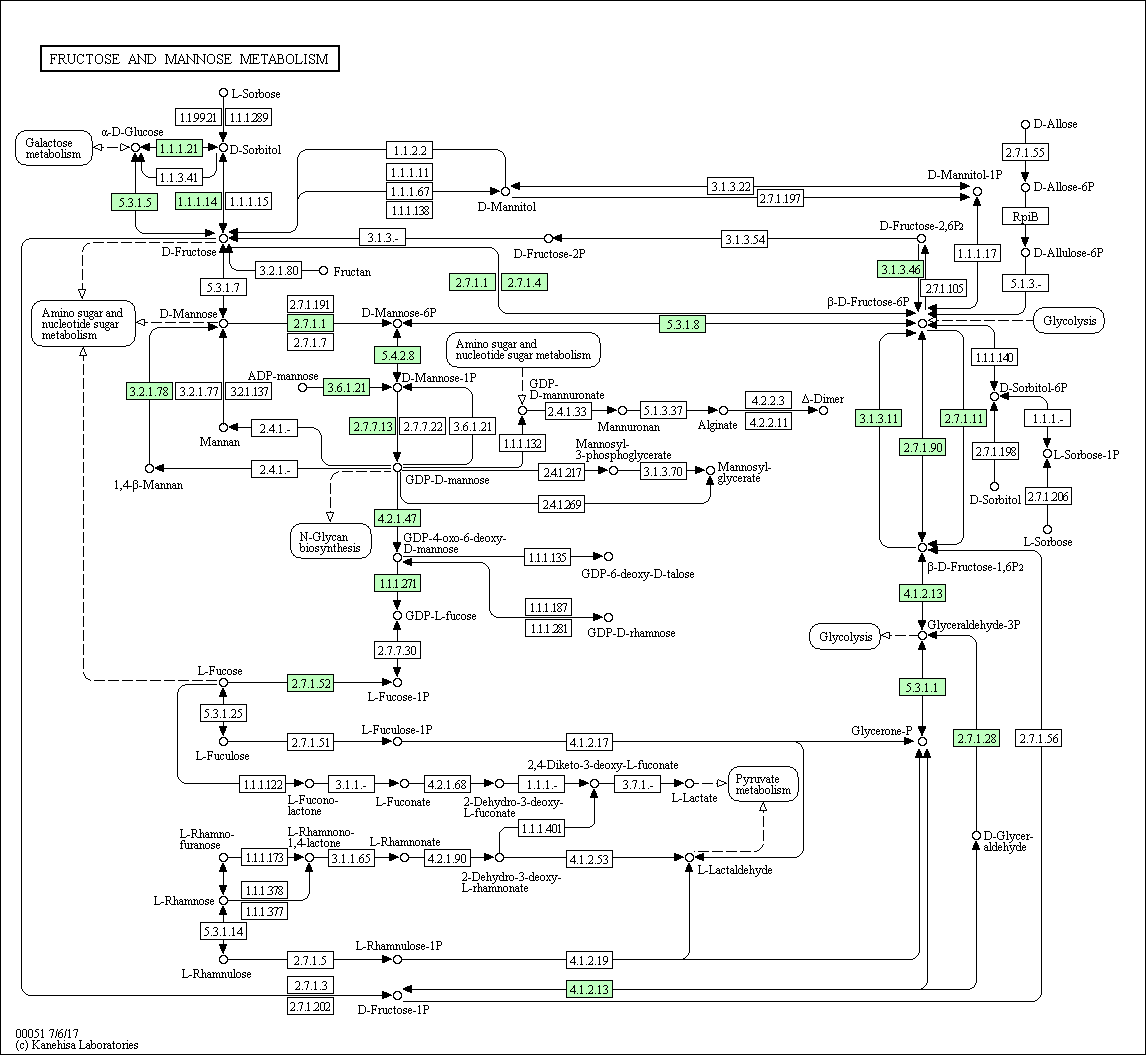

Supplement: S1 File — (ZIP) [file pone.0299259.s004.zip › S1 Zip/src/egu00051.png]

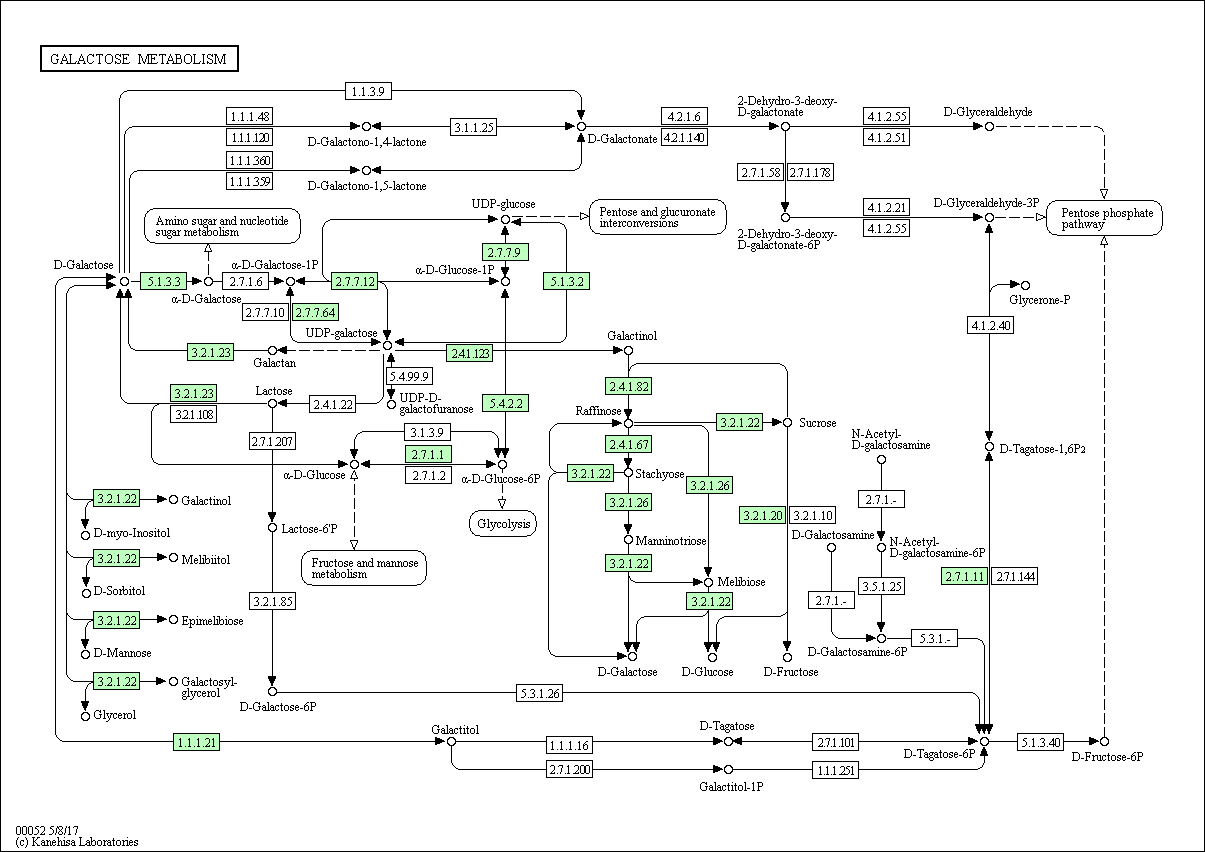

Supplement: S1 File — (ZIP) [file pone.0299259.s004.zip › S1 Zip/src/egu00052.png]

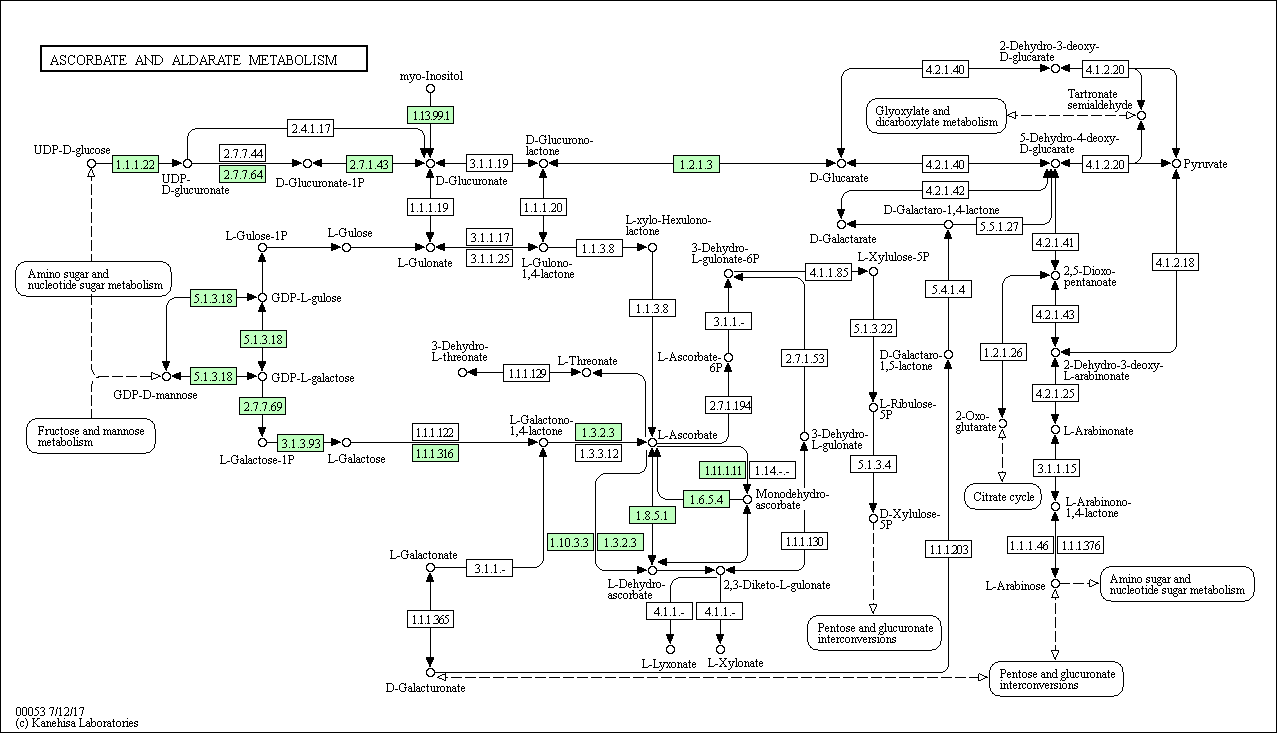

Supplement: S1 File — (ZIP) [file pone.0299259.s004.zip › S1 Zip/src/egu00053.png]

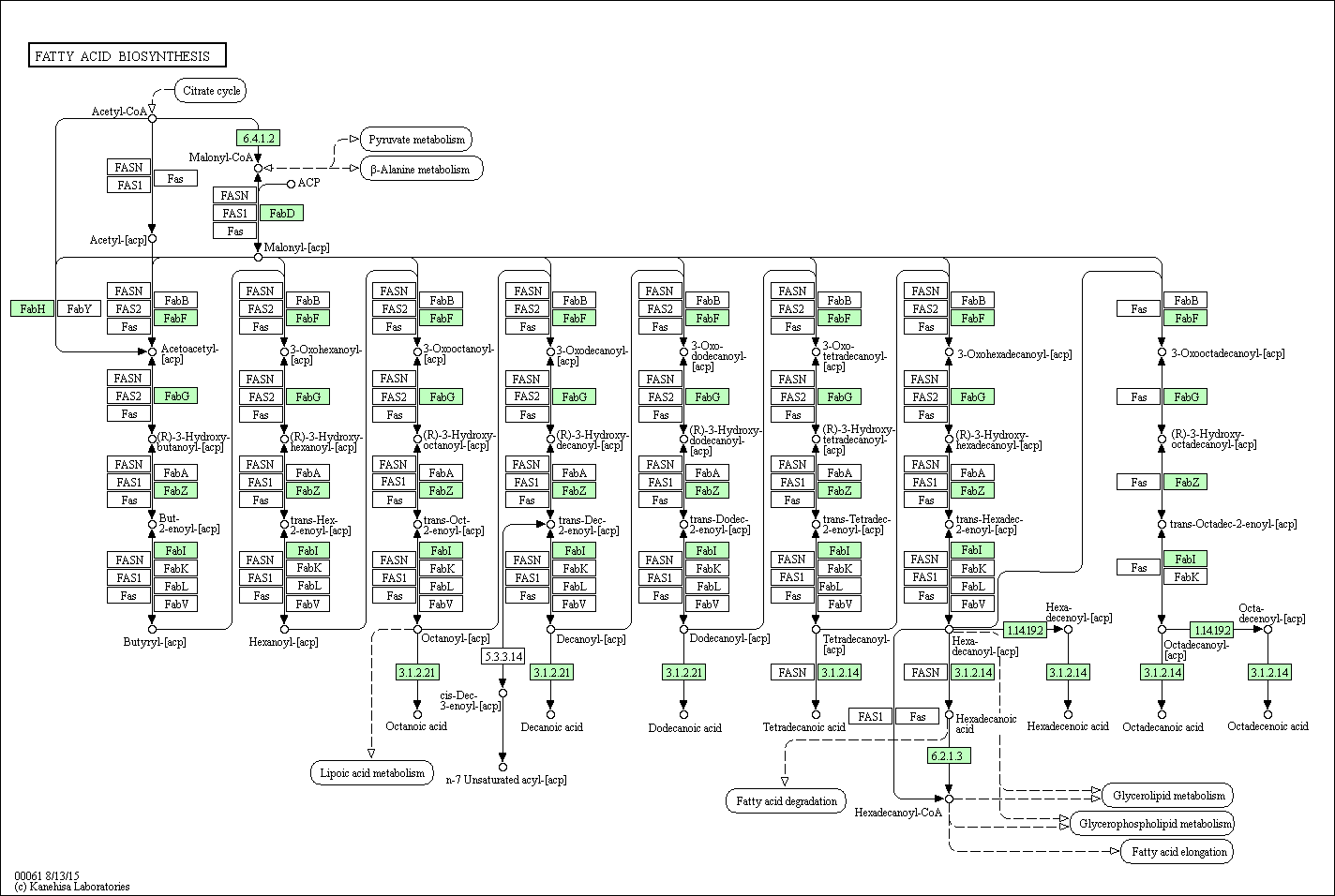

Supplement: S1 File — (ZIP) [file pone.0299259.s004.zip › S1 Zip/src/egu00061.png]

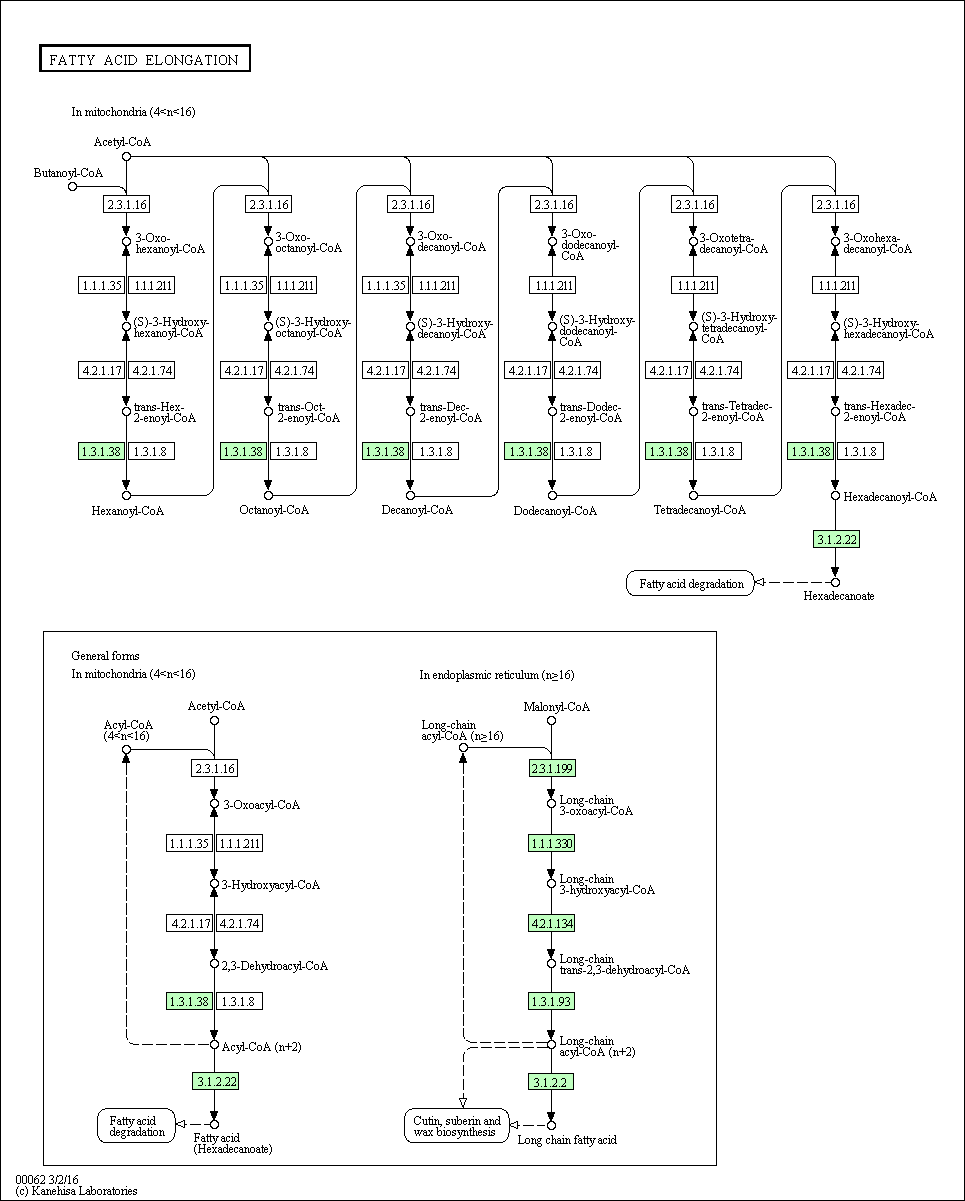

Supplement: S1 File — (ZIP) [file pone.0299259.s004.zip › S1 Zip/src/egu00062.png]

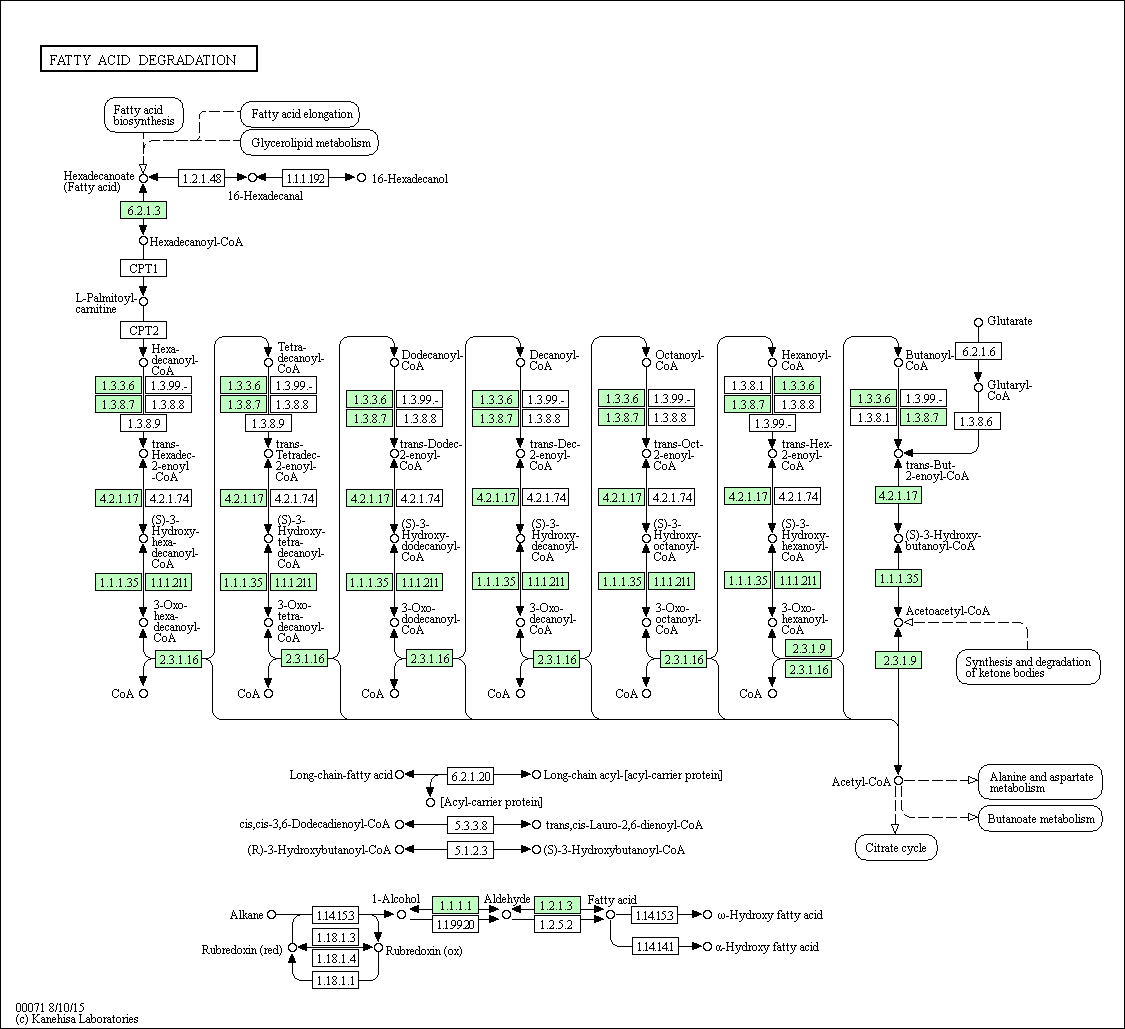

Supplement: S1 File — (ZIP) [file pone.0299259.s004.zip › S1 Zip/src/egu00071.png]

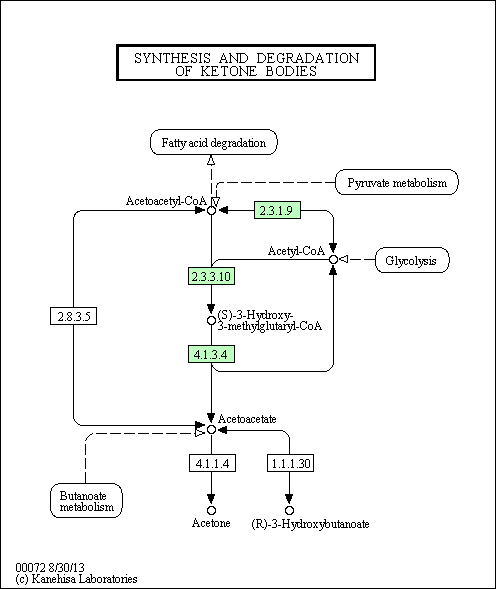

Supplement: S1 File — (ZIP) [file pone.0299259.s004.zip › S1 Zip/src/egu00072.png]

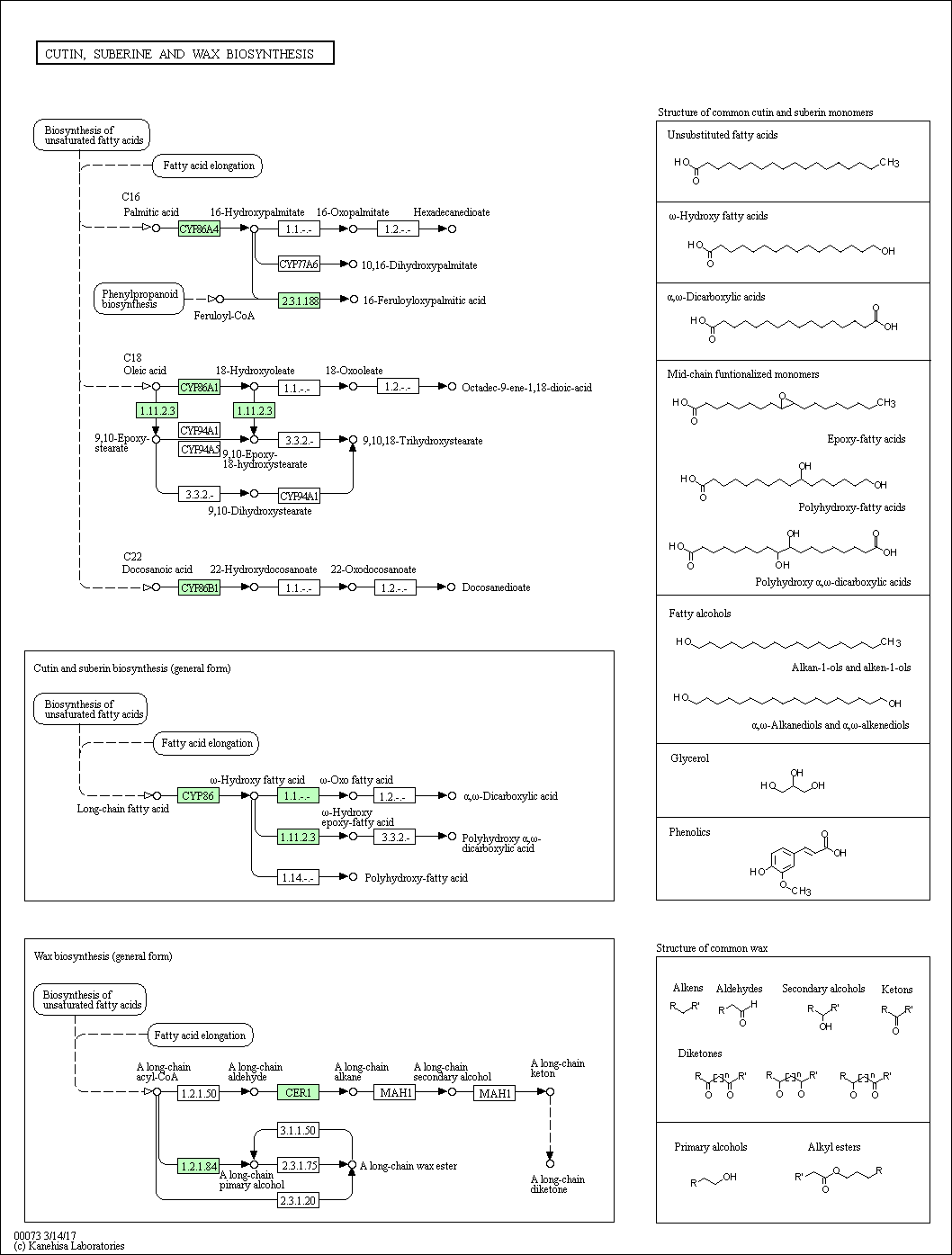

Supplement: S1 File — (ZIP) [file pone.0299259.s004.zip › S1 Zip/src/egu00073.png]

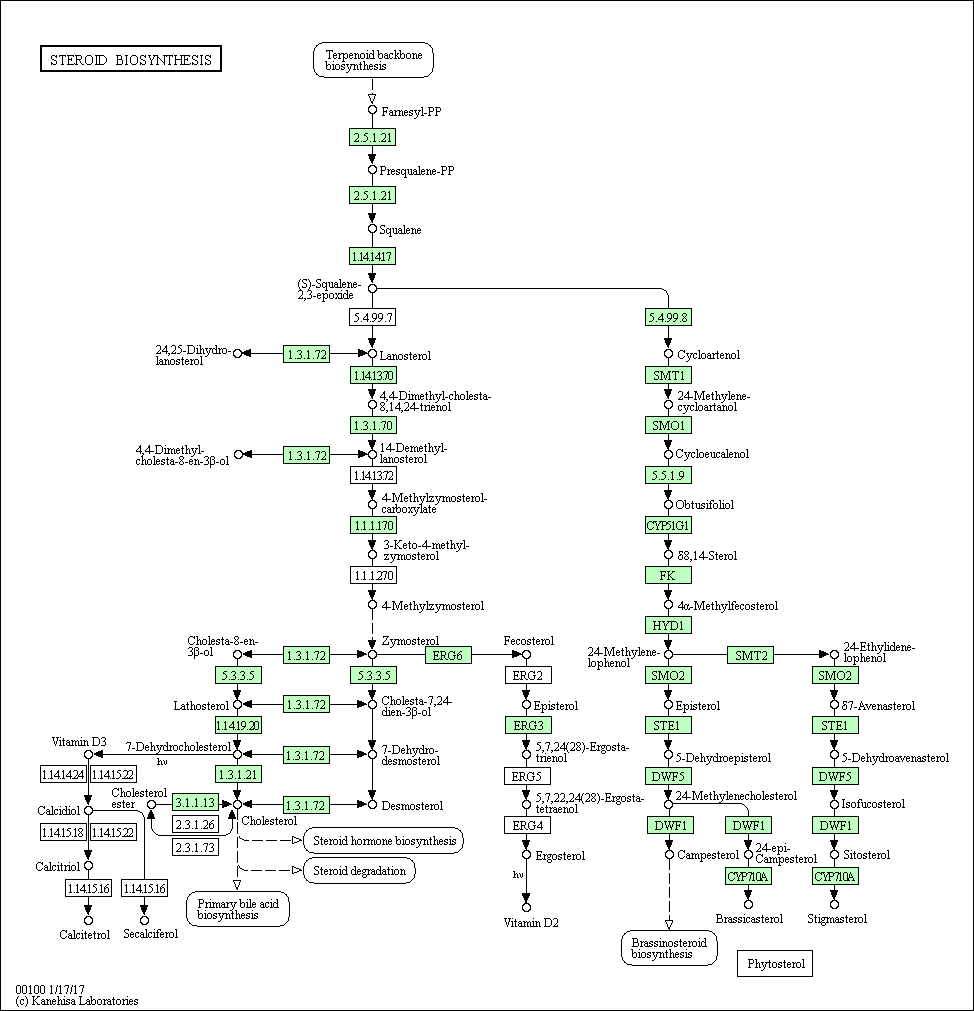

Supplement: S1 File — (ZIP) [file pone.0299259.s004.zip › S1 Zip/src/egu00100.png]

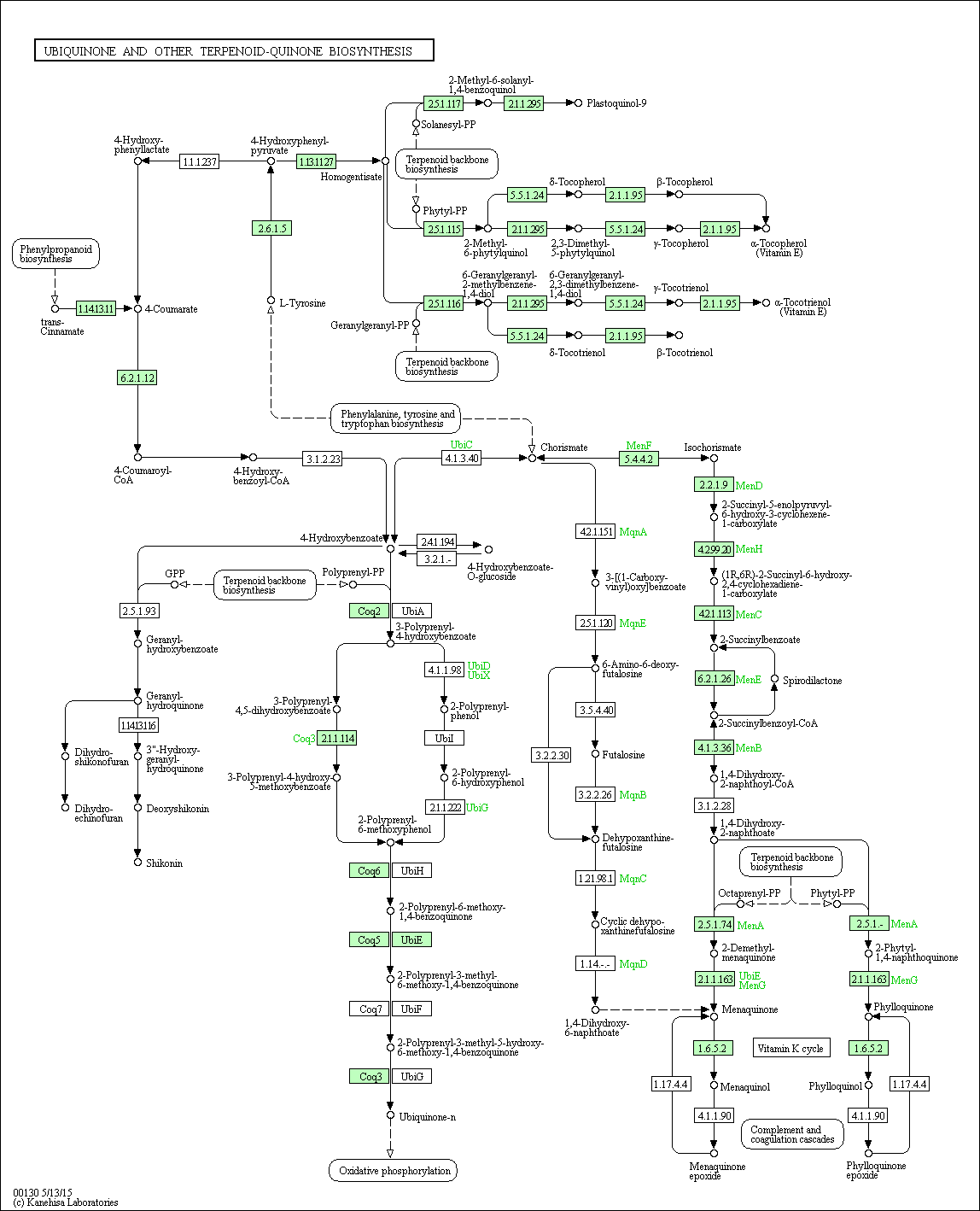

Supplement: S1 File — (ZIP) [file pone.0299259.s004.zip › S1 Zip/src/egu00130.png]

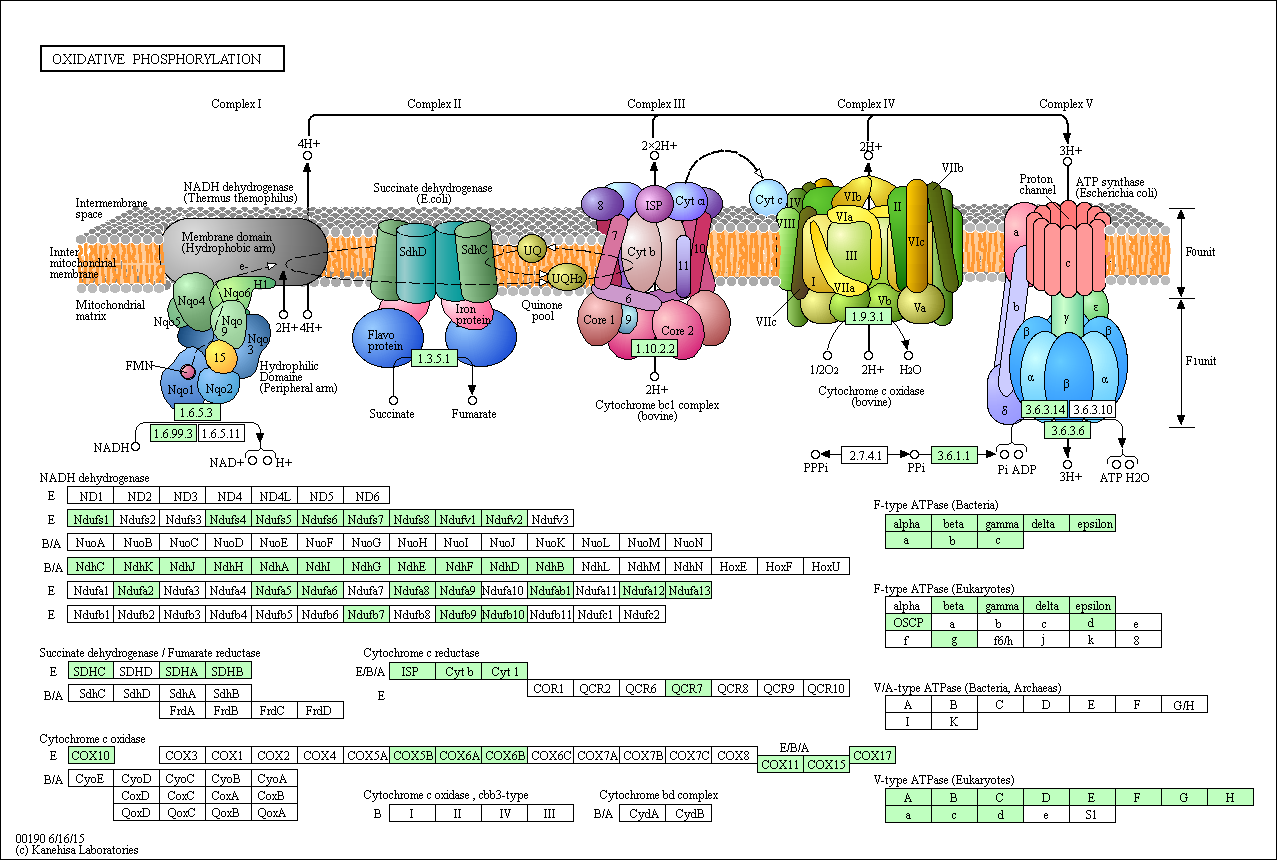

Supplement: S1 File — (ZIP) [file pone.0299259.s004.zip › S1 Zip/src/egu00190.png]

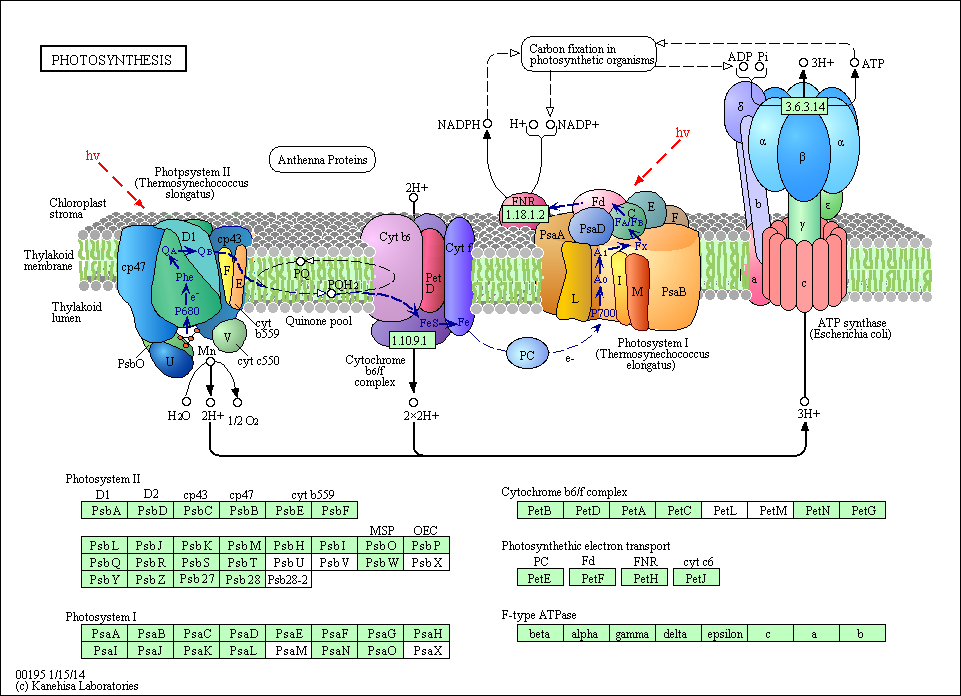

Supplement: S1 File — (ZIP) [file pone.0299259.s004.zip › S1 Zip/src/egu00195.png]

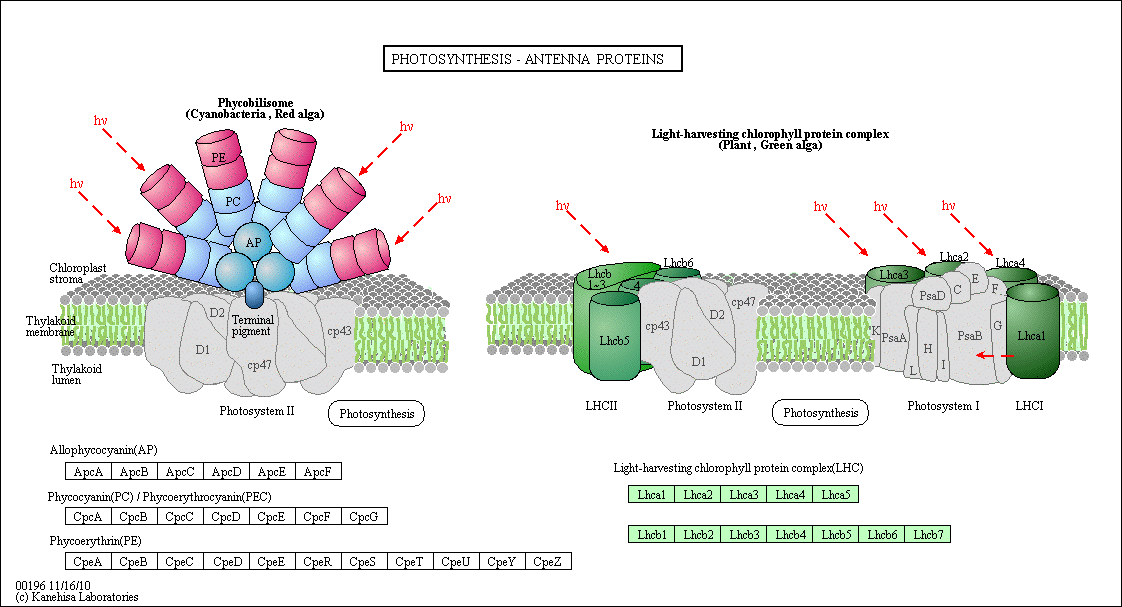

Supplement: S1 File — (ZIP) [file pone.0299259.s004.zip › S1 Zip/src/egu00196.png]

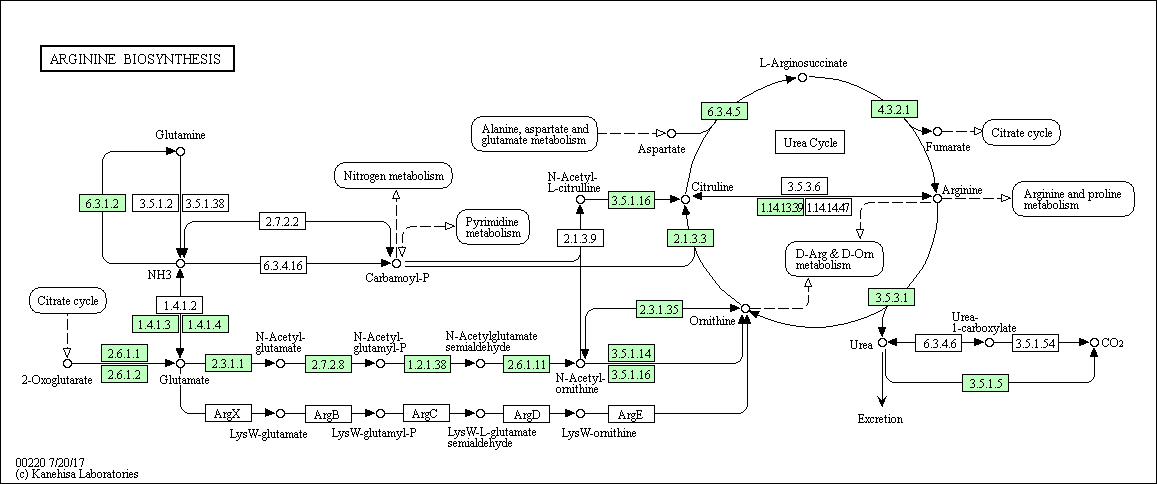

Supplement: S1 File — (ZIP) [file pone.0299259.s004.zip › S1 Zip/src/egu00220.png]

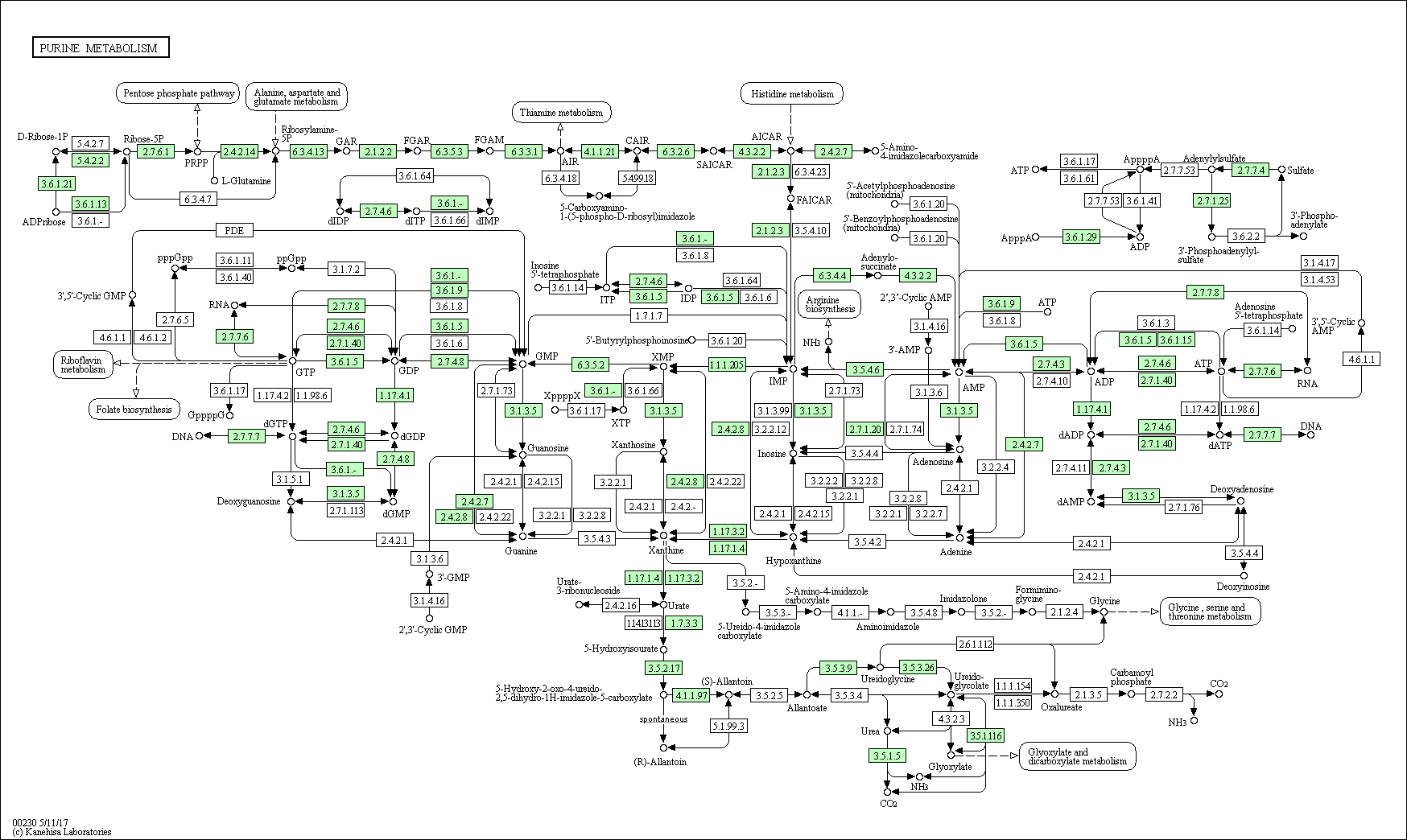

Supplement: S1 File — (ZIP) [file pone.0299259.s004.zip › S1 Zip/src/egu00230.png]

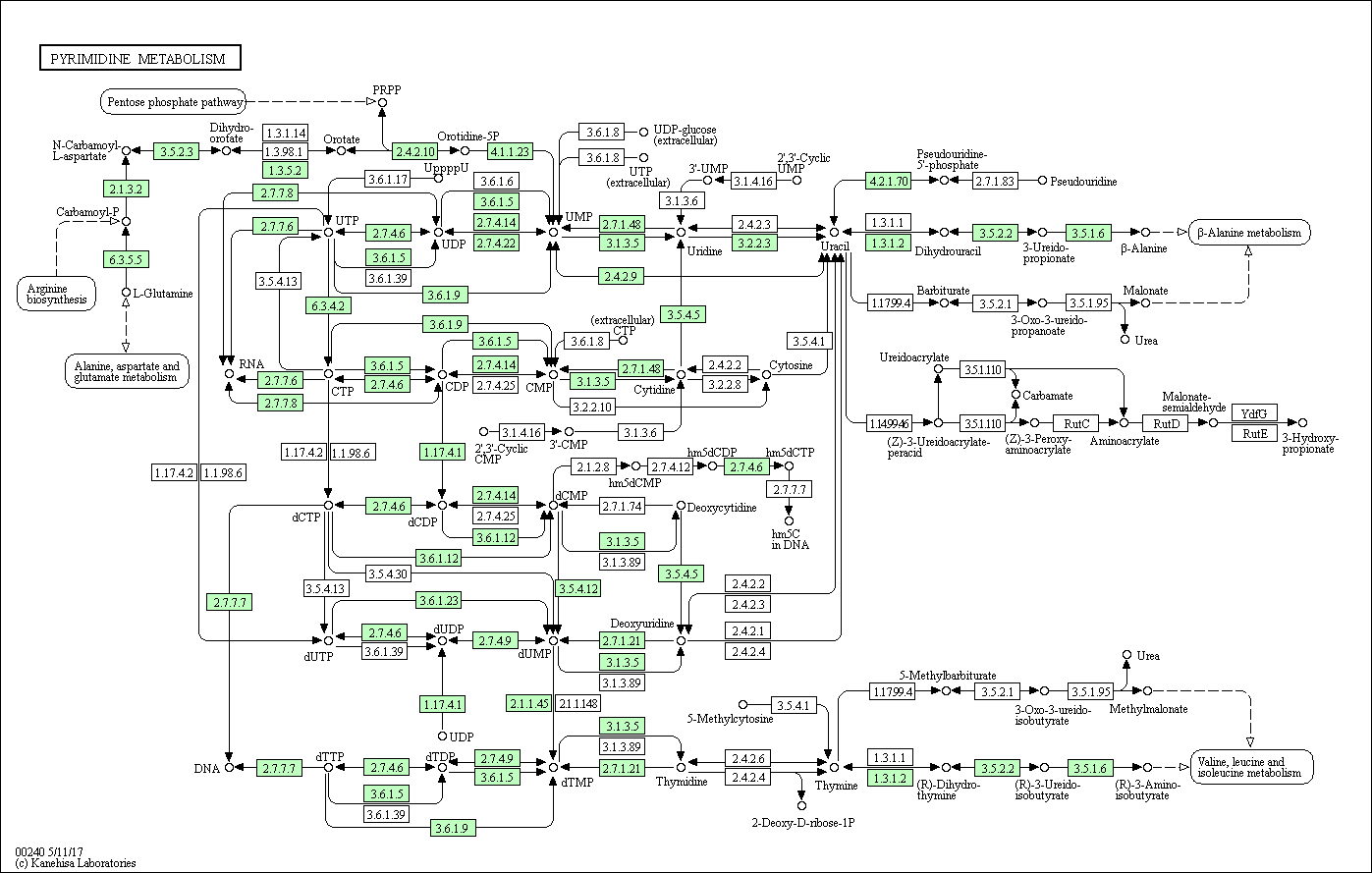

Supplement: S1 File — (ZIP) [file pone.0299259.s004.zip › S1 Zip/src/egu00240.png]

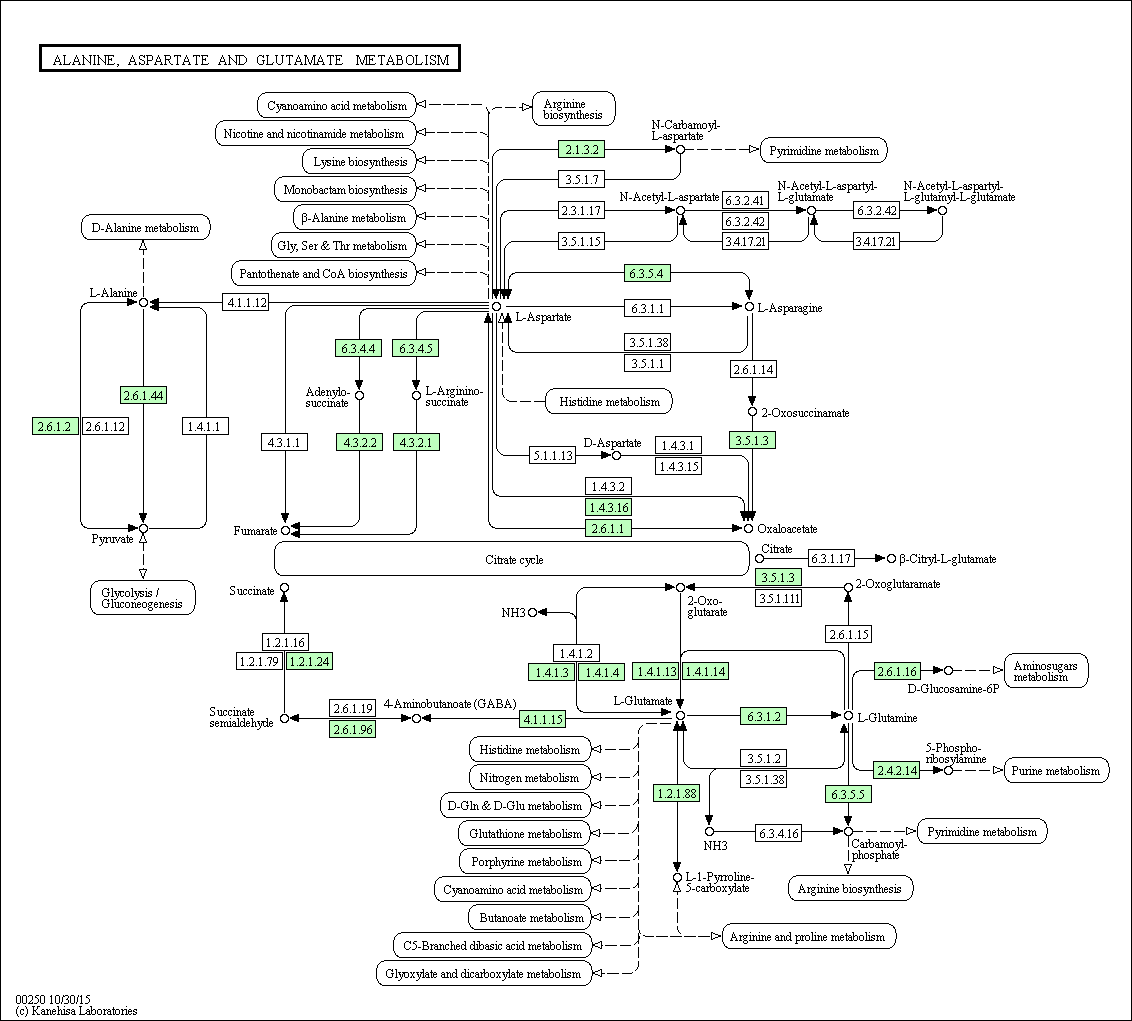

Supplement: S1 File — (ZIP) [file pone.0299259.s004.zip › S1 Zip/src/egu00250.png]

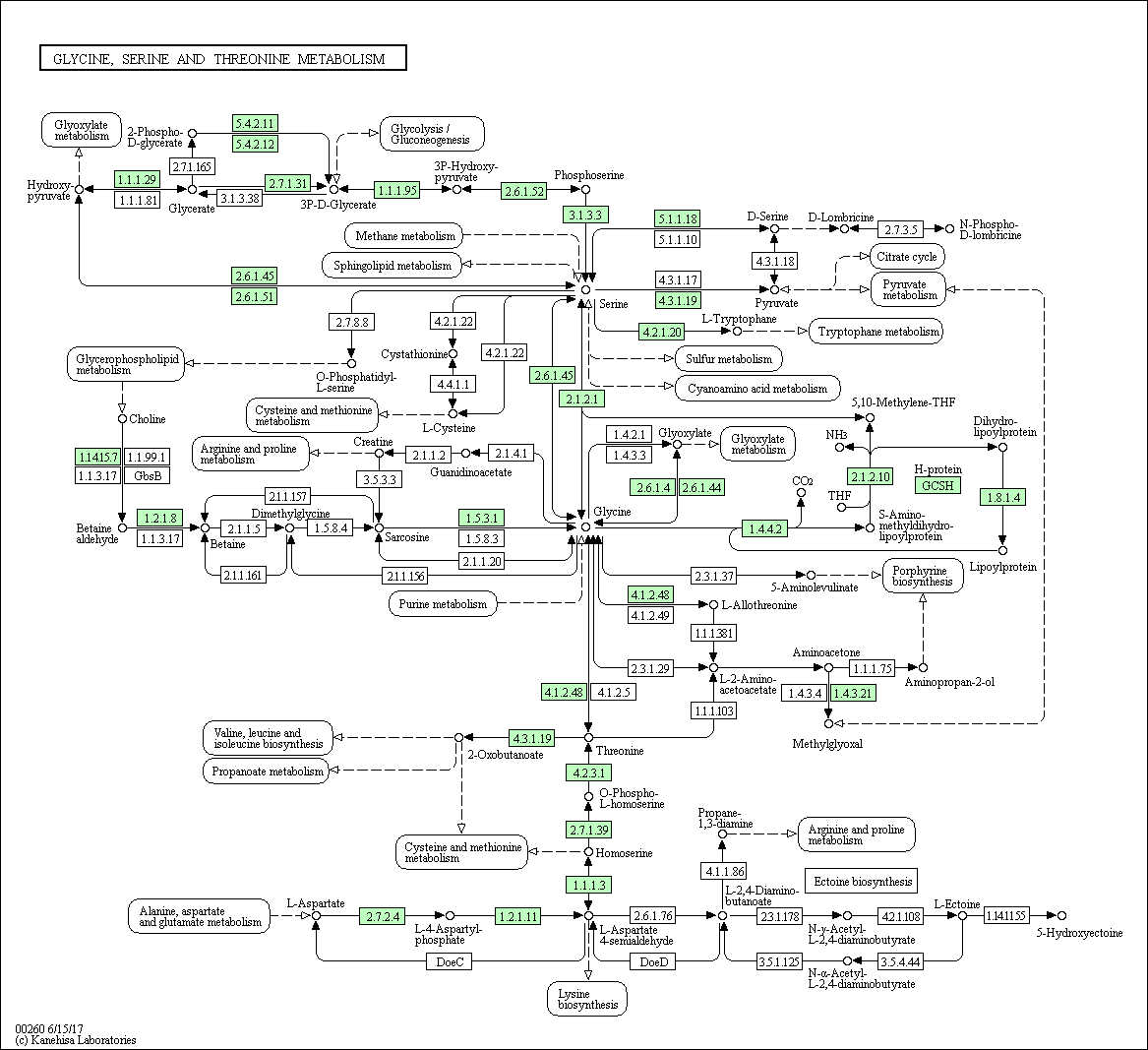

Supplement: S1 File — (ZIP) [file pone.0299259.s004.zip › S1 Zip/src/egu00260.png]

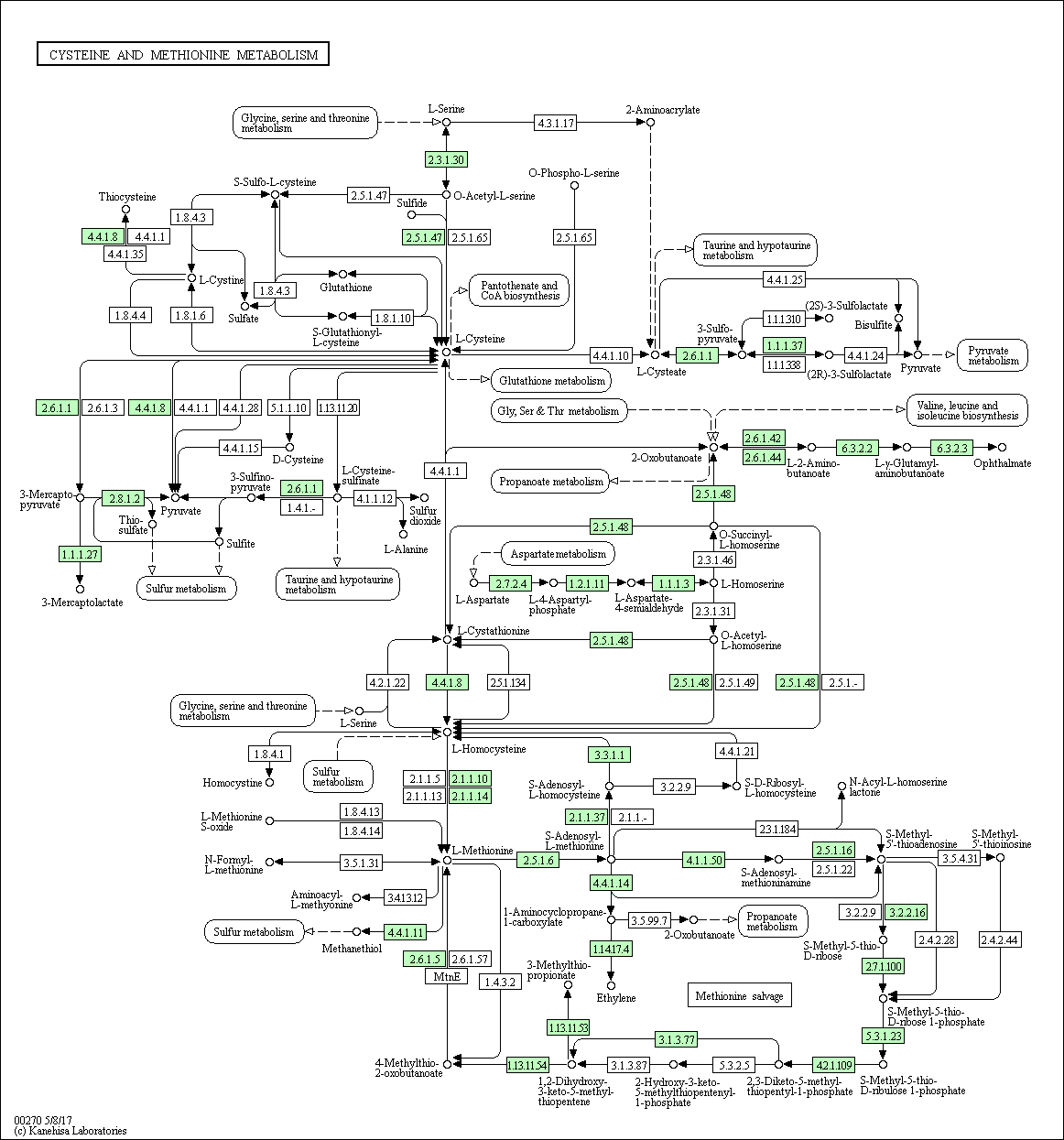

Supplement: S1 File — (ZIP) [file pone.0299259.s004.zip › S1 Zip/src/egu00270.png]

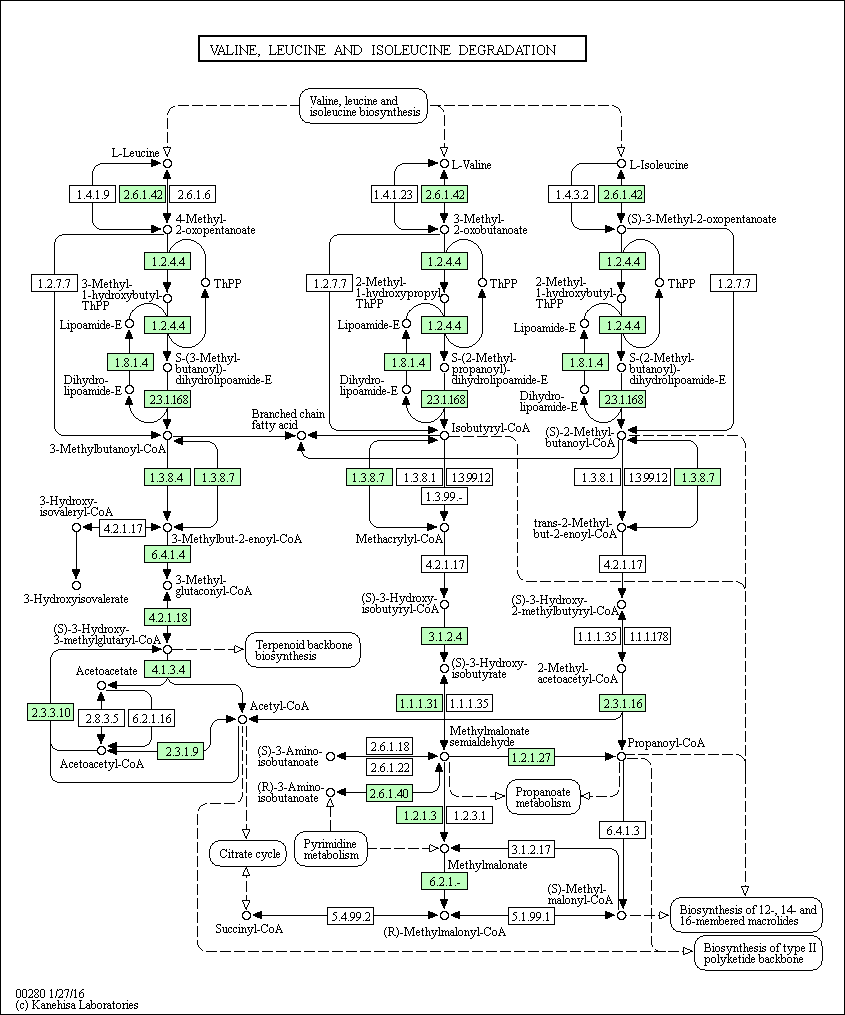

Supplement: S1 File — (ZIP) [file pone.0299259.s004.zip › S1 Zip/src/egu00280.png]

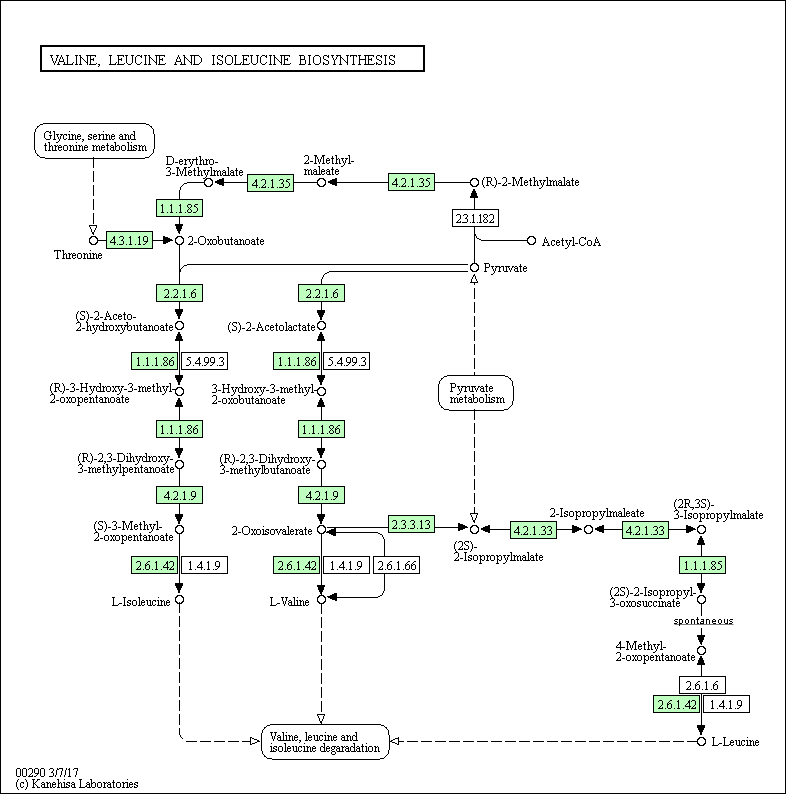

Supplement: S1 File — (ZIP) [file pone.0299259.s004.zip › S1 Zip/src/egu00290.png]

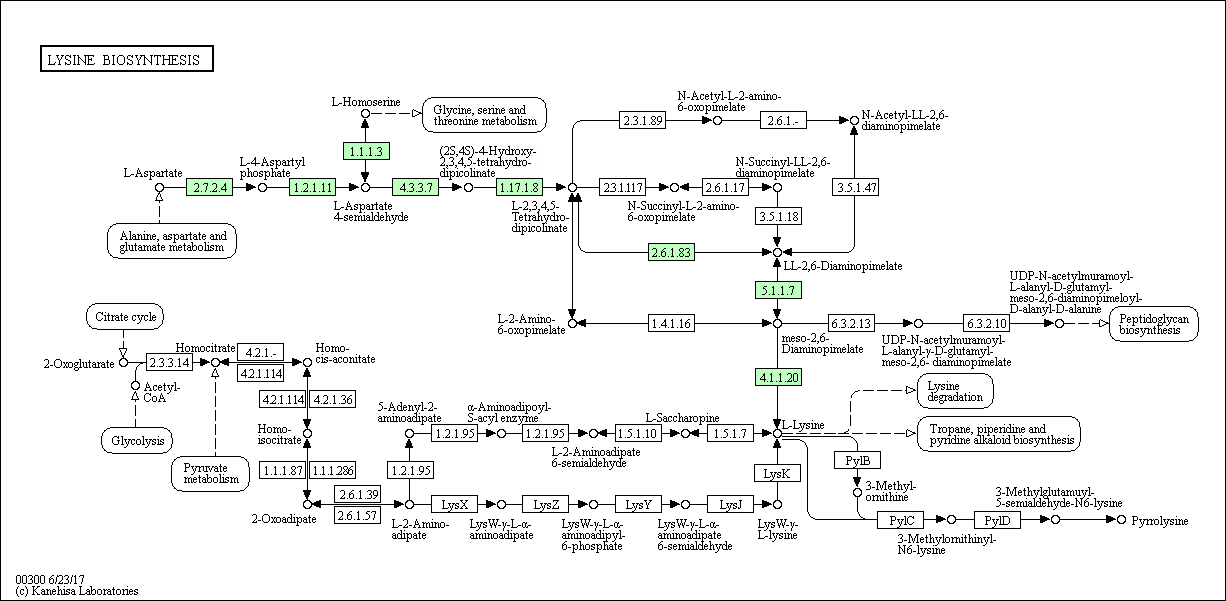

Supplement: S1 File — (ZIP) [file pone.0299259.s004.zip › S1 Zip/src/egu00300.png]

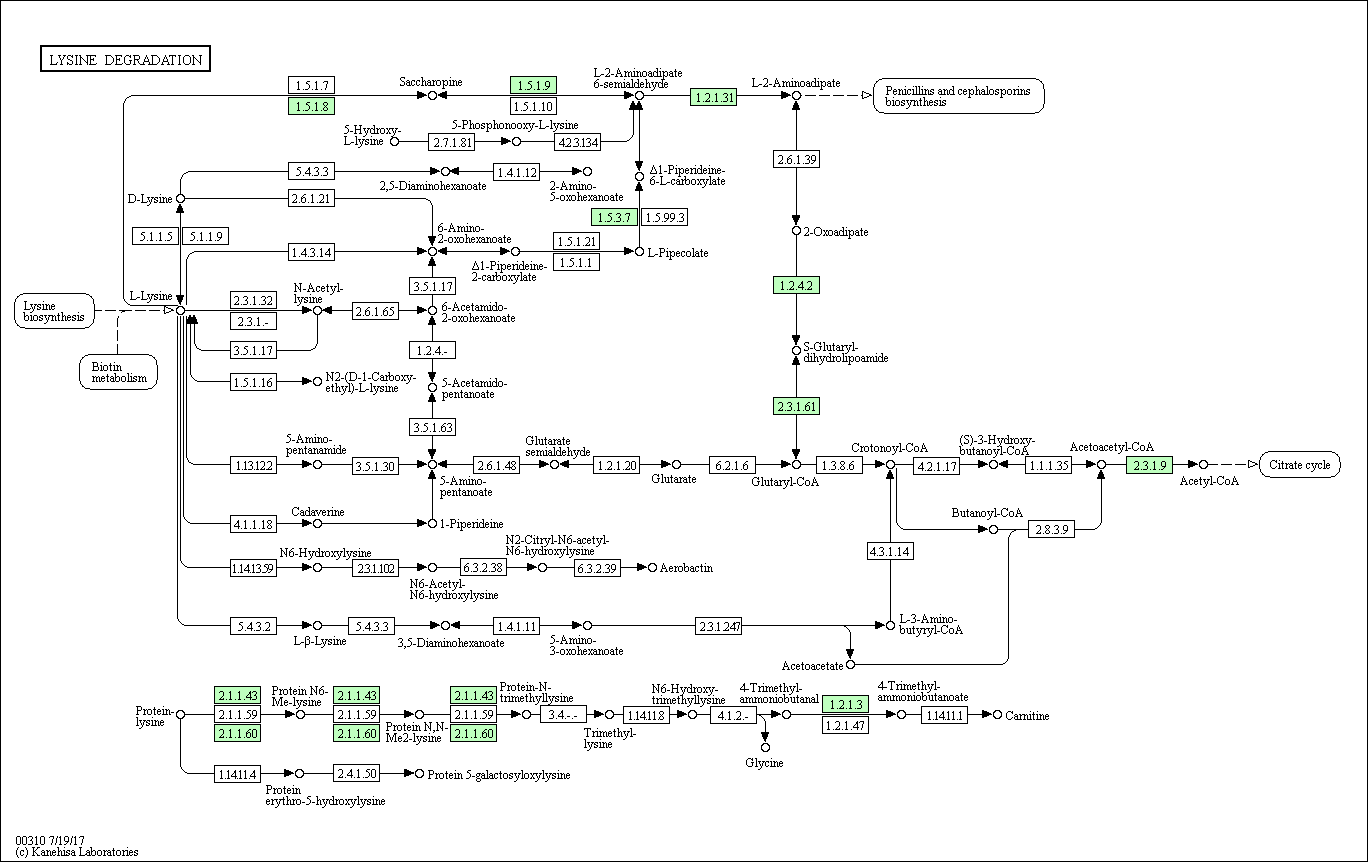

Supplement: S1 File — (ZIP) [file pone.0299259.s004.zip › S1 Zip/src/egu00310.png]

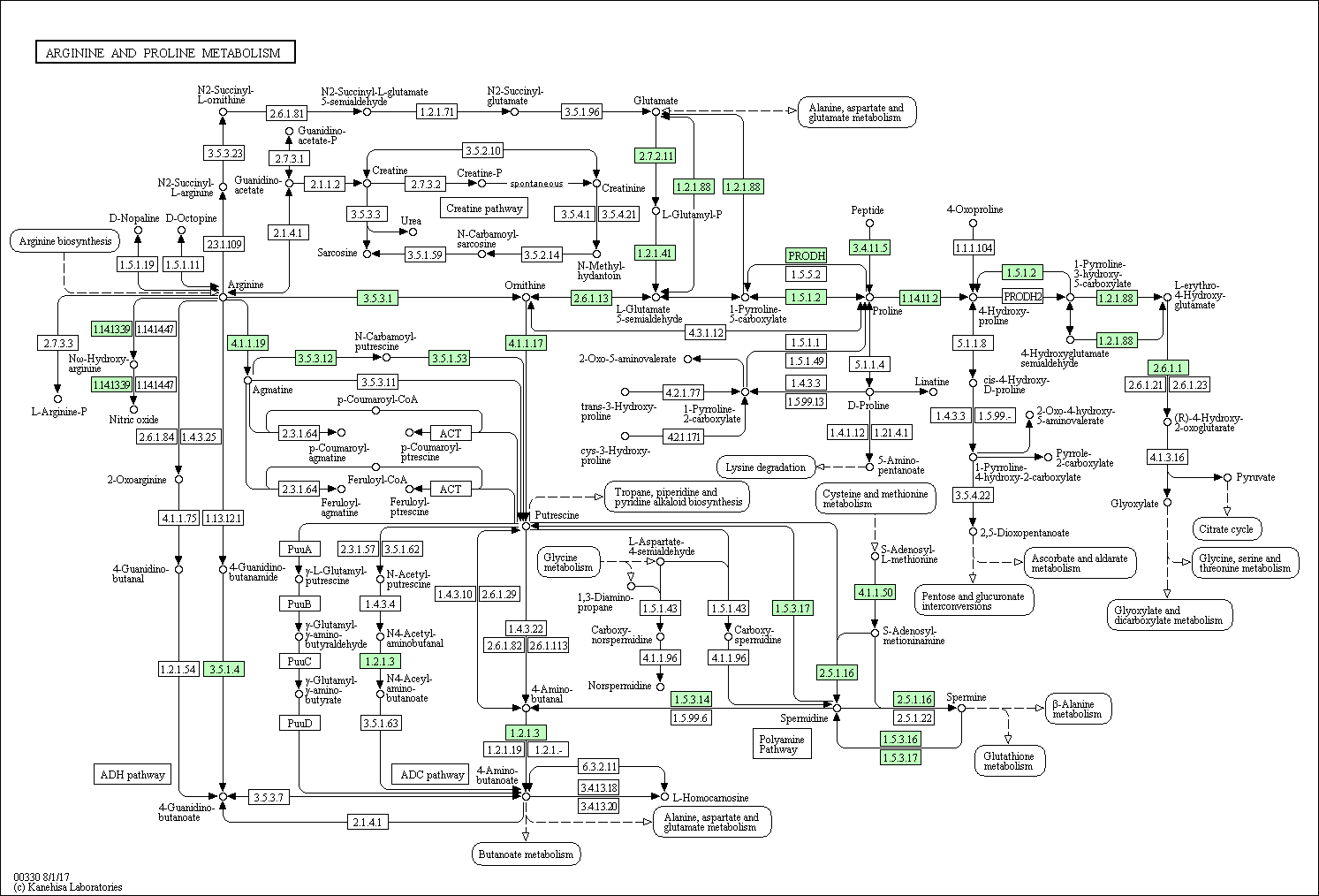

Supplement: S1 File — (ZIP) [file pone.0299259.s004.zip › S1 Zip/src/egu00330.png]

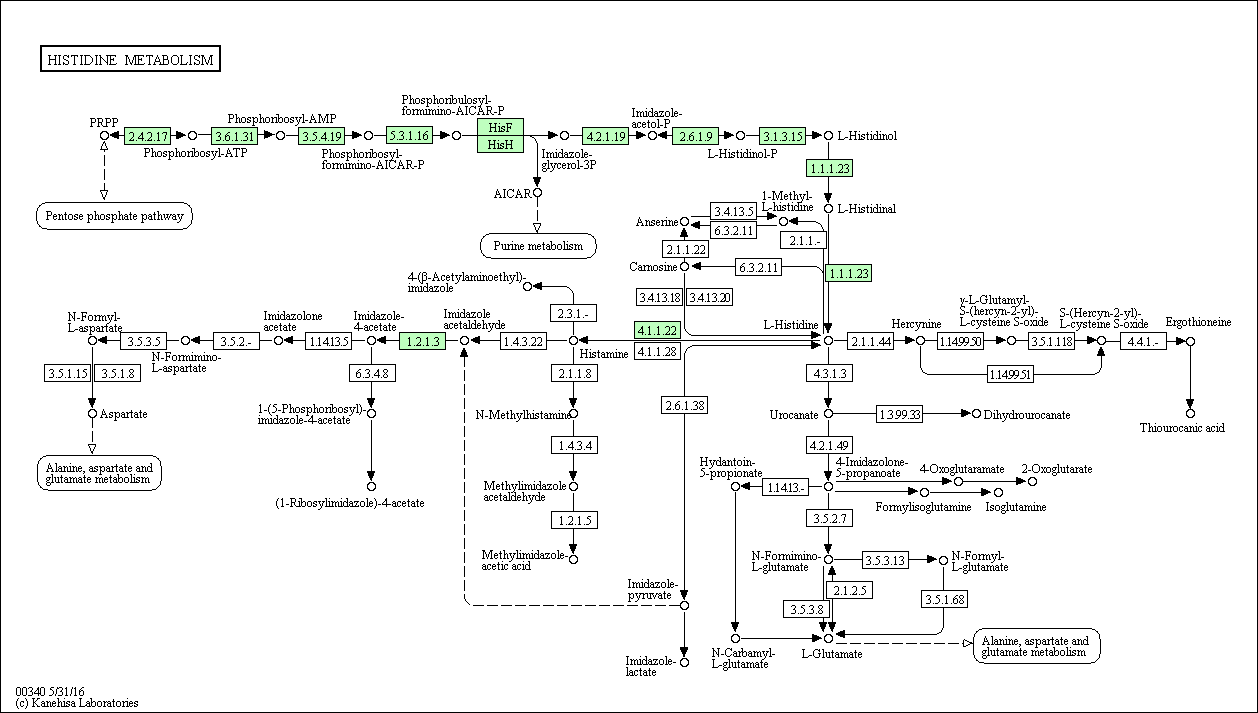

Supplement: S1 File — (ZIP) [file pone.0299259.s004.zip › S1 Zip/src/egu00340.png]

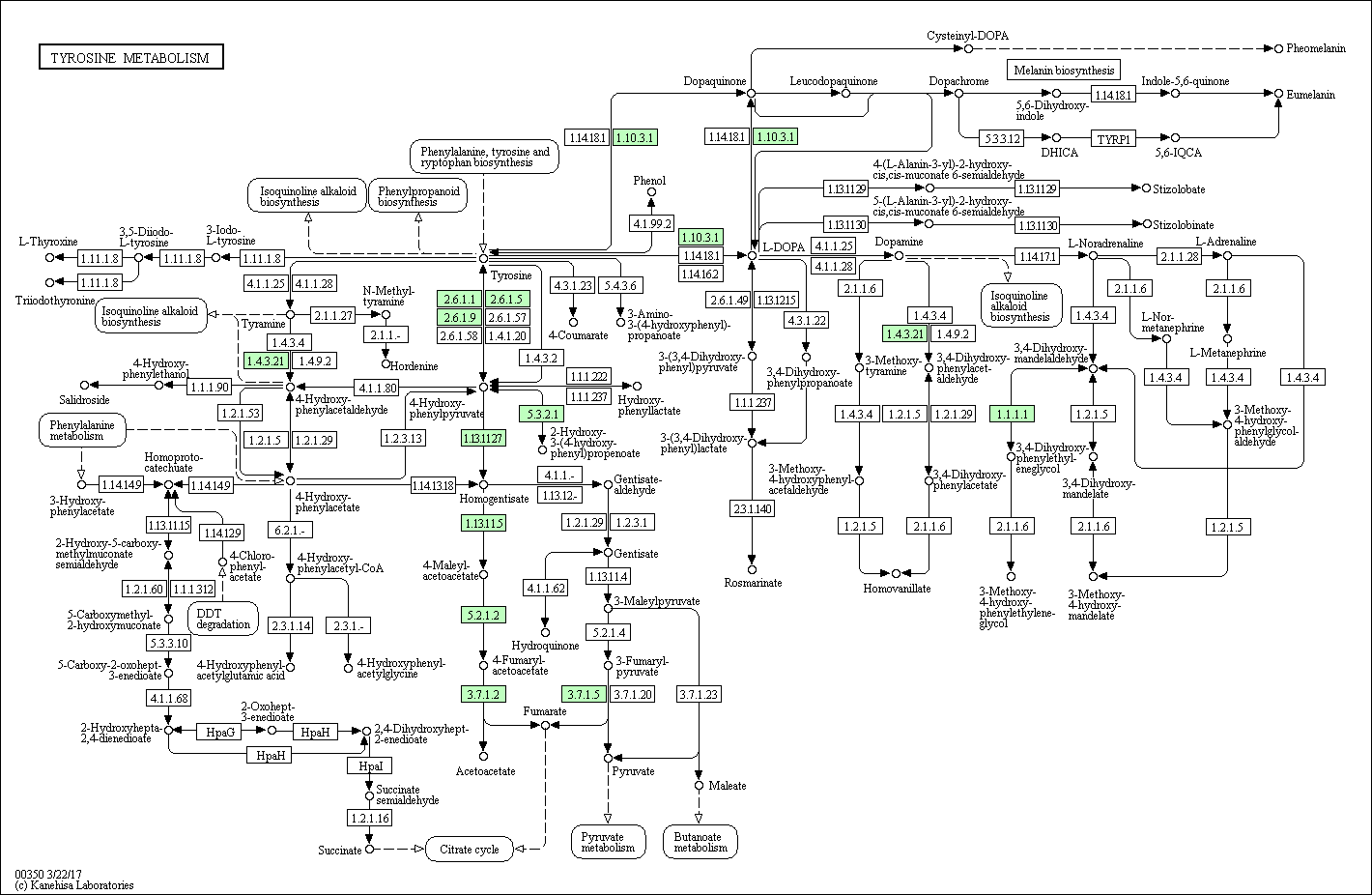

Supplement: S1 File — (ZIP) [file pone.0299259.s004.zip › S1 Zip/src/egu00350.png]

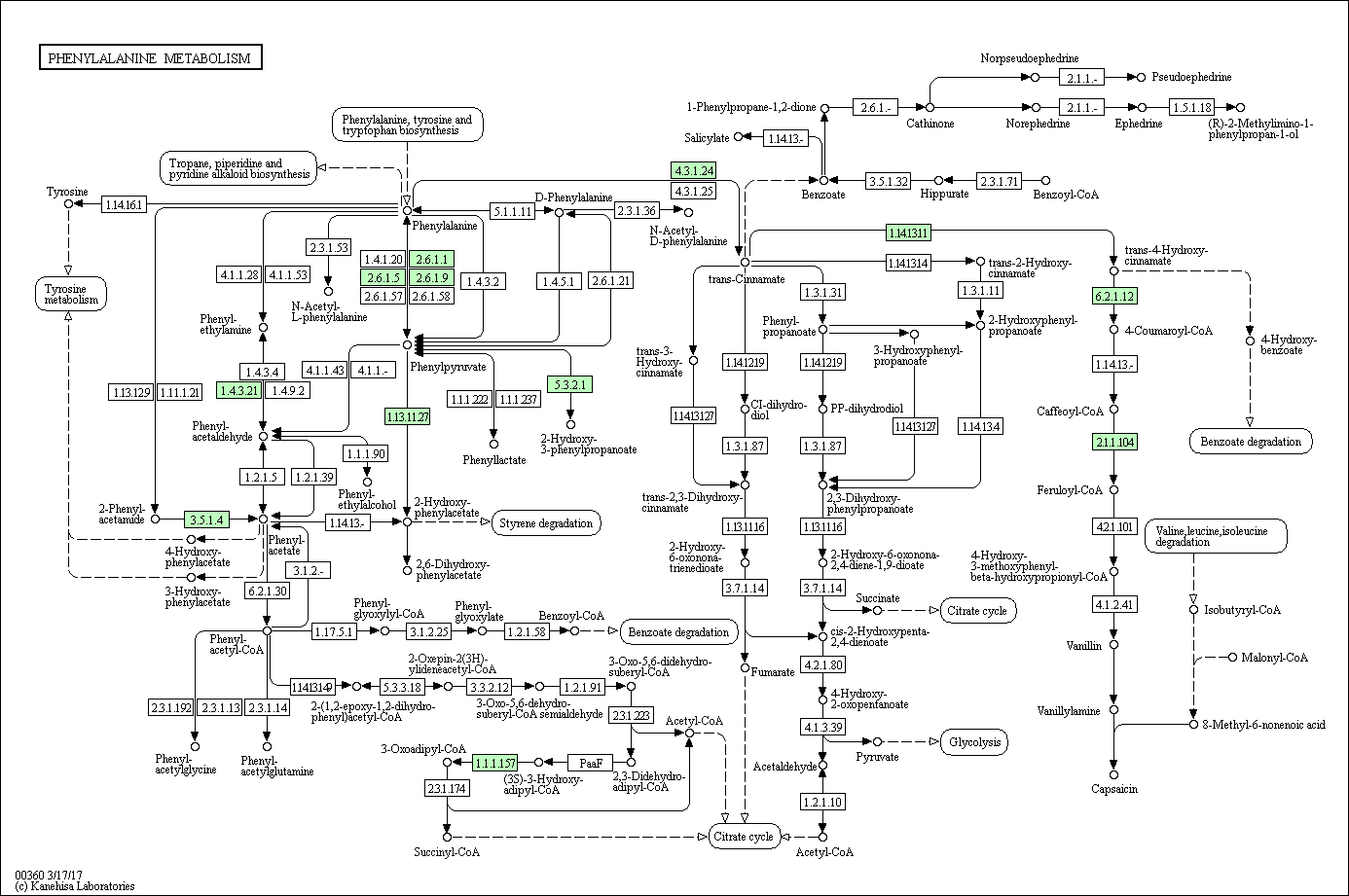

Supplement: S1 File — (ZIP) [file pone.0299259.s004.zip › S1 Zip/src/egu00360.png]

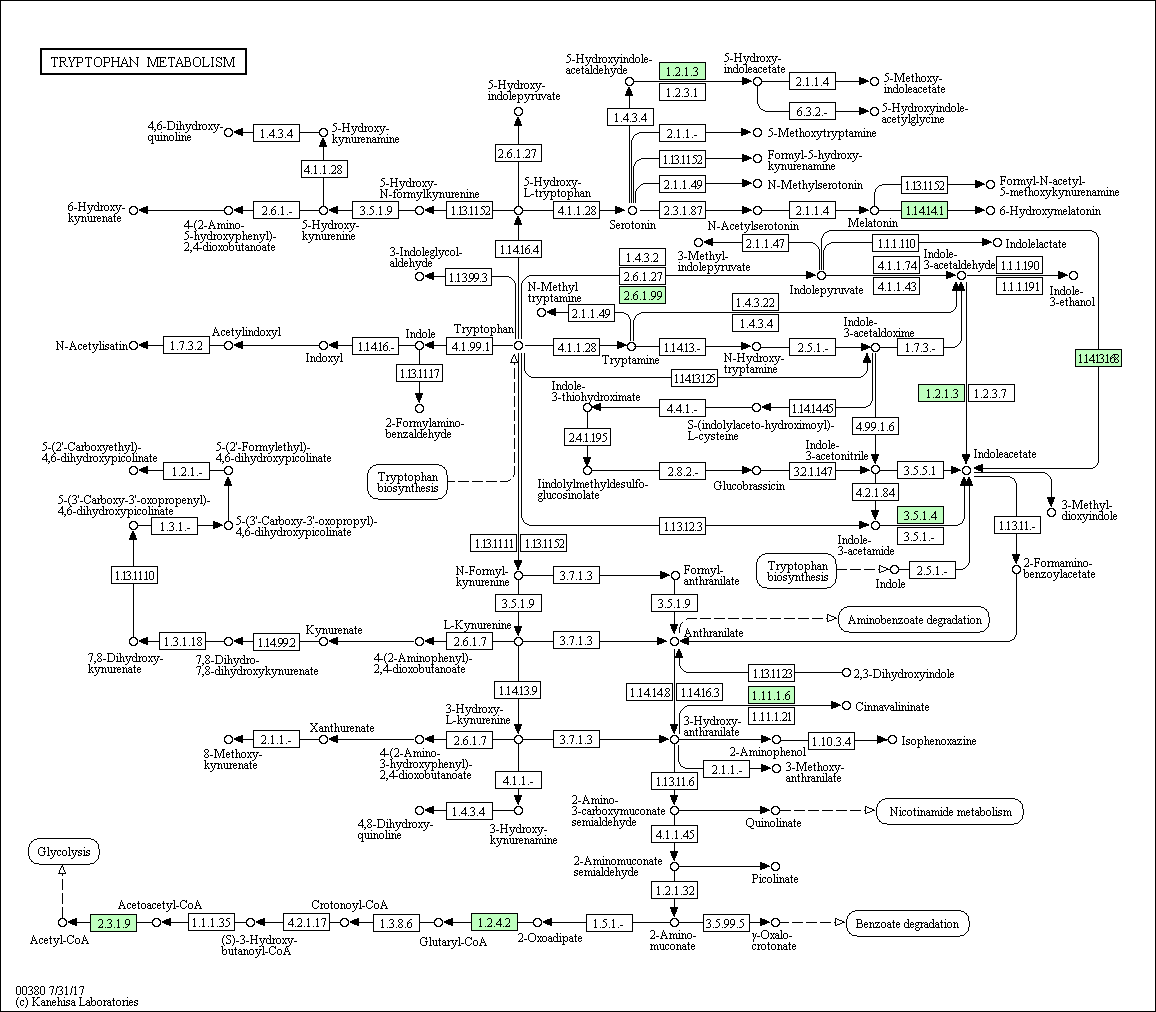

Supplement: S1 File — (ZIP) [file pone.0299259.s004.zip › S1 Zip/src/egu00380.png]

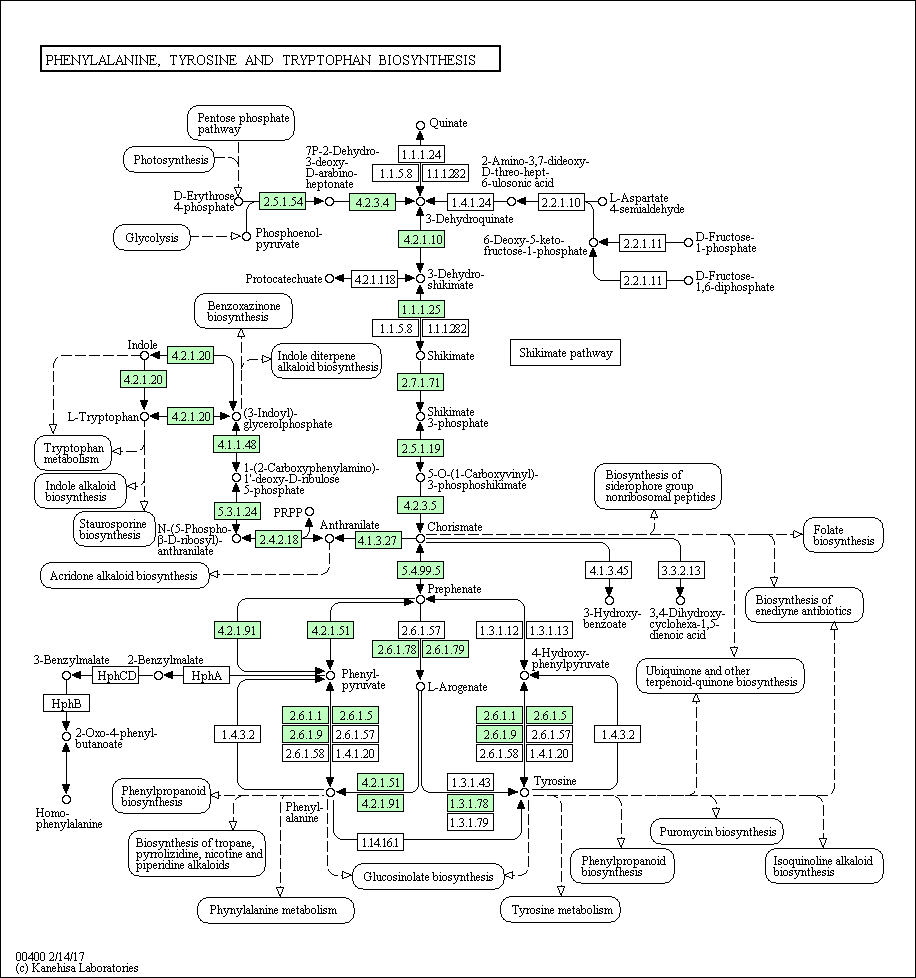

Supplement: S1 File — (ZIP) [file pone.0299259.s004.zip › S1 Zip/src/egu00400.png]

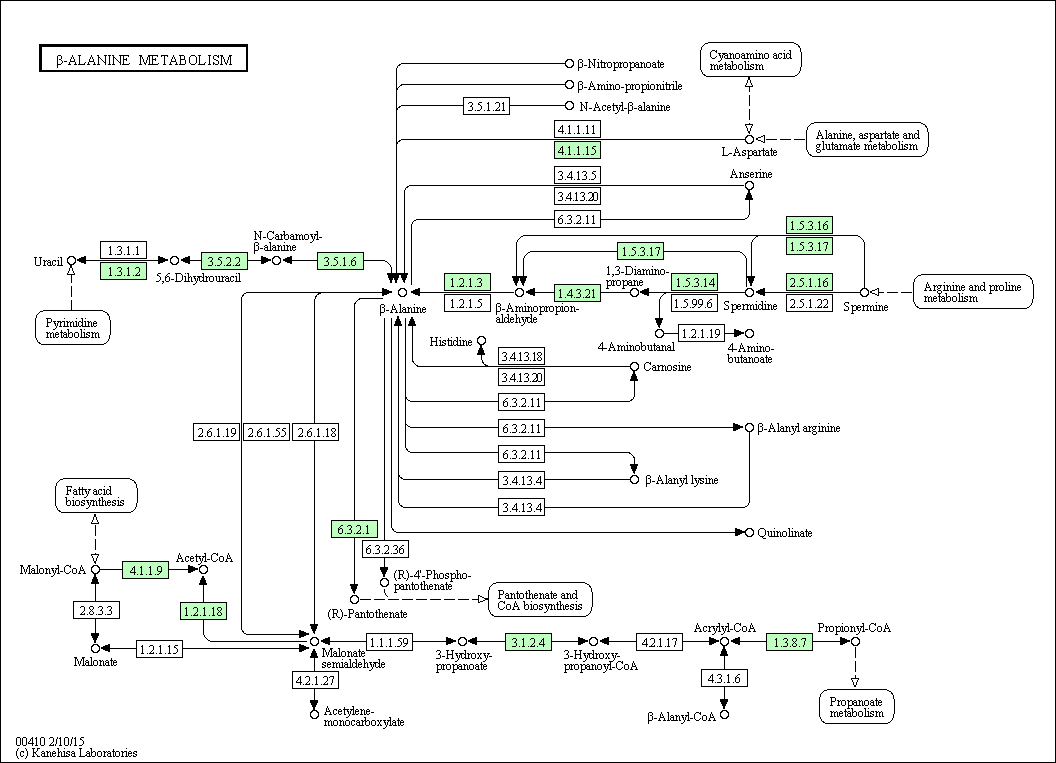

Supplement: S1 File — (ZIP) [file pone.0299259.s004.zip › S1 Zip/src/egu00410.png]

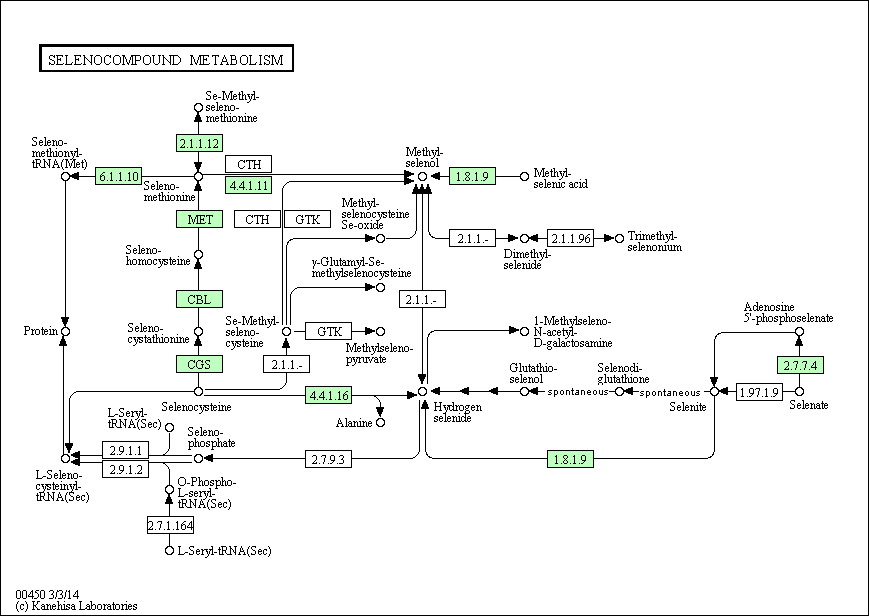

Supplement: S1 File — (ZIP) [file pone.0299259.s004.zip › S1 Zip/src/egu00450.png]

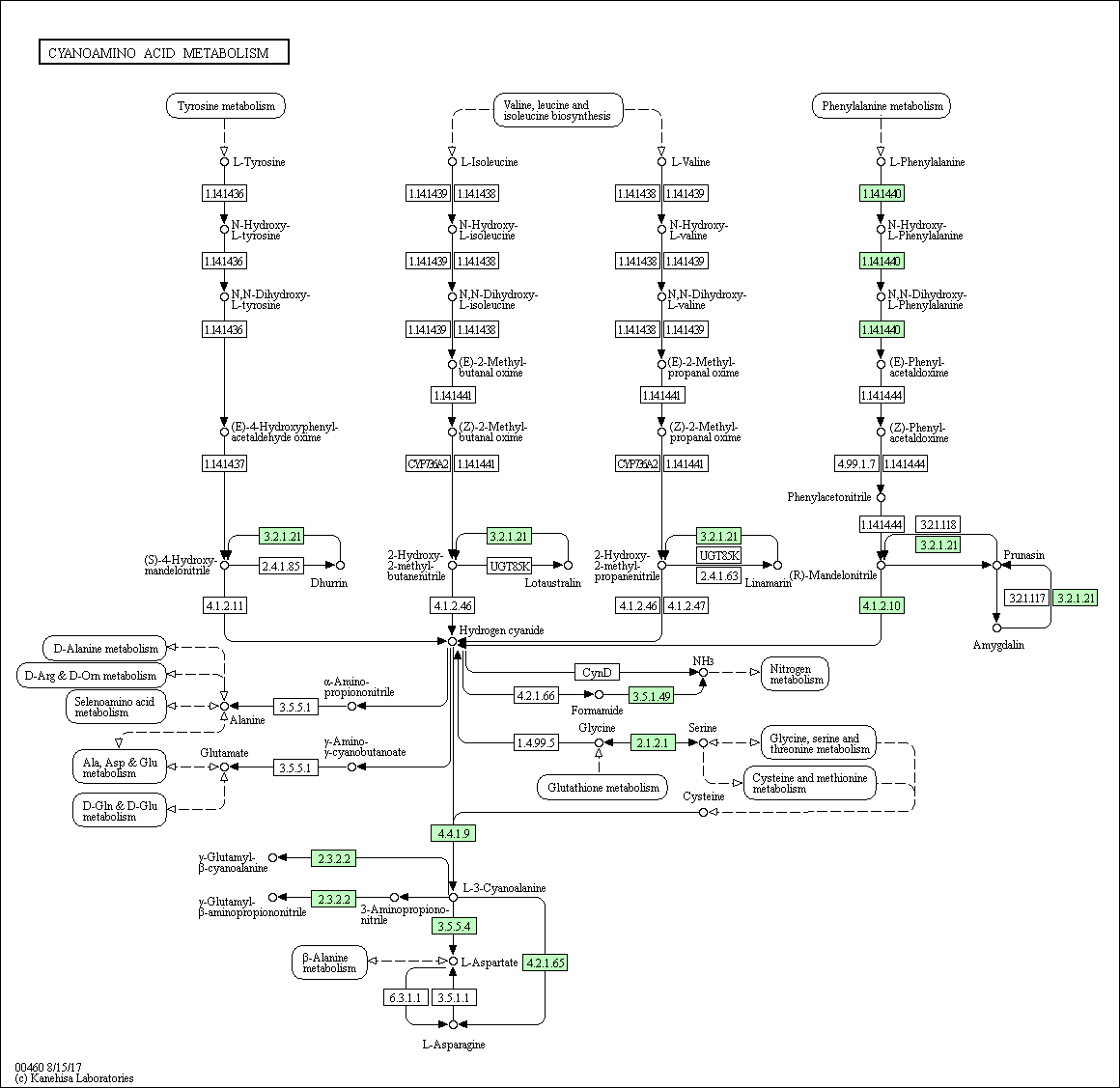

Supplement: S1 File — (ZIP) [file pone.0299259.s004.zip › S1 Zip/src/egu00460.png]

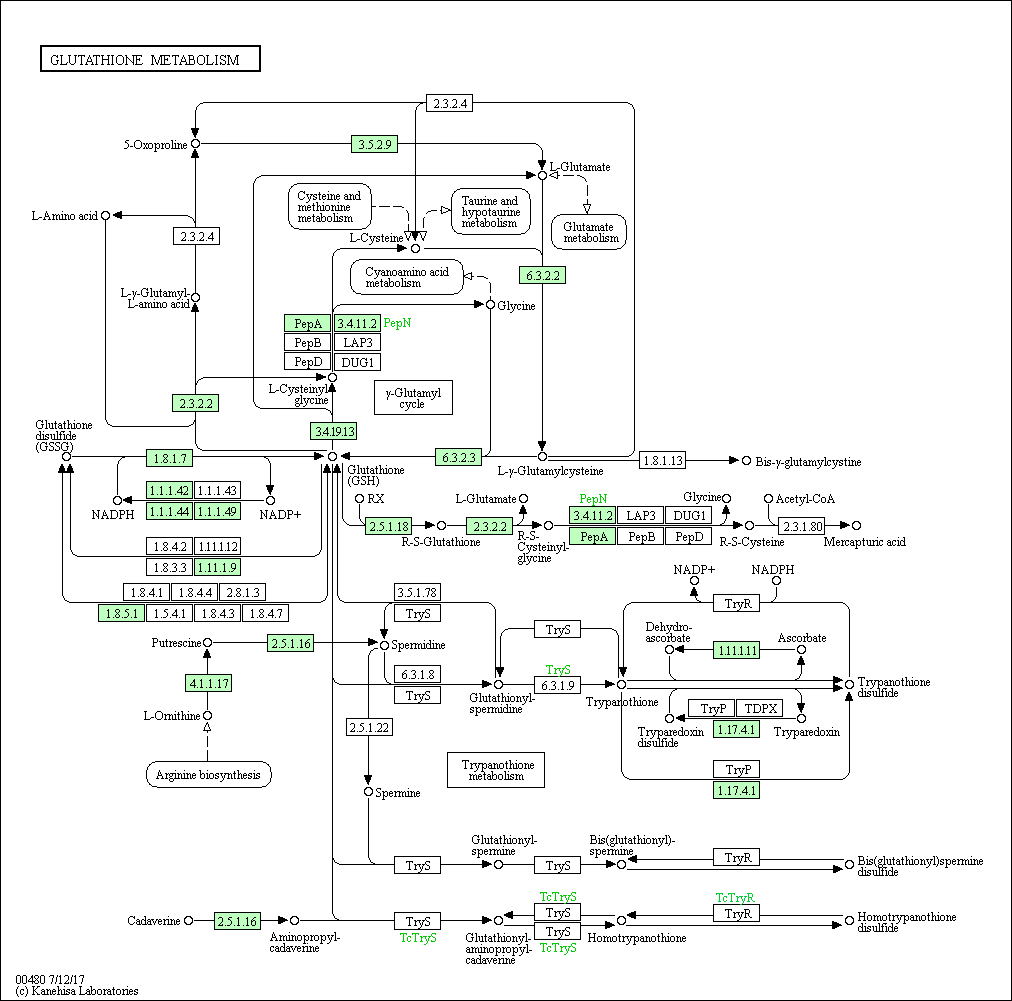

Supplement: S1 File — (ZIP) [file pone.0299259.s004.zip › S1 Zip/src/egu00480.png]

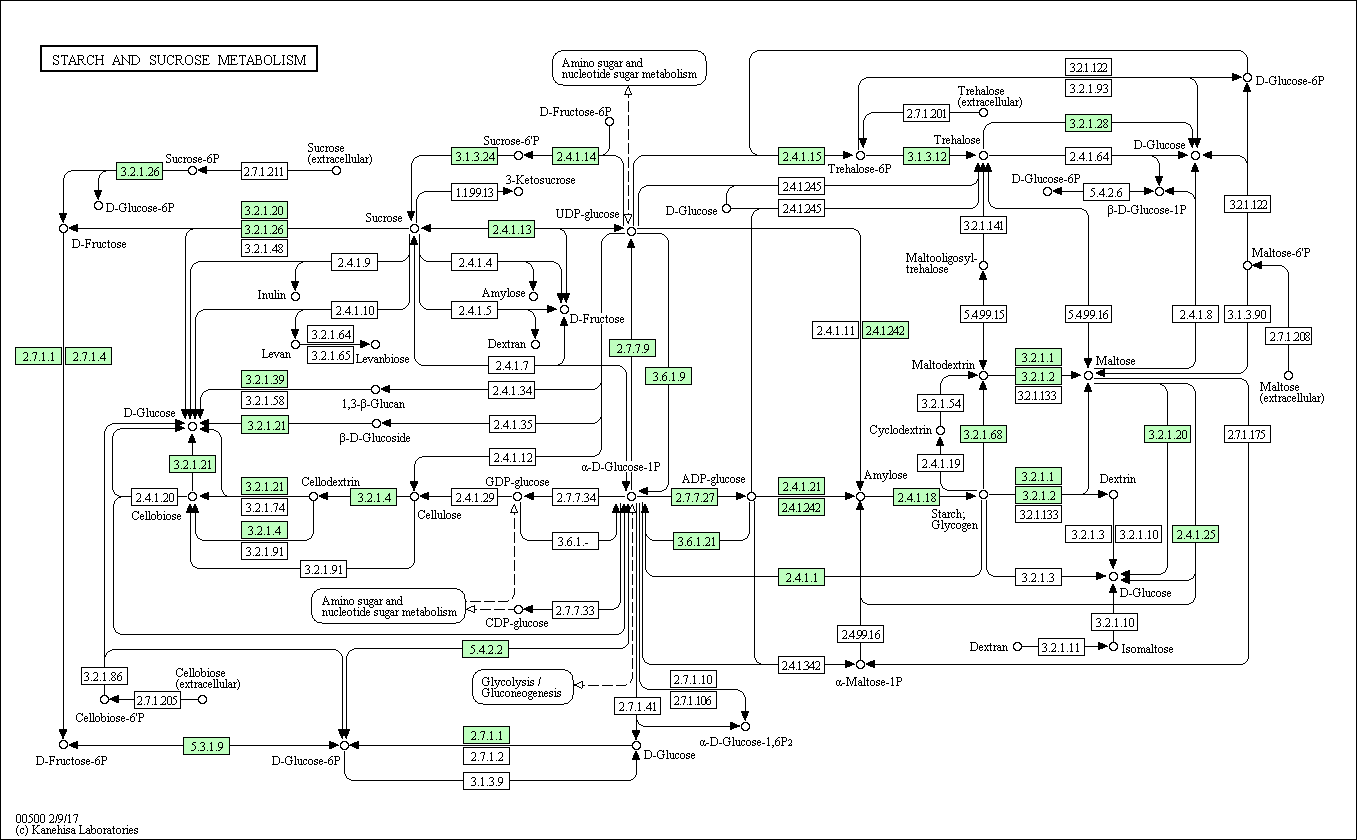

Supplement: S1 File — (ZIP) [file pone.0299259.s004.zip › S1 Zip/src/egu00500.png]

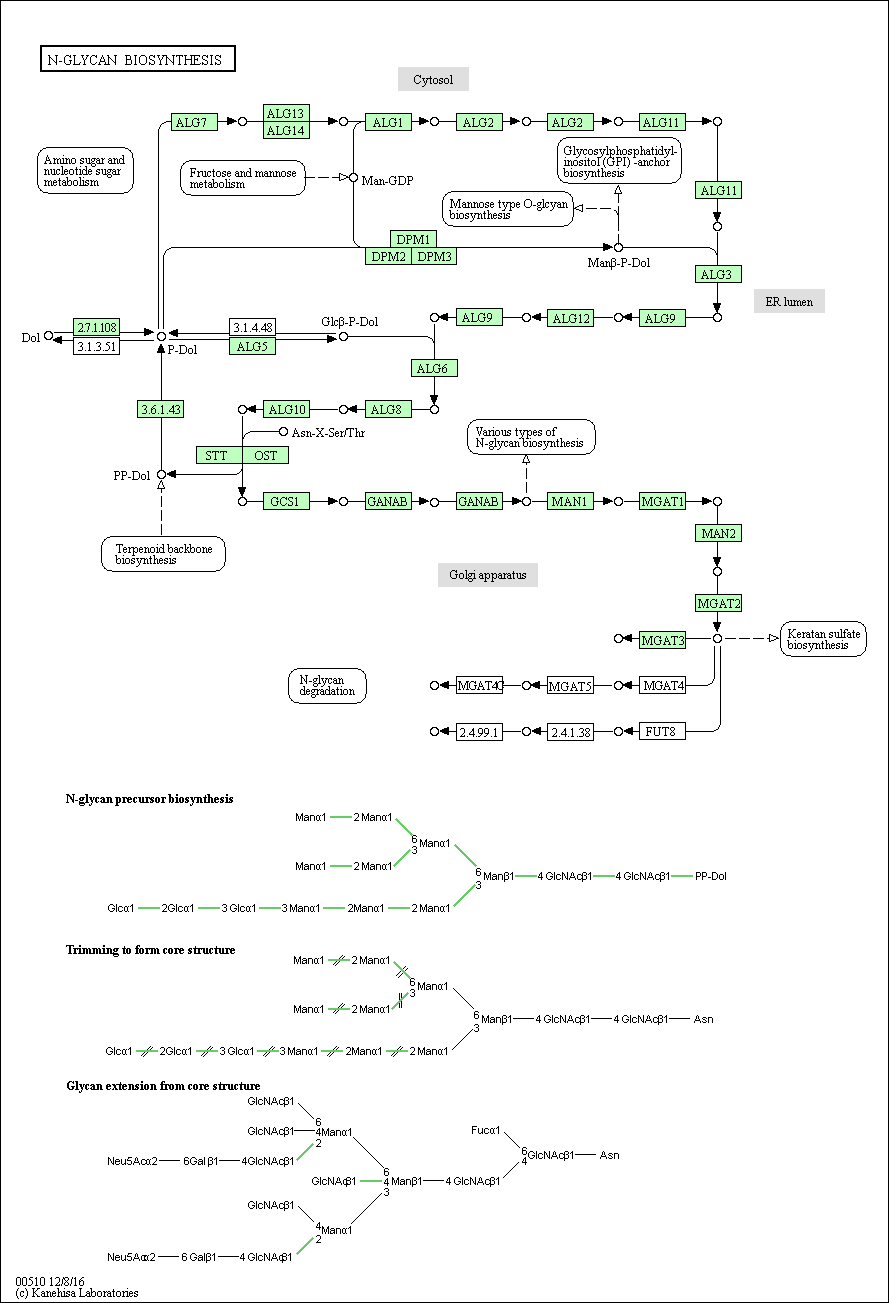

Supplement: S1 File — (ZIP) [file pone.0299259.s004.zip › S1 Zip/src/egu00510.png]

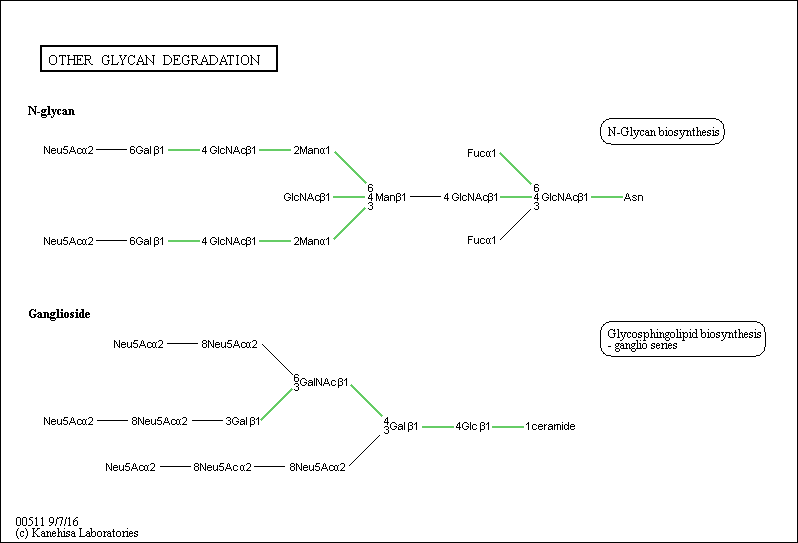

Supplement: S1 File — (ZIP) [file pone.0299259.s004.zip › S1 Zip/src/egu00511.png]

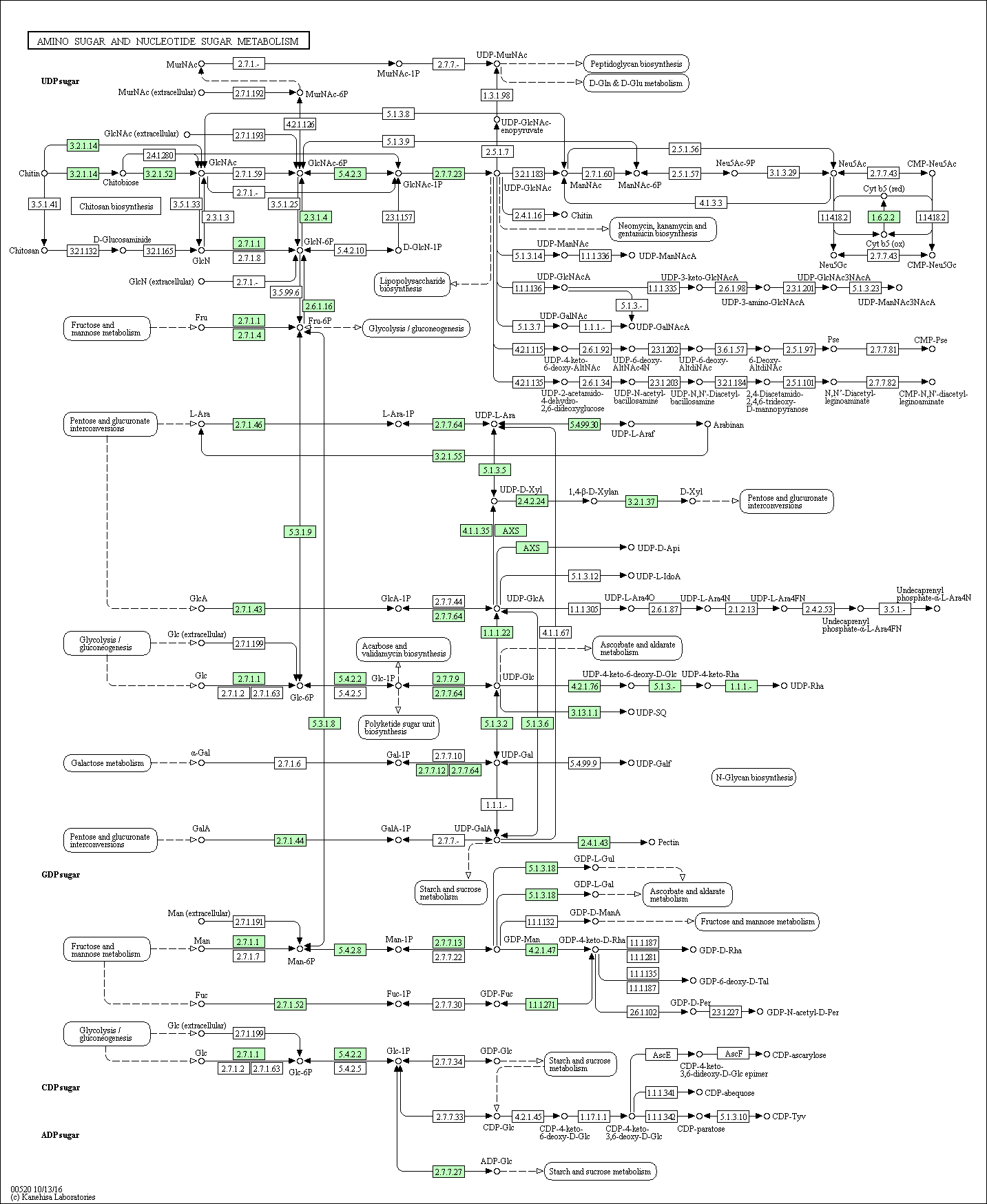

Supplement: S1 File — (ZIP) [file pone.0299259.s004.zip › S1 Zip/src/egu00520.png]

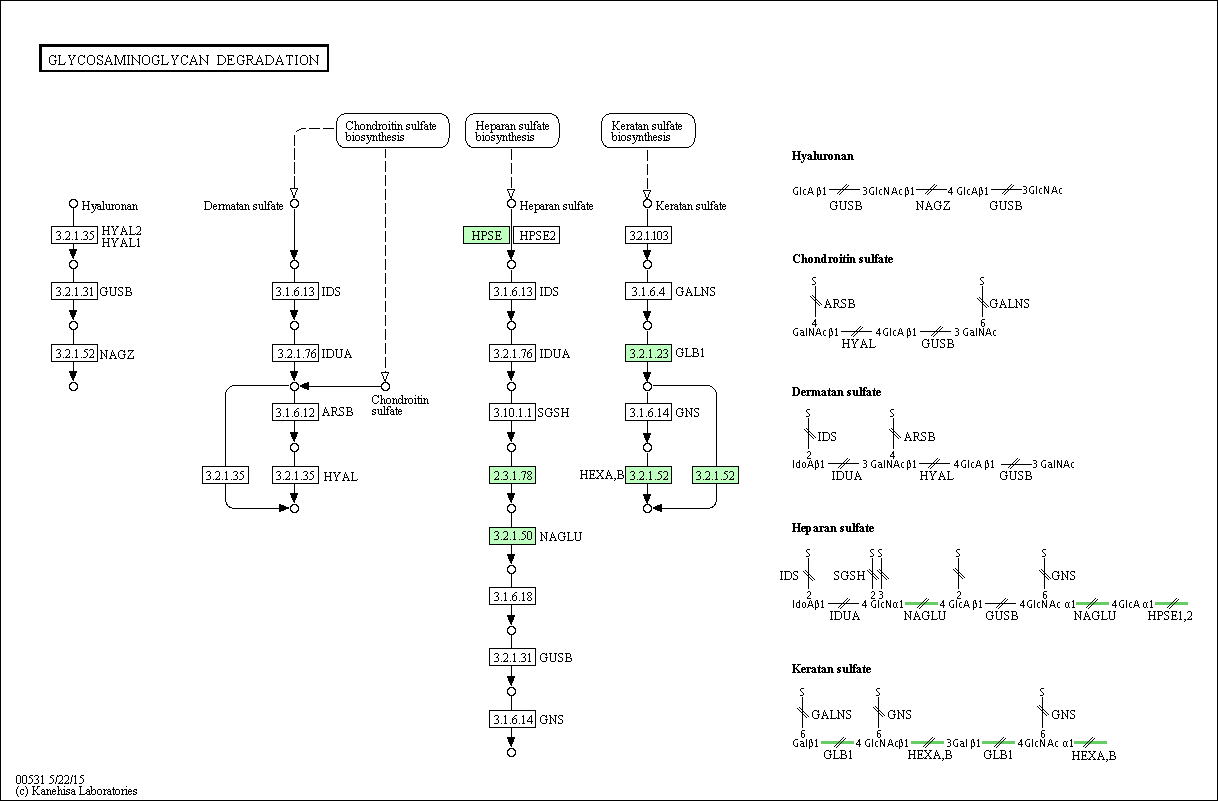

Supplement: S1 File — (ZIP) [file pone.0299259.s004.zip › S1 Zip/src/egu00531.png]

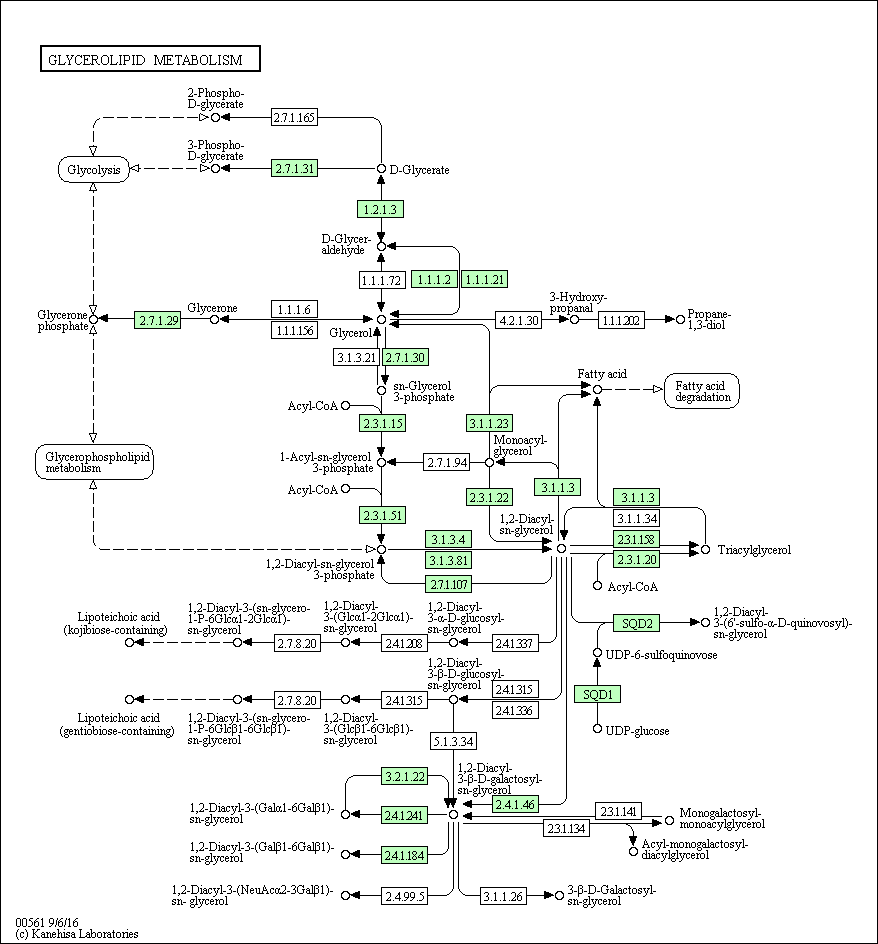

Supplement: S1 File — (ZIP) [file pone.0299259.s004.zip › S1 Zip/src/egu00561.png]

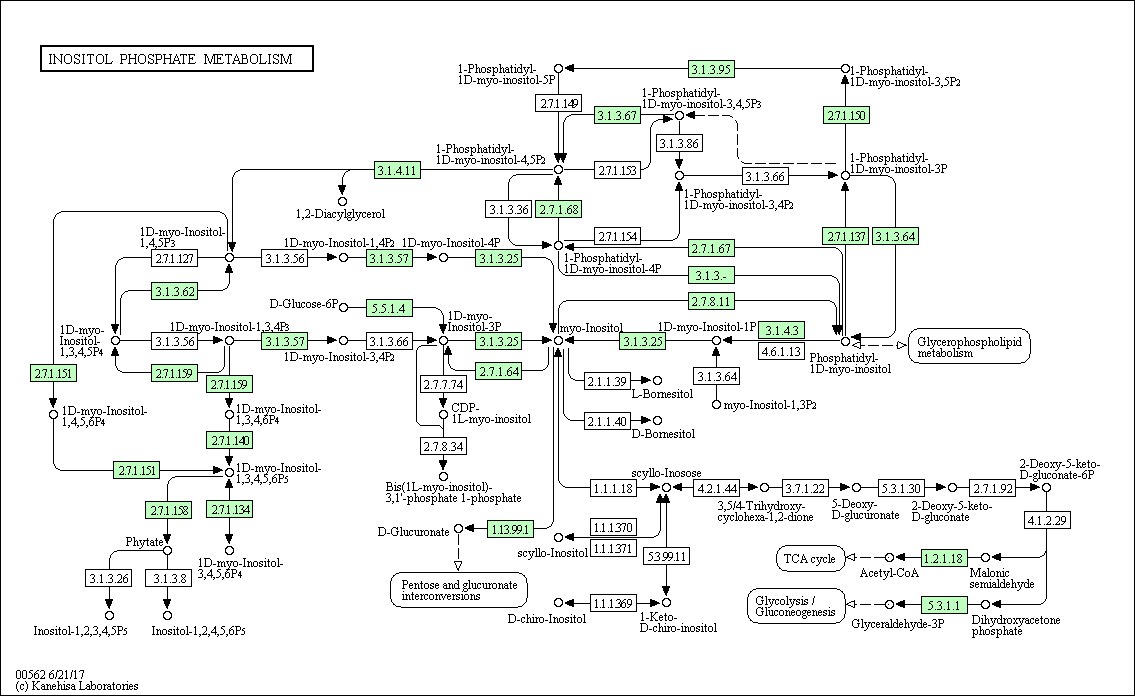

Supplement: S1 File — (ZIP) [file pone.0299259.s004.zip › S1 Zip/src/egu00562.png]

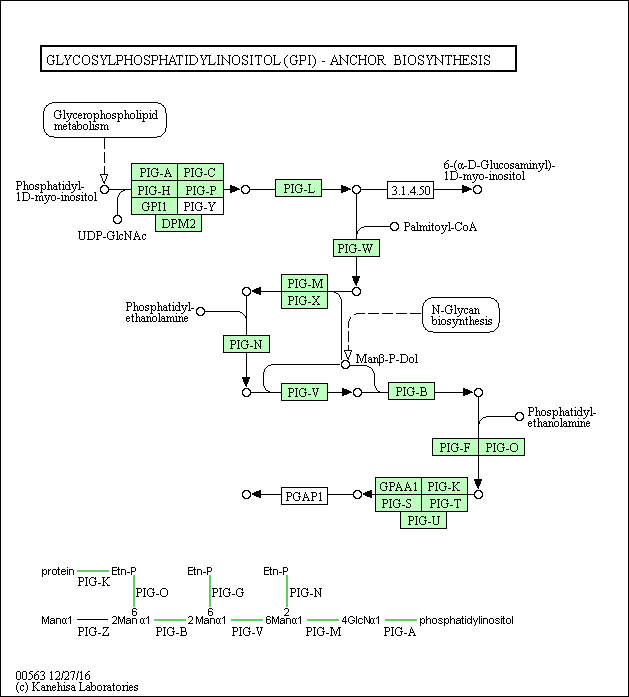

Supplement: S1 File — (ZIP) [file pone.0299259.s004.zip › S1 Zip/src/egu00563.png]

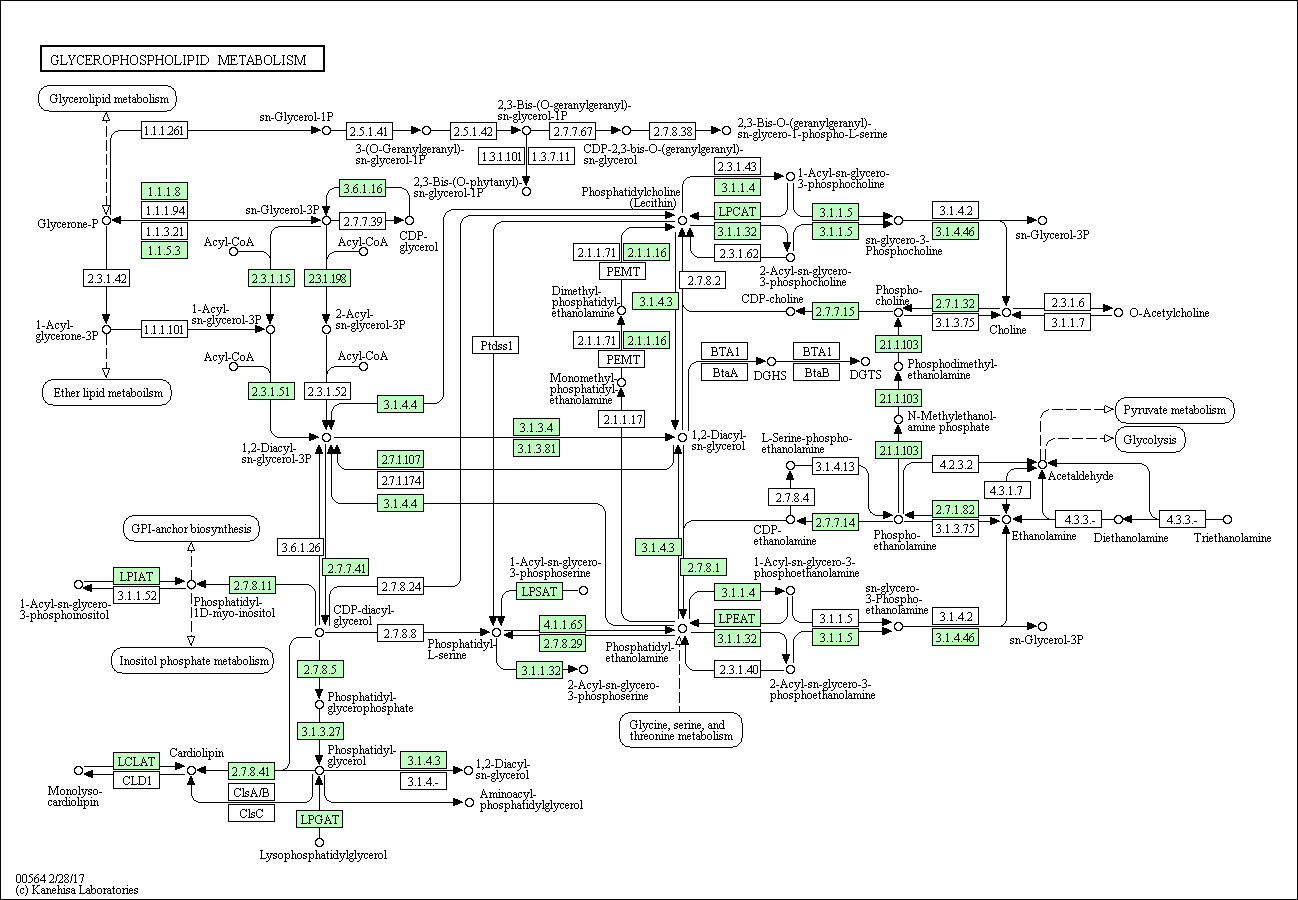

Supplement: S1 File — (ZIP) [file pone.0299259.s004.zip › S1 Zip/src/egu00564.png]

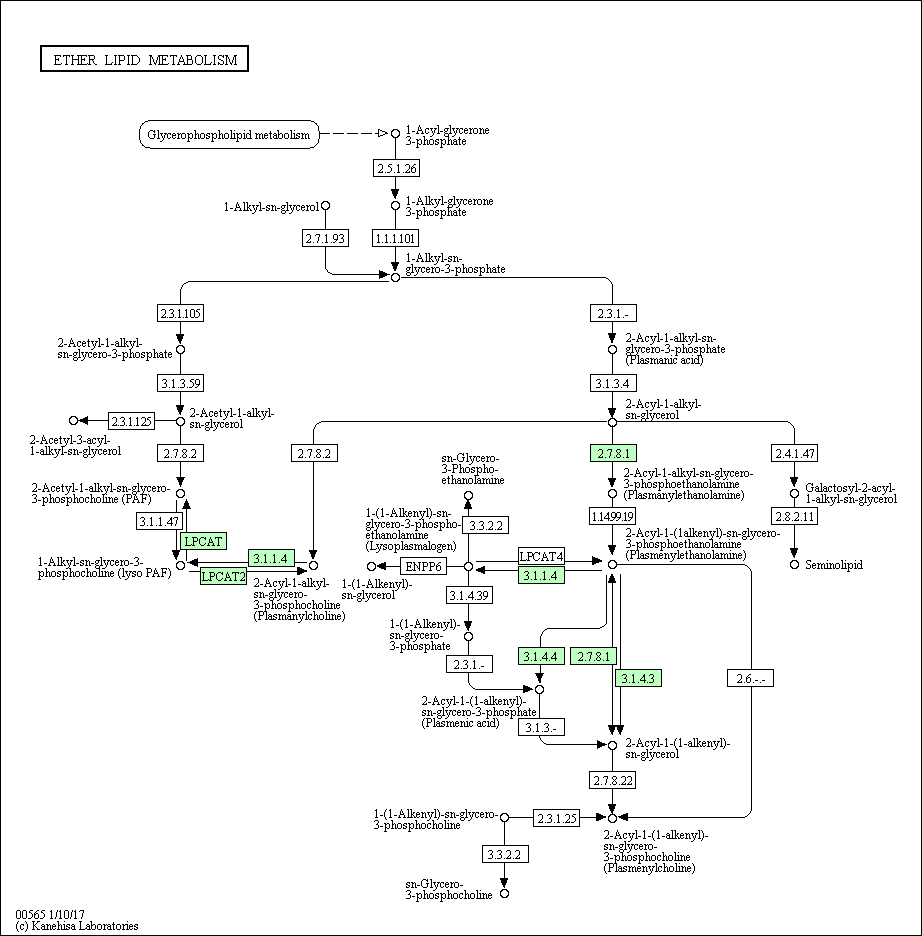

Supplement: S1 File — (ZIP) [file pone.0299259.s004.zip › S1 Zip/src/egu00565.png]

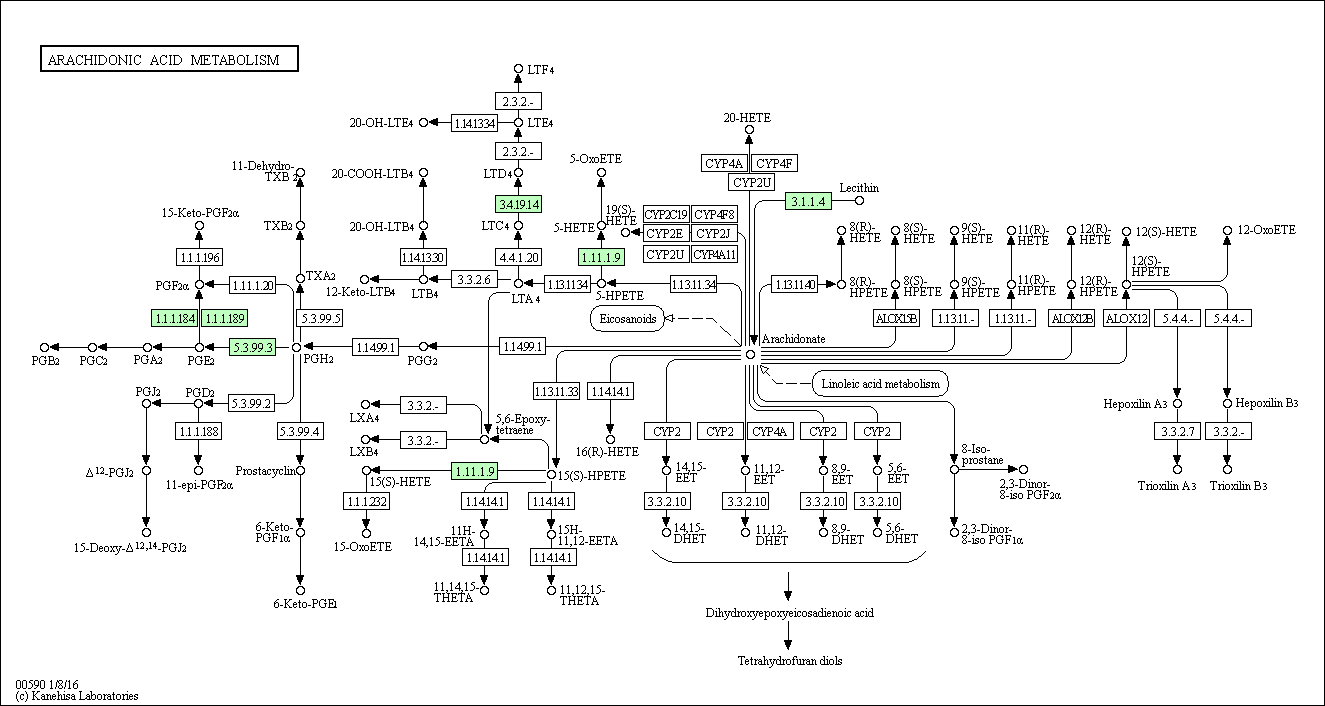

Supplement: S1 File — (ZIP) [file pone.0299259.s004.zip › S1 Zip/src/egu00590.png]

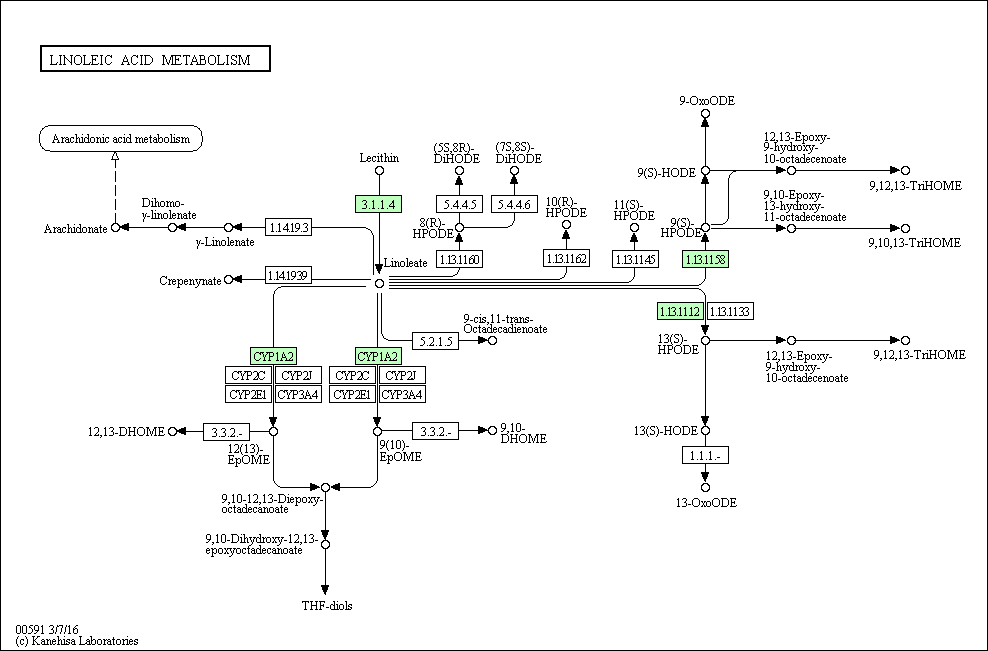

Supplement: S1 File — (ZIP) [file pone.0299259.s004.zip › S1 Zip/src/egu00591.png]

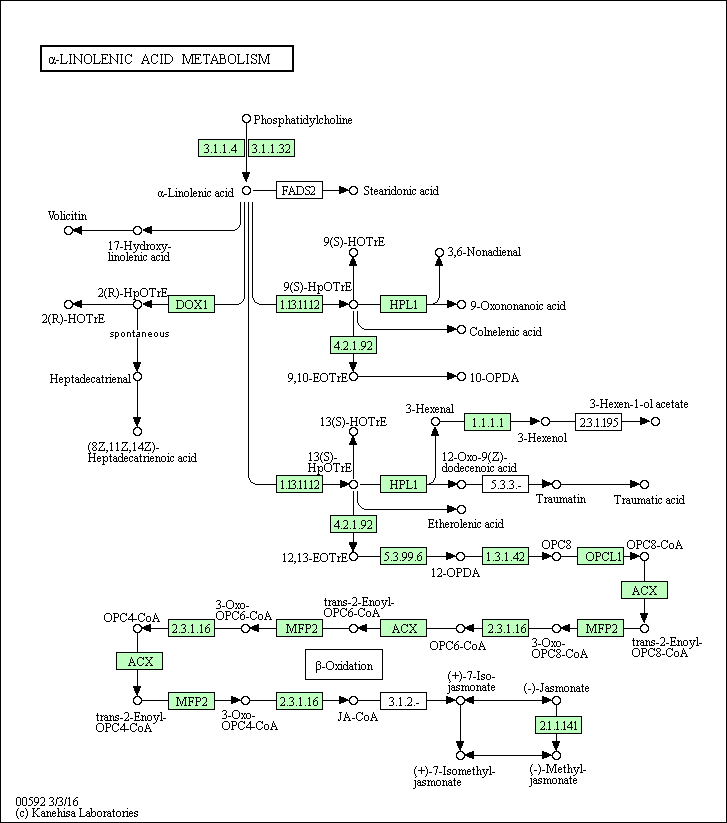

Supplement: S1 File — (ZIP) [file pone.0299259.s004.zip › S1 Zip/src/egu00592.png]

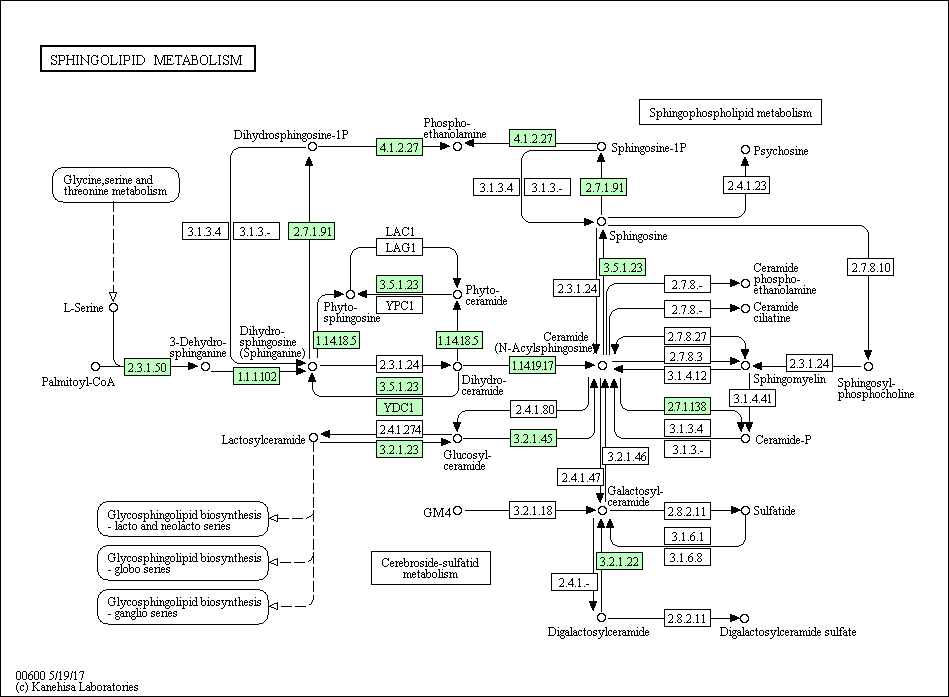

Supplement: S1 File — (ZIP) [file pone.0299259.s004.zip › S1 Zip/src/egu00600.png]

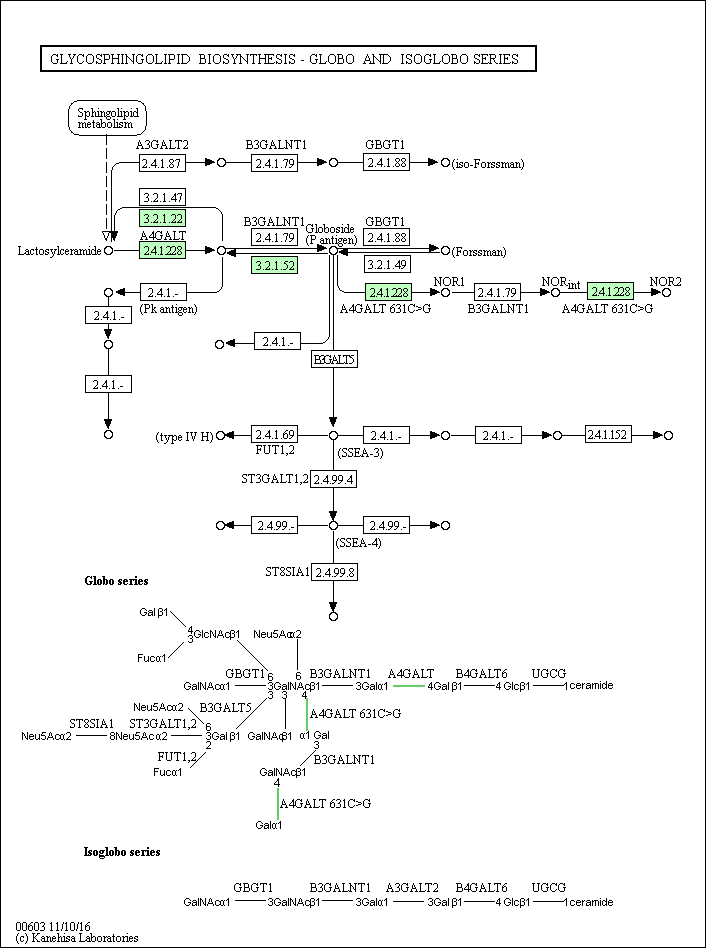

Supplement: S1 File — (ZIP) [file pone.0299259.s004.zip › S1 Zip/src/egu00603.png]

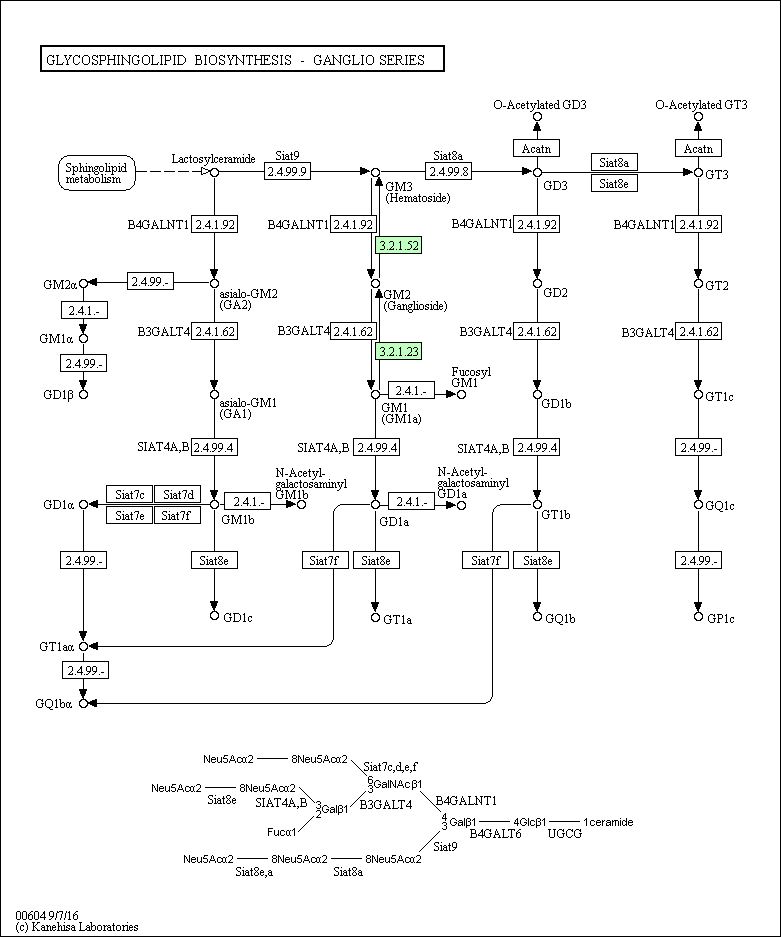

Supplement: S1 File — (ZIP) [file pone.0299259.s004.zip › S1 Zip/src/egu00604.png]

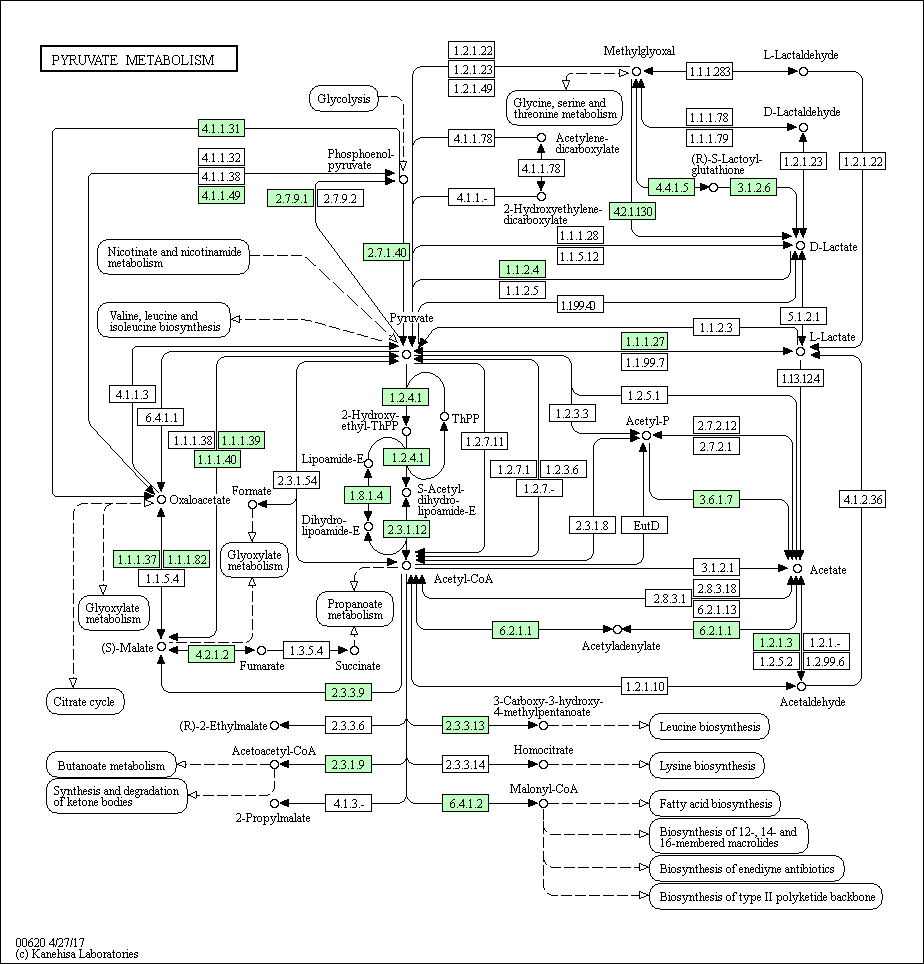

Supplement: S1 File — (ZIP) [file pone.0299259.s004.zip › S1 Zip/src/egu00620.png]

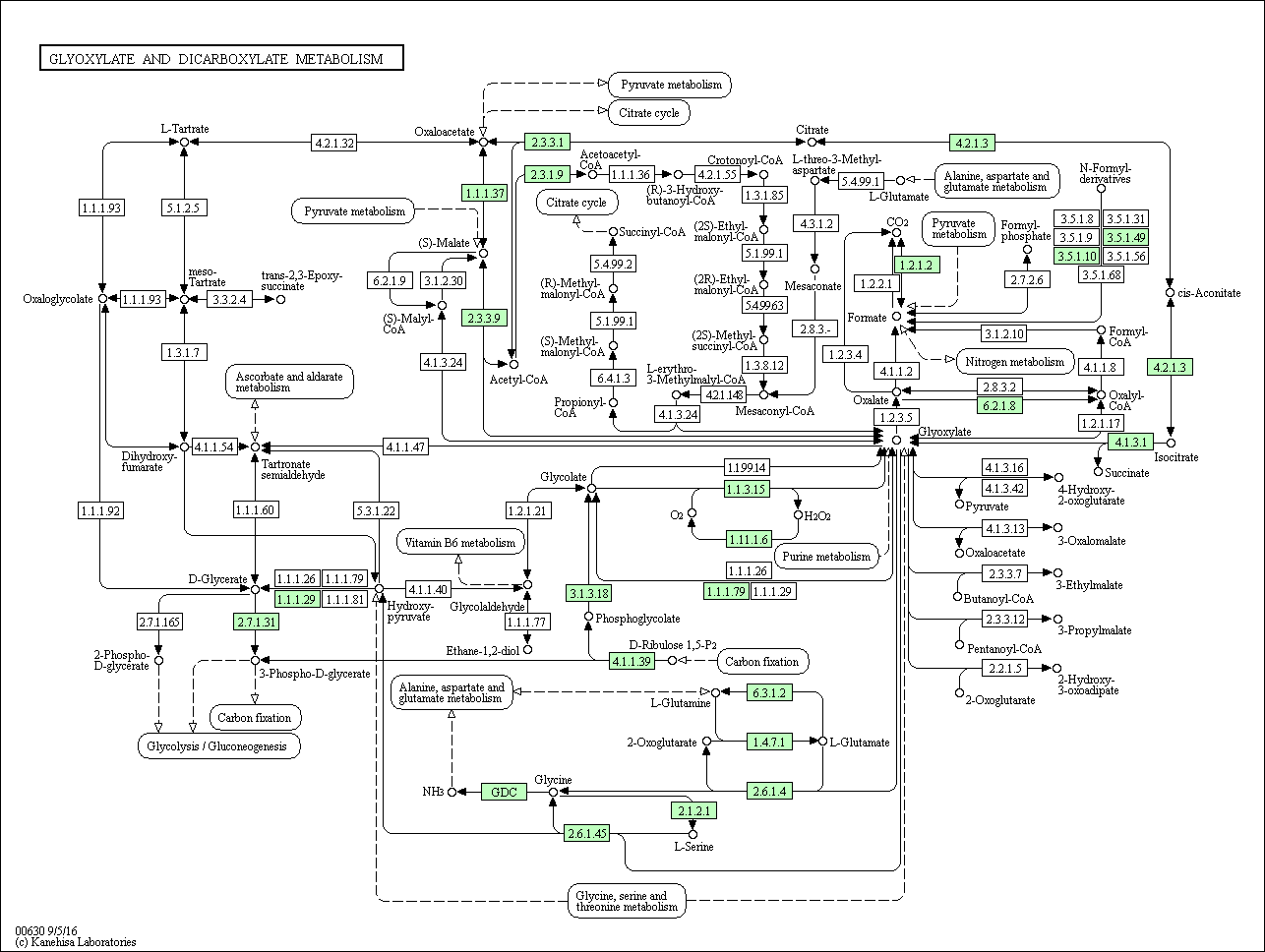

Supplement: S1 File — (ZIP) [file pone.0299259.s004.zip › S1 Zip/src/egu00630.png]

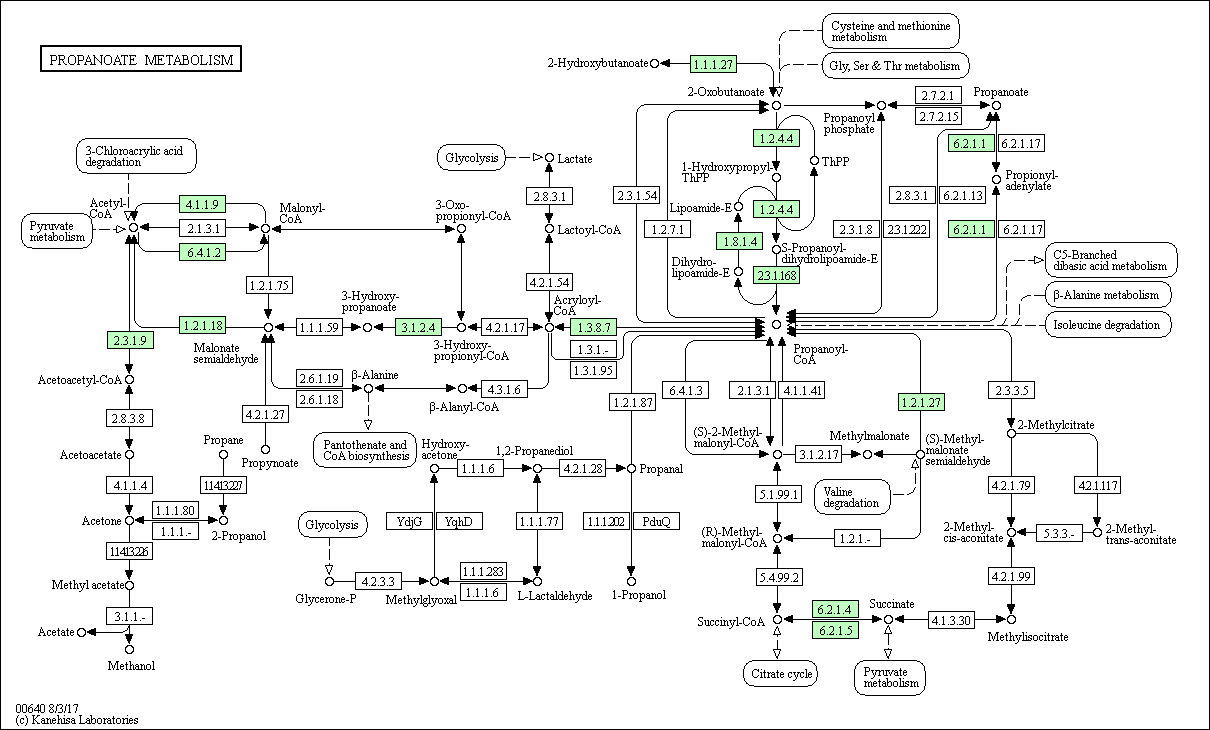

Supplement: S1 File — (ZIP) [file pone.0299259.s004.zip › S1 Zip/src/egu00640.png]

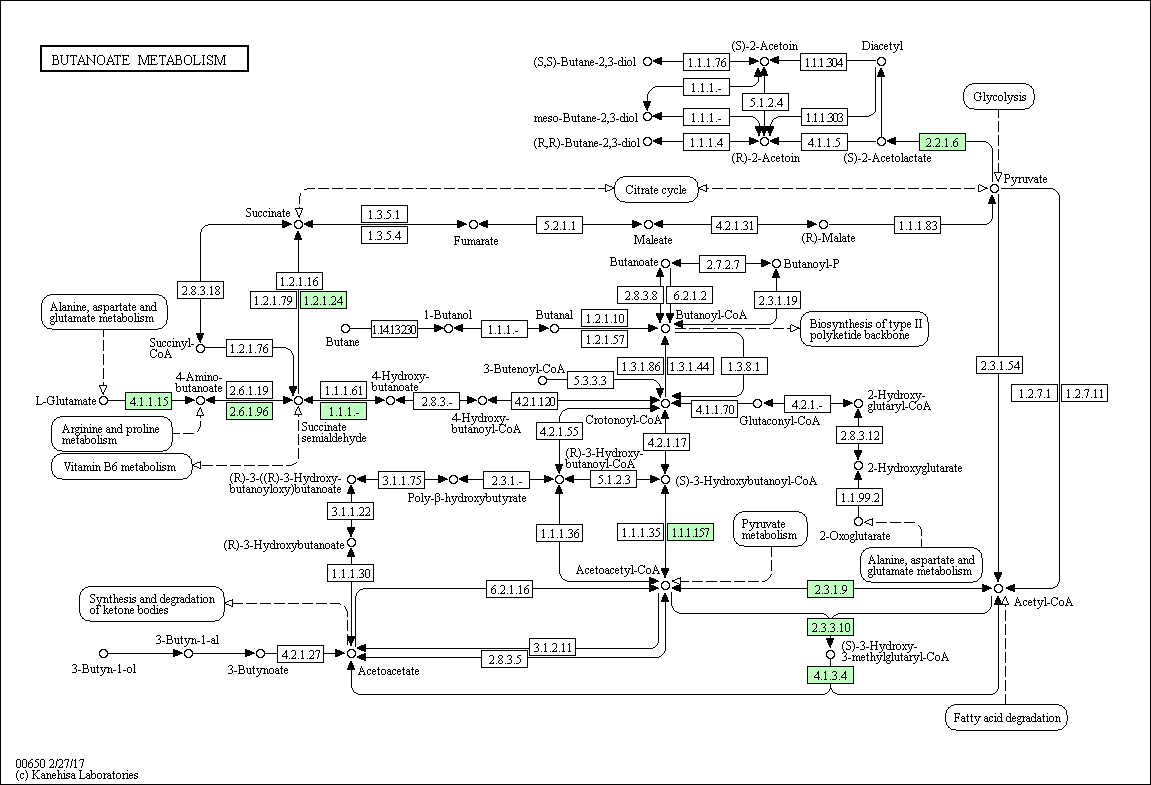

Supplement: S1 File — (ZIP) [file pone.0299259.s004.zip › S1 Zip/src/egu00650.png]

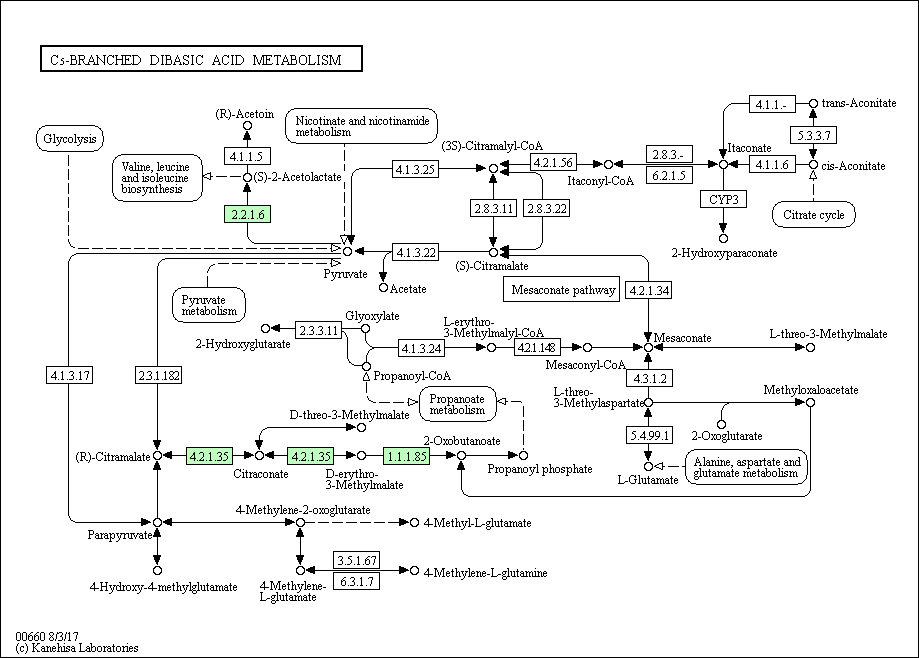

Supplement: S1 File — (ZIP) [file pone.0299259.s004.zip › S1 Zip/src/egu00660.png]

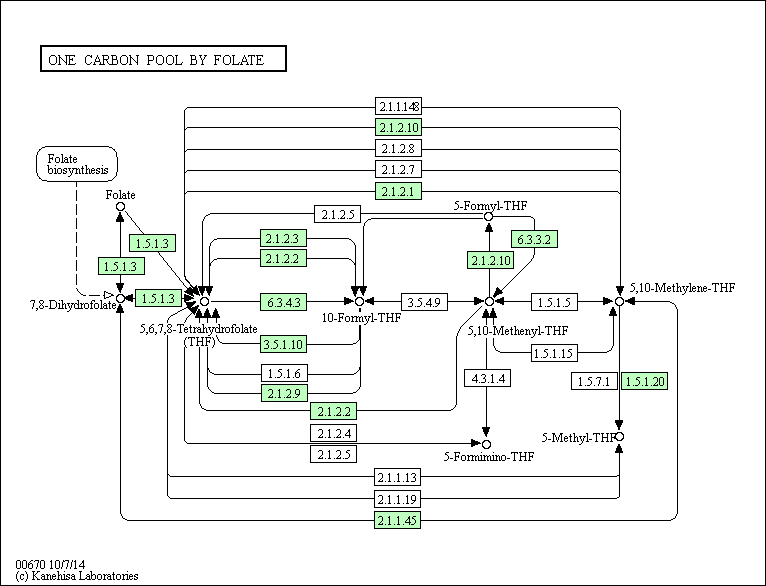

Supplement: S1 File — (ZIP) [file pone.0299259.s004.zip › S1 Zip/src/egu00670.png]

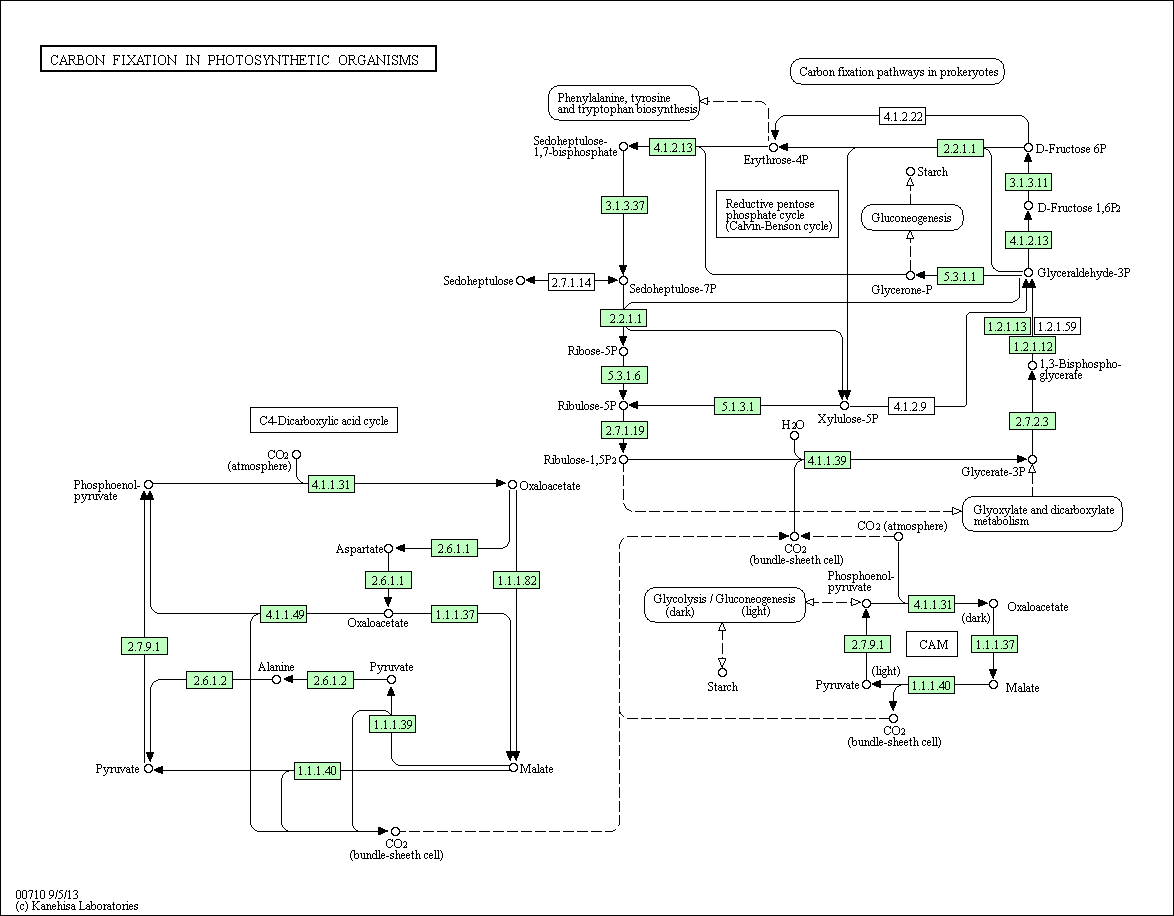

Supplement: S1 File — (ZIP) [file pone.0299259.s004.zip › S1 Zip/src/egu00710.png]

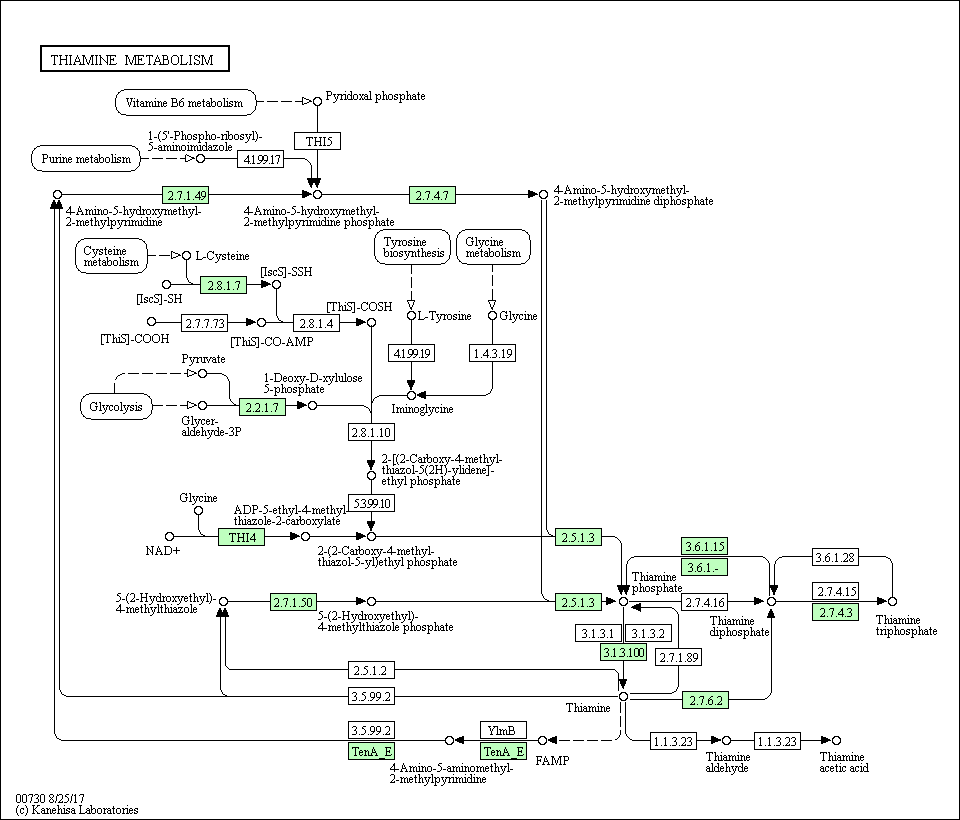

Supplement: S1 File — (ZIP) [file pone.0299259.s004.zip › S1 Zip/src/egu00730.png]

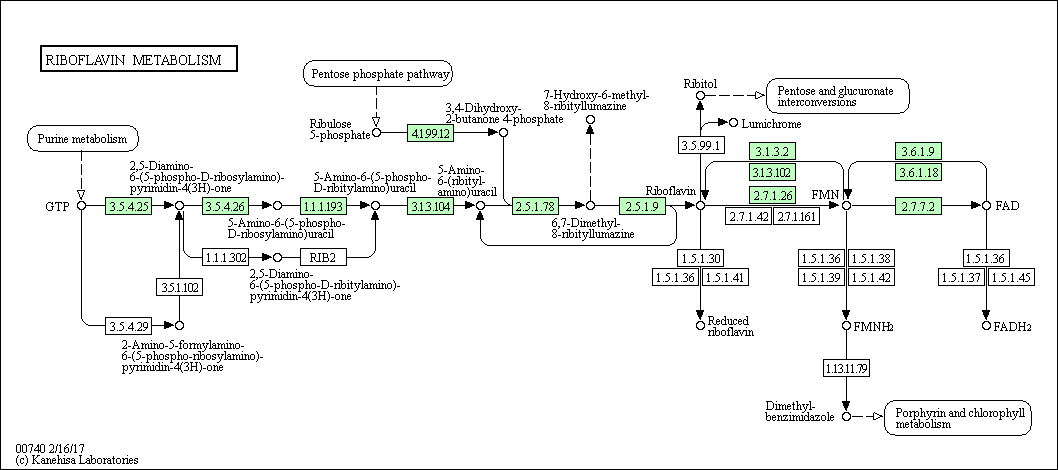

Supplement: S1 File — (ZIP) [file pone.0299259.s004.zip › S1 Zip/src/egu00740.png]

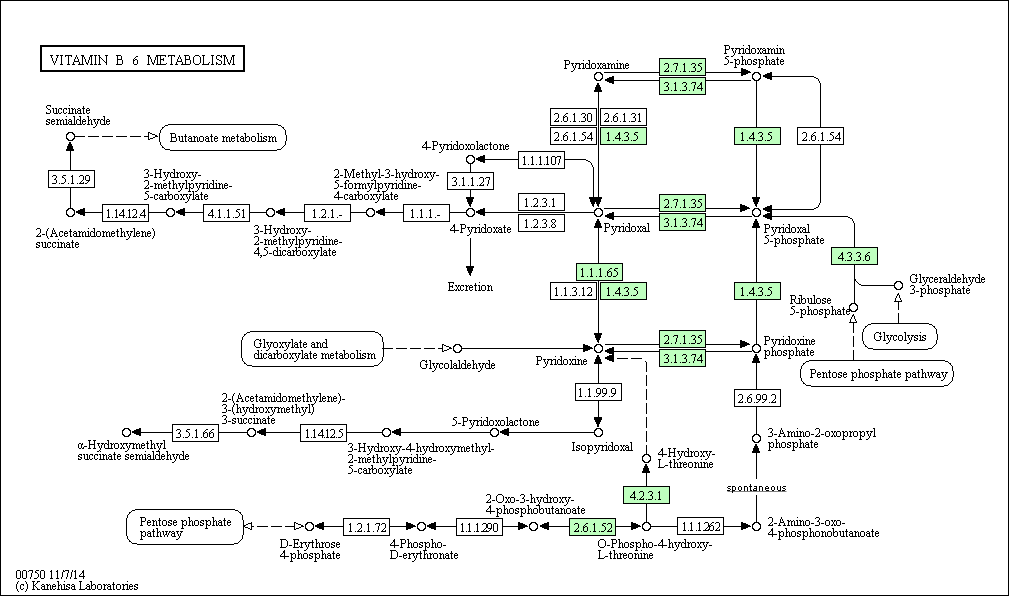

Supplement: S1 File — (ZIP) [file pone.0299259.s004.zip › S1 Zip/src/egu00750.png]

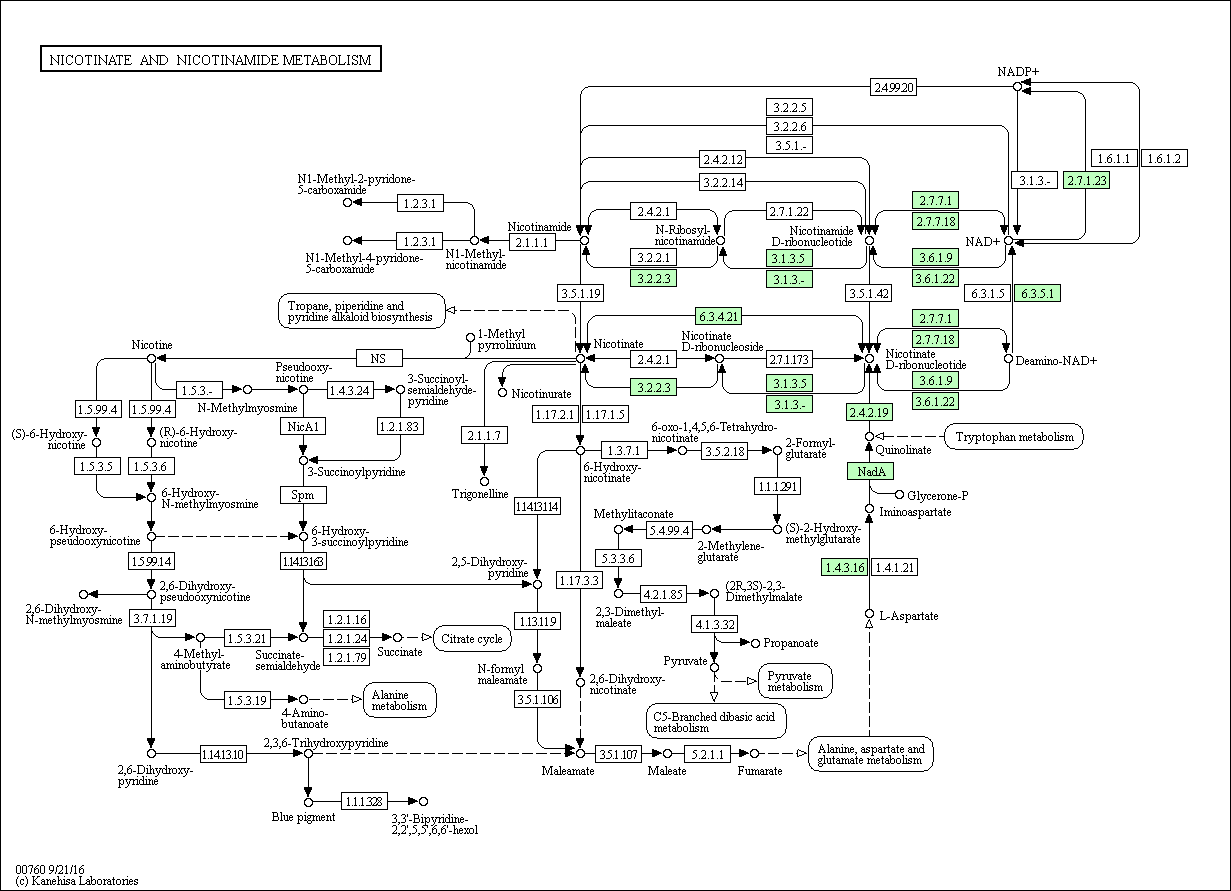

Supplement: S1 File — (ZIP) [file pone.0299259.s004.zip › S1 Zip/src/egu00760.png]

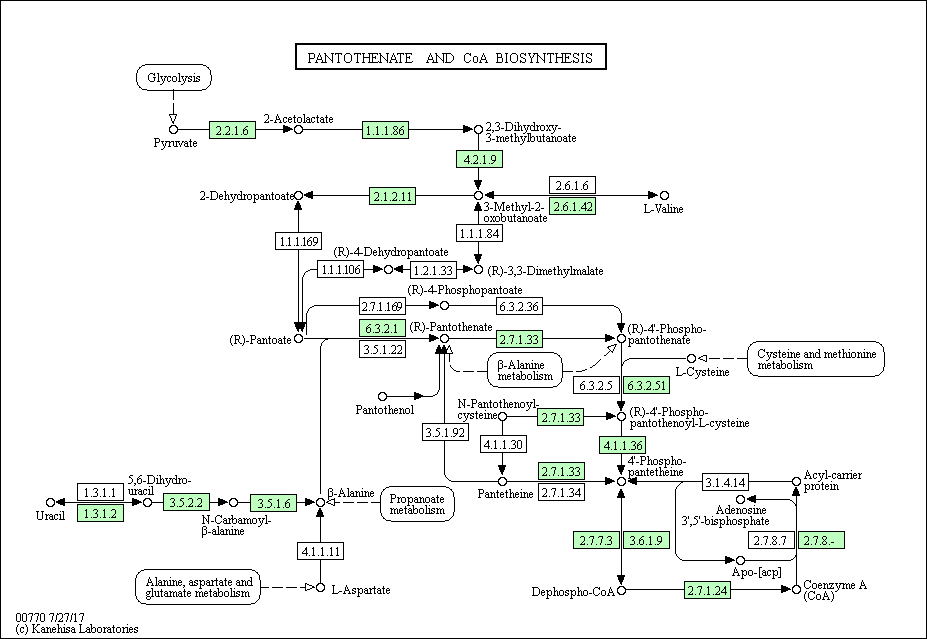

Supplement: S1 File — (ZIP) [file pone.0299259.s004.zip › S1 Zip/src/egu00770.png]

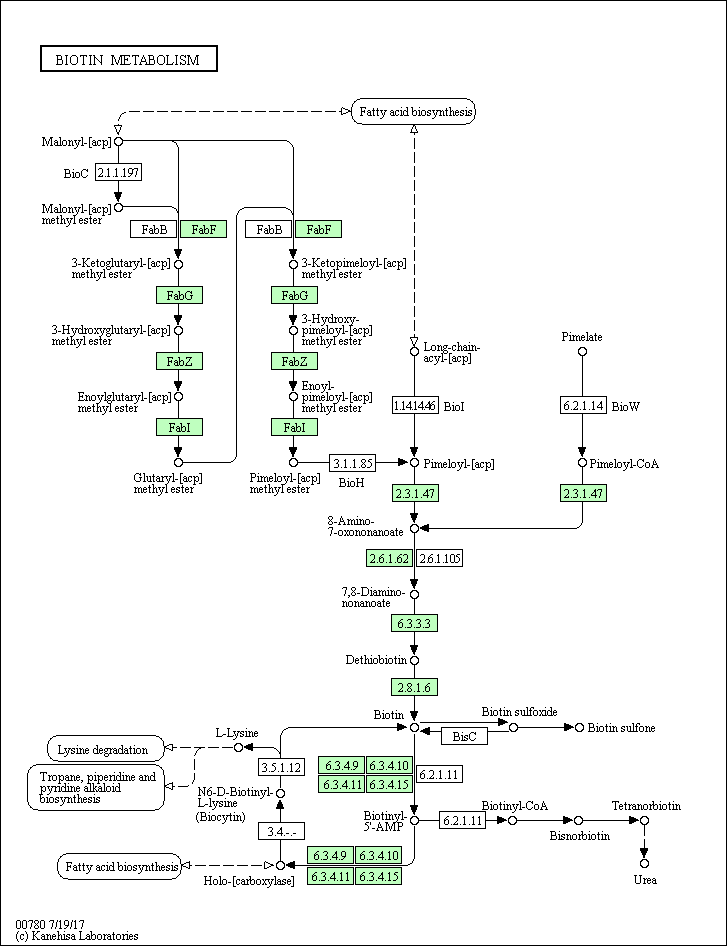

Supplement: S1 File — (ZIP) [file pone.0299259.s004.zip › S1 Zip/src/egu00780.png]

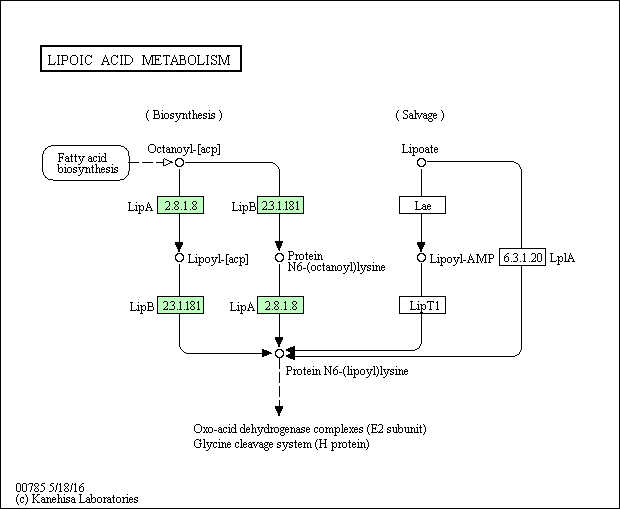

Supplement: S1 File — (ZIP) [file pone.0299259.s004.zip › S1 Zip/src/egu00785.png]

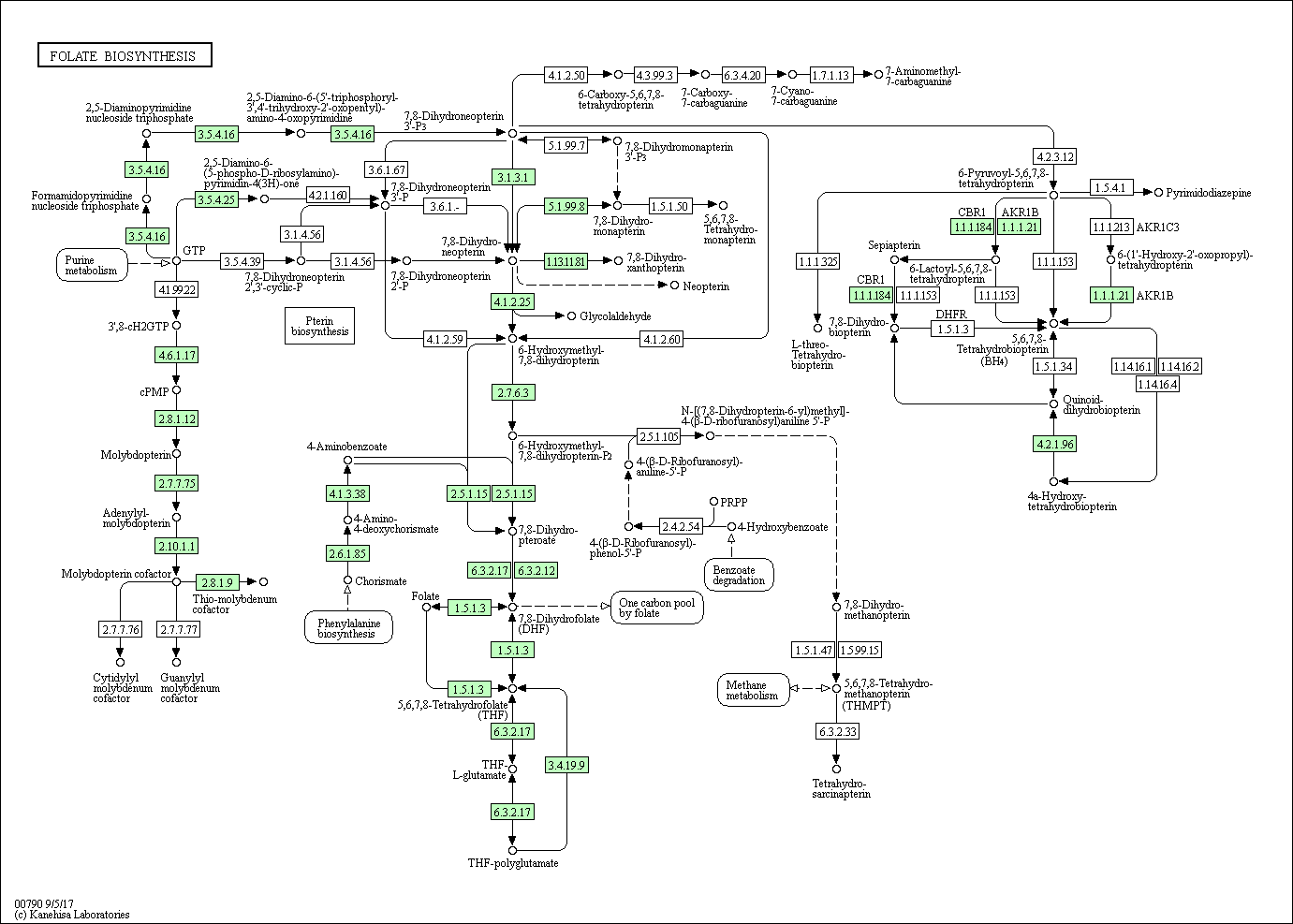

Supplement: S1 File — (ZIP) [file pone.0299259.s004.zip › S1 Zip/src/egu00790.png]

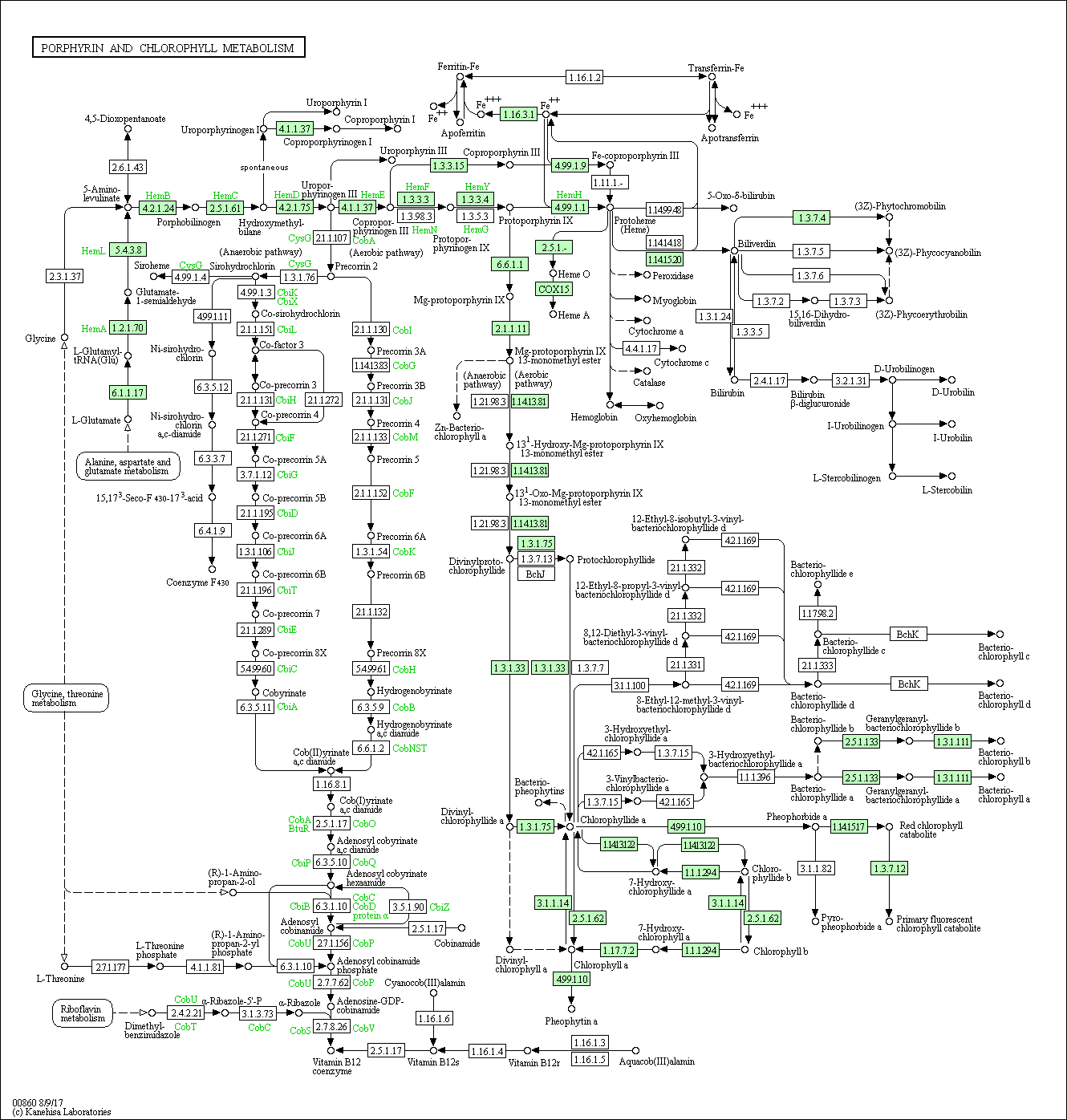

Supplement: S1 File — (ZIP) [file pone.0299259.s004.zip › S1 Zip/src/egu00860.png]

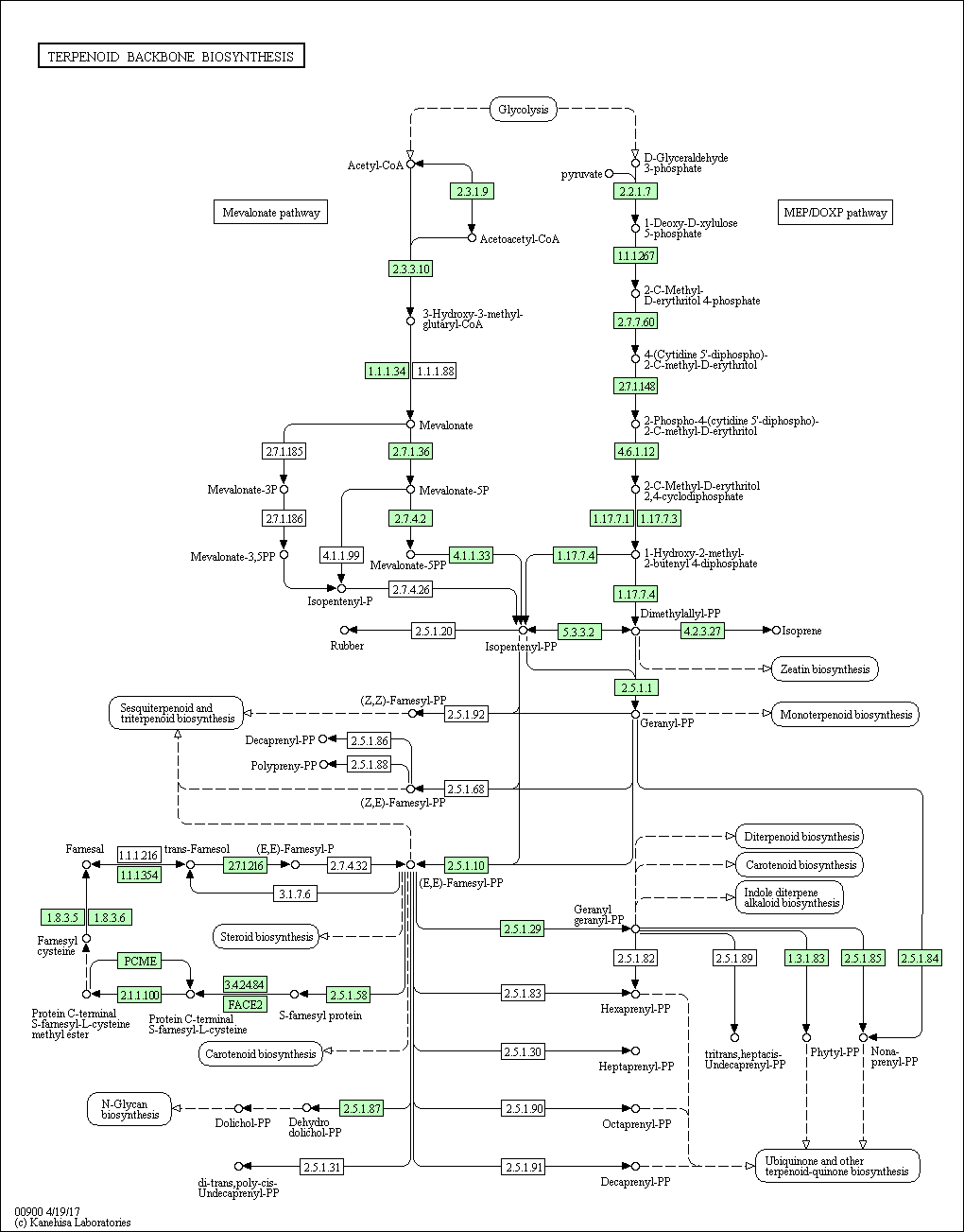

Supplement: S1 File — (ZIP) [file pone.0299259.s004.zip › S1 Zip/src/egu00900.png]

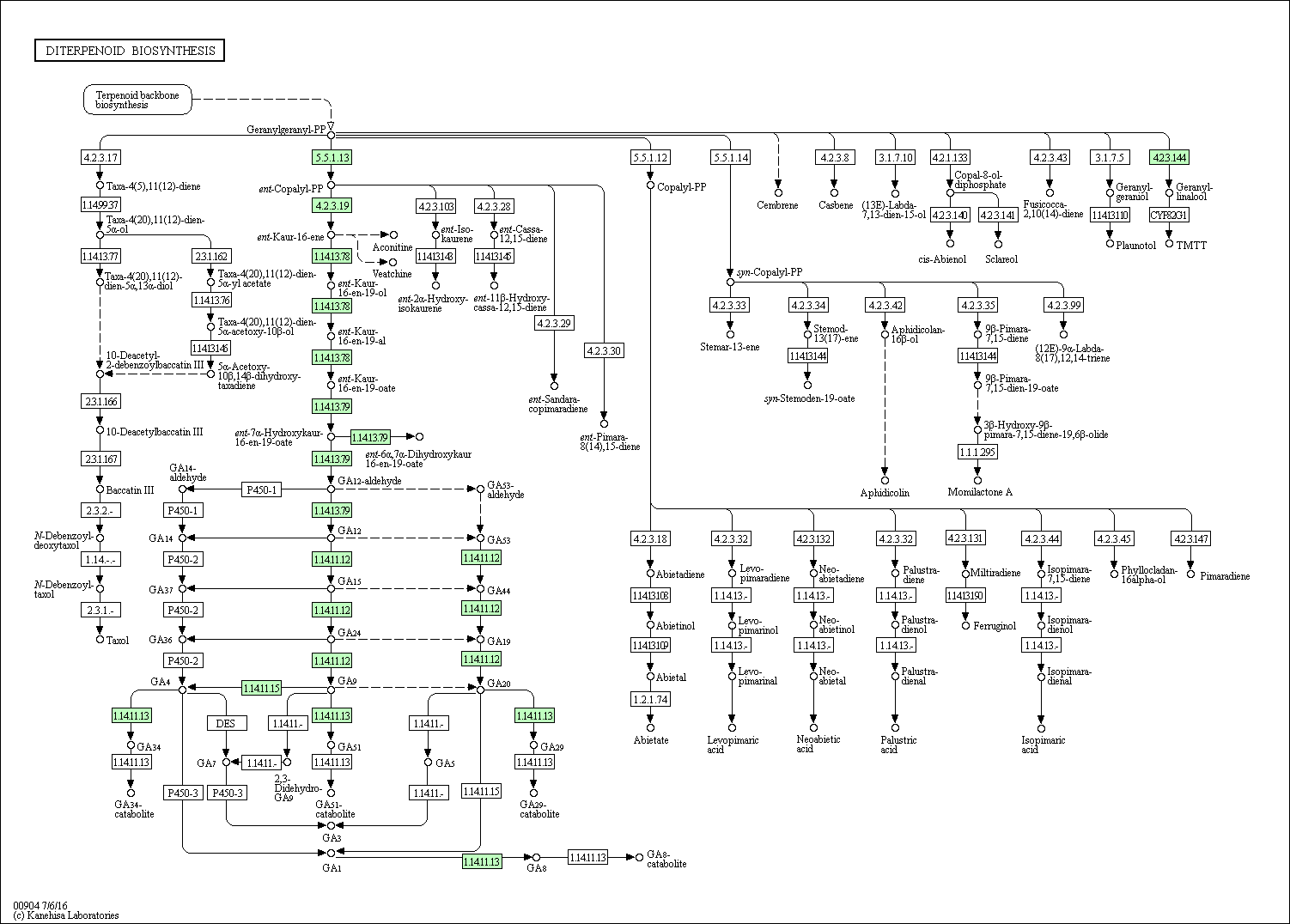

Supplement: S1 File — (ZIP) [file pone.0299259.s004.zip › S1 Zip/src/egu00904.png]

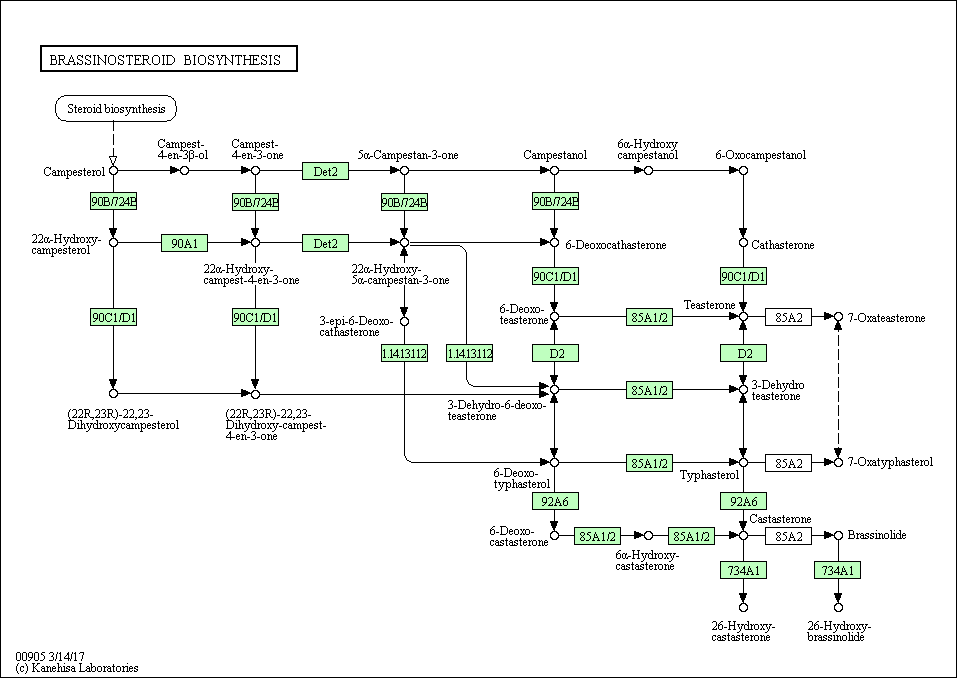

Supplement: S1 File — (ZIP) [file pone.0299259.s004.zip › S1 Zip/src/egu00905.png]

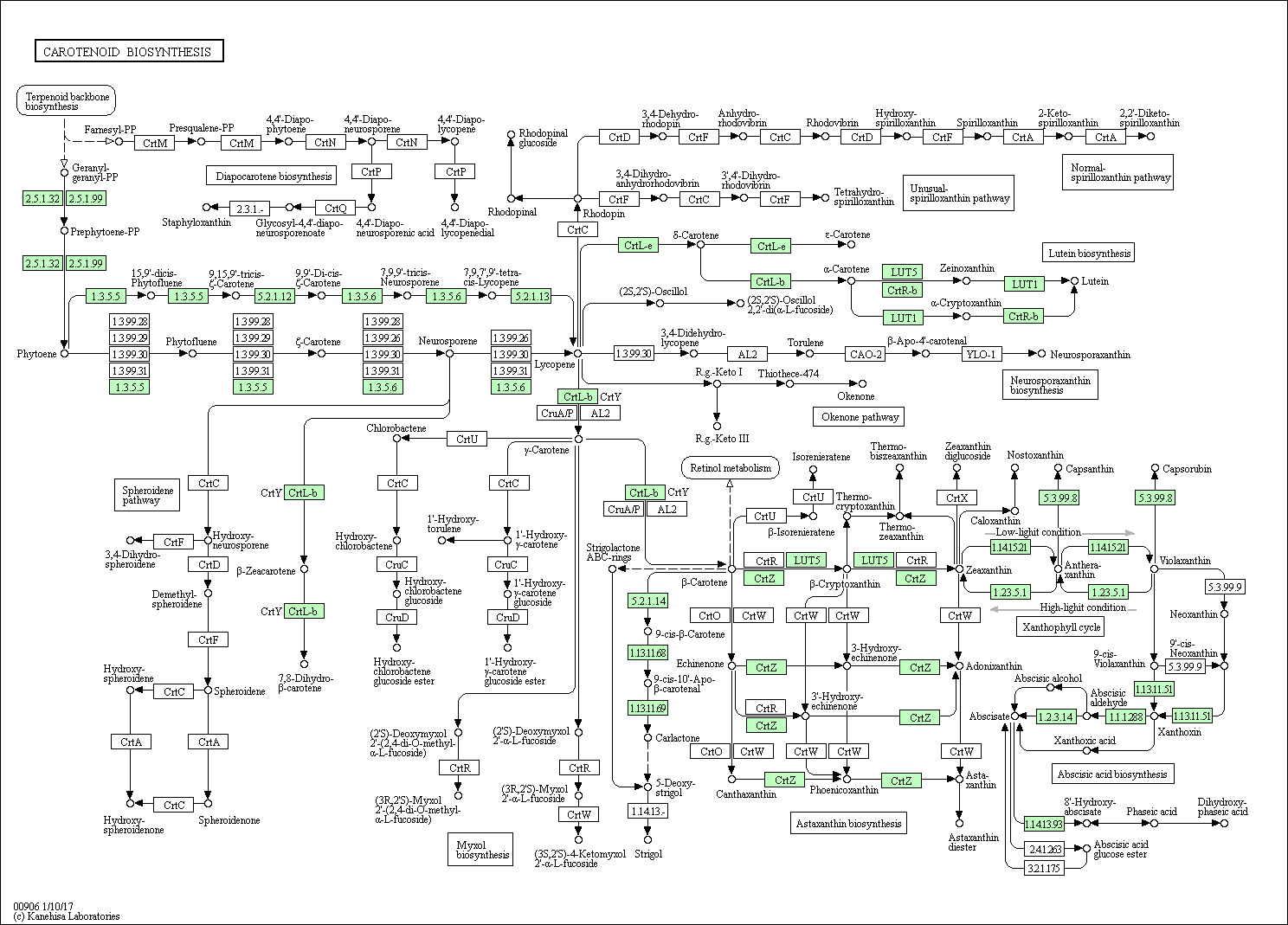

Supplement: S1 File — (ZIP) [file pone.0299259.s004.zip › S1 Zip/src/egu00906.png]

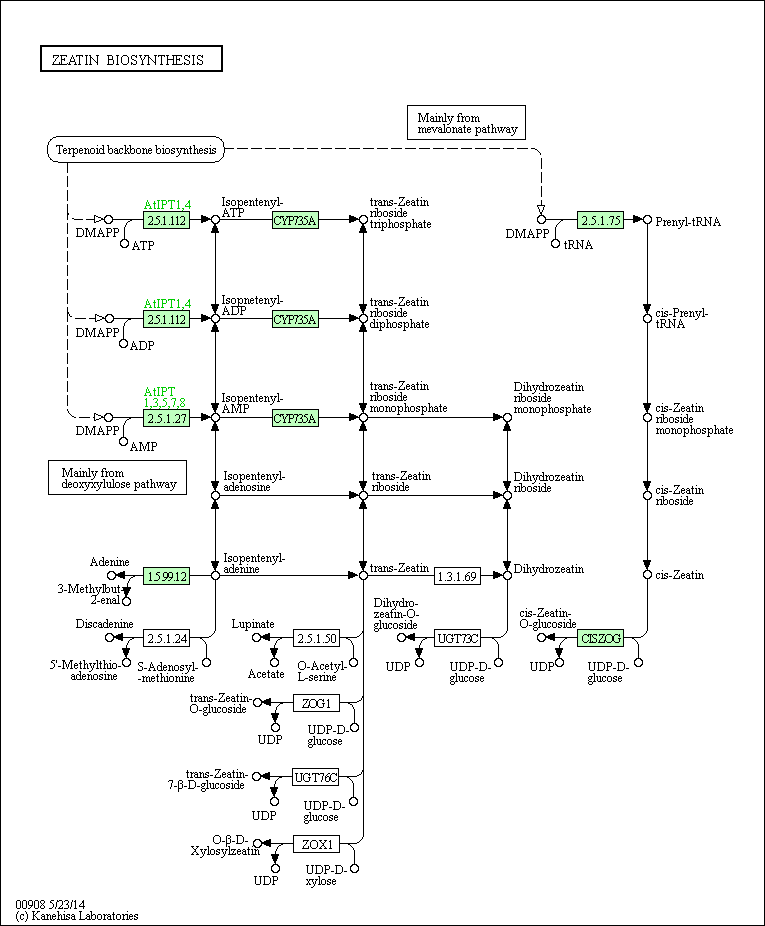

Supplement: S1 File — (ZIP) [file pone.0299259.s004.zip › S1 Zip/src/egu00908.png]

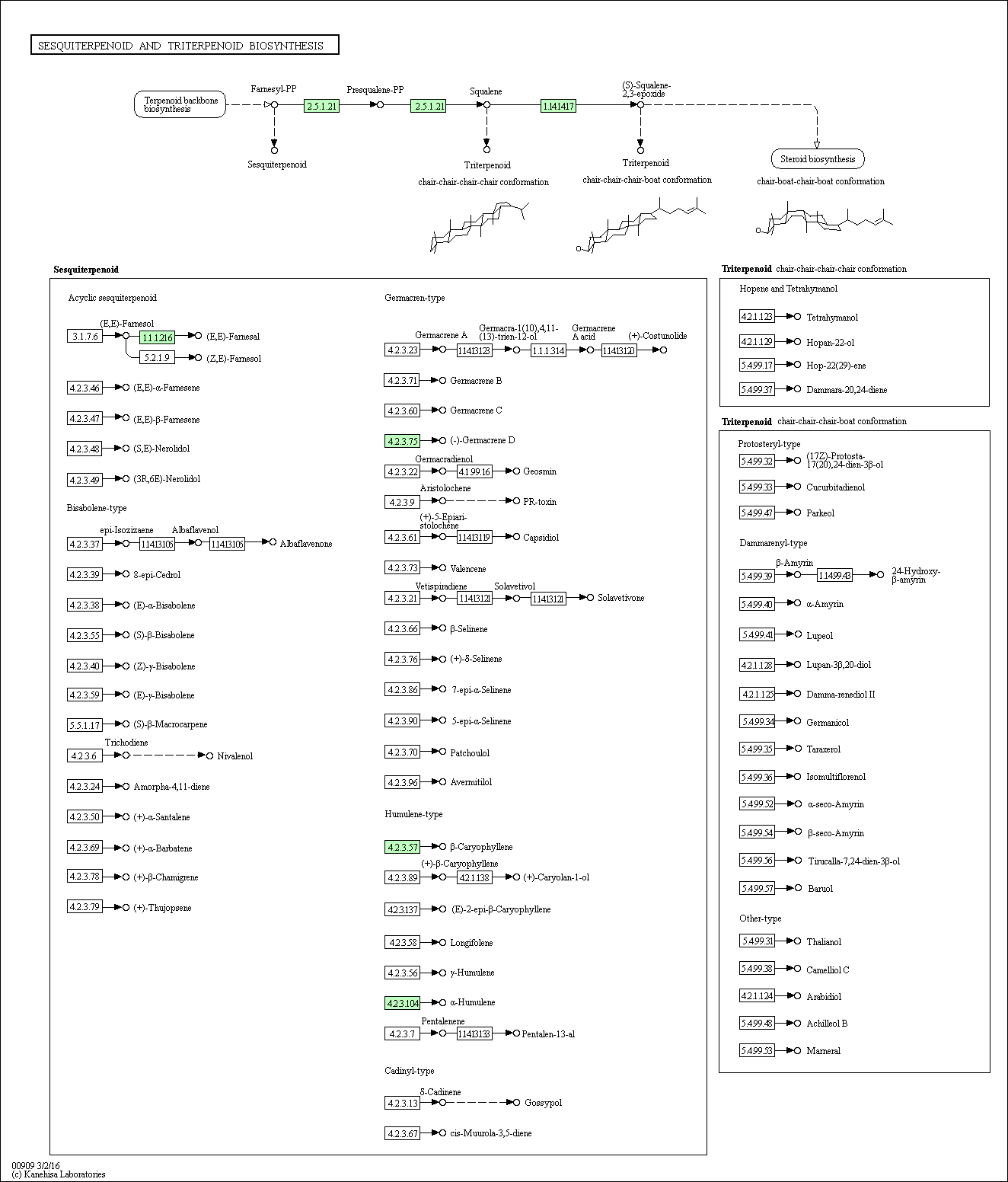

Supplement: S1 File — (ZIP) [file pone.0299259.s004.zip › S1 Zip/src/egu00909.png]

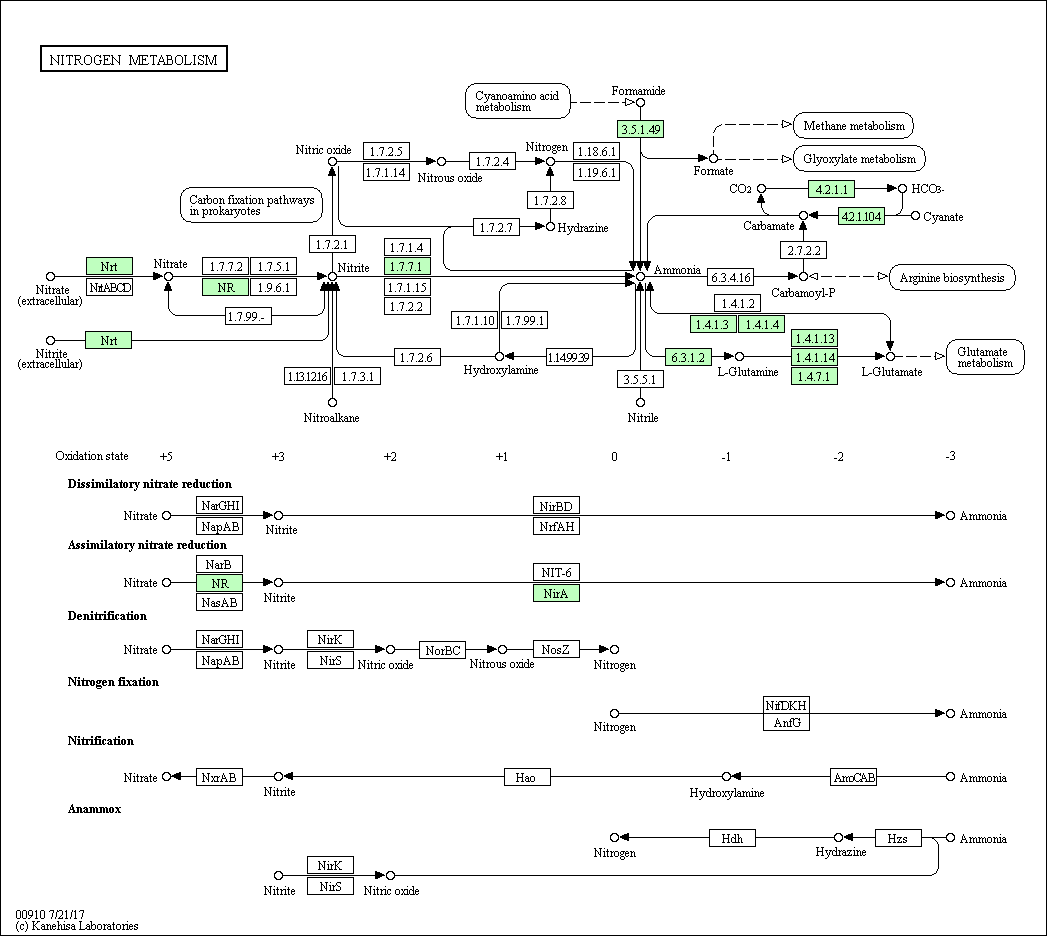

Supplement: S1 File — (ZIP) [file pone.0299259.s004.zip › S1 Zip/src/egu00910.png]

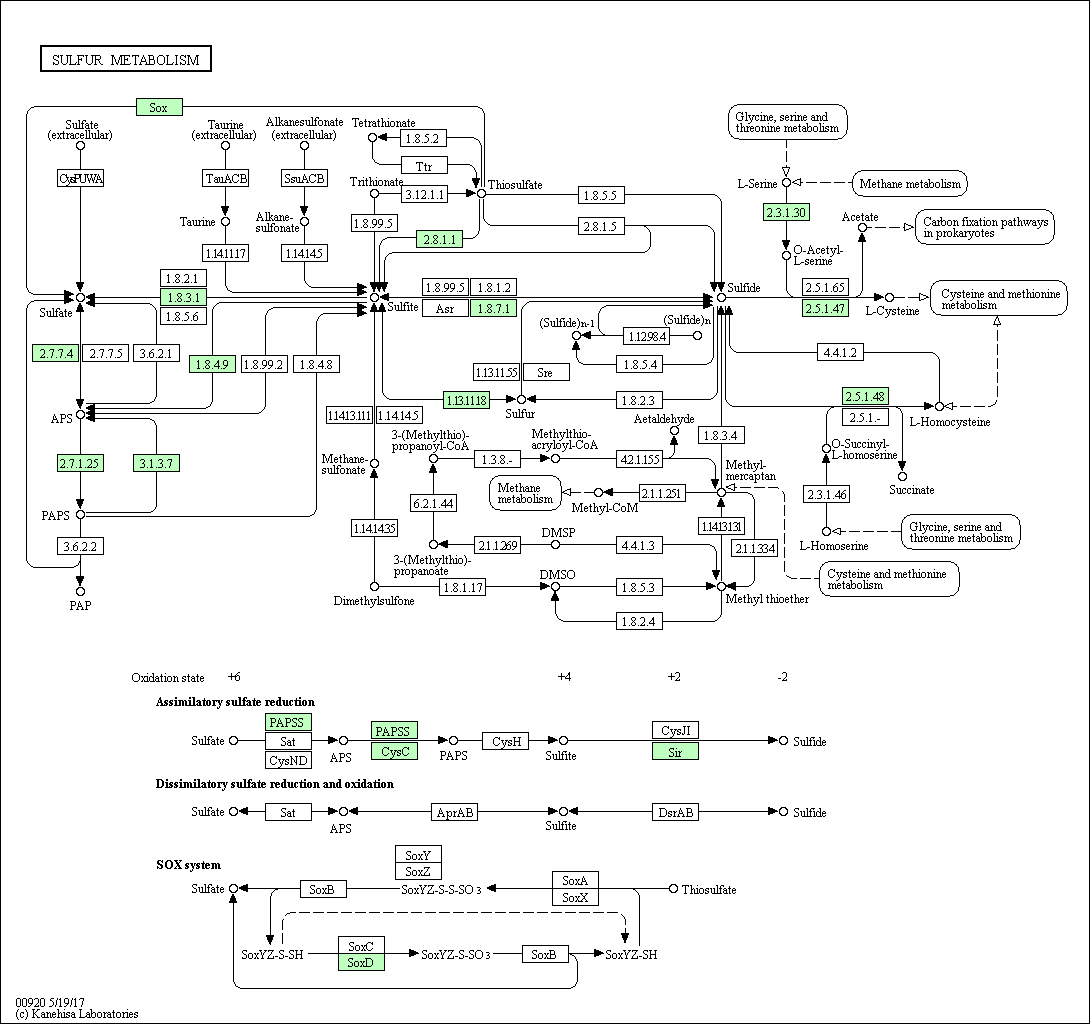

Supplement: S1 File — (ZIP) [file pone.0299259.s004.zip › S1 Zip/src/egu00920.png]

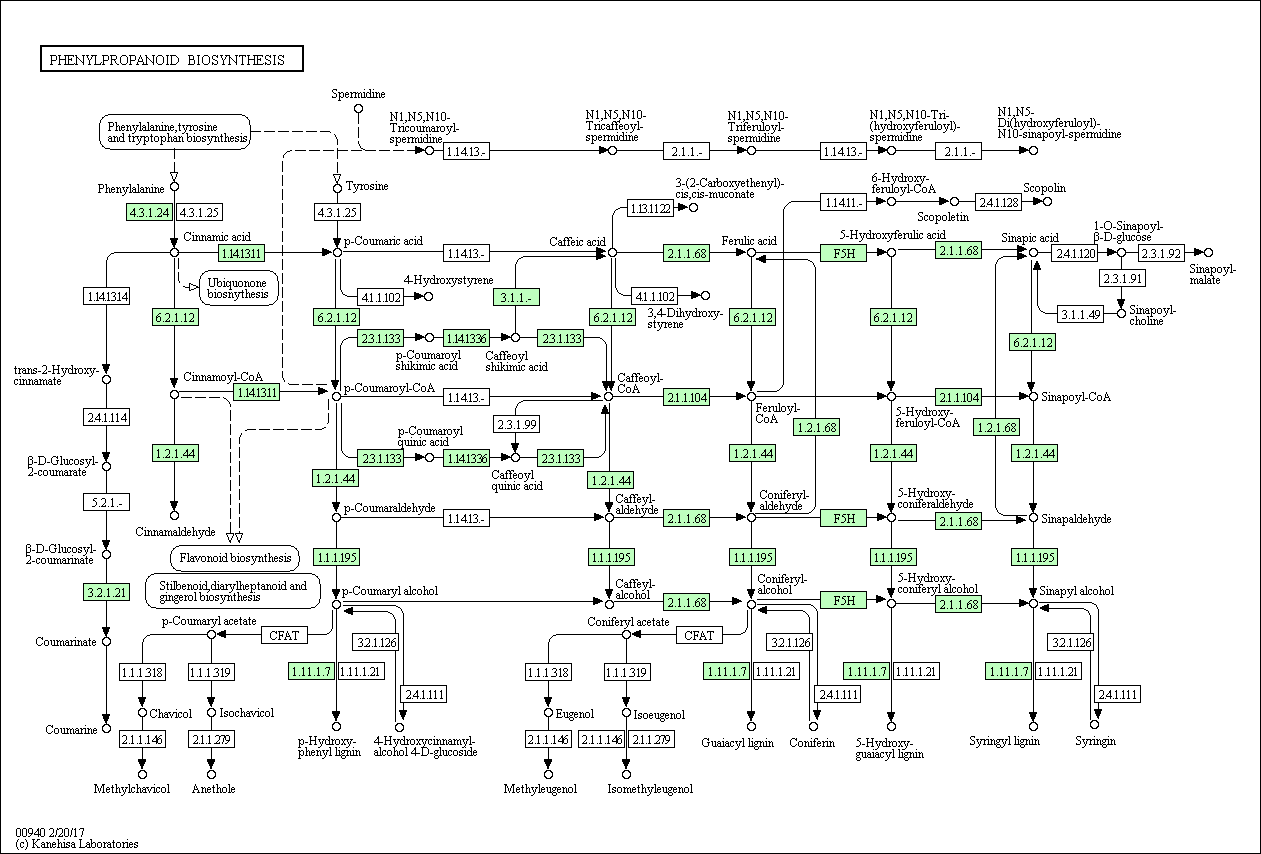

Supplement: S1 File — (ZIP) [file pone.0299259.s004.zip › S1 Zip/src/egu00940.png]

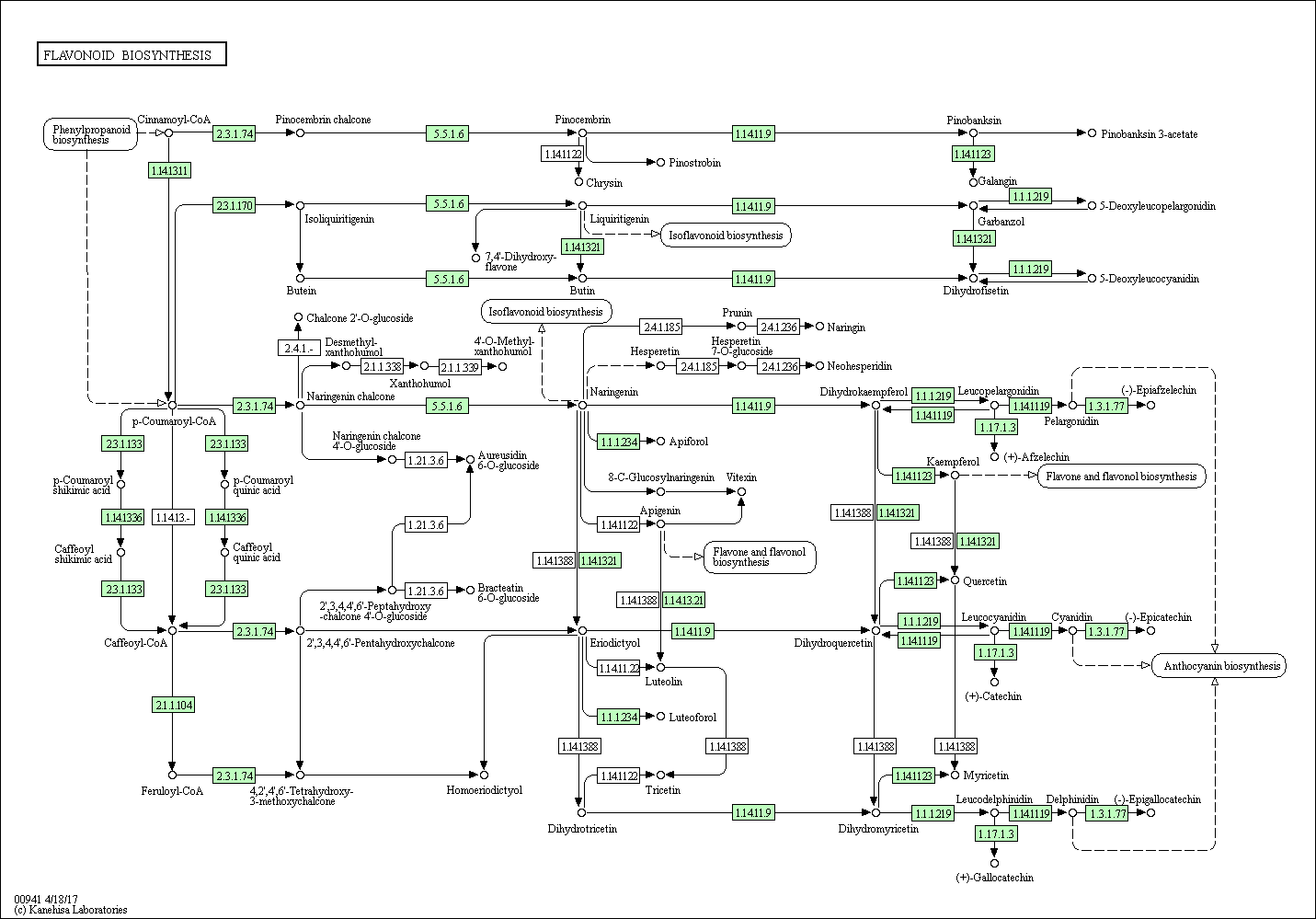

Supplement: S1 File — (ZIP) [file pone.0299259.s004.zip › S1 Zip/src/egu00941.png]

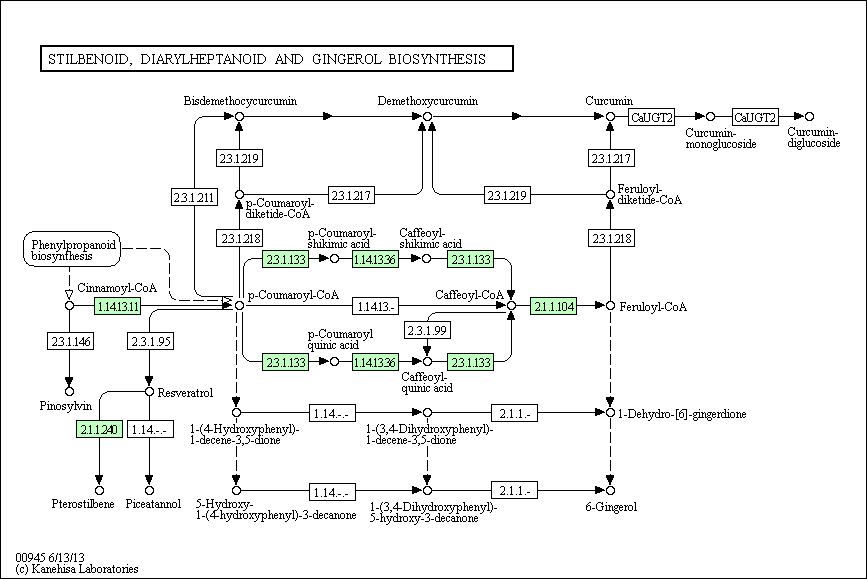

Supplement: S1 File — (ZIP) [file pone.0299259.s004.zip › S1 Zip/src/egu00945.png]

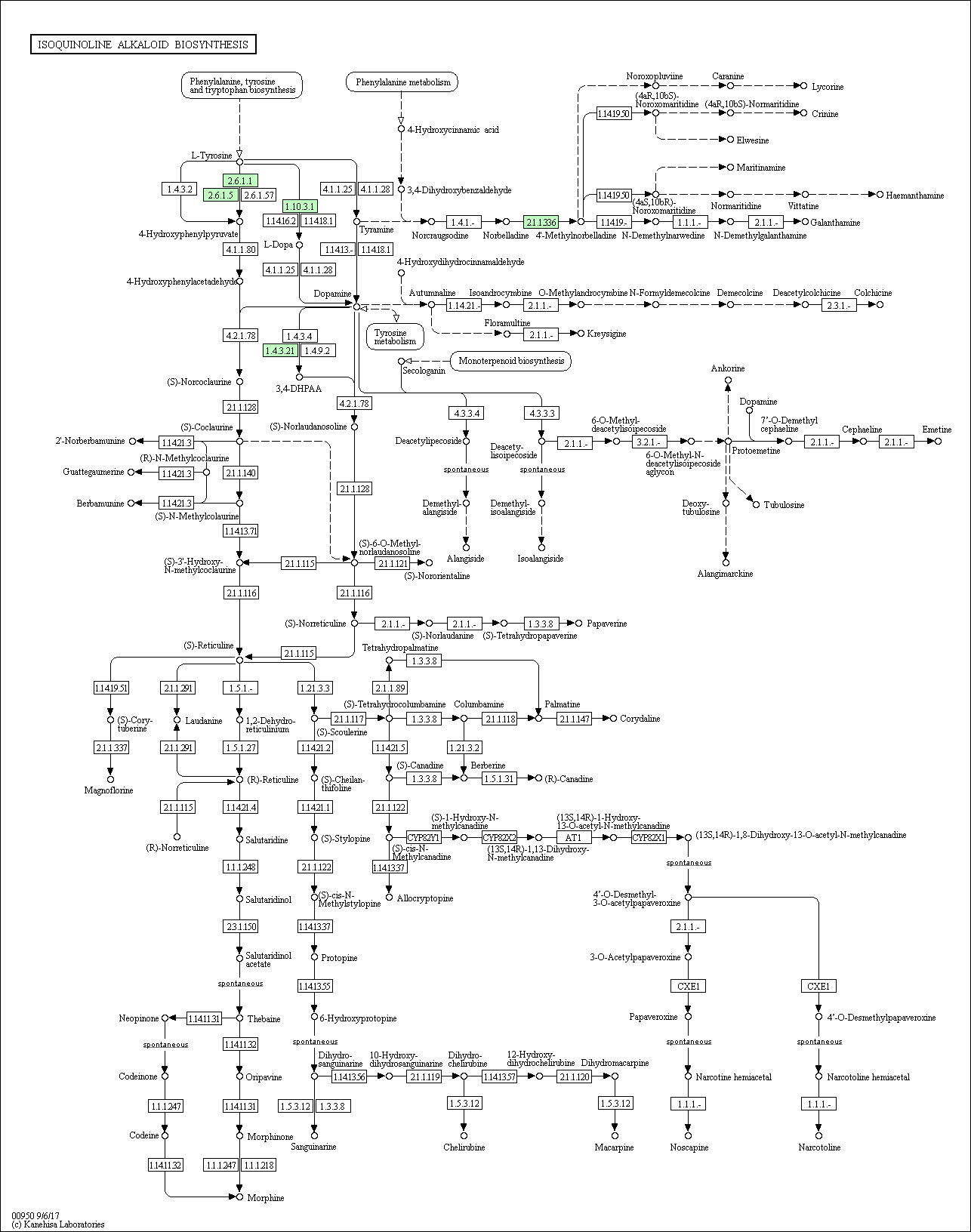

Supplement: S1 File — (ZIP) [file pone.0299259.s004.zip › S1 Zip/src/egu00950.png]

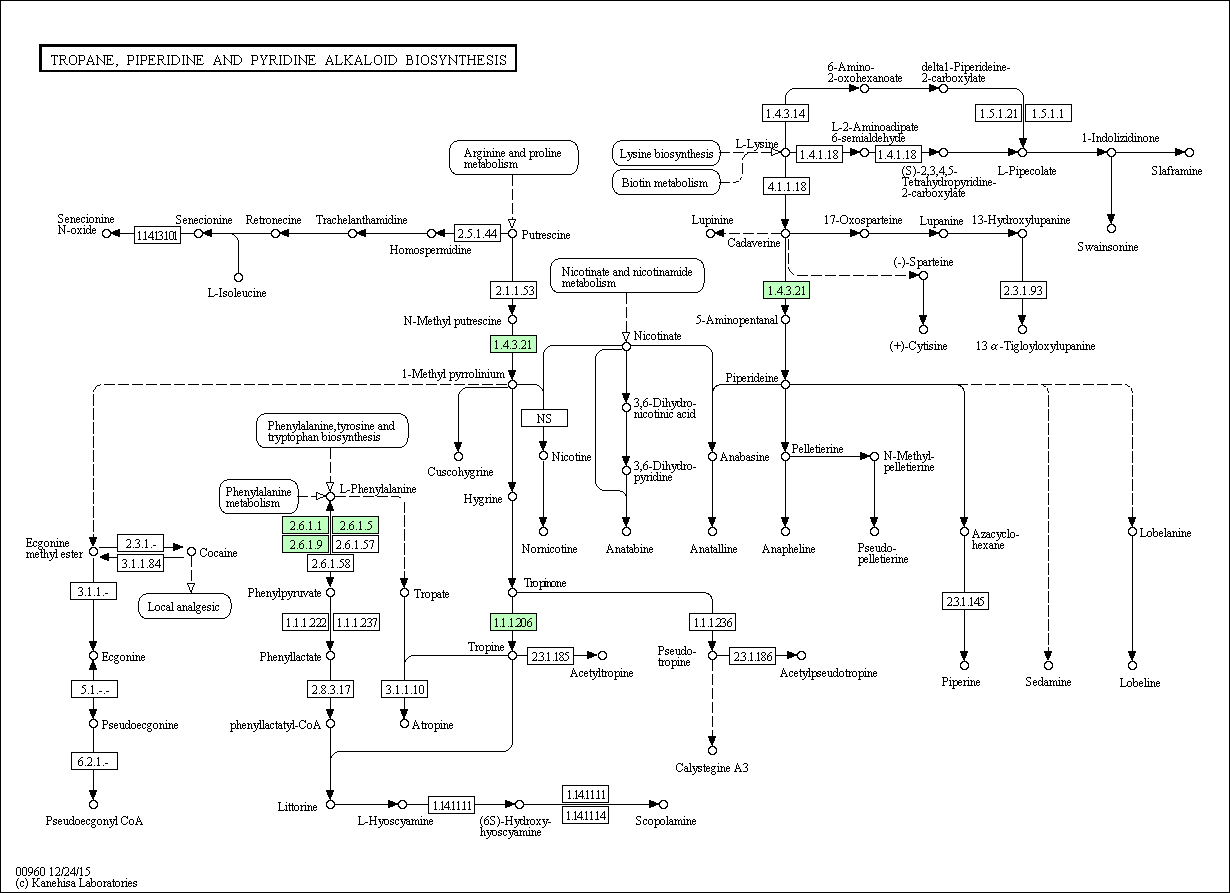

Supplement: S1 File — (ZIP) [file pone.0299259.s004.zip › S1 Zip/src/egu00960.png]

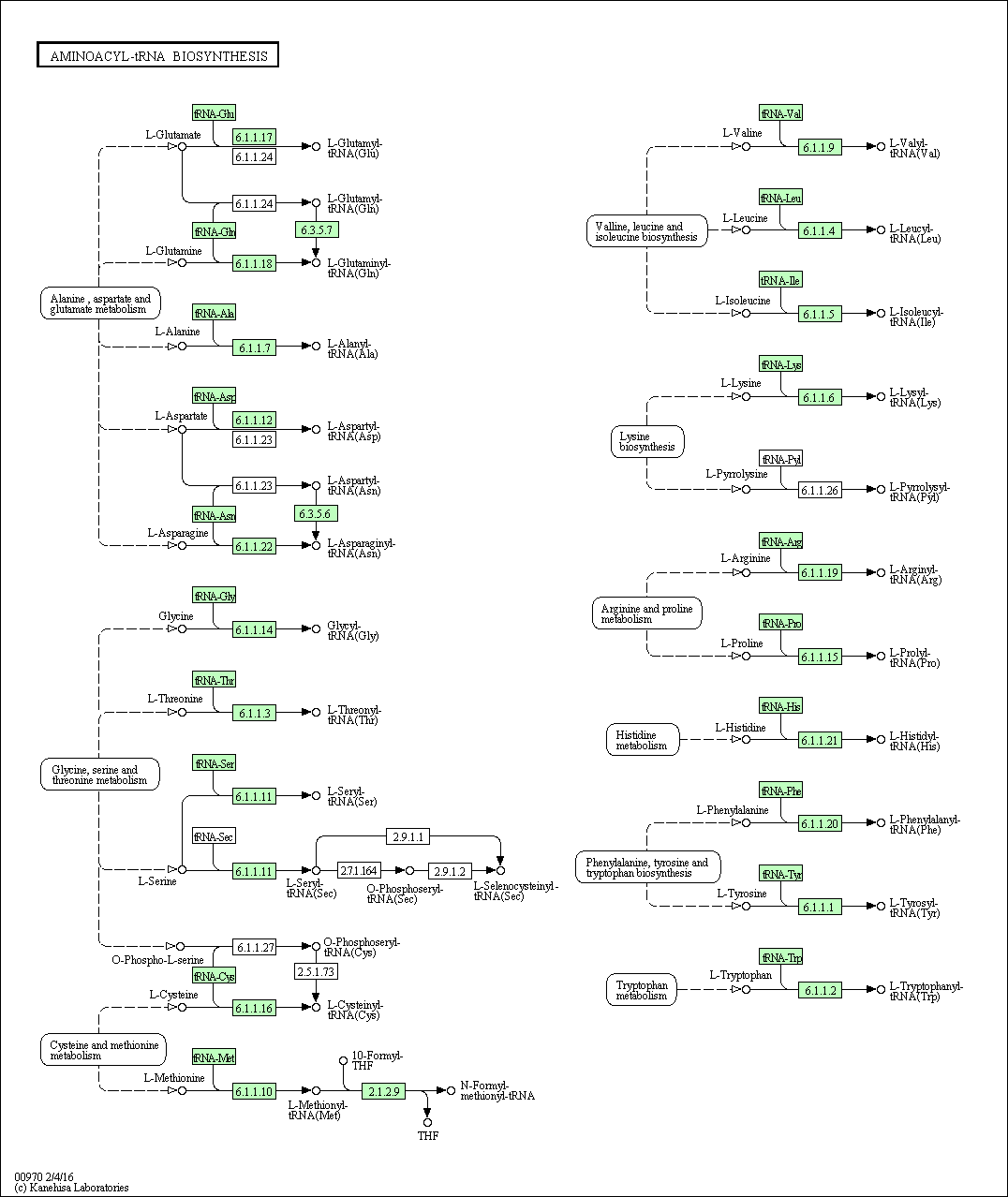

Supplement: S1 File — (ZIP) [file pone.0299259.s004.zip › S1 Zip/src/egu00970.png]

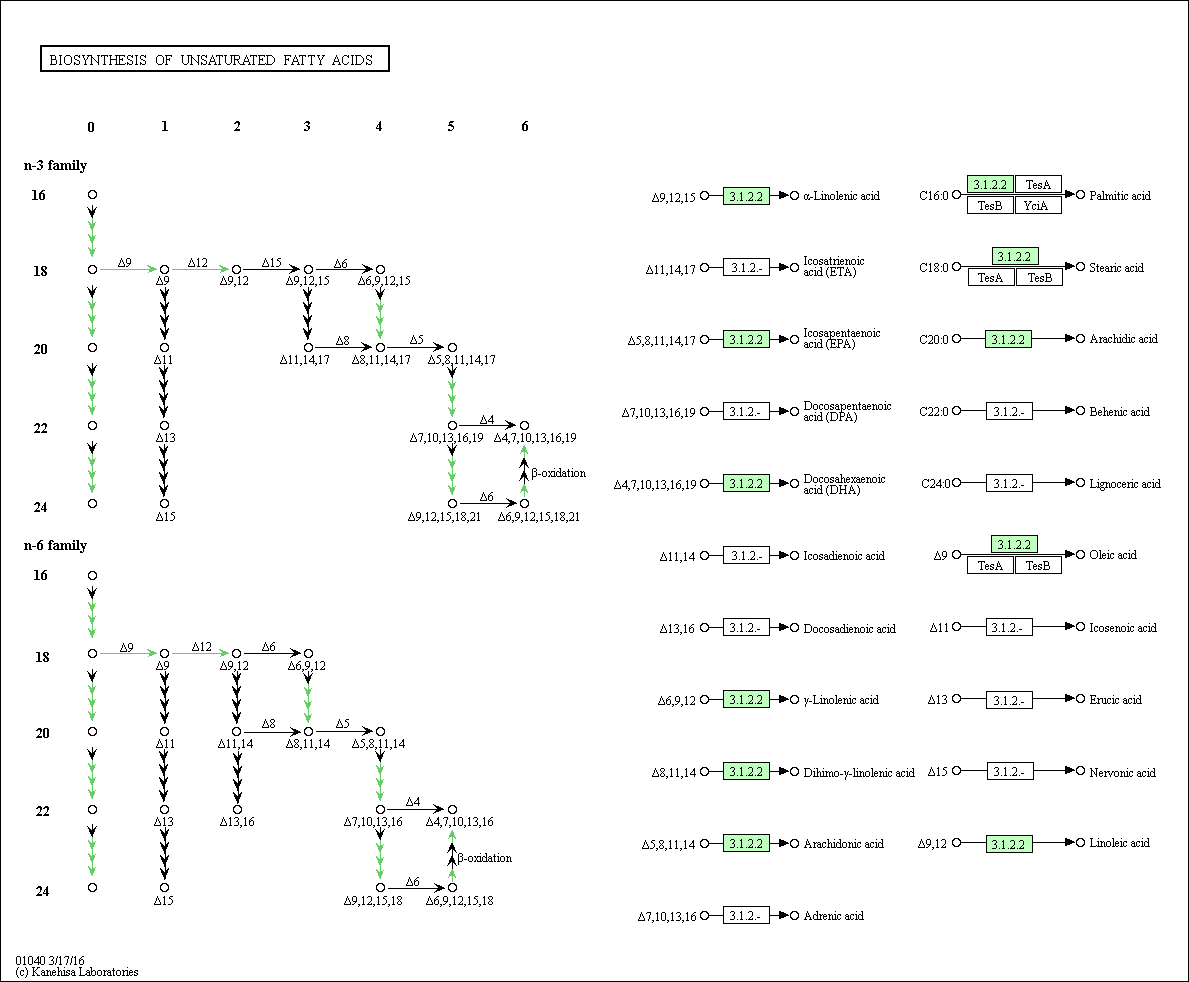

Supplement: S1 File — (ZIP) [file pone.0299259.s004.zip › S1 Zip/src/egu01040.png]

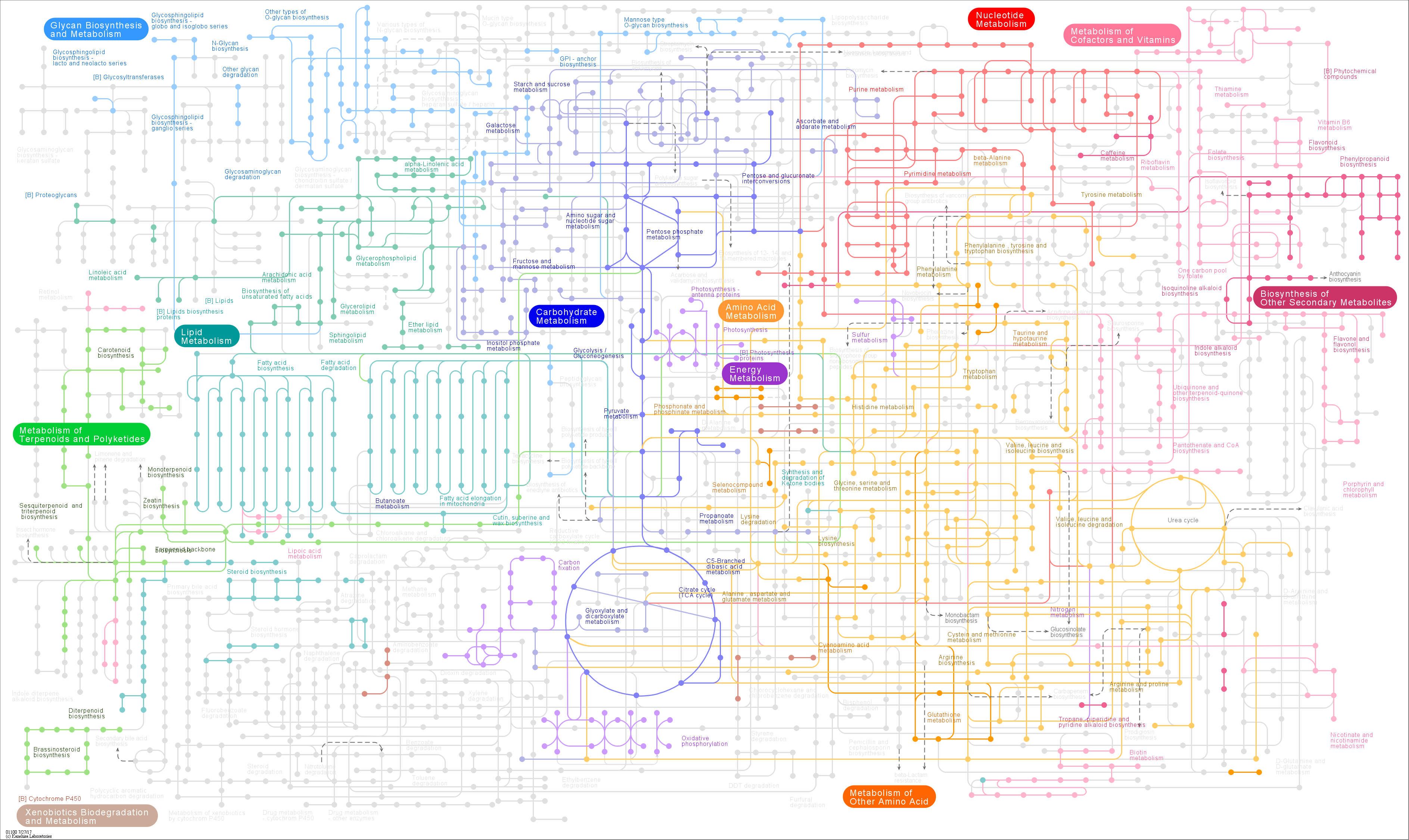

Supplement: S1 File — (ZIP) [file pone.0299259.s004.zip › S1 Zip/src/egu01100.png]

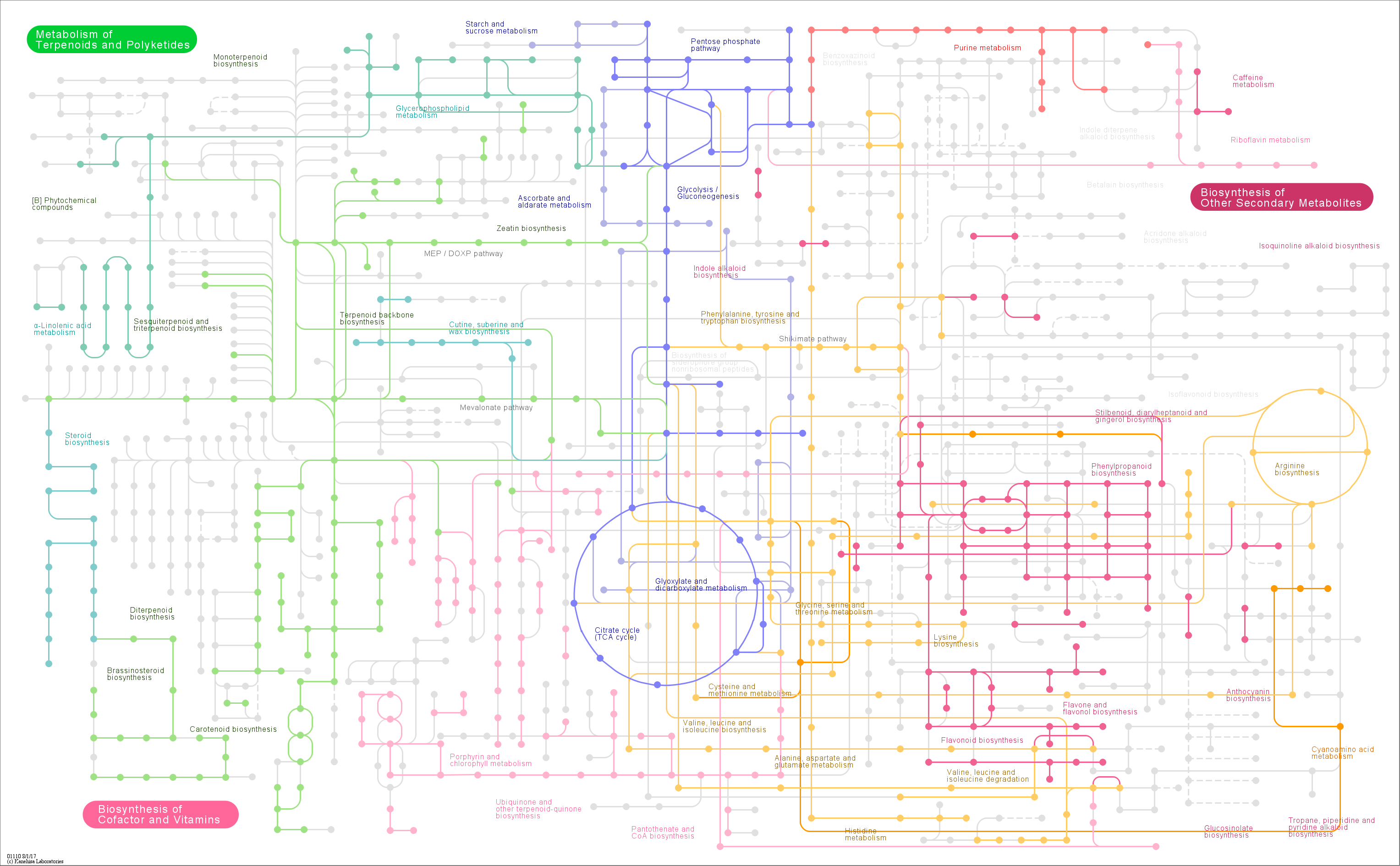

Supplement: S1 File — (ZIP) [file pone.0299259.s004.zip › S1 Zip/src/egu01110.png]

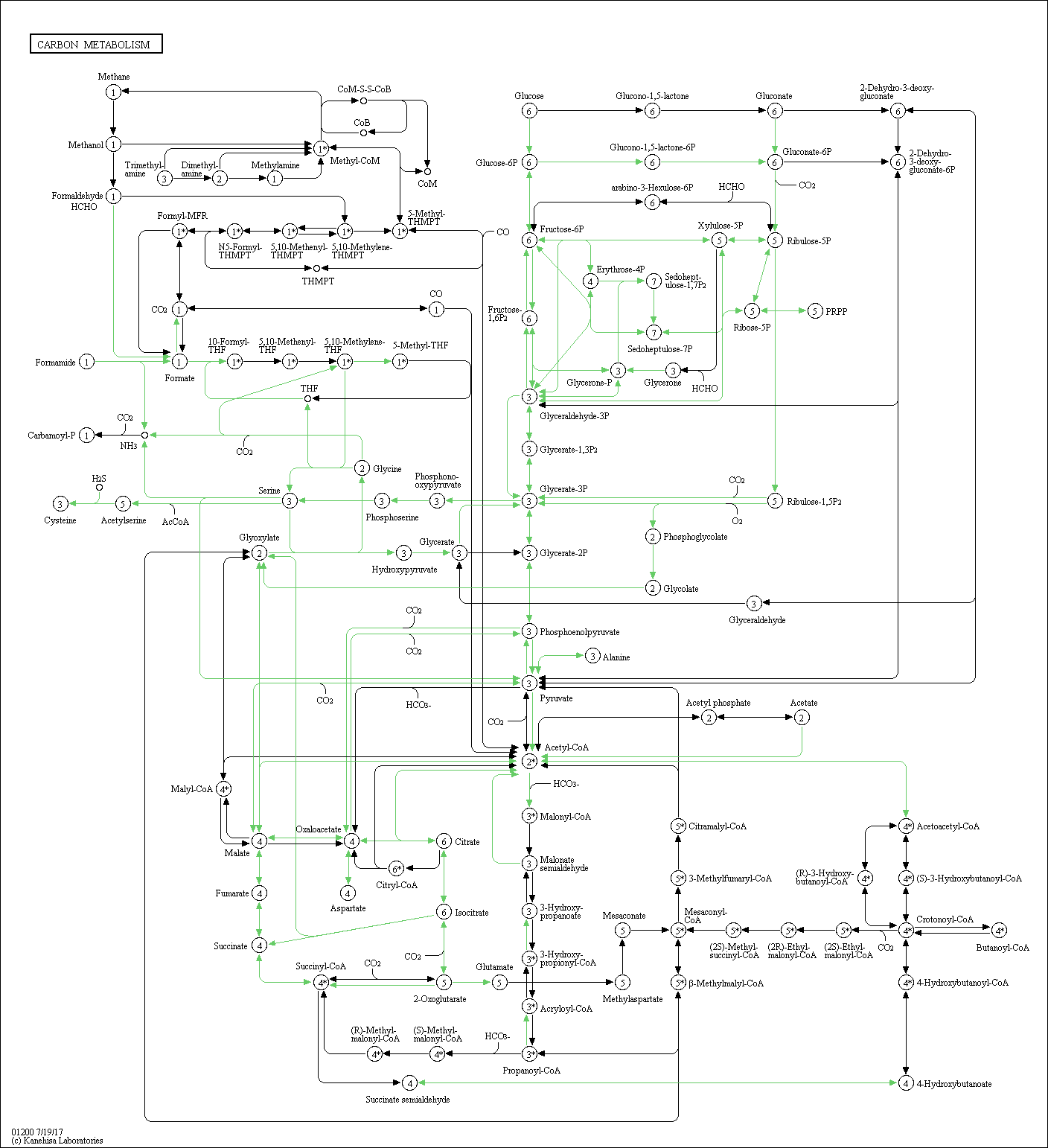

Supplement: S1 File — (ZIP) [file pone.0299259.s004.zip › S1 Zip/src/egu01200.png]

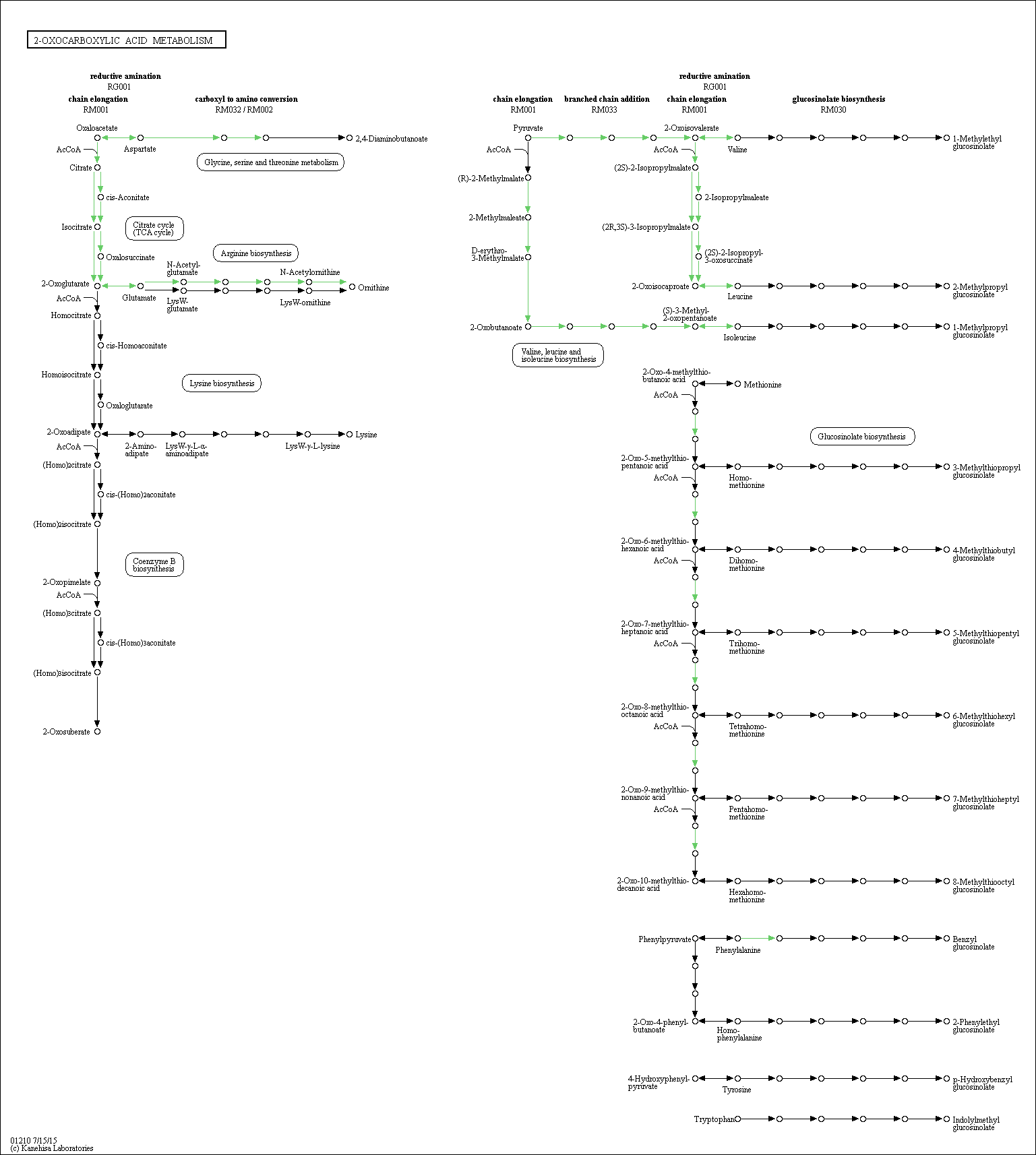

Supplement: S1 File — (ZIP) [file pone.0299259.s004.zip › S1 Zip/src/egu01210.png]

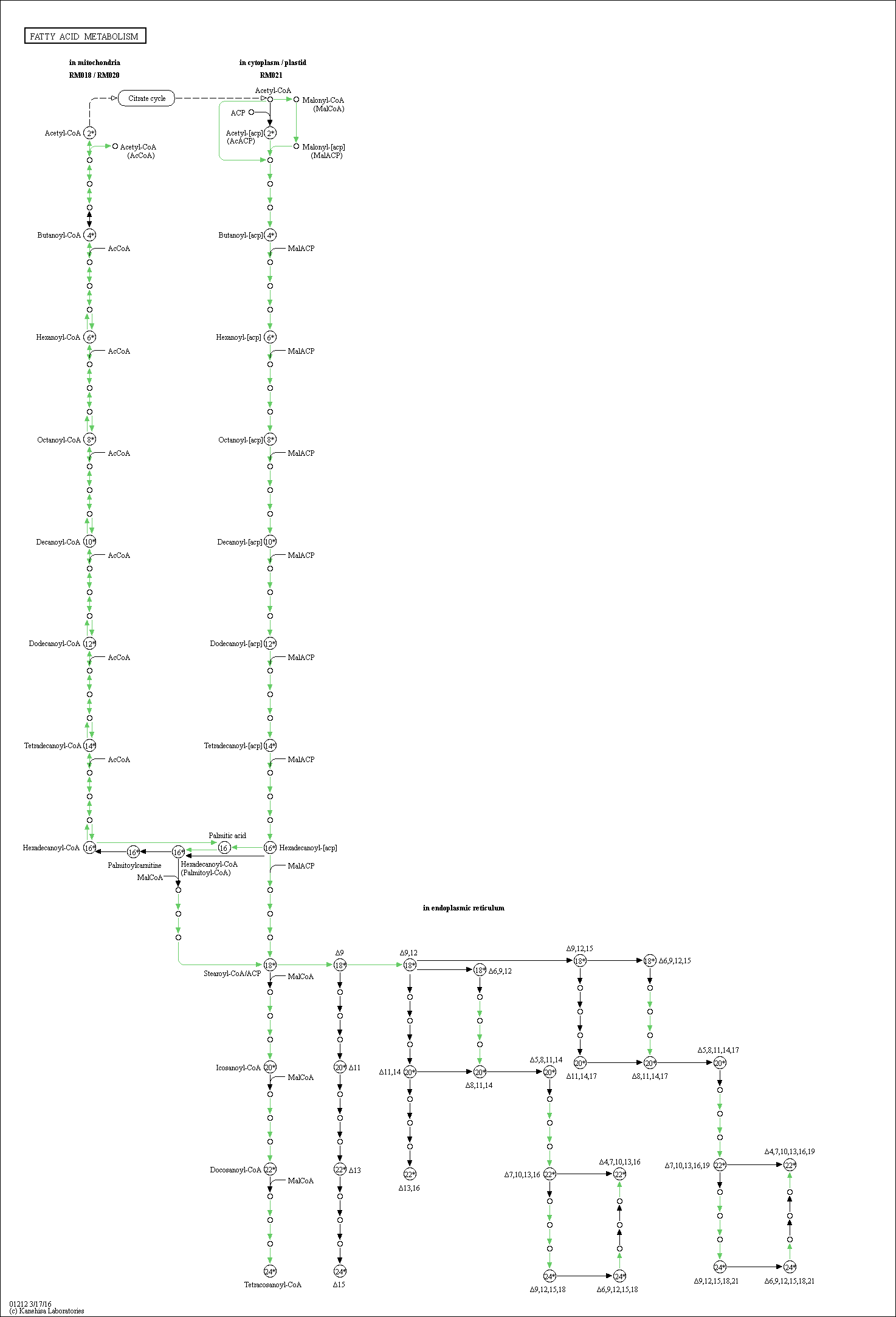

Supplement: S1 File — (ZIP) [file pone.0299259.s004.zip › S1 Zip/src/egu01212.png]

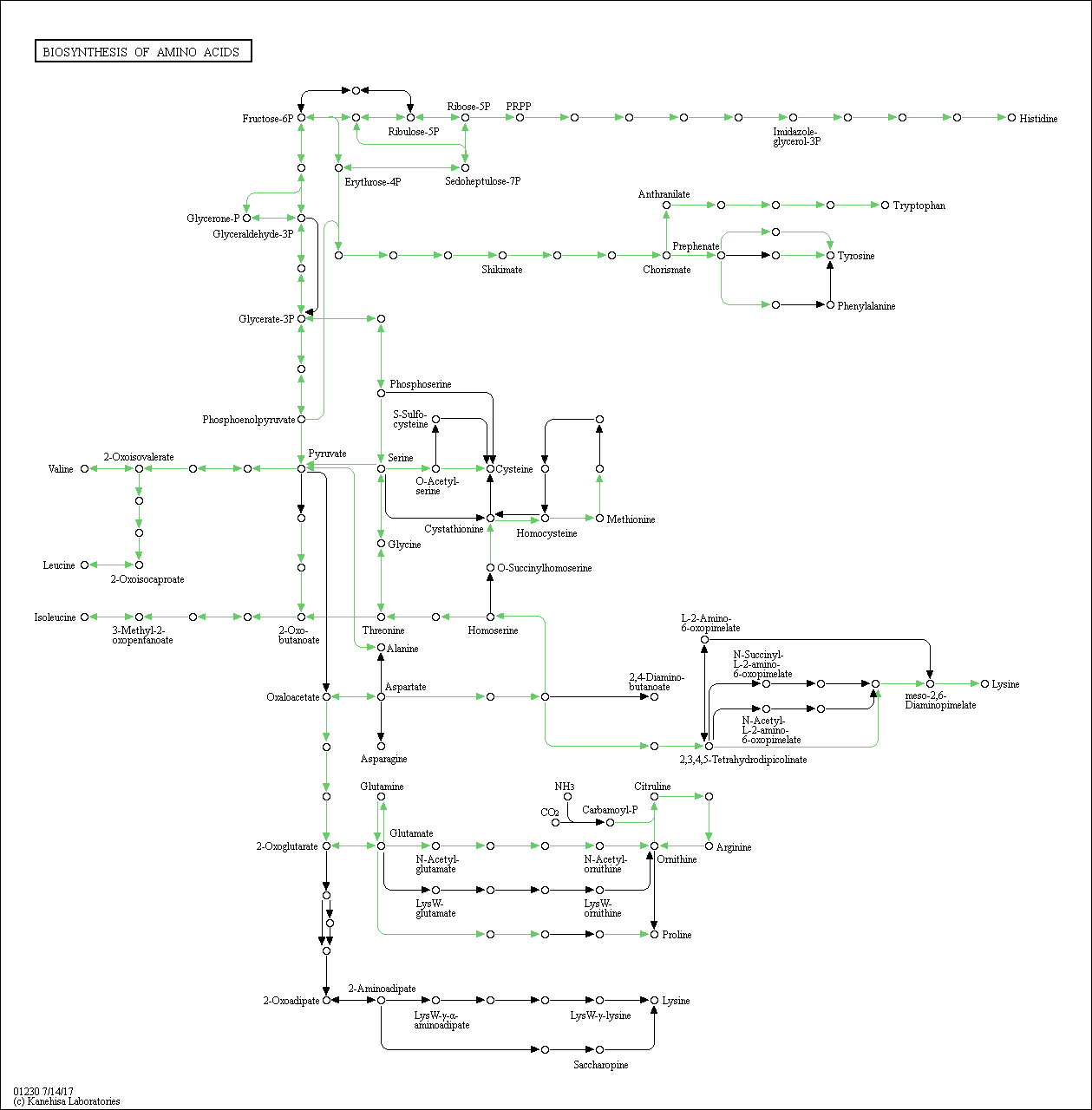

Supplement: S1 File — (ZIP) [file pone.0299259.s004.zip › S1 Zip/src/egu01230.png]

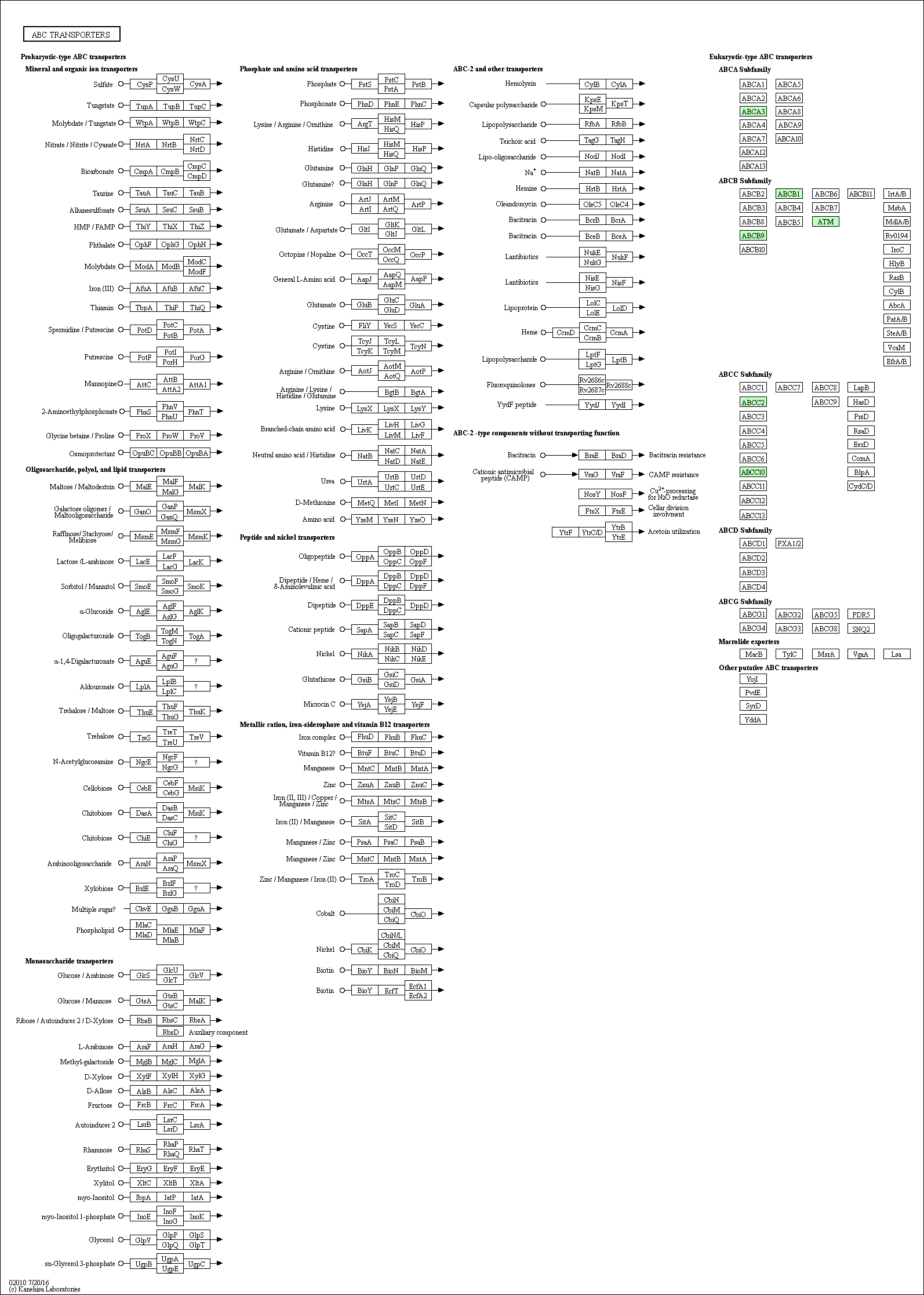

Supplement: S1 File — (ZIP) [file pone.0299259.s004.zip › S1 Zip/src/egu02010.png]

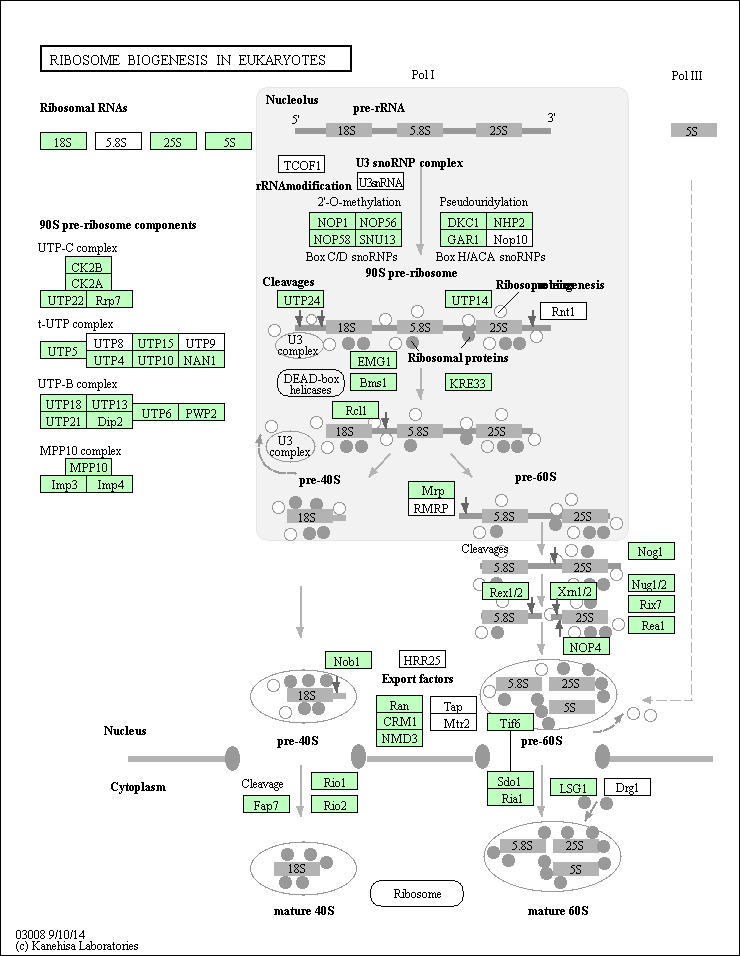

Supplement: S1 File — (ZIP) [file pone.0299259.s004.zip › S1 Zip/src/egu03008.png]

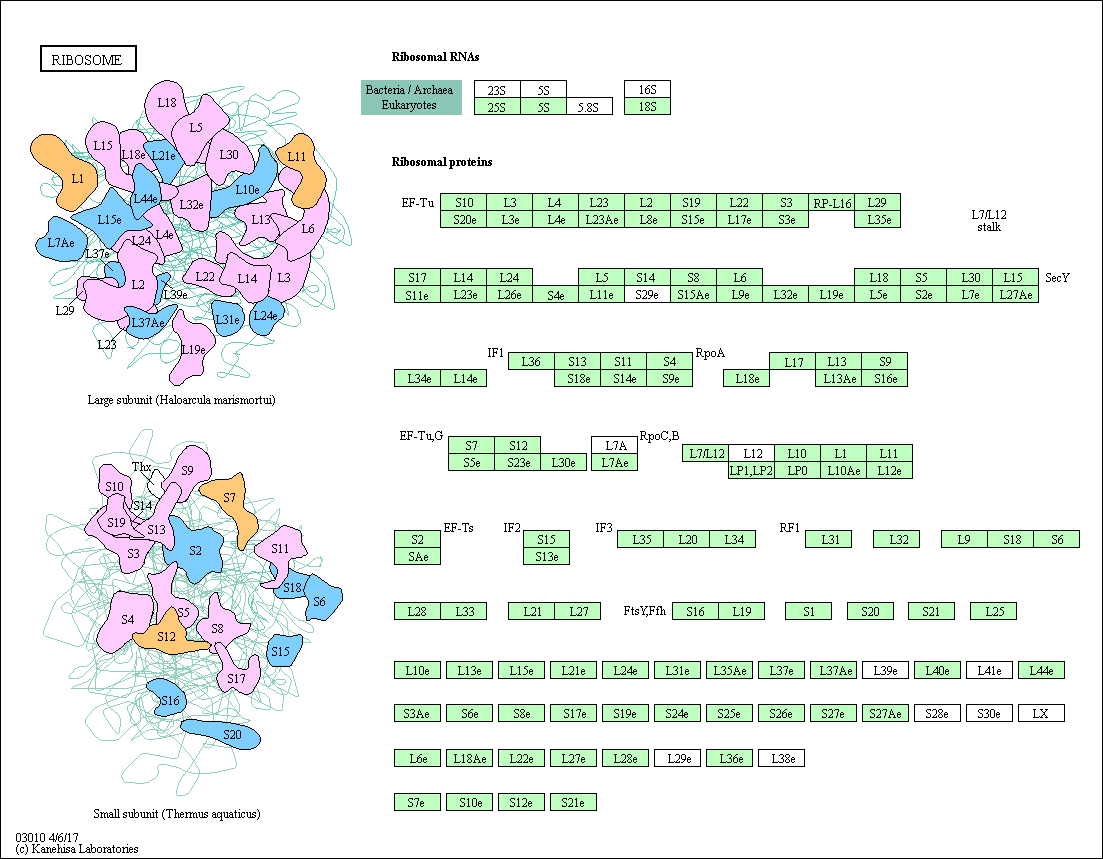

Supplement: S1 File — (ZIP) [file pone.0299259.s004.zip › S1 Zip/src/egu03010.png]

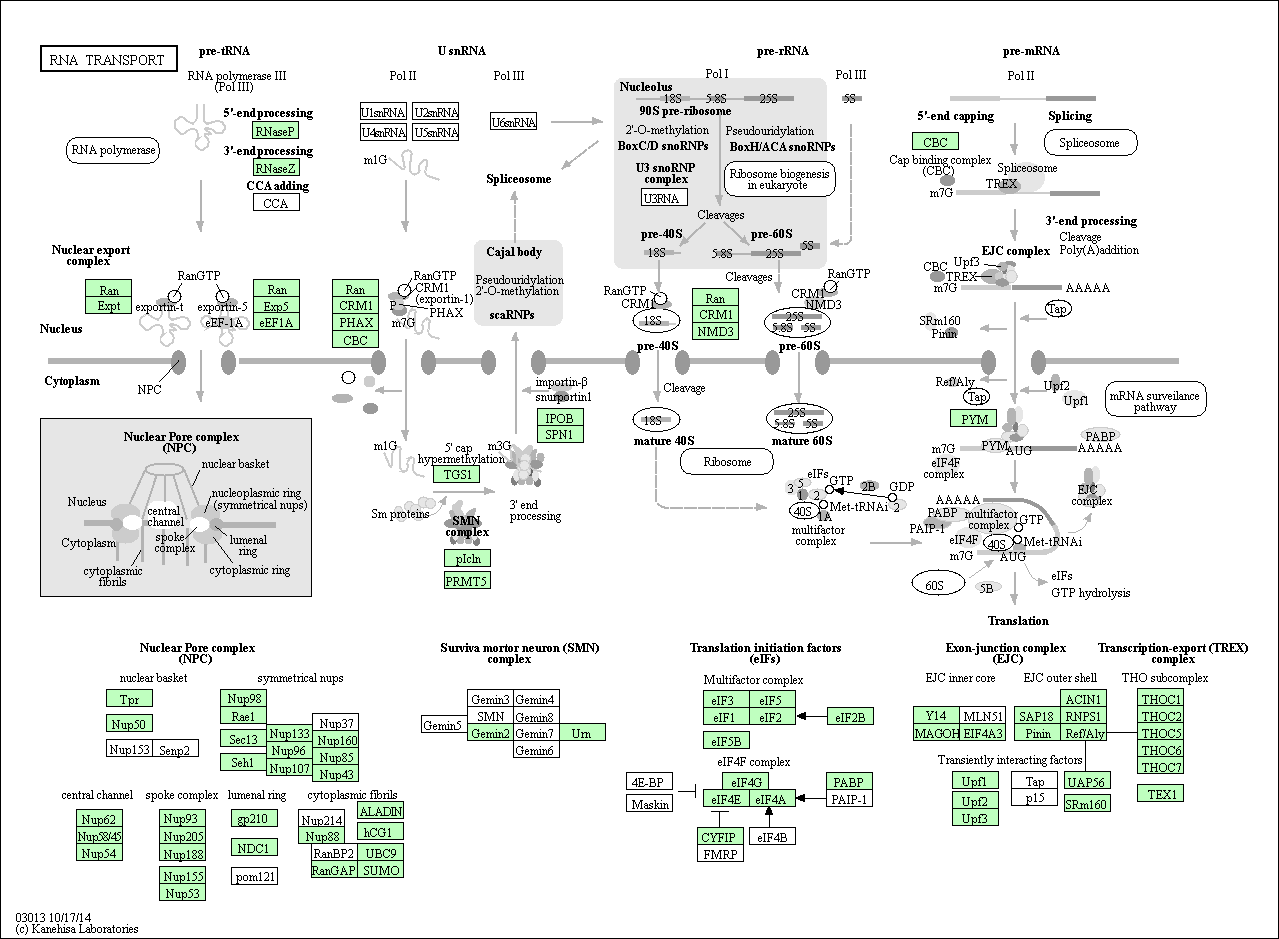

Supplement: S1 File — (ZIP) [file pone.0299259.s004.zip › S1 Zip/src/egu03013.png]

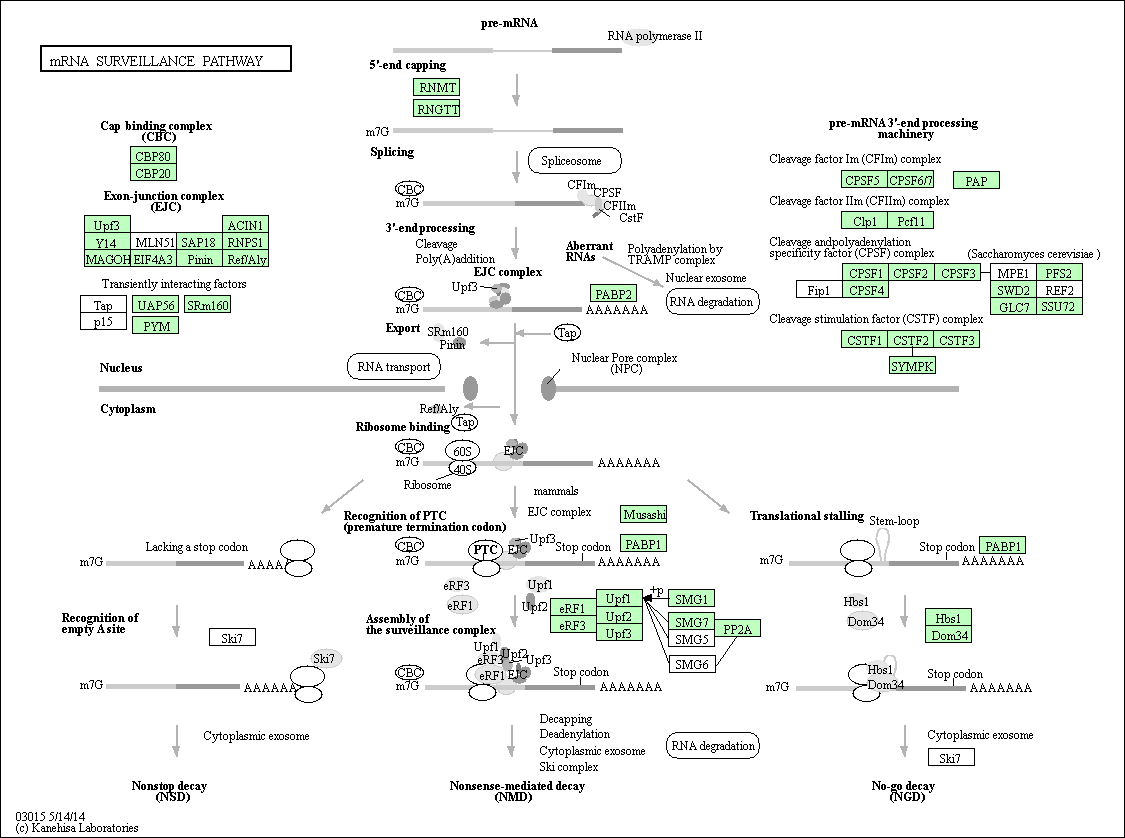

Supplement: S1 File — (ZIP) [file pone.0299259.s004.zip › S1 Zip/src/egu03015.png]

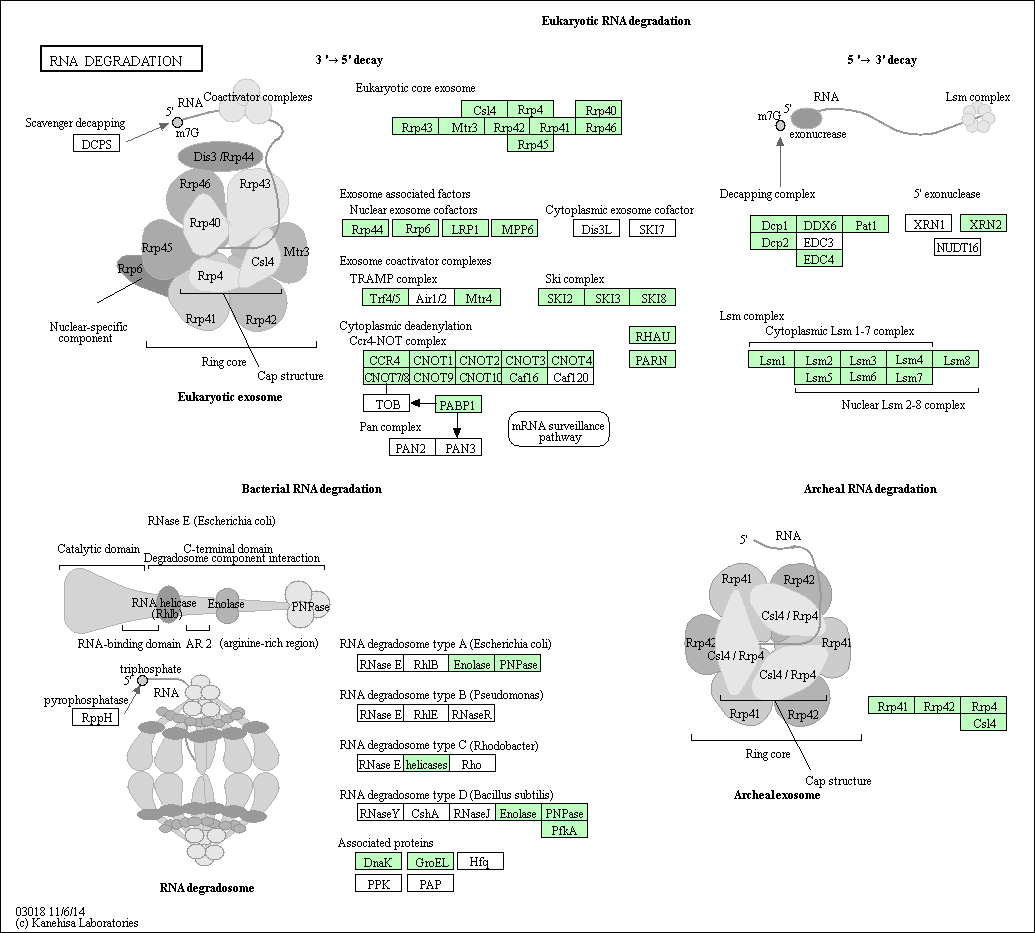

Supplement: S1 File — (ZIP) [file pone.0299259.s004.zip › S1 Zip/src/egu03018.png]
