# Supplementary material for: Key anti-freeze genes and pathways of Lanzhou lily (Lilium davidii, var. unicolor) during the seedling stage
Source: PLoS One. 2024 Mar 21;19(3):e0299259. doi: 10.1371/journal.pone.0299259 (PMC10956819; doi:10.1371/journal.pone.0299259)
Supplement: S2 File — (ZIP) [file pone.0299259.s005.zip › S2 Zip/CvsA_DOWN.html]

Pathway Enrichment

  

# The most enriched pathway terms

Statistic method: hypergeometric test

FDR correction method: Benjamini and Hochberg

| Term | Sample number | Background number | P-value | Corrected P-value | Gene\_id | KEGG\_ID/KO | Entrez ID | Gene name |
| --- | --- | --- | --- | --- | --- | --- | --- | --- |
| Photosynthesis | 27 | 82 | 1.35046395549e-08 | 1.39097787416e-06 | c158839\_g1 c48634\_g1 c84948\_g1 c237057\_g1 c42744\_g1 c71692\_g1 c152881\_g1 c121701\_g1 c237127\_g1 c153649\_g1 c13699\_g1 c76799\_g1 c153193\_g1 c152833\_g1 c106880\_g1 c198283\_g1 c158296\_g1 c143903\_g1 c123692\_g1 c84926\_g1 c149607\_g1 c156755\_g1 c76010\_g1 c140011\_g1 c151822\_g1 c134148\_g1 c131998\_g1 | egu:105033747 egu:105044241 egu:105055143 egu:105049872 egu:105038285 egu:105037273 egu:105044486 egu:105046198 egu:105043122 egu:105046700 egu:105035425 egu:105056235 egu:105053658 egu:105033023 egu:105047072 egu:105046752 egu:105034183 egu:105049540 egu:105038844 egu:105046935 egu:105044080 egu:105056630 egu:105051755 egu:105037935 egu:105054056 egu:105037794 egu:105055013 | 105033747 105044241 105055143 105049872 105038285 105037273 105044486 105046198 105043122 105046700 105035425 105056235 105053658 105033023 105047072 105046752 105034183 105049540 105038844 105046935 105044080 105056630 105051755 105037935 105054056 105037794 105055013 |  |
| Photosynthesis - antenna proteins | 11 | 20 | 6.78671740505e-06 | 0.00034951594636 | c139230\_g1 c115377\_g1 c174985\_g1 c155535\_g1 c211152\_g1 c149445\_g1 c153860\_g1 c152435\_g1 c164577\_g1 c157047\_g1 c149517\_g1 | egu:105053065 egu:105032174 egu:105051572 egu:105032321 egu:105032432 egu:105058558 egu:105058244 egu:105039517 egu:105035084 egu:105058393 egu:105033408 | 105053065 105032174 105051572 105032321 105032432 105058558 105058244 105039517 105035084 105058393 105033408 |  |
| Porphyrin and chlorophyll metabolism | 15 | 50 | 5.53224739532e-05 | 0.00189940493906 | c168519\_g1 c169028\_g1 c185147\_g2 c167947\_g1 c133188\_g1 c165450\_g1 c71809\_g1 c155686\_g1 c167743\_g1 c161205\_g1 c123480\_g1 c157598\_g1 c71670\_g1 c166557\_g2 c166557\_g1 | egu:105040768 egu:105057582 egu:105036097 egu:105054529 egu:105040656 egu:105044798 egu:105049221 egu:105044579 egu:105051026 egu:105052855 egu:105035938 egu:105045229 egu:105037930 egu:105058545 egu:105058545 | 105040768 105057582 105036097 105054529 105040656 105044798 105049221 105044579 105051026 105052855 105035938 105045229 105037930 105058545 105058545 |  |
| Carbon fixation in photosynthetic organisms | 18 | 89 | 0.000740912319491 | 0.0190784922269 | c121900\_g1 c162112\_g2 c164307\_g1 c104889\_g1 c154502\_g4 c198353\_g1 c172966\_g1 c170442\_g1 c168133\_g3 c147541\_g1 c160123\_g1 c104889\_g2 c174574\_g3 c71483\_g1 c158889\_g1 c154303\_g1 c43883\_g1 c159323\_g1 | egu:105042746 egu:105050625 egu:105043976 egu:105034557 egu:105035321 egu:105059611 egu:105042873 egu:105040530 egu:105048437 egu:105057517 egu:105060347 egu:105054530 egu:105054530 egu:105049882 egu:105046280 egu:105032039 egu:105045658 egu:105048107 | 105042746 105050625 105043976 105034557 105035321 105059611 105042873 105040530 105048437 105057517 105060347 105054530 105054530 105049882 105046280 105032039 105045658 105048107 |  |
| Fatty acid elongation | 9 | 30 | 0.00169601595403 | 0.0349379286531 | c166224\_g1 c163278\_g2 c117604\_g1 c134778\_g1 c150017\_g1 c148702\_g1 c134603\_g2 c164323\_g1 c164323\_g2 | egu:105041077 egu:105036065 egu:105052214 egu:105044431 egu:105041077 egu:105044215 egu:105039895 egu:105048315 egu:105047165 | 105041077 105036065 105052214 105044431 105041077 105044215 105039895 105048315 105047165 |  |
| Metabolic pathways | 200 | 2161 | 0.0120458319124 | 0.2024180455 | c121900\_g1 c132497\_g1 c198353\_g1 c133188\_g1 c155535\_g1 c237127\_g1 c162354\_g1 c163865\_g3 c165685\_g1 c162112\_g2 c141672\_g1 c164810\_g1 c153649\_g1 c85645\_g1 c156962\_g1 c164754\_g1 c167282\_g1 c173864\_g1 c152833\_g1 c113371\_g2 c153630\_g1 c149832\_g1 c153334\_g2 c153334\_g1 c168470\_g1 c162392\_g1 c143903\_g1 c123692\_g1 c167954\_g1 c171099\_g1 c171050\_g1 c71809\_g1 c170305\_g2 c171016\_g1 c137214\_g2 c104889\_g2 c104889\_g1 c157598\_g1 c154303\_g1 c162118\_g1 c157047\_g1 c173971\_g3 c185147\_g2 c76721\_g1 c167553\_g1 c165075\_g1 c134148\_g1 c166462\_g1 c127525\_g1 c98584\_g1 c171431\_g1 c164585\_g7 c152294\_g1 c166072\_g1 c174151\_g1 c158088\_g1 c154629\_g1 c156760\_g1 c151091\_g1 c171137\_g1 c152881\_g1 c159323\_g1 c173060\_g2 c134111\_g1 c169028\_g1 c154502\_g4 c133070\_g1 c169731\_g1 c164952\_g6 c163701\_g1 c106880\_g1 c167743\_g1 c165472\_g1 c167963\_g1 c163169\_g1 c155934\_g1 c156209\_g1 c155686\_g1 c164784\_g1 c169453\_g2 c163563\_g1 c71483\_g1 c169665\_g2 c160123\_g1 c174574\_g3 c163317\_g1 c158852\_g2 c166373\_g4 c157388\_g1 c154527\_g1 c149607\_g1 c158576\_g4 c84926\_g1 c156235\_g1 c162681\_g1 c161205\_g1 c174660\_g1 c146091\_g1 c140950\_g1 c174706\_g1 c164577\_g1 c43883\_g1 c48634\_g1 c125057\_g1 c171835\_g1 c158128\_g1 c160412\_g1 c146198\_g1 c160923\_g1 c123071\_g1 c168133\_g3 c166082\_g1 c167947\_g1 c153193\_g1 c170577\_g2 c165906\_g1 c166378\_g2 c172854\_g2 c172165\_g1 c147420\_g1 c164307\_g1 c174800\_g1 c141616\_g1 c159032\_g1 c164707\_g1 c171119\_g1 c162518\_g1 c152279\_g1 c172966\_g1 c161796\_g1 c140011\_g1 c168498\_g1 c165450\_g1 c156755\_g1 c173984\_g2 c132251\_g1 c172504\_g1 c151822\_g1 c170442\_g1 c171401\_g2 c147541\_g1 c157902\_g1 c163496\_g1 c71670\_g1 c237057\_g1 c147195\_g1 c150055\_g1 c101133\_g1 c158839\_g1 c158106\_g1 c174985\_g1 c163642\_g1 c155524\_g1 c164056\_g1 c172570\_g3 c71692\_g1 c158889\_g1 c171265\_g1 c152607\_g1 c121701\_g1 c152482\_g1 c166557\_g1 c150645\_g1 c140061\_g1 c174658\_g2 c159804\_g1 c132118\_g1 c154382\_g1 c163366\_g1 c165079\_g1 c146725\_g1 c163118\_g1 c166557\_g2 c134136\_g1 c159912\_g2 c150729\_g1 c171631\_g8 c171137\_g4 c115377\_g1 c198283\_g1 c168519\_g1 c173904\_g1 c169294\_g2 c159912\_g1 c166358\_g1 c131998\_g1 c123480\_g1 c148171\_g1 c107029\_g1 c147625\_g1 c166887\_g5 c122896\_g1 c76010\_g1 c161893\_g2 c143298\_g1 c156351\_g6 c171508\_g1 c131571\_g1 c168109\_g1 c123366\_g1 | egu:105042746 egu:105059577 egu:105059611 egu:105040656 egu:105032321 egu:105043122 egu:105047598 egu:105048612 egu:105041933 egu:105050625 egu:105054281 egu:105052573 egu:105046700 egu:105049380 egu:105040792 egu:105053059 egu:105050772 egu:105055141 egu:105033023 egu:105034969 egu:105051428 egu:105043809 egu:105050202 egu:105050202 egu:105053765 egu:105041436 egu:105049540 egu:105038844 egu:105047663 egu:105045006 egu:105048201 egu:105049221 egu:105048493 egu:105045448 egu:105038852 egu:105054530 egu:105034557 egu:105045229 egu:105032039 egu:105043957 egu:105058393 egu:105060927 egu:105036097 egu:105032351 egu:105035984 egu:105045599 egu:105037794 egu:105043601 egu:105059896 egu:105056213 egu:105034922 egu:105052170 egu:105055883 egu:105038832 egu:105052838 egu:105057795 egu:105047162 egu:105054024 egu:105034893 egu:105059466 egu:105044486 egu:105048107 egu:105042390 egu:105056640 egu:105057582 egu:105035321 egu:105060694 egu:105039221 egu:105061098 egu:105035926 egu:105047072 egu:105051026 egu:105037657 egu:105059048 egu:105038022 egu:105052647 egu:105044629 egu:105044579 egu:105040461 egu:105059758 egu:105042021 egu:105049882 egu:105044713 egu:105060347 egu:105054530 egu:105056567 egu:105051386 egu:105058765 egu:105052174 egu:105036212 egu:105044080 egu:105055982 egu:105046935 egu:105057764 egu:105039992 egu:105052855 egu:105047063 egu:105041436 egu:105048738 egu:105034542 egu:105035084 egu:105045658 egu:105044241 egu:105054147 egu:105036181 egu:105046284 egu:105035493 egu:105043195 egu:105046469 egu:105057959 egu:105048437 egu:105055812 egu:105054529 egu:105053658 egu:105035222 egu:105042911 egu:105059182 egu:105046898 egu:105039195 egu:105044732 egu:105043976 egu:105055783 egu:105051528 egu:105058894 egu:105035680 egu:105047967 egu:105037896 egu:105051936 egu:105042873 egu:105049214 egu:105037935 egu:105057579 egu:105044798 egu:105056630 egu:105041599 egu:105060929 egu:105052064 egu:105054056 egu:105040530 egu:105032472 egu:105057517 egu:105047380 egu:105059287 egu:105037930 egu:105049872 egu:105043983 egu:105054293 egu:105042952 egu:105033747 egu:105051928 egu:105051572 egu:105039219 egu:105043222 egu:105036454 egu:105032483 egu:105037273 egu:105046280 egu:105032439 egu:105043264 egu:105046198 egu:105046559 egu:105058545 egu:105053413 egu:105032793 egu:105059813 egu:105036836 egu:105034995 egu:105051526 egu:105035642 egu:105058402 egu:105058731 egu:105040940 egu:105058545 egu:105058937 egu:105036971 egu:105055901 egu:105034397 egu:105059466 egu:105032174 egu:105046752 egu:105040768 egu:105049267 egu:105034723 egu:105039425 egu:105046827 egu:105055013 egu:105035938 egu:105039544 egu:105039713 egu:105040827 egu:105042425 egu:105046147 egu:105051755 egu:105035544 egu:105041725 egu:105053174 egu:105052170 egu:105034341 egu:105057479 egu:105060320 | 105042746 105059577 105059611 105040656 105032321 105043122 105047598 105048612 105041933 105050625 105054281 105052573 105046700 105049380 105040792 105053059 105050772 105055141 105033023 105034969 105051428 105043809 105050202 105050202 105053765 105041436 105049540 105038844 105047663 105045006 105048201 105049221 105048493 105045448 105038852 105054530 105034557 105045229 105032039 105043957 105058393 105060927 105036097 105032351 105035984 105045599 105037794 105043601 105059896 105056213 105034922 105052170 105055883 105038832 105052838 105057795 105047162 105054024 105034893 105059466 105044486 105048107 105042390 105056640 105057582 105035321 105060694 105039221 105061098 105035926 105047072 105051026 105037657 105059048 105038022 105052647 105044629 105044579 105040461 105059758 105042021 105049882 105044713 105060347 105054530 105056567 105051386 105058765 105052174 105036212 105044080 105055982 105046935 105057764 105039992 105052855 105047063 105041436 105048738 105034542 105035084 105045658 105044241 105054147 105036181 105046284 105035493 105043195 105046469 105057959 105048437 105055812 105054529 105053658 105035222 105042911 105059182 105046898 105039195 105044732 105043976 105055783 105051528 105058894 105035680 105047967 105037896 105051936 105042873 105049214 105037935 105057579 105044798 105056630 105041599 105060929 105052064 105054056 105040530 105032472 105057517 105047380 105059287 105037930 105049872 105043983 105054293 105042952 105033747 105051928 105051572 105039219 105043222 105036454 105032483 105037273 105046280 105032439 105043264 105046198 105046559 105058545 105053413 105032793 105059813 105036836 105034995 105051526 105035642 105058402 105058731 105040940 105058545 105058937 105036971 105055901 105034397 105059466 105032174 105046752 105040768 105049267 105034723 105039425 105046827 105055013 105035938 105039544 105039713 105040827 105042425 105046147 105051755 105035544 105041725 105053174 105052170 105034341 105057479 105060320 |  |
| Biosynthesis of secondary metabolites | 116 | 1184 | 0.0137565661991 | 0.2024180455 | c132497\_g1 c198353\_g1 c133188\_g1 c162354\_g1 c165685\_g1 c148702\_g1 c141672\_g1 c167963\_g1 c85645\_g1 c156962\_g1 c153630\_g1 c134778\_g1 c167011\_g1 c113371\_g2 c166224\_g1 c168470\_g1 c150017\_g1 c162392\_g1 c171099\_g1 c171050\_g1 c71809\_g1 c170305\_g2 c171016\_g1 c137214\_g2 c104889\_g2 c104889\_g1 c157598\_g1 c154303\_g1 c162118\_g1 c173971\_g3 c185147\_g2 c167553\_g1 c163278\_g2 c165075\_g1 c127525\_g1 c152294\_g1 c166072\_g1 c174151\_g1 c154629\_g1 c159323\_g1 c173060\_g2 c169028\_g1 c154502\_g4 c133070\_g1 c164952\_g6 c163288\_g1 c160710\_g1 c163701\_g1 c167743\_g1 c165472\_g1 c163169\_g1 c155934\_g1 c156209\_g1 c155686\_g1 c164784\_g1 c117604\_g1 c169453\_g2 c71483\_g1 c140950\_g1 c174574\_g3 c158852\_g2 c157388\_g1 c158576\_g4 c156235\_g1 c161205\_g1 c159912\_g2 c146091\_g1 c174706\_g1 c43883\_g1 c125057\_g1 c158128\_g1 c146198\_g1 c160923\_g1 c167947\_g1 c164323\_g1 c159032\_g1 c162518\_g1 c166734\_g1 c168519\_g1 c165450\_g1 c132251\_g1 c174739\_g1 c171401\_g2 c147541\_g1 c157902\_g1 c163496\_g1 c71670\_g1 c101133\_g1 c163642\_g1 c155524\_g1 c164056\_g1 c172570\_g3 c171265\_g1 c152607\_g1 c166557\_g2 c166557\_g1 c150645\_g1 c140061\_g1 c162112\_g2 c159804\_g1 c154382\_g1 c163118\_g1 c134136\_g1 c134111\_g1 c173904\_g1 c169294\_g2 c159912\_g1 c166358\_g1 c123480\_g1 c134603\_g2 c147625\_g1 c164323\_g2 c166887\_g5 c122896\_g1 c12992\_g1 c131571\_g1 | egu:105059577 egu:105059611 egu:105040656 egu:105047598 egu:105041933 egu:105044215 egu:105054281 egu:105059048 egu:105049380 egu:105040792 egu:105051428 egu:105044431 egu:105036026 egu:105034969 egu:105041077 egu:105053765 egu:105041077 egu:105041436 egu:105045006 egu:105048201 egu:105049221 egu:105048493 egu:105045448 egu:105038852 egu:105054530 egu:105034557 egu:105045229 egu:105032039 egu:105043957 egu:105060927 egu:105036097 egu:105035984 egu:105036065 egu:105045599 egu:105059896 egu:105055883 egu:105038832 egu:105052838 egu:105047162 egu:105048107 egu:105042390 egu:105057582 egu:105035321 egu:105060694 egu:105061098 egu:105046233 egu:105034750 egu:105035926 egu:105051026 egu:105037657 egu:105038022 egu:105052647 egu:105044629 egu:105044579 egu:105040461 egu:105052214 egu:105059758 egu:105049882 egu:105048738 egu:105054530 egu:105051386 egu:105052174 egu:105055982 egu:105057764 egu:105052855 egu:105036971 egu:105041436 egu:105034542 egu:105045658 egu:105054147 egu:105046284 egu:105043195 egu:105046469 egu:105054529 egu:105048315 egu:105058894 egu:105037896 egu:105034750 egu:105040768 egu:105044798 egu:105060929 egu:105034750 egu:105032472 egu:105057517 egu:105047380 egu:105059287 egu:105037930 egu:105042952 egu:105039219 egu:105043222 egu:105036454 egu:105032483 egu:105032439 egu:105043264 egu:105058545 egu:105058545 egu:105053413 egu:105032793 egu:105050625 egu:105036836 egu:105051526 egu:105040940 egu:105058937 egu:105056640 egu:105049267 egu:105034723 egu:105039425 egu:105046827 egu:105035938 egu:105039895 egu:105040827 egu:105047165 egu:105042425 egu:105046147 egu:105052307 egu:105034341 | 105059577 105059611 105040656 105047598 105041933 105044215 105054281 105059048 105049380 105040792 105051428 105044431 105036026 105034969 105041077 105053765 105041077 105041436 105045006 105048201 105049221 105048493 105045448 105038852 105054530 105034557 105045229 105032039 105043957 105060927 105036097 105035984 105036065 105045599 105059896 105055883 105038832 105052838 105047162 105048107 105042390 105057582 105035321 105060694 105061098 105046233 105034750 105035926 105051026 105037657 105038022 105052647 105044629 105044579 105040461 105052214 105059758 105049882 105048738 105054530 105051386 105052174 105055982 105057764 105052855 105036971 105041436 105034542 105045658 105054147 105046284 105043195 105046469 105054529 105048315 105058894 105037896 105034750 105040768 105044798 105060929 105034750 105032472 105057517 105047380 105059287 105037930 105042952 105039219 105043222 105036454 105032483 105032439 105043264 105058545 105058545 105053413 105032793 105050625 105036836 105051526 105040940 105058937 105056640 105049267 105034723 105039425 105046827 105035938 105039895 105040827 105047165 105042425 105046147 105052307 105034341 |  |
| Arginine biosynthesis | 8 | 40 | 0.0223794254504 | 0.288135102674 | c174151\_g1 c132251\_g1 c146725\_g1 c158088\_g1 c71483\_g1 c160412\_g1 c152607\_g1 c159323\_g1 | egu:105052838 egu:105060929 egu:105058731 egu:105057795 egu:105049882 egu:105035493 egu:105043264 egu:105048107 | 105052838 105060929 105058731 105057795 105049882 105035493 105043264 105048107 |  |
| Carbon metabolism | 34 | 322 | 0.0684301040887 | 0.738841872405 | c121900\_g1 c104889\_g1 c198353\_g1 c163642\_g1 c158889\_g1 c169294\_g2 c159323\_g1 c140061\_g1 c153630\_g1 c162112\_g2 c154502\_g4 c133070\_g1 c146725\_g1 c168133\_g3 c163701\_g1 c113371\_g2 c164307\_g1 c167954\_g1 c162392\_g1 c164784\_g1 c171050\_g1 c172966\_g1 c160123\_g1 c104889\_g2 c174574\_g3 c154303\_g1 c147625\_g1 c170442\_g1 c147541\_g1 c157902\_g1 c146091\_g1 c163496\_g1 c71483\_g1 c43883\_g1 | egu:105042746 egu:105034557 egu:105059611 egu:105039219 egu:105046280 egu:105034723 egu:105048107 egu:105032793 egu:105051428 egu:105050625 egu:105035321 egu:105060694 egu:105058731 egu:105048437 egu:105035926 egu:105034969 egu:105043976 egu:105047663 egu:105041436 egu:105040461 egu:105048201 egu:105042873 egu:105060347 egu:105054530 egu:105054530 egu:105032039 egu:105040827 egu:105040530 egu:105057517 egu:105047380 egu:105041436 egu:105059287 egu:105049882 egu:105045658 | 105042746 105034557 105059611 105039219 105046280 105034723 105048107 105032793 105051428 105050625 105035321 105060694 105058731 105048437 105035926 105034969 105043976 105047663 105041436 105040461 105048201 105042873 105060347 105054530 105054530 105032039 105040827 105040530 105057517 105047380 105041436 105059287 105049882 105045658 |  |
| Oxidative phosphorylation | 19 | 163 | 0.0757046826492 | 0.738841872405 | c123071\_g1 c166082\_g1 c158106\_g1 c143903\_g1 c121701\_g1 c158605\_g1 c172080\_g4 c127506\_g1 c172680\_g2 c166378\_g2 c161726\_g1 c169665\_g2 c152833\_g1 c174660\_g1 c152482\_g1 c147195\_g1 c150055\_g1 c149832\_g1 c150729\_g1 | egu:105057959 egu:105055812 egu:105051928 egu:105049540 egu:105046198 egu:105060092 egu:105039662 egu:105043730 egu:105052943 egu:105059182 egu:105039277 egu:105044713 egu:105033023 egu:105047063 egu:105046559 egu:105043983 egu:105054293 egu:105043809 egu:105055901 | 105057959 105055812 105051928 105049540 105046198 105060092 105039662 105043730 105052943 105059182 105039277 105044713 105033023 105047063 105046559 105043983 105054293 105043809 105055901 |  |
| Ubiquinone and other terpenoid-quinone biosynthesis | 6 | 36 | 0.0843333234251 | 0.738841872405 | c164952\_g6 c163118\_g1 c154629\_g1 c165685\_g1 c159709\_g1 c173971\_g3 | egu:105061098 egu:105040940 egu:105047162 egu:105041933 egu:105046456 egu:105060927 | 105061098 105040940 105047162 105041933 105046456 105060927 |  |
| Lipoic acid metabolism | 2 | 5 | 0.0882968029751 | 0.738841872405 | c164707\_g1 c163865\_g3 | egu:105035680 egu:105048612 | 105035680 105048612 |  |
| Plant hormone signal transduction | 30 | 288 | 0.0932518868084 | 0.738841872405 | c166932\_g3 c173216\_g1 c163360\_g1 c164451\_g1 c153395\_g1 c157547\_g1 c146802\_g3 c156282\_g1 c141225\_g1 c163658\_g1 c116329\_g1 c153105\_g1 c155948\_g3 c146374\_g1 c169534\_g1 c93769\_g1 c146567\_g1 c78828\_g1 c155948\_g1 c141042\_g1 c167725\_g1 c167725\_g2 c156359\_g1 c133669\_g1 c164642\_g1 c142840\_g2 c140543\_g1 c164908\_g1 c173307\_g1 c173362\_g3 | egu:105040625 egu:105032733 egu:105045549 egu:105057151 egu:105043524 egu:105042455 egu:105052748 egu:105057151 egu:105039360 egu:105041587 egu:105042452 egu:105042180 egu:105050174 egu:105046249 egu:105033977 egu:105042455 egu:105060907 egu:105038391 egu:105050174 egu:105040857 egu:105036306 egu:105036306 egu:105039360 egu:105035706 egu:105052996 egu:105055597 egu:105044079 egu:105034824 egu:105060907 egu:105033626 | 105040625 105032733 105045549 105057151 105043524 105042455 105052748 105057151 105039360 105041587 105042452 105042180 105050174 105046249 105033977 105042455 105060907 105038391 105050174 105040857 105036306 105036306 105039360 105035706 105052996 105055597 105044079 105034824 105060907 105033626 |  |
| Nitrogen metabolism | 6 | 38 | 0.100546046238 | 0.739731625892 | c142597\_g1 c115915\_g1 c185512\_g1 c146725\_g1 c158088\_g1 c160412\_g1 | egu:105052122 egu:105048068 egu:105033813 egu:105058731 egu:105057795 egu:105035493 | 105052122 105048068 105033813 105058731 105057795 105035493 |  |
| Fatty acid biosynthesis | 8 | 59 | 0.116285466737 | 0.798477616048 | c153334\_g2 c153334\_g1 c104546\_g1 c163366\_g1 c152279\_g1 c164754\_g1 c169731\_g1 c147420\_g1 | egu:105050202 egu:105050202 egu:105049664 egu:105035642 egu:105051936 egu:105053059 egu:105039221 egu:105044732 | 105050202 105050202 105049664 105035642 105051936 105053059 105039221 105044732 |  |
| Glycolysis / Gluconeogenesis | 17 | 154 | 0.124035357833 | 0.798477616048 | c162112\_g2 c85645\_g1 c154502\_g4 c198353\_g1 c133070\_g1 c163642\_g1 c163496\_g1 c171050\_g1 c104889\_g1 c170305\_g2 c146091\_g1 c174574\_g3 c113371\_g2 c162392\_g1 c43883\_g1 c169294\_g2 c104889\_g2 | egu:105050625 egu:105049380 egu:105035321 egu:105059611 egu:105060694 egu:105039219 egu:105059287 egu:105048201 egu:105034557 egu:105048493 egu:105041436 egu:105054530 egu:105034969 egu:105041436 egu:105045658 egu:105034723 egu:105054530 | 105050625 105049380 105035321 105059611 105060694 105039219 105059287 105048201 105034557 105048493 105041436 105054530 105034969 105041436 105045658 105034723 105054530 |  |
| Glyoxylate and dicarboxylate metabolism | 10 | 83 | 0.143497139996 | 0.866239383369 | c153630\_g1 c132497\_g1 c167954\_g1 c158088\_g1 c168133\_g3 c71483\_g1 c157902\_g1 c163701\_g1 c160412\_g1 c147625\_g1 | egu:105051428 egu:105059577 egu:105047663 egu:105057795 egu:105048437 egu:105049882 egu:105047380 egu:105035926 egu:105035493 egu:105040827 | 105051428 105059577 105047663 105057795 105048437 105049882 105047380 105035926 105035493 105040827 |  |
| Fatty acid metabolism | 10 | 85 | 0.158131910408 | 0.866239383369 | c153334\_g2 c153334\_g1 c163366\_g1 c152279\_g1 c117604\_g1 c164754\_g1 c137480\_g1 c169731\_g1 c147420\_g1 c134778\_g1 | egu:105050202 egu:105050202 egu:105035642 egu:105051936 egu:105052214 egu:105053059 egu:105061227 egu:105039221 egu:105044732 egu:105044431 | 105050202 105050202 105035642 105051936 105052214 105053059 105061227 105039221 105044732 105044431 |  |
| Ribosome | 36 | 377 | 0.159791730913 | 0.866239383369 | c142600\_g1 c27497\_g1 c163280\_g1 c156427\_g1 c157065\_g1 c158737\_g2 c151905\_g1 c173687\_g6 c155204\_g1 c141711\_g1 c172832\_g1 c145214\_g1 c106647\_g1 c143129\_g1 c160468\_g1 c224200\_g1 c94348\_g1 c116569\_g1 c160112\_g1 c141240\_g1 c237068\_g1 c168022\_g1 c178004\_g1 c146729\_g1 c138659\_g1 c161109\_g1 c132904\_g1 c159369\_g1 c48496\_g1 c159746\_g1 c150484\_g1 c140525\_g1 c132540\_g1 c134374\_g1 c156664\_g1 c143884\_g1 | egu:105047611 egu:105052661 egu:105052800 egu:105059074 egu:105034390 egu:105032797 egu:105035316 egu:105044731 egu:105032412 egu:105048529 egu:105045120 egu:105034395 egu:105053938 egu:105056818 egu:105041074 egu:105059189 egu:105048206 egu:105046043 egu:105041287 egu:105048988 egu:105032842 egu:105044673 egu:105032412 egu:105058445 egu:105041319 egu:105060039 egu:105036502 egu:105061575 egu:105033340 egu:105059802 egu:105040763 egu:105042443 egu:105034754 egu:105040137 egu:105057725 egu:105043313 | 105047611 105052661 105052800 105059074 105034390 105032797 105035316 105044731 105032412 105048529 105045120 105034395 105053938 105056818 105041074 105059189 105048206 105046043 105041287 105048988 105032842 105044673 105032412 105058445 105041319 105060039 105036502 105061575 105033340 105059802 105040763 105042443 105034754 105040137 105057725 105043313 |  |
| Biotin metabolism | 4 | 26 | 0.174658281879 | 0.899490151677 | c153334\_g2 c152279\_g1 c153334\_g1 c164754\_g1 | egu:105050202 egu:105051936 egu:105050202 egu:105053059 | 105050202 105051936 105050202 105053059 |  |
| Glycosaminoglycan degradation | 3 | 18 | 0.196841304581 | 0.901291216811 | c151091\_g1 c168109\_g1 c168498\_g1 | egu:105034893 egu:105057479 egu:105057579 | 105034893 105057479 105057579 |  |
| Cutin, suberine and wax biosynthesis | 4 | 28 | 0.205154964778 | 0.901291216811 | c140465\_g1 c103250\_g1 c145863\_g1 c165906\_g1 | egu:105036883 egu:105061386 egu:105047658 egu:105042911 | 105036883 105061386 105047658 105042911 |  |
| Tyrosine metabolism | 5 | 39 | 0.218989142381 | 0.901291216811 | c146198\_g1 c162392\_g1 c146091\_g1 c163118\_g1 c159323\_g1 | egu:105043195 egu:105041436 egu:105041436 egu:105040940 egu:105048107 | 105043195 105041436 105041436 105040940 105048107 |  |
| ABC transporters | 4 | 29 | 0.220948048504 | 0.901291216811 | c169285\_g2 c169285\_g1 c163163\_g1 c173719\_g3 | egu:105055560 egu:105059124 egu:105034865 egu:105052956 | 105055560 105059124 105034865 105052956 |  |
| Citrate cycle (TCA cycle) | 8 | 71 | 0.222068168508 | 0.901291216811 | c163496\_g1 c174574\_g3 c171099\_g1 c169453\_g2 c104889\_g2 c104889\_g1 c113371\_g2 c169294\_g2 | egu:105059287 egu:105054530 egu:105045006 egu:105059758 egu:105054530 egu:105034557 egu:105034969 egu:105034723 | 105059287 105054530 105045006 105059758 105054530 105034557 105034969 105034723 |  |
| Terpenoid backbone biosynthesis | 7 | 61 | 0.230365204407 | 0.901291216811 | c122896\_g1 c165075\_g1 c171401\_g2 c159912\_g2 c159912\_g1 c166557\_g2 c166557\_g1 | egu:105046147 egu:105045599 egu:105032472 egu:105036971 egu:105039425 egu:105058545 egu:105058545 | 105046147 105045599 105032472 105036971 105039425 105058545 105058545 |  |
| Stilbenoid, diarylheptanoid and gingerol biosynthesis | 3 | 20 | 0.236260804407 | 0.901291216811 | c171265\_g1 c168470\_g1 c171016\_g1 | egu:105032439 egu:105053765 egu:105045448 | 105032439 105053765 105045448 |  |
| Other glycan degradation | 3 | 21 | 0.256457195166 | 0.910865210416 | c151091\_g1 c172934\_g1 c173576\_g2 | egu:105034893 egu:105056147 egu:105044052 | 105034893 105056147 105044052 |  |
| Isoquinoline alkaloid biosynthesis | 3 | 21 | 0.256457195166 | 0.910865210416 | c146198\_g1 c159323\_g1 c163118\_g1 | egu:105043195 egu:105048107 egu:105040940 | 105043195 105048107 105040940 |  |
| Riboflavin metabolism | 2 | 12 | 0.274632627377 | 0.914302137024 | c154382\_g1 c162354\_g1 | egu:105051526 egu:105047598 | 105051526 105047598 |  |
| Regulation of autophagy | 5 | 43 | 0.275178313085 | 0.914302137024 | c146711\_g1 c140039\_g1 c145728\_g1 c167169\_g2 c173419\_g5 | egu:105058890 egu:105051198 egu:105058890 egu:105061507 egu:105058890 | 105058890 105051198 105058890 105061507 105058890 |  |
| Aminoacyl-tRNA biosynthesis | 9 | 94 | 0.343671449292 | 0.999982120026 | c169070\_g1 c170522\_g1 c132118\_g1 c163858\_g1 c161964\_g1 c161205\_g1 c163317\_g1 c174800\_g1 c162019\_g1 | egu:105041858 egu:105041146 egu:105034995 egu:105055562 egu:105039813 egu:105052855 egu:105056567 egu:105055783 egu:105050113 | 105041858 105041146 105034995 105055562 105039813 105052855 105056567 105055783 105050113 |  |
| Glycosphingolipid biosynthesis - ganglio series | 1 | 5 | 0.367152246023 | 0.999982120026 | c151091\_g1 | egu:105034893 | 105034893 |  |
| Linoleic acid metabolism | 2 | 16 | 0.385024938599 | 0.999982120026 | c158852\_g2 c140950\_g1 | egu:105051386 egu:105048738 | 105051386 105048738 |  |
| Sesquiterpenoid and triterpenoid biosynthesis | 1 | 6 | 0.413628153197 | 0.999982120026 | c152294\_g1 | egu:105055883 | 105055883 |  |
| Diterpenoid biosynthesis | 3 | 29 | 0.420452840275 | 0.999982120026 | c159595\_g1 c142425\_g1 c155524\_g1 | egu:105032920 egu:105032920 egu:105043222 | 105032920 105032920 105043222 |  |
| Glycerolipid metabolism | 7 | 77 | 0.421354094997 | 0.999982120026 | c167963\_g1 c163169\_g1 c158576\_g4 c122861\_g1 c172504\_g1 c148171\_g1 c162118\_g1 | egu:105059048 egu:105038022 egu:105055982 egu:105041806 egu:105052064 egu:105039544 egu:105043957 | 105059048 105038022 105055982 105041806 105052064 105039544 105043957 |  |
| Tropane, piperidine and pyridine alkaloid biosynthesis | 2 | 18 | 0.437561765452 | 0.999982120026 | c159323\_g1 c163118\_g1 | egu:105048107 egu:105040940 | 105048107 105040940 |  |
| Flavonoid biosynthesis | 5 | 54 | 0.438038016529 | 0.999982120026 | c171265\_g1 c141672\_g1 c168470\_g1 c171016\_g1 c167553\_g1 | egu:105032439 egu:105054281 egu:105053765 egu:105045448 egu:105035984 | 105032439 105054281 105053765 105045448 105035984 |  |
| Pyruvate metabolism | 9 | 104 | 0.450192436822 | 0.999982120026 | c104889\_g1 c163496\_g1 c171050\_g1 c172966\_g1 c170442\_g1 c104889\_g2 c174574\_g3 c113371\_g2 c169294\_g2 | egu:105034557 egu:105059287 egu:105048201 egu:105042873 egu:105040530 egu:105054530 egu:105054530 egu:105034969 egu:105034723 | 105034557 105059287 105048201 105042873 105040530 105054530 105054530 105034969 105034723 |  |
| Pentose phosphate pathway | 7 | 80 | 0.457972037436 | 0.999982120026 | c85645\_g1 c154502\_g4 c133070\_g1 c170305\_g2 c147541\_g1 c154303\_g1 c162112\_g2 | egu:105049380 egu:105035321 egu:105060694 egu:105048493 egu:105057517 egu:105032039 egu:105050625 | 105049380 105035321 105060694 105048493 105057517 105032039 105050625 |  |
| Phenylalanine, tyrosine and tryptophan biosynthesis | 5 | 56 | 0.46729491676 | 0.999982120026 | c164056\_g1 c125057\_g1 c159323\_g1 c163118\_g1 c166358\_g1 | egu:105036454 egu:105054147 egu:105048107 egu:105040940 egu:105046827 | 105036454 105054147 105048107 105040940 105046827 |  |
| Alanine, aspartate and glutamate metabolism | 5 | 57 | 0.481749209467 | 0.999982120026 | c160412\_g1 c71483\_g1 c146725\_g1 c159323\_g1 c158088\_g1 | egu:105035493 egu:105049882 egu:105058731 egu:105048107 egu:105057795 | 105035493 105049882 105058731 105048107 105057795 |  |
| Brassinosteroid biosynthesis | 2 | 20 | 0.487554640342 | 0.999982120026 | c154790\_g1 c163288\_g1 | egu:105033219 egu:105046233 | 105033219 105046233 |  |
| alpha-Linolenic acid metabolism | 5 | 58 | 0.496066696677 | 0.999982120026 | c158852\_g2 c140950\_g1 c172570\_g3 c154400\_g1 c167011\_g1 | egu:105051386 egu:105048738 egu:105032483 egu:105056286 egu:105036026 | 105051386 105048738 105032483 105056286 105036026 |  |
| Arginine and proline metabolism | 5 | 58 | 0.496066696677 | 0.999982120026 | c98584\_g1 c170577\_g2 c152607\_g1 c159323\_g1 c163847\_g1 | egu:105056213 egu:105035222 egu:105043264 egu:105048107 egu:105032618 | 105056213 105035222 105043264 105048107 105032618 |  |
| C5-Branched dibasic acid metabolism | 1 | 8 | 0.496600434777 | 0.999982120026 | c156235\_g1 | egu:105057764 | 105057764 |  |
| Carotenoid biosynthesis | 3 | 34 | 0.517183206103 | 0.999982120026 | c137214\_g2 c166072\_g1 c159032\_g1 | egu:105038852 egu:105038832 egu:105058894 | 105038852 105038832 105058894 |  |
| 2-Oxocarboxylic acid metabolism | 5 | 60 | 0.524235028972 | 0.999982120026 | c71483\_g1 c156235\_g1 c159323\_g1 c132251\_g1 c174151\_g1 | egu:105049882 egu:105057764 egu:105048107 egu:105060929 egu:105052838 | 105049882 105057764 105048107 105060929 105052838 |  |
| Circadian rhythm - plant | 5 | 60 | 0.524235028972 | 0.999982120026 | c161583\_g1 c150867\_g1 c170910\_g1 c113253\_g1 c150398\_g4 | egu:105040294 egu:105054949 egu:105044676 egu:105054640 egu:105054641 | 105040294 105054949 105044676 105054640 105054641 |  |
| Fatty acid degradation | 4 | 48 | 0.53668327 | 0.999982120026 | c163366\_g1 c169731\_g1 c146091\_g1 c162392\_g1 | egu:105035642 egu:105039221 egu:105041436 egu:105041436 | 105035642 105039221 105041436 105041436 |  |
| Peroxisome | 9 | 113 | 0.543882340061 | 0.999982120026 | c103250\_g1 c147579\_g1 c163366\_g1 c151165\_g1 c140758\_g1 c160079\_g1 c169731\_g1 c156939\_g1 c152778\_g1 | egu:105061386 egu:105058041 egu:105035642 egu:105059069 egu:105052909 egu:105044749 egu:105039221 egu:105047481 egu:105060838 | 105061386 105058041 105035642 105059069 105052909 105044749 105039221 105047481 105060838 |  |
| Biosynthesis of unsaturated fatty acids | 3 | 36 | 0.553384133452 | 0.999982120026 | c137480\_g1 c134778\_g1 c117604\_g1 | egu:105061227 egu:105044431 egu:105052214 | 105061227 105044431 105052214 |  |
| Glycosylphosphatidylinositol(GPI)-anchor biosynthesis | 2 | 23 | 0.557089123671 | 0.999982120026 | c171137\_g4 c171137\_g1 | egu:105059466 egu:105059466 | 105059466 105059466 |  |
| Glycosphingolipid biosynthesis - globo series | 1 | 11 | 0.599592323892 | 0.999982120026 | c151091\_g1 | egu:105034893 | 105034893 |  |
| Biosynthesis of amino acids | 22 | 290 | 0.610561597068 | 0.999982120026 | c174151\_g1 c198353\_g1 c163642\_g1 c158088\_g1 c164056\_g1 c160412\_g1 c162112\_g2 c152607\_g1 c159323\_g1 c140061\_g1 c163118\_g1 c134111\_g1 c164784\_g1 c171050\_g1 c166358\_g1 c125057\_g1 c154303\_g1 c132251\_g1 c156235\_g1 c147541\_g1 c71483\_g1 c43883\_g1 | egu:105052838 egu:105059611 egu:105039219 egu:105057795 egu:105036454 egu:105035493 egu:105050625 egu:105043264 egu:105048107 egu:105032793 egu:105040940 egu:105056640 egu:105040461 egu:105048201 egu:105046827 egu:105054147 egu:105032039 egu:105060929 egu:105057764 egu:105057517 egu:105049882 egu:105045658 | 105052838 105059611 105039219 105057795 105036454 105035493 105050625 105043264 105048107 105032793 105040940 105056640 105040461 105048201 105046827 105054147 105032039 105060929 105057764 105057517 105049882 105045658 |  |
| Phenylalanine metabolism | 3 | 40 | 0.62069054679 | 0.999982120026 | c171265\_g1 c159323\_g1 c163118\_g1 | egu:105032439 egu:105048107 egu:105040940 | 105032439 105048107 105040940 |  |
| Thiamine metabolism | 1 | 12 | 0.6290116791 | 0.999982120026 | c172854\_g2 | egu:105046898 | 105046898 |  |
| Cyanoamino acid metabolism | 3 | 43 | 0.666422230844 | 0.999982120026 | c166887\_g5 c173060\_g2 c174706\_g1 | egu:105042425 egu:105042390 egu:105034542 | 105042425 105042390 105034542 |  |
| Sulfur metabolism | 3 | 43 | 0.666422230844 | 0.999982120026 | c140061\_g1 c164784\_g1 c134111\_g1 | egu:105032793 egu:105040461 egu:105056640 | 105032793 105040461 105056640 |  |
| Steroid biosynthesis | 3 | 45 | 0.694598874959 | 0.999982120026 | c173904\_g1 c152294\_g1 c134136\_g1 | egu:105049267 egu:105055883 egu:105058937 | 105049267 105055883 105058937 |  |
| Arachidonic acid metabolism | 1 | 15 | 0.704934976398 | 0.999982120026 | c140950\_g1 | egu:105048738 | 105048738 |  |
| Fructose and mannose metabolism | 6 | 90 | 0.716038953989 | 0.999982120026 | c85645\_g1 c154502\_g4 c198353\_g1 c162112\_g2 c43883\_g1 c157388\_g1 | egu:105049380 egu:105035321 egu:105059611 egu:105050625 egu:105045658 egu:105052174 | 105049380 105035321 105059611 105050625 105045658 105052174 |  |
| Pantothenate and CoA biosynthesis | 2 | 32 | 0.723897736181 | 0.999982120026 | c171119\_g1 c156962\_g1 | egu:105047967 egu:105040792 | 105047967 105040792 |  |
| Valine, leucine and isoleucine biosynthesis | 1 | 17 | 0.746715063729 | 0.999982120026 | c156235\_g1 | egu:105057764 | 105057764 |  |
| Glycine, serine and threonine metabolism | 5 | 79 | 0.74785993707 | 0.999982120026 | c71483\_g1 c157902\_g1 c163701\_g1 c132497\_g1 c147625\_g1 | egu:105049882 egu:105047380 egu:105035926 egu:105059577 egu:105040827 | 105049882 105047380 105035926 105059577 105040827 |  |
| Phenylpropanoid biosynthesis | 11 | 168 | 0.77186262863 | 0.999982120026 | c166887\_g5 c101133\_g1 c155934\_g1 c168470\_g1 c156209\_g1 c171016\_g1 c127525\_g1 c174706\_g1 c171265\_g1 c165472\_g1 c173060\_g2 | egu:105042425 egu:105042952 egu:105052647 egu:105053765 egu:105044629 egu:105045448 egu:105059896 egu:105034542 egu:105032439 egu:105037657 egu:105042390 | 105042425 105042952 105052647 105053765 105044629 105045448 105059896 105034542 105032439 105037657 105042390 |  |
| Glycerophospholipid metabolism | 7 | 112 | 0.779588388373 | 0.999982120026 | c167963\_g1 c163169\_g1 c158576\_g4 c163563\_g1 c140950\_g1 c12992\_g1 c162118\_g1 | egu:105059048 egu:105038022 egu:105055982 egu:105042021 egu:105048738 egu:105052307 egu:105043957 | 105059048 105038022 105055982 105042021 105048738 105052307 105043957 |  |
| One carbon pool by folate | 1 | 19 | 0.782584604718 | 0.999982120026 | c163701\_g1 | egu:105035926 | 105035926 |  |
| Sphingolipid metabolism | 2 | 39 | 0.813964951818 | 0.999982120026 | c143298\_g1 c173984\_g2 | egu:105041725 egu:105041599 | 105041725 105041599 |  |
| Amino sugar and nucleotide sugar metabolism | 10 | 161 | 0.814465996586 | 0.999982120026 | c133303\_g2 c133070\_g1 c157388\_g1 c170305\_g2 c171835\_g1 c158447\_g1 c151091\_g1 c95508\_g1 c123366\_g1 c150645\_g1 | egu:105040562 egu:105060694 egu:105052174 egu:105048493 egu:105036181 egu:105039344 egu:105034893 egu:105047457 egu:105060320 egu:105053413 | 105040562 105060694 105052174 105048493 105036181 105039344 105034893 105047457 105060320 105053413 |  |
| Pentose and glucuronate interconversions | 5 | 87 | 0.814797644993 | 0.999982120026 | c162681\_g1 c149552\_g1 c161417\_g4 c161417\_g3 c198267\_g1 | egu:105039992 egu:105053626 egu:105051305 egu:105043162 egu:105043158 | 105039992 105053626 105051305 105043162 105043158 |  |
| Inositol phosphate metabolism | 4 | 72 | 0.818528950479 | 0.999982120026 | c161796\_g1 c43883\_g1 c166373\_g4 c198353\_g1 | egu:105049214 egu:105045658 egu:105058765 egu:105059611 | 105049214 105045658 105058765 105059611 |  |
| Phagosome | 7 | 118 | 0.820374271323 | 0.999982120026 | c141394\_g1 c141394\_g3 c158106\_g1 c105637\_g1 c141160\_g2 c150055\_g1 c158225\_g2 | egu:105059836 egu:105045457 egu:105051928 egu:105046462 egu:105043351 egu:105054293 egu:105054774 | 105059836 105045457 105051928 105046462 105043351 105054293 105054774 |  |
| Nicotinate and nicotinamide metabolism | 1 | 22 | 0.827101340645 | 0.999982120026 | c171119\_g1 | egu:105047967 | 105047967 |  |
| Folate biosynthesis | 1 | 22 | 0.827101340645 | 0.999982120026 | c107029\_g1 | egu:105039713 | 105039713 |  |
| Butanoate metabolism | 1 | 22 | 0.827101340645 | 0.999982120026 | c167954\_g1 | egu:105047663 | 105047663 |  |
| beta-Alanine metabolism | 2 | 41 | 0.834375550968 | 0.999982120026 | c98584\_g1 c163847\_g1 | egu:105056213 egu:105032618 | 105056213 105032618 |  |
| Selenocompound metabolism | 1 | 23 | 0.839815662 | 0.999982120026 | c134111\_g1 | egu:105056640 | 105056640 |  |
| Tryptophan metabolism | 2 | 42 | 0.8438031797 | 0.999982120026 | c154527\_g1 c76721\_g1 | egu:105036212 egu:105032351 | 105036212 105032351 |  |
| Protein export | 3 | 60 | 0.850939264116 | 0.999982120026 | c132523\_g1 c170040\_g1 c123568\_g1 | egu:105055789 egu:105038252 egu:105044437 | 105055789 105038252 105044437 |  |
| Starch and sucrose metabolism | 15 | 243 | 0.859350749574 | 0.999982120026 | c166887\_g5 c167282\_g1 c171119\_g1 c172165\_g1 c133070\_g1 c165079\_g1 c158128\_g1 c174658\_g2 c162681\_g1 c170305\_g2 c166462\_g1 c174706\_g1 c156351\_g6 c161893\_g2 c173060\_g2 | egu:105042425 egu:105050772 egu:105047967 egu:105039195 egu:105060694 egu:105058402 egu:105046284 egu:105059813 egu:105039992 egu:105048493 egu:105043601 egu:105034542 egu:105053174 egu:105035544 egu:105042390 | 105042425 105050772 105047967 105039195 105060694 105058402 105046284 105059813 105039992 105048493 105043601 105034542 105053174 105035544 105042390 |  |
| RNA polymerase | 2 | 45 | 0.869230860366 | 0.999982120026 | c173864\_g1 c171631\_g8 | egu:105055141 egu:105034397 | 105055141 105034397 |  |
| Cysteine and methionine metabolism | 6 | 114 | 0.882744891778 | 0.999982120026 | c140061\_g1 c134111\_g1 c164784\_g1 c163118\_g1 c171431\_g1 c159323\_g1 | egu:105032793 egu:105056640 egu:105040461 egu:105040940 egu:105034922 egu:105048107 | 105032793 105056640 105040461 105040940 105034922 105048107 |  |
| Ascorbate and aldarate metabolism | 2 | 48 | 0.890802217776 | 0.999982120026 | c149412\_g1 c150645\_g1 | egu:105055090 egu:105053413 | 105055090 105053413 |  |
| Pyrimidine metabolism | 7 | 134 | 0.900564954767 | 0.999982120026 | c164585\_g7 c167990\_g1 c173864\_g1 c171508\_g1 c131571\_g1 c171631\_g8 c164810\_g1 | egu:105052170 egu:105035219 egu:105055141 egu:105052170 egu:105034341 egu:105034397 egu:105052573 | 105052170 105035219 105055141 105052170 105034341 105034397 105052573 |  |
| Phosphatidylinositol signaling system | 3 | 71 | 0.9161443888 | 0.999982120026 | c161796\_g1 c158576\_g4 c166373\_g4 | egu:105049214 egu:105055982 egu:105058765 | 105049214 105055982 105058765 |  |
| Ether lipid metabolism | 1 | 32 | 0.919470433808 | 0.999982120026 | c140950\_g1 | egu:105048738 | 105048738 |  |
| Ubiquitin mediated proteolysis | 8 | 159 | 0.930100907133 | 0.999982120026 | c163535\_g1 c151484\_g1 c27396\_g2 c156645\_g1 c133199\_g1 c127313\_g1 c145598\_g2 c167530\_g4 | egu:105057710 egu:105040742 egu:105040742 egu:105055105 egu:105059434 egu:105053631 egu:105047918 egu:105055896 | 105057710 105040742 105040742 105055105 105059434 105053631 105047918 105055896 |  |
| RNA degradation | 6 | 130 | 0.940230506999 | 0.999982120026 | c121960\_g1 c167990\_g1 c163642\_g1 c168902\_g1 c159391\_g2 c166194\_g5 | egu:105035877 egu:105035219 egu:105039219 egu:105050147 egu:105040019 egu:105060207 | 105035877 105035219 105039219 105050147 105040019 105060207 |  |
| Purine metabolism | 9 | 181 | 0.944423562617 | 0.999982120026 | c171119\_g1 c170305\_g2 c171631\_g8 c162518\_g1 c171050\_g1 c173864\_g1 c156760\_g1 c131571\_g1 c167990\_g1 | egu:105047967 egu:105048493 egu:105034397 egu:105037896 egu:105048201 egu:105055141 egu:105054024 egu:105034341 egu:105035219 | 105047967 105048493 105034397 105037896 105048201 105055141 105054024 105034341 105035219 |  |
| Galactose metabolism | 2 | 62 | 0.954220510183 | 0.999982120026 | c174658\_g2 c170305\_g2 | egu:105059813 egu:105048493 | 105059813 105048493 |  |
| Base excision repair | 1 | 44 | 0.96783393559 | 0.999982120026 | c157238\_g1 | egu:105050470 | 105050470 |  |
| Proteasome | 2 | 75 | 0.980215629593 | 0.999982120026 | c140041\_g1 c161627\_g1 | egu:105050449 egu:105042820 | 105050449 105042820 |  |
| Protein processing in endoplasmic reticulum | 12 | 257 | 0.9804565231 | 0.999982120026 | c132613\_g1 c168095\_g1 c159704\_g1 c141023\_g1 c168421\_g1 c167026\_g8 c172850\_g2 c156645\_g1 c152923\_g1 c151484\_g1 c27396\_g2 c171346\_g1 | egu:105042852 egu:105050569 egu:105042748 egu:105034003 egu:105050014 egu:105056346 egu:105050569 egu:105055105 egu:105042852 egu:105040742 egu:105040742 egu:105056954 | 105042852 105050569 105042748 105034003 105050014 105056346 105050569 105055105 105042852 105040742 105040742 105056954 |  |
| Glutathione metabolism | 3 | 105 | 0.988083116695 | 0.999982120026 | c140974\_g1 c84836\_g1 c149412\_g1 | egu:105055154 egu:105055154 egu:105055090 | 105055154 105055154 105055090 |  |
| mRNA surveillance pathway | 5 | 149 | 0.990195110809 | 0.999982120026 | c116624\_g1 c121960\_g1 c153337\_g3 c158179\_g1 c168902\_g1 | egu:105039096 egu:105035877 egu:105046347 egu:105052667 egu:105050147 | 105039096 105035877 105046347 105052667 105050147 |  |
| Ribosome biogenesis in eukaryotes | 2 | 94 | 0.994414949927 | 0.999982120026 | c113253\_g1 c150398\_g4 | egu:105054640 egu:105054641 | 105054640 105054641 |  |
| Nucleotide excision repair | 1 | 70 | 0.995609349185 | 0.999982120026 | c141023\_g1 | egu:105034003 | 105034003 |  |
| Spliceosome | 7 | 227 | 0.998733161474 | 0.999982120026 | c162358\_g1 c83498\_g1 c169453\_g6 c156221\_g2 c156221\_g1 c155072\_g1 c171346\_g1 | egu:105036282 egu:105053459 egu:105045262 egu:105054037 egu:105054037 egu:105038221 egu:105056954 | 105036282 105053459 105045262 105054037 105054037 105038221 105056954 |  |
| Plant-pathogen interaction | 5 | 220 | 0.999831484112 | 0.999982120026 | c163371\_g1 c27081\_g1 c143148\_g1 c168421\_g1 c164943\_g1 | egu:105052035 egu:105057238 egu:105046801 egu:105050014 egu:105057983 | 105052035 105057238 105046801 105050014 105057983 |  |
| Endocytosis | 3 | 197 | 0.999969165584 | 0.999982120026 | c168008\_g1 c164929\_g1 c171346\_g1 | egu:105038113 egu:105061464 egu:105056954 | 105038113 105061464 105056954 |  |
| RNA transport | 3 | 205 | 0.999982120026 | 0.999982120026 | c162792\_g2 c121960\_g1 c168902\_g1 | egu:105047852 egu:105035877 egu:105050147 | 105047852 105035877 105050147 |  |
