# Supplementary material for: Key anti-freeze genes and pathways of Lanzhou lily (Lilium davidii, var. unicolor) during the seedling stage
Source: PLoS One. 2024 Mar 21;19(3):e0299259. doi: 10.1371/journal.pone.0299259 (PMC10956819; doi:10.1371/journal.pone.0299259)
Supplement: S2 File — (ZIP) [file pone.0299259.s005.zip › S2 Zip/src/egu00010.html]

egu00010


- egu:105050625

- Down regulated genes

c162112\_g2(-0.95192)

- egu:105041436

- Down regulated genes

c146091\_g1(-1.5306) c162392\_g1(-1.1431)

- egu:105059287

- Down regulated genes

c163496\_g1(-0.6363)
- egu:105034969

- Down regulated genes

c113371\_g2(-0.73611)
- egu:105034723

- Down regulated genes

c169294\_g2(-0.85273)

- egu:105059287

- Down regulated genes

c163496\_g1(-0.6363)
- egu:105034969

- Down regulated genes

c113371\_g2(-0.73611)
- egu:105034723

- Down regulated genes

c169294\_g2(-0.85273)

- egu:105048201

- Down regulated genes

c171050\_g1(-0.65915)

- egu:105039219

- Down regulated genes

c163642\_g1(-0.73792)

- egu:105059611

- Down regulated genes

c198353\_g1(-0.63625)
- egu:105045658

- Down regulated genes

c43883\_g1(-0.68594)

- egu:105035321

- Down regulated genes

c154502\_g4(-1.1888)

- egu:105060694

- Down regulated genes

c133070\_g1(-0.83686)

- egu:105048493

- Down regulated genes

c170305\_g2(-0.65115)

- egu:105060694

- Down regulated genes

c133070\_g1(-0.83686)

- egu:105060694

- Down regulated genes

c133070\_g1(-0.83686)

- egu:105054530

- Down regulated genes

c174574\_g3(-3.7395) c104889\_g2(-1.5635)
- egu:105034557

- Down regulated genes

c104889\_g1(-1.7144)

- egu:105049380

- Down regulated genes

c85645\_g1(-1.0533)

Close
