# Supplementary material for: Key anti-freeze genes and pathways of Lanzhou lily (Lilium davidii, var. unicolor) during the seedling stage
Source: PLoS One. 2024 Mar 21;19(3):e0299259. doi: 10.1371/journal.pone.0299259 (PMC10956819; doi:10.1371/journal.pone.0299259)
Supplement: S2 File — (ZIP) [file pone.0299259.s005.zip › S2 Zip/src/egu00030.html]

egu00030


- egu:105060694

- Down regulated genes

c133070\_g1(-0.83686)

- egu:105057517

- Down regulated genes

c147541\_g1(-0.99782)
- egu:105032039

- Down regulated genes

c154303\_g1(-1.1463)

- egu:105050625

- Down regulated genes

c162112\_g2(-0.95192)

- egu:105035321

- Down regulated genes

c154502\_g4(-1.1888)

- egu:105060694

- Down regulated genes

c133070\_g1(-0.83686)

- egu:105048493

- Down regulated genes

c170305\_g2(-0.65115)

- egu:105049380

- Down regulated genes

c85645\_g1(-1.0533)

Close
