# Supplementary material for: Key anti-freeze genes and pathways of Lanzhou lily (Lilium davidii, var. unicolor) during the seedling stage
Source: PLoS One. 2024 Mar 21;19(3):e0299259. doi: 10.1371/journal.pone.0299259 (PMC10956819; doi:10.1371/journal.pone.0299259)
Supplement: S2 File — (ZIP) [file pone.0299259.s005.zip › S2 Zip/src/egu00040.html]

egu00040


- egu:105039992

- Down regulated genes

c162681\_g1(-2.6629)

- egu:105043158

- Down regulated genes

c198267\_g1(-4.5233)
- egu:105053626

- Down regulated genes

c149552\_g1(-2.7525)
- egu:105051305

- Down regulated genes

c161417\_g4(-2.2958)
- egu:105043162

- Down regulated genes

c161417\_g3(-1.871)

Close
