# Supplementary material for: Key anti-freeze genes and pathways of Lanzhou lily (Lilium davidii, var. unicolor) during the seedling stage
Source: PLoS One. 2024 Mar 21;19(3):e0299259. doi: 10.1371/journal.pone.0299259 (PMC10956819; doi:10.1371/journal.pone.0299259)
Supplement: S2 File — (ZIP) [file pone.0299259.s005.zip › S2 Zip/src/egu00051.html]

egu00051


- egu:105052174

- Down regulated genes

c157388\_g1(-0.57474)

- egu:105049380

- Down regulated genes

c85645\_g1(-1.0533)

- egu:105059611

- Down regulated genes

c198353\_g1(-0.63625)
- egu:105045658

- Down regulated genes

c43883\_g1(-0.68594)

- egu:105050625

- Down regulated genes

c162112\_g2(-0.95192)

- egu:105050625

- Down regulated genes

c162112\_g2(-0.95192)

- egu:105035321

- Down regulated genes

c154502\_g4(-1.1888)

Close
