# Supplementary material for: Key anti-freeze genes and pathways of Lanzhou lily (Lilium davidii, var. unicolor) during the seedling stage
Source: PLoS One. 2024 Mar 21;19(3):e0299259. doi: 10.1371/journal.pone.0299259 (PMC10956819; doi:10.1371/journal.pone.0299259)
Supplement: S2 File — (ZIP) [file pone.0299259.s005.zip › S2 Zip/src/egu00061.html]

egu00061


- egu:105049664

- Down regulated genes

c104546\_g1(-0.7195)

- egu:105050202

- Down regulated genes

c153334\_g2(-1.1084) c153334\_g1(-1.4904)

- egu:105051936

- Down regulated genes

c152279\_g1(-0.68831)

- egu:105053059

- Down regulated genes

c164754\_g1(-1.0312)

- egu:105049664

- Down regulated genes

c104546\_g1(-0.7195)

- egu:105049664

- Down regulated genes

c104546\_g1(-0.7195)

- egu:105053059

- Down regulated genes

c164754\_g1(-1.0312)

- egu:105053059

- Down regulated genes

c164754\_g1(-1.0312)

- egu:105053059

- Down regulated genes

c164754\_g1(-1.0312)

- egu:105053059

- Down regulated genes

c164754\_g1(-1.0312)

- egu:105053059

- Down regulated genes

c164754\_g1(-1.0312)

- egu:105053059

- Down regulated genes

c164754\_g1(-1.0312)

- egu:105050202

- Down regulated genes

c153334\_g2(-1.1084) c153334\_g1(-1.4904)

- egu:105050202

- Down regulated genes

c153334\_g2(-1.1084) c153334\_g1(-1.4904)

- egu:105050202

- Down regulated genes

c153334\_g2(-1.1084) c153334\_g1(-1.4904)

- egu:105050202

- Down regulated genes

c153334\_g2(-1.1084) c153334\_g1(-1.4904)

- egu:105050202

- Down regulated genes

c153334\_g2(-1.1084) c153334\_g1(-1.4904)

- egu:105050202

- Down regulated genes

c153334\_g2(-1.1084) c153334\_g1(-1.4904)

- egu:105050202

- Down regulated genes

c153334\_g2(-1.1084) c153334\_g1(-1.4904)

- egu:105044732

- Down regulated genes

c147420\_g1(-0.9672)

- egu:105053059

- Down regulated genes

c164754\_g1(-1.0312)

- egu:105051936

- Down regulated genes

c152279\_g1(-0.68831)

- egu:105051936

- Down regulated genes

c152279\_g1(-0.68831)

- egu:105051936

- Down regulated genes

c152279\_g1(-0.68831)

- egu:105051936

- Down regulated genes

c152279\_g1(-0.68831)

- egu:105051936

- Down regulated genes

c152279\_g1(-0.68831)

- egu:105051936

- Down regulated genes

c152279\_g1(-0.68831)

- egu:105051936

- Down regulated genes

c152279\_g1(-0.68831)

- egu:105035642

- Down regulated genes

c163366\_g1(-1.7043)
- egu:105039221

- Down regulated genes

c169731\_g1(-1.0084)

Close
