# Supplementary material for: Key anti-freeze genes and pathways of Lanzhou lily (Lilium davidii, var. unicolor) during the seedling stage
Source: PLoS One. 2024 Mar 21;19(3):e0299259. doi: 10.1371/journal.pone.0299259 (PMC10956819; doi:10.1371/journal.pone.0299259)
Supplement: S2 File — (ZIP) [file pone.0299259.s005.zip › S2 Zip/src/egu00062.html]

egu00062


- egu:105052214

- Down regulated genes

c117604\_g1(-0.96857)

- egu:105044431

- Down regulated genes

c134778\_g1(-1.5005)

- egu:105039895

- Down regulated genes

c134603\_g2(-1.9882)
- egu:105048315

- Down regulated genes

c164323\_g1(-0.74079)
- egu:105041077

- Down regulated genes

c166224\_g1(-1.1149) c150017\_g1(-1.3389)
- egu:105036065

- Down regulated genes

c163278\_g2(-3.3645)
- egu:105044215

- Down regulated genes

c148702\_g1(-1.9471)
- egu:105047165

- Down regulated genes

c164323\_g2(-0.67365)

Close
