# Supplementary material for: Key anti-freeze genes and pathways of Lanzhou lily (Lilium davidii, var. unicolor) during the seedling stage
Source: PLoS One. 2024 Mar 21;19(3):e0299259. doi: 10.1371/journal.pone.0299259 (PMC10956819; doi:10.1371/journal.pone.0299259)
Supplement: S2 File — (ZIP) [file pone.0299259.s005.zip › S2 Zip/src/egu00073.html]

egu00073


- egu:105042911

- Down regulated genes

c165906\_g1(-3.6344)

- egu:105036883

- Down regulated genes

c140465\_g1(-2.549)

- egu:105047658

- Down regulated genes

c145863\_g1(-1.687)

- egu:105061386

- Down regulated genes

c103250\_g1(-2.8827)

- egu:105042911

- Down regulated genes

c165906\_g1(-3.6344)
- egu:105036883

- Down regulated genes

c140465\_g1(-2.549)

Close
