# Supplementary material for: Key anti-freeze genes and pathways of Lanzhou lily (Lilium davidii, var. unicolor) during the seedling stage
Source: PLoS One. 2024 Mar 21;19(3):e0299259. doi: 10.1371/journal.pone.0299259 (PMC10956819; doi:10.1371/journal.pone.0299259)
Supplement: S2 File — (ZIP) [file pone.0299259.s005.zip › S2 Zip/src/egu00190.html]

egu00190


- egu:105055812

- Down regulated genes

c166082\_g1(-0.608)

- egu:105044713

- Down regulated genes

c169665\_g2(-0.856)

- egu:105046559

- Down regulated genes

c152482\_g1(-0.69839)

- egu:105057959

- Down regulated genes

c123071\_g1(-0.66584)

- egu:105055812

- Down regulated genes

c166082\_g1(-0.608)
- egu:105057959

- Down regulated genes

c123071\_g1(-0.66584)
- egu:105043983

- Down regulated genes

c147195\_g1(-0.7483)
- egu:105044713

- Down regulated genes

c169665\_g2(-0.856)
- egu:105046559

- Down regulated genes

c152482\_g1(-0.69839)

- egu:105055812

- Down regulated genes

c166082\_g1(-0.608)
- egu:105057959

- Down regulated genes

c123071\_g1(-0.66584)
- egu:105043983

- Down regulated genes

c147195\_g1(-0.7483)
- egu:105044713

- Down regulated genes

c169665\_g2(-0.856)
- egu:105046559

- Down regulated genes

c152482\_g1(-0.69839)

- egu:105059182

- Down regulated genes

c166378\_g2(-1.1839)

- egu:105055901

- Down regulated genes

c150729\_g1(-0.75488)

- egu:105054293

- Down regulated genes

c150055\_g1(-0.56168)
- egu:105051928

- Down regulated genes

c158106\_g1(-0.60077)
- egu:105049540

- Down regulated genes

c143903\_g1(-0.92645)
- egu:105043809

- Down regulated genes

c149832\_g1(-0.57763)
- egu:105047063

- Down regulated genes

c174660\_g1(-2.5215)
- egu:105033023

- Down regulated genes

c152833\_g1(-1.4059)
- egu:105046198

- Down regulated genes

c121701\_g1(-0.78195)

- egu:105043730

- Down regulated genes

c127506\_g1(-1.9574)
- egu:105052943

- Down regulated genes

c172680\_g2(-2.2265)
- egu:105039662

- Down regulated genes

c172080\_g4(-1.023)
- egu:105039277

- Down regulated genes

c161726\_g1(-1.4471)

- egu:105060092

- Down regulated genes

c158605\_g1(-0.67396)

- egu:105059182

- Down regulated genes

c166378\_g2(-1.1839)

- egu:105055901

- Down regulated genes

c150729\_g1(-0.75488)

- egu:105033023

- Down regulated genes

c152833\_g1(-1.4059)
- egu:105046198

- Down regulated genes

c121701\_g1(-0.78195)

- egu:105049540

- Down regulated genes

c143903\_g1(-0.92645)

- egu:105047063

- Down regulated genes

c174660\_g1(-2.5215)

- egu:105043809

- Down regulated genes

c149832\_g1(-0.57763)

- egu:105054293

- Down regulated genes

c150055\_g1(-0.56168)

- egu:105051928

- Down regulated genes

c158106\_g1(-0.60077)

- egu:105043983

- Down regulated genes

c147195\_g1(-0.7483)

Close
