# Supplementary material for: Key anti-freeze genes and pathways of Lanzhou lily (Lilium davidii, var. unicolor) during the seedling stage
Source: PLoS One. 2024 Mar 21;19(3):e0299259. doi: 10.1371/journal.pone.0299259 (PMC10956819; doi:10.1371/journal.pone.0299259)
Supplement: S2 File — (ZIP) [file pone.0299259.s005.zip › S2 Zip/src/egu00195.html]

egu00195


- egu:105044241

- Down regulated genes

c48634\_g1(-1.025)

- egu:105049540

- Down regulated genes

c143903\_g1(-0.92645)

- egu:105056235

- Down regulated genes

c76799\_g1(-1.1023)

- egu:105053658

- Down regulated genes

c153193\_g1(-1.4265)

- egu:105037935

- Down regulated genes

c140011\_g1(-1.0823)

- egu:105044486

- Down regulated genes

c152881\_g1(-1.2793)
- egu:105033747

- Down regulated genes

c158839\_g1(-1.0869)

- egu:105044080

- Down regulated genes

c149607\_g1(-0.83277)

- egu:105033023

- Down regulated genes

c152833\_g1(-1.4059)
- egu:105046198

- Down regulated genes

c121701\_g1(-0.78195)

- egu:105049872

- Down regulated genes

c237057\_g1(-1.0446)

- egu:105049872

- Down regulated genes

c237057\_g1(-1.0446)

- egu:105055143

- Down regulated genes

c84948\_g1(-0.95125)

- egu:105035425

- Down regulated genes

c13699\_g1(-1.0054)
- egu:105034183

- Down regulated genes

c158296\_g1(-1.2751)

- egu:105044080

- Down regulated genes

c149607\_g1(-0.83277)

- egu:105055013

- Down regulated genes

c131998\_g1(-1.1935)

- egu:105043122

- Down regulated genes

c237127\_g1(-0.75112)

- egu:105046752

- Down regulated genes

c198283\_g1(-1.0695)

- egu:105037273

- Down regulated genes

c71692\_g1(-1.3464)

- egu:105046700

- Down regulated genes

c153649\_g1(-1.3114)

- egu:105038844

- Down regulated genes

c123692\_g1(-0.97171)

- egu:105051755

- Down regulated genes

c76010\_g1(-1.0173)

- egu:105047072

- Down regulated genes

c106880\_g1(-0.94767)

- egu:105037794

- Down regulated genes

c134148\_g1(-1.0521)

- egu:105054056

- Down regulated genes

c151822\_g1(-0.84601)

- egu:105056630

- Down regulated genes

c156755\_g1(-0.94505)
- egu:105046935

- Down regulated genes

c84926\_g1(-0.70039)

- egu:105049540

- Down regulated genes

c143903\_g1(-0.92645)
- egu:105033023

- Down regulated genes

c152833\_g1(-1.4059)
- egu:105046198

- Down regulated genes

c121701\_g1(-0.78195)

- egu:105038285

- Down regulated genes

c42744\_g1(-1.0193)

Close
