# Supplementary material for: Key anti-freeze genes and pathways of Lanzhou lily (Lilium davidii, var. unicolor) during the seedling stage
Source: PLoS One. 2024 Mar 21;19(3):e0299259. doi: 10.1371/journal.pone.0299259 (PMC10956819; doi:10.1371/journal.pone.0299259)
Supplement: S2 File — (ZIP) [file pone.0299259.s005.zip › S2 Zip/src/egu00230.html]

egu00230


- egu:105035219

- Down regulated genes

c167990\_g1(-0.71128)

- egu:105055141

- Down regulated genes

c173864\_g1(-0.58137)
- egu:105034397

- Down regulated genes

c171631\_g8(-3.183)

- egu:105034341

- Down regulated genes

c131571\_g1(-0.85538)

- egu:105034341

- Down regulated genes

c131571\_g1(-0.85538)

- egu:105037896

- Down regulated genes

c162518\_g1(-0.75828)

- egu:105054024

- Down regulated genes

c156760\_g1(-2.1256)

- egu:105034341

- Down regulated genes

c131571\_g1(-0.85538)

- egu:105035219

- Down regulated genes

c167990\_g1(-0.71128)

- egu:105034341

- Down regulated genes

c131571\_g1(-0.85538)

- egu:105034341

- Down regulated genes

c131571\_g1(-0.85538)

- egu:105055141

- Down regulated genes

c173864\_g1(-0.58137)
- egu:105034397

- Down regulated genes

c171631\_g8(-3.183)

- egu:105037896

- Down regulated genes

c162518\_g1(-0.75828)

- egu:105034341

- Down regulated genes

c131571\_g1(-0.85538)

- egu:105048201

- Down regulated genes

c171050\_g1(-0.65915)

- egu:105048201

- Down regulated genes

c171050\_g1(-0.65915)

- egu:105054024

- Down regulated genes

c156760\_g1(-2.1256)

- egu:105054024

- Down regulated genes

c156760\_g1(-2.1256)

- egu:105048201

- Down regulated genes

c171050\_g1(-0.65915)

- egu:105048201

- Down regulated genes

c171050\_g1(-0.65915)

- egu:105048493

- Down regulated genes

c170305\_g2(-0.65115)

- egu:105047967

- Down regulated genes

c171119\_g1(-0.79494)

- egu:105047967

- Down regulated genes

c171119\_g1(-0.79494)

Close
