# Supplementary material for: Key anti-freeze genes and pathways of Lanzhou lily (Lilium davidii, var. unicolor) during the seedling stage
Source: PLoS One. 2024 Mar 21;19(3):e0299259. doi: 10.1371/journal.pone.0299259 (PMC10956819; doi:10.1371/journal.pone.0299259)
Supplement: S2 File — (ZIP) [file pone.0299259.s005.zip › S2 Zip/src/egu00240.html]

egu00240


- egu:105035219

- Down regulated genes

c167990\_g1(-0.71128)

- egu:105055141

- Down regulated genes

c173864\_g1(-0.58137)
- egu:105034397

- Down regulated genes

c171631\_g8(-3.183)

- egu:105034341

- Down regulated genes

c131571\_g1(-0.85538)

- egu:105052573

- Down regulated genes

c164810\_g1(-0.92191)

- egu:105052170

- Down regulated genes

c164585\_g7(-0.83601) c171508\_g1(-0.91708)

- egu:105055141

- Down regulated genes

c173864\_g1(-0.58137)
- egu:105034397

- Down regulated genes

c171631\_g8(-3.183)

- egu:105035219

- Down regulated genes

c167990\_g1(-0.71128)

- egu:105034341

- Down regulated genes

c131571\_g1(-0.85538)

- egu:105034341

- Down regulated genes

c131571\_g1(-0.85538)

- egu:105034341

- Down regulated genes

c131571\_g1(-0.85538)

- egu:105034341

- Down regulated genes

c131571\_g1(-0.85538)

- egu:105034341

- Down regulated genes

c131571\_g1(-0.85538)

Close
