# Supplementary material for: Key anti-freeze genes and pathways of Lanzhou lily (Lilium davidii, var. unicolor) during the seedling stage
Source: PLoS One. 2024 Mar 21;19(3):e0299259. doi: 10.1371/journal.pone.0299259 (PMC10956819; doi:10.1371/journal.pone.0299259)
Supplement: S2 File — (ZIP) [file pone.0299259.s005.zip › S2 Zip/src/egu00260.html]

egu00260


- egu:105047380

- Down regulated genes

c157902\_g1(-0.6411)
- egu:105040827

- Down regulated genes

c147625\_g1(-0.82125)

- egu:105049882

- Down regulated genes

c71483\_g1(-0.84471)

- egu:105035926

- Down regulated genes

c163701\_g1(-1.3044)

- egu:105049882

- Down regulated genes

c71483\_g1(-0.84471)

- egu:105059577

- Down regulated genes

c132497\_g1(-1.3436)

Close
