# Supplementary material for: Key anti-freeze genes and pathways of Lanzhou lily (Lilium davidii, var. unicolor) during the seedling stage
Source: PLoS One. 2024 Mar 21;19(3):e0299259. doi: 10.1371/journal.pone.0299259 (PMC10956819; doi:10.1371/journal.pone.0299259)
Supplement: S2 File — (ZIP) [file pone.0299259.s005.zip › S2 Zip/src/egu00460.html]

egu00460


- egu:105034542

- Down regulated genes

c174706\_g1(-1.4093)
- egu:105042425

- Down regulated genes

c166887\_g5(-1.2344)
- egu:105042390

- Down regulated genes

c173060\_g2(-1.0904)

- egu:105034542

- Down regulated genes

c174706\_g1(-1.4093)
- egu:105042425

- Down regulated genes

c166887\_g5(-1.2344)
- egu:105042390

- Down regulated genes

c173060\_g2(-1.0904)

- egu:105034542

- Down regulated genes

c174706\_g1(-1.4093)
- egu:105042425

- Down regulated genes

c166887\_g5(-1.2344)
- egu:105042390

- Down regulated genes

c173060\_g2(-1.0904)

- egu:105034542

- Down regulated genes

c174706\_g1(-1.4093)
- egu:105042425

- Down regulated genes

c166887\_g5(-1.2344)
- egu:105042390

- Down regulated genes

c173060\_g2(-1.0904)

- egu:105034542

- Down regulated genes

c174706\_g1(-1.4093)
- egu:105042425

- Down regulated genes

c166887\_g5(-1.2344)
- egu:105042390

- Down regulated genes

c173060\_g2(-1.0904)

Close
