# Supplementary material for: Key anti-freeze genes and pathways of Lanzhou lily (Lilium davidii, var. unicolor) during the seedling stage
Source: PLoS One. 2024 Mar 21;19(3):e0299259. doi: 10.1371/journal.pone.0299259 (PMC10956819; doi:10.1371/journal.pone.0299259)
Supplement: S2 File — (ZIP) [file pone.0299259.s005.zip › S2 Zip/src/egu00500.html]

egu00500


- egu:105042425

- Down regulated genes

c166887\_g5(-1.2344)
- egu:105042390

- Down regulated genes

c173060\_g2(-1.0904)
- egu:105034542

- Down regulated genes

c174706\_g1(-1.4093)

- egu:105042425

- Down regulated genes

c166887\_g5(-1.2344)
- egu:105042390

- Down regulated genes

c173060\_g2(-1.0904)
- egu:105034542

- Down regulated genes

c174706\_g1(-1.4093)

- egu:105059813

- Down regulated genes

c174658\_g2(-0.92638)

- egu:105042425

- Down regulated genes

c166887\_g5(-1.2344)
- egu:105042390

- Down regulated genes

c173060\_g2(-1.0904)
- egu:105034542

- Down regulated genes

c174706\_g1(-1.4093)

- egu:105035544

- Down regulated genes

c161893\_g2(-2.1524)

- egu:105047967

- Down regulated genes

c171119\_g1(-0.79494)

- egu:105050772

- Down regulated genes

c167282\_g1(-1.0581)
- egu:105053174

- Down regulated genes

c156351\_g6(-1.1871)
- egu:105039195

- Down regulated genes

c172165\_g1(-1.3118)

- egu:105048493

- Down regulated genes

c170305\_g2(-0.65115)

- egu:105060694

- Down regulated genes

c133070\_g1(-0.83686)

- egu:105043601

- Down regulated genes

c166462\_g1(-1.5815)
- egu:105050772

- Down regulated genes

c167282\_g1(-1.0581)
- egu:105053174

- Down regulated genes

c156351\_g6(-1.1871)
- egu:105039195

- Down regulated genes

c172165\_g1(-1.3118)
- egu:105058402

- Down regulated genes

c165079\_g1(-2.4836)

- egu:105059813

- Down regulated genes

c174658\_g2(-0.92638)

- egu:105046284

- Down regulated genes

c158128\_g1(-1.2155)

- egu:105035544

- Down regulated genes

c161893\_g2(-2.1524)

Close
