# Supplementary material for: Key anti-freeze genes and pathways of Lanzhou lily (Lilium davidii, var. unicolor) during the seedling stage
Source: PLoS One. 2024 Mar 21;19(3):e0299259. doi: 10.1371/journal.pone.0299259 (PMC10956819; doi:10.1371/journal.pone.0299259)
Supplement: S2 File — (ZIP) [file pone.0299259.s005.zip › S2 Zip/src/egu00520.html]

egu00520


- egu:105034893

- Down regulated genes

c151091\_g1(-1.0101)

- egu:105060320

- Down regulated genes

c123366\_g1(-1.074)

- egu:105060320

- Down regulated genes

c123366\_g1(-1.074)

- egu:105039344

- Down regulated genes

c158447\_g1(-0.83819)

- egu:105047457

- Down regulated genes

c95508\_g1(-0.92387)
- egu:105040562

- Down regulated genes

c133303\_g2(-1.1351)

- egu:105048493

- Down regulated genes

c170305\_g2(-0.65115)

- egu:105052174

- Down regulated genes

c157388\_g1(-0.57474)

- egu:105036181

- Down regulated genes

c171835\_g1(-0.67)

- egu:105048493

- Down regulated genes

c170305\_g2(-0.65115)

- egu:105036181

- Down regulated genes

c171835\_g1(-0.67)

- egu:105053413

- Down regulated genes

c150645\_g1(-1.0292)

- egu:105053413

- Down regulated genes

c150645\_g1(-1.0292)

- egu:105060694

- Down regulated genes

c133070\_g1(-0.83686)

Close
