# Supplementary material for: Key anti-freeze genes and pathways of Lanzhou lily (Lilium davidii, var. unicolor) during the seedling stage
Source: PLoS One. 2024 Mar 21;19(3):e0299259. doi: 10.1371/journal.pone.0299259 (PMC10956819; doi:10.1371/journal.pone.0299259)
Supplement: S2 File — (ZIP) [file pone.0299259.s005.zip › S2 Zip/src/egu00531.html]

egu00531


- egu:105057479

- Down regulated genes

c168109\_g1(-1.4718)

- egu:105034893

- Down regulated genes

c151091\_g1(-1.0101)

- egu:105057579

- Down regulated genes

c168498\_g1(-1.1912)

- egu:105034893

- Down regulated genes

c151091\_g1(-1.0101)

- egu:105057579

- Down regulated genes

c168498\_g1(-1.1912)

- egu:105057579

- Down regulated genes

c168498\_g1(-1.1912)

- egu:105034893

- Down regulated genes

c151091\_g1(-1.0101)

- egu:105034893

- Down regulated genes

c151091\_g1(-1.0101)

Close
