# Supplementary material for: Key anti-freeze genes and pathways of Lanzhou lily (Lilium davidii, var. unicolor) during the seedling stage
Source: PLoS One. 2024 Mar 21;19(3):e0299259. doi: 10.1371/journal.pone.0299259 (PMC10956819; doi:10.1371/journal.pone.0299259)
Supplement: S2 File — (ZIP) [file pone.0299259.s005.zip › S2 Zip/src/egu00561.html]

egu00561


- egu:105041806

- Down regulated genes

c122861\_g1(-0.60726)

- egu:105052064

- Down regulated genes

c172504\_g1(-0.55075)

- egu:105039544

- Down regulated genes

c148171\_g1(-1.063)

- egu:105043957

- Down regulated genes

c162118\_g1(-0.71227)
- egu:105059048

- Down regulated genes

c167963\_g1(-1.7891)
- egu:105038022

- Down regulated genes

c163169\_g1(-0.78154)

- egu:105055982

- Down regulated genes

c158576\_g4(-3.3247)

Close
