# Supplementary material for: Key anti-freeze genes and pathways of Lanzhou lily (Lilium davidii, var. unicolor) during the seedling stage
Source: PLoS One. 2024 Mar 21;19(3):e0299259. doi: 10.1371/journal.pone.0299259 (PMC10956819; doi:10.1371/journal.pone.0299259)
Supplement: S2 File — (ZIP) [file pone.0299259.s005.zip › S2 Zip/src/egu00564.html]

egu00564


- egu:105042021

- Down regulated genes

c163563\_g1(-1.423)

- egu:105048738

- Down regulated genes

c140950\_g1(-0.93241)

- egu:105055982

- Down regulated genes

c158576\_g4(-3.3247)

- egu:105048738

- Down regulated genes

c140950\_g1(-0.93241)

- egu:105043957

- Down regulated genes

c162118\_g1(-0.71227)
- egu:105059048

- Down regulated genes

c167963\_g1(-1.7891)
- egu:105038022

- Down regulated genes

c163169\_g1(-0.78154)

- egu:105052307

- Down regulated genes

c12992\_g1(-1.6016)

- egu:105059048

- Down regulated genes

c167963\_g1(-1.7891)

Close
