# Supplementary material for: Key anti-freeze genes and pathways of Lanzhou lily (Lilium davidii, var. unicolor) during the seedling stage
Source: PLoS One. 2024 Mar 21;19(3):e0299259. doi: 10.1371/journal.pone.0299259 (PMC10956819; doi:10.1371/journal.pone.0299259)
Supplement: S2 File — (ZIP) [file pone.0299259.s005.zip › S2 Zip/src/egu00592.html]

egu00592


- egu:105051386

- Down regulated genes

c158852\_g2(-0.86032)

- egu:105048738

- Down regulated genes

c140950\_g1(-0.93241)

- egu:105032483

- Down regulated genes

c172570\_g3(-0.99938)

- egu:105056286

- Down regulated genes

c154400\_g1(-1.8377)

- egu:105051386

- Down regulated genes

c158852\_g2(-0.86032)

Close
