# Supplementary material for: Key anti-freeze genes and pathways of Lanzhou lily (Lilium davidii, var. unicolor) during the seedling stage
Source: PLoS One. 2024 Mar 21;19(3):e0299259. doi: 10.1371/journal.pone.0299259 (PMC10956819; doi:10.1371/journal.pone.0299259)
Supplement: S2 File — (ZIP) [file pone.0299259.s005.zip › S2 Zip/src/egu00620.html]

egu00620


- egu:105059287

- Down regulated genes

c163496\_g1(-0.6363)
- egu:105034969

- Down regulated genes

c113371\_g2(-0.73611)
- egu:105034723

- Down regulated genes

c169294\_g2(-0.85273)

- egu:105054530

- Down regulated genes

c104889\_g2(-1.5635) c174574\_g3(-3.7395)
- egu:105034557

- Down regulated genes

c104889\_g1(-1.7144)

- egu:105048201

- Down regulated genes

c171050\_g1(-0.65915)

- egu:105042873

- Down regulated genes

c172966\_g1(-0.77465)

- egu:105040530

- Down regulated genes

c170442\_g1(-0.78779)

- egu:105059287

- Down regulated genes

c163496\_g1(-0.6363)
- egu:105034969

- Down regulated genes

c113371\_g2(-0.73611)
- egu:105034723

- Down regulated genes

c169294\_g2(-0.85273)

Close
