# Supplementary material for: Key anti-freeze genes and pathways of Lanzhou lily (Lilium davidii, var. unicolor) during the seedling stage
Source: PLoS One. 2024 Mar 21;19(3):e0299259. doi: 10.1371/journal.pone.0299259 (PMC10956819; doi:10.1371/journal.pone.0299259)
Supplement: S2 File — (ZIP) [file pone.0299259.s005.zip › S2 Zip/src/egu00710.html]

egu00710


- egu:105050625

- Down regulated genes

c162112\_g2(-0.95192)

- egu:105046280

- Down regulated genes

c158889\_g1(-0.90678)

- egu:105032039

- Down regulated genes

c154303\_g1(-1.1463)
- egu:105057517

- Down regulated genes

c147541\_g1(-0.99782)

- egu:105043976

- Down regulated genes

c164307\_g1(-0.79674)

- egu:105035321

- Down regulated genes

c154502\_g4(-1.1888)

- egu:105050625

- Down regulated genes

c162112\_g2(-0.95192)

- egu:105045658

- Down regulated genes

c43883\_g1(-0.68594)
- egu:105059611

- Down regulated genes

c198353\_g1(-0.63625)

- egu:105042746

- Down regulated genes

c121900\_g1(-0.60083)
- egu:105060347

- Down regulated genes

c160123\_g1(-1.6452)

- egu:105048437

- Down regulated genes

c168133\_g3(-1.5782)

- egu:105048107

- Down regulated genes

c159323\_g1(-0.98431)

- egu:105049882

- Down regulated genes

c71483\_g1(-0.84471)

- egu:105049882

- Down regulated genes

c71483\_g1(-0.84471)

- egu:105040530

- Down regulated genes

c170442\_g1(-0.78779)

- egu:105042873

- Down regulated genes

c172966\_g1(-0.77465)

- egu:105040530

- Down regulated genes

c170442\_g1(-0.78779)

- egu:105054530

- Down regulated genes

c104889\_g2(-1.5635) c174574\_g3(-3.7395)
- egu:105034557

- Down regulated genes

c104889\_g1(-1.7144)

Close
