# Supplementary material for: Key anti-freeze genes and pathways of Lanzhou lily (Lilium davidii, var. unicolor) during the seedling stage
Source: PLoS One. 2024 Mar 21;19(3):e0299259. doi: 10.1371/journal.pone.0299259 (PMC10956819; doi:10.1371/journal.pone.0299259)
Supplement: S2 File — (ZIP) [file pone.0299259.s005.zip › S2 Zip/src/egu00860.html]

egu00860


- egu:105045229

- Down regulated genes

c157598\_g1(-1.0974)

- egu:105036097

- Down regulated genes

c185147\_g2(-0.93846)

- egu:105036097

- Down regulated genes

c185147\_g2(-0.93846)

- egu:105036097

- Down regulated genes

c185147\_g2(-0.93846)

- egu:105044579

- Down regulated genes

c155686\_g1(-0.57873)

- egu:105045229

- Down regulated genes

c157598\_g1(-1.0974)

- egu:105035938

- Down regulated genes

c123480\_g1(-1.2045)

- egu:105057582

- Down regulated genes

c169028\_g1(-2.0429)
- egu:105040656

- Down regulated genes

c133188\_g1(-1.0414)

- egu:105044798

- Down regulated genes

c165450\_g1(-0.88869)
- egu:105040768

- Down regulated genes

c168519\_g1(-1.0519)

- egu:105044798

- Down regulated genes

c165450\_g1(-0.88869)
- egu:105040768

- Down regulated genes

c168519\_g1(-1.0519)

- egu:105049221

- Down regulated genes

c71809\_g1(-0.98861)

- egu:105051026

- Down regulated genes

c167743\_g1(-0.97445)

- egu:105054529

- Down regulated genes

c167947\_g1(-2.0036)

- egu:105052855

- Down regulated genes

c161205\_g1(-0.78676)

- egu:105037930

- Down regulated genes

c71670\_g1(-0.96871)

- egu:105044579

- Down regulated genes

c155686\_g1(-0.57873)

- egu:105037930

- Down regulated genes

c71670\_g1(-0.96871)

- egu:105044579

- Down regulated genes

c155686\_g1(-0.57873)

- egu:105044579

- Down regulated genes

c155686\_g1(-0.57873)

- egu:105058545

- Down regulated genes

c166557\_g2(-1.4097) c166557\_g1(-1.5029)

- egu:105058545

- Down regulated genes

c166557\_g2(-1.4097) c166557\_g1(-1.5029)

Close
