# Supplementary material for: Key anti-freeze genes and pathways of Lanzhou lily (Lilium davidii, var. unicolor) during the seedling stage
Source: PLoS One. 2024 Mar 21;19(3):e0299259. doi: 10.1371/journal.pone.0299259 (PMC10956819; doi:10.1371/journal.pone.0299259)
Supplement: S2 File — (ZIP) [file pone.0299259.s005.zip › S2 Zip/src/egu00900.html]

egu00900


- egu:105045599

- Down regulated genes

c165075\_g1(-0.65473)

- egu:105036971

- Down regulated genes

c159912\_g2(-2.2265)
- egu:105039425

- Down regulated genes

c159912\_g1(-2.087)

- egu:105032472

- Down regulated genes

c171401\_g2(-0.65414)
- egu:105046147

- Down regulated genes

c122896\_g1(-0.73511)

- egu:105032472

- Down regulated genes

c171401\_g2(-0.65414)
- egu:105046147

- Down regulated genes

c122896\_g1(-0.73511)

- egu:105032472

- Down regulated genes

c171401\_g2(-0.65414)

- egu:105058545

- Down regulated genes

c166557\_g2(-1.4097) c166557\_g1(-1.5029)

Close
