# Supplementary material for: Key anti-freeze genes and pathways of Lanzhou lily (Lilium davidii, var. unicolor) during the seedling stage
Source: PLoS One. 2024 Mar 21;19(3):e0299259. doi: 10.1371/journal.pone.0299259 (PMC10956819; doi:10.1371/journal.pone.0299259)
Supplement: S2 File — (ZIP) [file pone.0299259.s005.zip › S2 Zip/src/egu00904.html]

egu00904


- egu:105043222

- Down regulated genes

c155524\_g1(-2.4289)

- egu:105043222

- Down regulated genes

c155524\_g1(-2.4289)

- egu:105043222

- Down regulated genes

c155524\_g1(-2.4289)

- egu:105043222

- Down regulated genes

c155524\_g1(-2.4289)

- egu:105043222

- Down regulated genes

c155524\_g1(-2.4289)

- egu:105043222

- Down regulated genes

c155524\_g1(-2.4289)

- egu:105032920

- Down regulated genes

c159595\_g1(-2.5898) c142425\_g1(-3.0449)

Close
