# Supplementary material for: Key anti-freeze genes and pathways of Lanzhou lily (Lilium davidii, var. unicolor) during the seedling stage
Source: PLoS One. 2024 Mar 21;19(3):e0299259. doi: 10.1371/journal.pone.0299259 (PMC10956819; doi:10.1371/journal.pone.0299259)
Supplement: S2 File — (ZIP) [file pone.0299259.s005.zip › S2 Zip/src/egu00910.html]

egu00910


- egu:105048068

- Down regulated genes

c115915\_g1(-1.1753)
- egu:105052122

- Down regulated genes

c142597\_g1(-0.79179)

- egu:105033813

- Down regulated genes

c185512\_g1(-0.87801)

- egu:105033813

- Down regulated genes

c185512\_g1(-0.87801)

- egu:105057795

- Down regulated genes

c158088\_g1(-1.4881)
- egu:105035493

- Down regulated genes

c160412\_g1(-0.71149)

- egu:105058731

- Down regulated genes

c146725\_g1(-1.1138)

Close
