# Supplementary material for: Key anti-freeze genes and pathways of Lanzhou lily (Lilium davidii, var. unicolor) during the seedling stage
Source: PLoS One. 2024 Mar 21;19(3):e0299259. doi: 10.1371/journal.pone.0299259 (PMC10956819; doi:10.1371/journal.pone.0299259)
Supplement: S2 File — (ZIP) [file pone.0299259.s005.zip › S2 Zip/src/egu00940.html]

egu00940


- egu:105042425

- Down regulated genes

c166887\_g5(-1.2344)
- egu:105042390

- Down regulated genes

c173060\_g2(-1.0904)
- egu:105034542

- Down regulated genes

c174706\_g1(-1.4093)

- egu:105045448

- Down regulated genes

c171016\_g1(-1.9038)
- egu:105053765

- Down regulated genes

c168470\_g1(-2.7871)

- egu:105045448

- Down regulated genes

c171016\_g1(-1.9038)
- egu:105053765

- Down regulated genes

c168470\_g1(-2.7871)

- egu:105045448

- Down regulated genes

c171016\_g1(-1.9038)
- egu:105053765

- Down regulated genes

c168470\_g1(-2.7871)

- egu:105045448

- Down regulated genes

c171016\_g1(-1.9038)
- egu:105053765

- Down regulated genes

c168470\_g1(-2.7871)

- egu:105052647

- Down regulated genes

c155934\_g1(-3.5798)
- egu:105042952

- Down regulated genes

c101133\_g1(-4.2291)
- egu:105044629

- Down regulated genes

c156209\_g1(-1.376)

- egu:105037657

- Down regulated genes

c165472\_g1(-1.1637)
- egu:105059896

- Down regulated genes

c127525\_g1(-1.98)

- egu:105037657

- Down regulated genes

c165472\_g1(-1.1637)
- egu:105059896

- Down regulated genes

c127525\_g1(-1.98)

- egu:105037657

- Down regulated genes

c165472\_g1(-1.1637)
- egu:105059896

- Down regulated genes

c127525\_g1(-1.98)

- egu:105037657

- Down regulated genes

c165472\_g1(-1.1637)
- egu:105059896

- Down regulated genes

c127525\_g1(-1.98)

- egu:105037657

- Down regulated genes

c165472\_g1(-1.1637)
- egu:105059896

- Down regulated genes

c127525\_g1(-1.98)

- egu:105052647

- Down regulated genes

c155934\_g1(-3.5798)
- egu:105042952

- Down regulated genes

c101133\_g1(-4.2291)
- egu:105044629

- Down regulated genes

c156209\_g1(-1.376)

- egu:105052647

- Down regulated genes

c155934\_g1(-3.5798)
- egu:105042952

- Down regulated genes

c101133\_g1(-4.2291)
- egu:105044629

- Down regulated genes

c156209\_g1(-1.376)

- egu:105052647

- Down regulated genes

c155934\_g1(-3.5798)
- egu:105042952

- Down regulated genes

c101133\_g1(-4.2291)
- egu:105044629

- Down regulated genes

c156209\_g1(-1.376)

- egu:105032439

- Down regulated genes

c171265\_g1(-2.6191)

- egu:105032439

- Down regulated genes

c171265\_g1(-2.6191)

Close
