# Supplementary material for: Key anti-freeze genes and pathways of Lanzhou lily (Lilium davidii, var. unicolor) during the seedling stage
Source: PLoS One. 2024 Mar 21;19(3):e0299259. doi: 10.1371/journal.pone.0299259 (PMC10956819; doi:10.1371/journal.pone.0299259)
Supplement: S2 File — (ZIP) [file pone.0299259.s005.zip › S2 Zip/src/egu00950.html]

egu00950


- egu:105040940

- Down regulated genes

c163118\_g1(-1.775)

- egu:105048107

- Down regulated genes

c159323\_g1(-0.98431)

Close
