# Supplementary material for: Key anti-freeze genes and pathways of Lanzhou lily (Lilium davidii, var. unicolor) during the seedling stage
Source: PLoS One. 2024 Mar 21;19(3):e0299259. doi: 10.1371/journal.pone.0299259 (PMC10956819; doi:10.1371/journal.pone.0299259)
Supplement: S2 File — (ZIP) [file pone.0299259.s005.zip › S2 Zip/src/egu01040.html]

egu01040


- egu:105044431

- Down regulated genes

c134778\_g1(-1.5005)

- egu:105052214

- Down regulated genes

c117604\_g1(-0.96857)

- egu:105044431

- Down regulated genes

c134778\_g1(-1.5005)

- egu:105052214

- Down regulated genes

c117604\_g1(-0.96857)

- egu:105044431

- Down regulated genes

c134778\_g1(-1.5005)

- egu:105052214

- Down regulated genes

c117604\_g1(-0.96857)

- egu:105044431

- Down regulated genes

c134778\_g1(-1.5005)

- egu:105052214

- Down regulated genes

c117604\_g1(-0.96857)

- egu:105044431

- Down regulated genes

c134778\_g1(-1.5005)

- egu:105052214

- Down regulated genes

c117604\_g1(-0.96857)

- egu:105044431

- Down regulated genes

c134778\_g1(-1.5005)

- egu:105052214

- Down regulated genes

c117604\_g1(-0.96857)

- egu:105044431

- Down regulated genes

c134778\_g1(-1.5005)

- egu:105052214

- Down regulated genes

c117604\_g1(-0.96857)

- egu:105044431

- Down regulated genes

c134778\_g1(-1.5005)

- egu:105052214

- Down regulated genes

c117604\_g1(-0.96857)

- egu:105044431

- Down regulated genes

c134778\_g1(-1.5005)

- egu:105052214

- Down regulated genes

c117604\_g1(-0.96857)

- egu:105044431

- Down regulated genes

c134778\_g1(-1.5005)

- egu:105052214

- Down regulated genes

c117604\_g1(-0.96857)

- egu:105044431

- Down regulated genes

c134778\_g1(-1.5005)

- egu:105052214

- Down regulated genes

c117604\_g1(-0.96857)

- egu:105044431

- Down regulated genes

c134778\_g1(-1.5005)

- egu:105052214

- Down regulated genes

c117604\_g1(-0.96857)

- egu:105044431

- Down regulated genes

c134778\_g1(-1.5005)

- egu:105052214

- Down regulated genes

c117604\_g1(-0.96857)

- egu:105044431

- Down regulated genes

c134778\_g1(-1.5005)

- egu:105052214

- Down regulated genes

c117604\_g1(-0.96857)

Close
