# Supplementary material for: Key anti-freeze genes and pathways of Lanzhou lily (Lilium davidii, var. unicolor) during the seedling stage
Source: PLoS One. 2024 Mar 21;19(3):e0299259. doi: 10.1371/journal.pone.0299259 (PMC10956819; doi:10.1371/journal.pone.0299259)
Supplement: S2 File — (ZIP) [file pone.0299259.s005.zip › S2 Zip/src/egu01110.html]

egu01110


- egu:105032439

- Down regulated genes

c171265\_g1(-2.6191)

- egu:105040768

- Down regulated genes

c168519\_g1(-1.0519)
- egu:105044798

- Down regulated genes

c165450\_g1(-0.88869)

- egu:105058545

- Down regulated genes

c166557\_g2(-1.4097) c166557\_g1(-1.5029)

- egu:105044579

- Down regulated genes

c155686\_g1(-0.57873)

- egu:105042425

- Down regulated genes

c166887\_g5(-1.2344)
- egu:105042390

- Down regulated genes

c173060\_g2(-1.0904)
- egu:105034542

- Down regulated genes

c174706\_g1(-1.4093)

- egu:105046284

- Down regulated genes

c158128\_g1(-1.2155)

- egu:105046284

- Down regulated genes

c158128\_g1(-1.2155)

- egu:105046284

- Down regulated genes

c158128\_g1(-1.2155)

- egu:105046284

- Down regulated genes

c158128\_g1(-1.2155)

- egu:105046284

- Down regulated genes

c158128\_g1(-1.2155)

- egu:105046284

- Down regulated genes

c158128\_g1(-1.2155)

- egu:105046284

- Down regulated genes

c158128\_g1(-1.2155)

- egu:105046284

- Down regulated genes

c158128\_g1(-1.2155)

- egu:105046284

- Down regulated genes

c158128\_g1(-1.2155)

- egu:105046284

- Down regulated genes

c158128\_g1(-1.2155)

- egu:105046284

- Down regulated genes

c158128\_g1(-1.2155)

- egu:105051526

- Down regulated genes

c154382\_g1(-0.69072)

- egu:105051526

- Down regulated genes

c154382\_g1(-0.69072)

- egu:105051526

- Down regulated genes

c154382\_g1(-0.69072)

- egu:105051526

- Down regulated genes

c154382\_g1(-0.69072)

- egu:105051526

- Down regulated genes

c154382\_g1(-0.69072)

- egu:105051526

- Down regulated genes

c154382\_g1(-0.69072)

- egu:105051526

- Down regulated genes

c154382\_g1(-0.69072)

- egu:105051526

- Down regulated genes

c154382\_g1(-0.69072)

- egu:105051526

- Down regulated genes

c154382\_g1(-0.69072)

- egu:105051526

- Down regulated genes

c154382\_g1(-0.69072)

- egu:105051526

- Down regulated genes

c154382\_g1(-0.69072)

- egu:105051526

- Down regulated genes

c154382\_g1(-0.69072)

- egu:105047598

- Down regulated genes

c162354\_g1(-0.98364)

- egu:105052174

- Down regulated genes

c157388\_g1(-0.57474)

- egu:105053413

- Down regulated genes

c150645\_g1(-1.0292)

- egu:105032793

- Down regulated genes

c140061\_g1(-0.79319)

- egu:105040461

- Down regulated genes

c164784\_g1(-1.0012)

- egu:105048738

- Down regulated genes

c140950\_g1(-0.93241)

- egu:105051386

- Down regulated genes

c158852\_g2(-0.86032)

- egu:105051386

- Down regulated genes

c158852\_g2(-0.86032)

- egu:105051386

- Down regulated genes

c158852\_g2(-0.86032)

- egu:105051386

- Down regulated genes

c158852\_g2(-0.86032)

- egu:105051386

- Down regulated genes

c158852\_g2(-0.86032)

- egu:105051386

- Down regulated genes

c158852\_g2(-0.86032)

- egu:105051386

- Down regulated genes

c158852\_g2(-0.86032)

- egu:105051386

- Down regulated genes

c158852\_g2(-0.86032)

- egu:105051386

- Down regulated genes

c158852\_g2(-0.86032)

- egu:105051386

- Down regulated genes

c158852\_g2(-0.86032)

- egu:105051386

- Down regulated genes

c158852\_g2(-0.86032)

- egu:105032483

- Down regulated genes

c172570\_g3(-0.99938)

- egu:105051386

- Down regulated genes

c158852\_g2(-0.86032)

- egu:105052307

- Down regulated genes

c12992\_g1(-1.6016)

- egu:105034750

- Down regulated genes

c160710\_g1(-0.88897) c166734\_g1(-1.5248) c174739\_g1(-1.5421)

- egu:105034750

- Down regulated genes

c160710\_g1(-0.88897) c166734\_g1(-1.5248) c174739\_g1(-1.5421)

- egu:105034750

- Down regulated genes

c160710\_g1(-0.88897) c166734\_g1(-1.5248) c174739\_g1(-1.5421)

- egu:105034750

- Down regulated genes

c160710\_g1(-0.88897) c166734\_g1(-1.5248) c174739\_g1(-1.5421)

- egu:105034750

- Down regulated genes

c160710\_g1(-0.88897) c166734\_g1(-1.5248) c174739\_g1(-1.5421)

- egu:105034750

- Down regulated genes

c160710\_g1(-0.88897) c166734\_g1(-1.5248) c174739\_g1(-1.5421)

- egu:105034750

- Down regulated genes

c160710\_g1(-0.88897) c166734\_g1(-1.5248) c174739\_g1(-1.5421)

- egu:105034750

- Down regulated genes

c160710\_g1(-0.88897) c166734\_g1(-1.5248) c174739\_g1(-1.5421)

- egu:105034750

- Down regulated genes

c160710\_g1(-0.88897) c166734\_g1(-1.5248) c174739\_g1(-1.5421)

- egu:105034750

- Down regulated genes

c160710\_g1(-0.88897) c166734\_g1(-1.5248) c174739\_g1(-1.5421)

- egu:105034750

- Down regulated genes

c160710\_g1(-0.88897) c166734\_g1(-1.5248) c174739\_g1(-1.5421)

- egu:105034750

- Down regulated genes

c160710\_g1(-0.88897) c166734\_g1(-1.5248) c174739\_g1(-1.5421)

- egu:105034750

- Down regulated genes

c160710\_g1(-0.88897) c166734\_g1(-1.5248) c174739\_g1(-1.5421)

- egu:105034750

- Down regulated genes

c160710\_g1(-0.88897) c166734\_g1(-1.5248) c174739\_g1(-1.5421)

- egu:105034750

- Down regulated genes

c160710\_g1(-0.88897) c166734\_g1(-1.5248) c174739\_g1(-1.5421)

- egu:105034750

- Down regulated genes

c160710\_g1(-0.88897) c166734\_g1(-1.5248) c174739\_g1(-1.5421)

- egu:105034750

- Down regulated genes

c160710\_g1(-0.88897) c166734\_g1(-1.5248) c174739\_g1(-1.5421)

- egu:105034750

- Down regulated genes

c160710\_g1(-0.88897) c166734\_g1(-1.5248) c174739\_g1(-1.5421)

- egu:105034750

- Down regulated genes

c160710\_g1(-0.88897) c166734\_g1(-1.5248) c174739\_g1(-1.5421)

- egu:105034750

- Down regulated genes

c160710\_g1(-0.88897) c166734\_g1(-1.5248) c174739\_g1(-1.5421)

- egu:105034750

- Down regulated genes

c160710\_g1(-0.88897) c166734\_g1(-1.5248) c174739\_g1(-1.5421)

- egu:105034750

- Down regulated genes

c160710\_g1(-0.88897) c166734\_g1(-1.5248) c174739\_g1(-1.5421)

- egu:105034750

- Down regulated genes

c160710\_g1(-0.88897) c166734\_g1(-1.5248) c174739\_g1(-1.5421)

- egu:105034750

- Down regulated genes

c160710\_g1(-0.88897) c166734\_g1(-1.5248) c174739\_g1(-1.5421)

- egu:105034750

- Down regulated genes

c160710\_g1(-0.88897) c166734\_g1(-1.5248) c174739\_g1(-1.5421)

- egu:105034750

- Down regulated genes

c160710\_g1(-0.88897) c166734\_g1(-1.5248) c174739\_g1(-1.5421)

- egu:105034750

- Down regulated genes

c160710\_g1(-0.88897) c166734\_g1(-1.5248) c174739\_g1(-1.5421)

- egu:105034750

- Down regulated genes

c160710\_g1(-0.88897) c166734\_g1(-1.5248) c174739\_g1(-1.5421)

- egu:105034750

- Down regulated genes

c160710\_g1(-0.88897) c166734\_g1(-1.5248) c174739\_g1(-1.5421)

- egu:105034750

- Down regulated genes

c160710\_g1(-0.88897) c166734\_g1(-1.5248) c174739\_g1(-1.5421)

- egu:105034750

- Down regulated genes

c160710\_g1(-0.88897) c166734\_g1(-1.5248) c174739\_g1(-1.5421)

- egu:105055883

- Down regulated genes

c152294\_g1(-1.1731)

- egu:105041933

- Down regulated genes

c165685\_g1(-0.89504)

- egu:105041933

- Down regulated genes

c165685\_g1(-0.89504)

- egu:105041933

- Down regulated genes

c165685\_g1(-0.89504)

- egu:105041933

- Down regulated genes

c165685\_g1(-0.89504)

- egu:105041933

- Down regulated genes

c165685\_g1(-0.89504)

- egu:105041933

- Down regulated genes

c165685\_g1(-0.89504)

- egu:105041933

- Down regulated genes

c165685\_g1(-0.89504)

- egu:105041933

- Down regulated genes

c165685\_g1(-0.89504)

- egu:105041933

- Down regulated genes

c165685\_g1(-0.89504)

- egu:105041933

- Down regulated genes

c165685\_g1(-0.89504)

- egu:105041933

- Down regulated genes

c165685\_g1(-0.89504)

- egu:105041933

- Down regulated genes

c165685\_g1(-0.89504)

- egu:105041933

- Down regulated genes

c165685\_g1(-0.89504)

- egu:105041933

- Down regulated genes

c165685\_g1(-0.89504)

- egu:105041933

- Down regulated genes

c165685\_g1(-0.89504)

- egu:105041933

- Down regulated genes

c165685\_g1(-0.89504)

- egu:105041933

- Down regulated genes

c165685\_g1(-0.89504)

- egu:105041933

- Down regulated genes

c165685\_g1(-0.89504)

- egu:105041933

- Down regulated genes

c165685\_g1(-0.89504)

- egu:105041933

- Down regulated genes

c165685\_g1(-0.89504)

- egu:105041933

- Down regulated genes

c165685\_g1(-0.89504)

- egu:105041933

- Down regulated genes

c165685\_g1(-0.89504)

- egu:105061098

- Down regulated genes

c164952\_g6(-0.86221)

- egu:105046469

- Down regulated genes

c160923\_g1(-0.61624)
- egu:105036836

- Down regulated genes

c159804\_g1(-0.73249)

- egu:105046469

- Down regulated genes

c160923\_g1(-0.61624)
- egu:105036836

- Down regulated genes

c159804\_g1(-0.73249)

- egu:105046469

- Down regulated genes

c160923\_g1(-0.61624)
- egu:105036836

- Down regulated genes

c159804\_g1(-0.73249)

- egu:105046469

- Down regulated genes

c160923\_g1(-0.61624)
- egu:105036836

- Down regulated genes

c159804\_g1(-0.73249)

- egu:105046469

- Down regulated genes

c160923\_g1(-0.61624)
- egu:105036836

- Down regulated genes

c159804\_g1(-0.73249)

- egu:105046469

- Down regulated genes

c160923\_g1(-0.61624)
- egu:105036836

- Down regulated genes

c159804\_g1(-0.73249)

- egu:105046469

- Down regulated genes

c160923\_g1(-0.61624)
- egu:105036836

- Down regulated genes

c159804\_g1(-0.73249)

- egu:105046469

- Down regulated genes

c160923\_g1(-0.61624)
- egu:105036836

- Down regulated genes

c159804\_g1(-0.73249)

- egu:105046469

- Down regulated genes

c160923\_g1(-0.61624)
- egu:105036836

- Down regulated genes

c159804\_g1(-0.73249)

- egu:105046469

- Down regulated genes

c160923\_g1(-0.61624)
- egu:105036836

- Down regulated genes

c159804\_g1(-0.73249)

- egu:105046469

- Down regulated genes

c160923\_g1(-0.61624)
- egu:105036836

- Down regulated genes

c159804\_g1(-0.73249)

- egu:105046469

- Down regulated genes

c160923\_g1(-0.61624)
- egu:105036836

- Down regulated genes

c159804\_g1(-0.73249)

- egu:105046469

- Down regulated genes

c160923\_g1(-0.61624)
- egu:105036836

- Down regulated genes

c159804\_g1(-0.73249)

- egu:105046469

- Down regulated genes

c160923\_g1(-0.61624)
- egu:105036836

- Down regulated genes

c159804\_g1(-0.73249)

- egu:105046469

- Down regulated genes

c160923\_g1(-0.61624)
- egu:105036836

- Down regulated genes

c159804\_g1(-0.73249)

- egu:105046469

- Down regulated genes

c160923\_g1(-0.61624)
- egu:105036836

- Down regulated genes

c159804\_g1(-0.73249)

- egu:105046469

- Down regulated genes

c160923\_g1(-0.61624)
- egu:105036836

- Down regulated genes

c159804\_g1(-0.73249)

- egu:105046469

- Down regulated genes

c160923\_g1(-0.61624)
- egu:105036836

- Down regulated genes

c159804\_g1(-0.73249)

- egu:105046469

- Down regulated genes

c160923\_g1(-0.61624)
- egu:105036836

- Down regulated genes

c159804\_g1(-0.73249)

- egu:105046469

- Down regulated genes

c160923\_g1(-0.61624)
- egu:105036836

- Down regulated genes

c159804\_g1(-0.73249)

- egu:105046469

- Down regulated genes

c160923\_g1(-0.61624)
- egu:105036836

- Down regulated genes

c159804\_g1(-0.73249)

- egu:105046469

- Down regulated genes

c160923\_g1(-0.61624)
- egu:105036836

- Down regulated genes

c159804\_g1(-0.73249)

- egu:105051428

- Down regulated genes

c153630\_g1(-0.76725)

- egu:105049882

- Down regulated genes

c71483\_g1(-0.84471)

- egu:105049882

- Down regulated genes

c71483\_g1(-0.84471)

- egu:105049882

- Down regulated genes

c71483\_g1(-0.84471)

- egu:105049882

- Down regulated genes

c71483\_g1(-0.84471)

- egu:105049882

- Down regulated genes

c71483\_g1(-0.84471)

- egu:105049882

- Down regulated genes

c71483\_g1(-0.84471)

- egu:105049882

- Down regulated genes

c71483\_g1(-0.84471)

- egu:105049882

- Down regulated genes

c71483\_g1(-0.84471)

- egu:105049882

- Down regulated genes

c71483\_g1(-0.84471)

- egu:105049882

- Down regulated genes

c71483\_g1(-0.84471)

- egu:105049882

- Down regulated genes

c71483\_g1(-0.84471)

- egu:105049882

- Down regulated genes

c71483\_g1(-0.84471)

- egu:105049882

- Down regulated genes

c71483\_g1(-0.84471)

- egu:105049882

- Down regulated genes

c71483\_g1(-0.84471)

- egu:105049882

- Down regulated genes

c71483\_g1(-0.84471)

- egu:105049882

- Down regulated genes

c71483\_g1(-0.84471)

- egu:105049882

- Down regulated genes

c71483\_g1(-0.84471)

- egu:105049882

- Down regulated genes

c71483\_g1(-0.84471)

- egu:105049882

- Down regulated genes

c71483\_g1(-0.84471)

- egu:105049882

- Down regulated genes

c71483\_g1(-0.84471)

- egu:105049882

- Down regulated genes

c71483\_g1(-0.84471)

- egu:105049882

- Down regulated genes

c71483\_g1(-0.84471)

- egu:105040827

- Down regulated genes

c147625\_g1(-0.82125)
- egu:105047380

- Down regulated genes

c157902\_g1(-0.6411)

- egu:105035926

- Down regulated genes

c163701\_g1(-1.3044)
- egu:105059577

- Down regulated genes

c132497\_g1(-1.3436)

- egu:105046233

- Down regulated genes

c163288\_g1(-0.70063)

- egu:105040792

- Down regulated genes

c156962\_g1(-0.85102)

- egu:105060929

- Down regulated genes

c132251\_g1(-0.6859)
- egu:105052838

- Down regulated genes

c174151\_g1(-1.0054)

- egu:105060929

- Down regulated genes

c132251\_g1(-0.6859)
- egu:105052838

- Down regulated genes

c174151\_g1(-1.0054)

- egu:105060929

- Down regulated genes

c132251\_g1(-0.6859)
- egu:105052838

- Down regulated genes

c174151\_g1(-1.0054)

- egu:105060929

- Down regulated genes

c132251\_g1(-0.6859)
- egu:105052838

- Down regulated genes

c174151\_g1(-1.0054)

- egu:105060929

- Down regulated genes

c132251\_g1(-0.6859)
- egu:105052838

- Down regulated genes

c174151\_g1(-1.0054)

- egu:105060929

- Down regulated genes

c132251\_g1(-0.6859)
- egu:105052838

- Down regulated genes

c174151\_g1(-1.0054)

- egu:105060929

- Down regulated genes

c132251\_g1(-0.6859)
- egu:105052838

- Down regulated genes

c174151\_g1(-1.0054)

- egu:105060929

- Down regulated genes

c132251\_g1(-0.6859)
- egu:105052838

- Down regulated genes

c174151\_g1(-1.0054)

- egu:105060929

- Down regulated genes

c132251\_g1(-0.6859)
- egu:105052838

- Down regulated genes

c174151\_g1(-1.0054)

- egu:105060929

- Down regulated genes

c132251\_g1(-0.6859)
- egu:105052838

- Down regulated genes

c174151\_g1(-1.0054)

- egu:105060929

- Down regulated genes

c132251\_g1(-0.6859)
- egu:105052838

- Down regulated genes

c174151\_g1(-1.0054)

- egu:105056640

- Down regulated genes

c134111\_g1(-1.286)

- egu:105059758

- Down regulated genes

c169453\_g2(-1.0615)
- egu:105045006

- Down regulated genes

c171099\_g1(-0.60519)

- egu:105059758

- Down regulated genes

c169453\_g2(-1.0615)
- egu:105045006

- Down regulated genes

c171099\_g1(-0.60519)

- egu:105059758

- Down regulated genes

c169453\_g2(-1.0615)
- egu:105045006

- Down regulated genes

c171099\_g1(-0.60519)

- egu:105059758

- Down regulated genes

c169453\_g2(-1.0615)
- egu:105045006

- Down regulated genes

c171099\_g1(-0.60519)

- egu:105059758

- Down regulated genes

c169453\_g2(-1.0615)
- egu:105045006

- Down regulated genes

c171099\_g1(-0.60519)

- egu:105059758

- Down regulated genes

c169453\_g2(-1.0615)
- egu:105045006

- Down regulated genes

c171099\_g1(-0.60519)

- egu:105059758

- Down regulated genes

c169453\_g2(-1.0615)
- egu:105045006

- Down regulated genes

c171099\_g1(-0.60519)

- egu:105059758

- Down regulated genes

c169453\_g2(-1.0615)
- egu:105045006

- Down regulated genes

c171099\_g1(-0.60519)

- egu:105059758

- Down regulated genes

c169453\_g2(-1.0615)
- egu:105045006

- Down regulated genes

c171099\_g1(-0.60519)

- egu:105059758

- Down regulated genes

c169453\_g2(-1.0615)
- egu:105045006

- Down regulated genes

c171099\_g1(-0.60519)

- egu:105059758

- Down regulated genes

c169453\_g2(-1.0615)
- egu:105045006

- Down regulated genes

c171099\_g1(-0.60519)

- egu:105059758

- Down regulated genes

c169453\_g2(-1.0615)
- egu:105045006

- Down regulated genes

c171099\_g1(-0.60519)

- egu:105059758

- Down regulated genes

c169453\_g2(-1.0615)
- egu:105045006

- Down regulated genes

c171099\_g1(-0.60519)

- egu:105059758

- Down regulated genes

c169453\_g2(-1.0615)
- egu:105045006

- Down regulated genes

c171099\_g1(-0.60519)

- egu:105059758

- Down regulated genes

c169453\_g2(-1.0615)
- egu:105045006

- Down regulated genes

c171099\_g1(-0.60519)

- egu:105059758

- Down regulated genes

c169453\_g2(-1.0615)
- egu:105045006

- Down regulated genes

c171099\_g1(-0.60519)

- egu:105059758

- Down regulated genes

c169453\_g2(-1.0615)
- egu:105045006

- Down regulated genes

c171099\_g1(-0.60519)

- egu:105059758

- Down regulated genes

c169453\_g2(-1.0615)
- egu:105045006

- Down regulated genes

c171099\_g1(-0.60519)

- egu:105059758

- Down regulated genes

c169453\_g2(-1.0615)
- egu:105045006

- Down regulated genes

c171099\_g1(-0.60519)

- egu:105059758

- Down regulated genes

c169453\_g2(-1.0615)
- egu:105045006

- Down regulated genes

c171099\_g1(-0.60519)

- egu:105059758

- Down regulated genes

c169453\_g2(-1.0615)
- egu:105045006

- Down regulated genes

c171099\_g1(-0.60519)

- egu:105059758

- Down regulated genes

c169453\_g2(-1.0615)
- egu:105045006

- Down regulated genes

c171099\_g1(-0.60519)

- egu:105034723

- Down regulated genes

c169294\_g2(-0.85273)
- egu:105059287

- Down regulated genes

c163496\_g1(-0.6363)
- egu:105034969

- Down regulated genes

c113371\_g2(-0.73611)

- egu:105048201

- Down regulated genes

c171050\_g1(-0.65915)

- egu:105039219

- Down regulated genes

c163642\_g1(-0.73792)

- egu:105041436

- Down regulated genes

c162392\_g1(-1.1431) c146091\_g1(-1.5306)

- egu:105034557

- Down regulated genes

c104889\_g1(-1.7144)
- egu:105054530

- Down regulated genes

c104889\_g2(-1.5635) c174574\_g3(-3.7395)

- egu:105034557

- Down regulated genes

c104889\_g1(-1.7144)
- egu:105054530

- Down regulated genes

c104889\_g2(-1.5635) c174574\_g3(-3.7395)

- egu:105034557

- Down regulated genes

c104889\_g1(-1.7144)
- egu:105054530

- Down regulated genes

c104889\_g2(-1.5635) c174574\_g3(-3.7395)

- egu:105034557

- Down regulated genes

c104889\_g1(-1.7144)
- egu:105054530

- Down regulated genes

c104889\_g2(-1.5635) c174574\_g3(-3.7395)

- egu:105034557

- Down regulated genes

c104889\_g1(-1.7144)
- egu:105054530

- Down regulated genes

c104889\_g2(-1.5635) c174574\_g3(-3.7395)

- egu:105034557

- Down regulated genes

c104889\_g1(-1.7144)
- egu:105054530

- Down regulated genes

c104889\_g2(-1.5635) c174574\_g3(-3.7395)

- egu:105034557

- Down regulated genes

c104889\_g1(-1.7144)
- egu:105054530

- Down regulated genes

c104889\_g2(-1.5635) c174574\_g3(-3.7395)

- egu:105034557

- Down regulated genes

c104889\_g1(-1.7144)
- egu:105054530

- Down regulated genes

c104889\_g2(-1.5635) c174574\_g3(-3.7395)

- egu:105034557

- Down regulated genes

c104889\_g1(-1.7144)
- egu:105054530

- Down regulated genes

c104889\_g2(-1.5635) c174574\_g3(-3.7395)

- egu:105034557

- Down regulated genes

c104889\_g1(-1.7144)
- egu:105054530

- Down regulated genes

c104889\_g2(-1.5635) c174574\_g3(-3.7395)

- egu:105034557

- Down regulated genes

c104889\_g1(-1.7144)
- egu:105054530

- Down regulated genes

c104889\_g2(-1.5635) c174574\_g3(-3.7395)

- egu:105050625

- Down regulated genes

c162112\_g2(-0.95192)

- egu:105050625

- Down regulated genes

c162112\_g2(-0.95192)

- egu:105050625

- Down regulated genes

c162112\_g2(-0.95192)

- egu:105050625

- Down regulated genes

c162112\_g2(-0.95192)

- egu:105050625

- Down regulated genes

c162112\_g2(-0.95192)

- egu:105050625

- Down regulated genes

c162112\_g2(-0.95192)

- egu:105050625

- Down regulated genes

c162112\_g2(-0.95192)

- egu:105050625

- Down regulated genes

c162112\_g2(-0.95192)

- egu:105050625

- Down regulated genes

c162112\_g2(-0.95192)

- egu:105050625

- Down regulated genes

c162112\_g2(-0.95192)

- egu:105050625

- Down regulated genes

c162112\_g2(-0.95192)

- egu:105050625

- Down regulated genes

c162112\_g2(-0.95192)

- egu:105050625

- Down regulated genes

c162112\_g2(-0.95192)

- egu:105050625

- Down regulated genes

c162112\_g2(-0.95192)

- egu:105050625

- Down regulated genes

c162112\_g2(-0.95192)

- egu:105050625

- Down regulated genes

c162112\_g2(-0.95192)

- egu:105050625

- Down regulated genes

c162112\_g2(-0.95192)

- egu:105050625

- Down regulated genes

c162112\_g2(-0.95192)

- egu:105050625

- Down regulated genes

c162112\_g2(-0.95192)

- egu:105050625

- Down regulated genes

c162112\_g2(-0.95192)

- egu:105050625

- Down regulated genes

c162112\_g2(-0.95192)

- egu:105050625

- Down regulated genes

c162112\_g2(-0.95192)

- egu:105035321

- Down regulated genes

c154502\_g4(-1.1888)
- egu:105049380

- Down regulated genes

c85645\_g1(-1.0533)

- egu:105060694

- Down regulated genes

c133070\_g1(-0.83686)

- egu:105060694

- Down regulated genes

c133070\_g1(-0.83686)

- egu:105060694

- Down regulated genes

c133070\_g1(-0.83686)

- egu:105060694

- Down regulated genes

c133070\_g1(-0.83686)

- egu:105060694

- Down regulated genes

c133070\_g1(-0.83686)

- egu:105060694

- Down regulated genes

c133070\_g1(-0.83686)

- egu:105060694

- Down regulated genes

c133070\_g1(-0.83686)

- egu:105060694

- Down regulated genes

c133070\_g1(-0.83686)

- egu:105060694

- Down regulated genes

c133070\_g1(-0.83686)

- egu:105060694

- Down regulated genes

c133070\_g1(-0.83686)

- egu:105060694

- Down regulated genes

c133070\_g1(-0.83686)

- egu:105060694

- Down regulated genes

c133070\_g1(-0.83686)

- egu:105060694

- Down regulated genes

c133070\_g1(-0.83686)

- egu:105057517

- Down regulated genes

c147541\_g1(-0.99782)
- egu:105032039

- Down regulated genes

c154303\_g1(-1.1463)

- egu:105048107

- Down regulated genes

c159323\_g1(-0.98431)
- egu:105040940

- Down regulated genes

c163118\_g1(-1.775)

- egu:105048107

- Down regulated genes

c159323\_g1(-0.98431)
- egu:105040940

- Down regulated genes

c163118\_g1(-1.775)

- egu:105059896

- Down regulated genes

c127525\_g1(-1.98)
- egu:105037657

- Down regulated genes

c165472\_g1(-1.1637)

- egu:105044629

- Down regulated genes

c156209\_g1(-1.376)
- egu:105052647

- Down regulated genes

c155934\_g1(-3.5798)
- egu:105042952

- Down regulated genes

c101133\_g1(-4.2291)

- egu:105042425

- Down regulated genes

c166887\_g5(-1.2344)
- egu:105042390

- Down regulated genes

c173060\_g2(-1.0904)
- egu:105034542

- Down regulated genes

c174706\_g1(-1.4093)

- egu:105059896

- Down regulated genes

c127525\_g1(-1.98)
- egu:105037657

- Down regulated genes

c165472\_g1(-1.1637)

- egu:105045448

- Down regulated genes

c171016\_g1(-1.9038)
- egu:105053765

- Down regulated genes

c168470\_g1(-2.7871)

- egu:105045448

- Down regulated genes

c171016\_g1(-1.9038)
- egu:105053765

- Down regulated genes

c168470\_g1(-2.7871)

- egu:105045448

- Down regulated genes

c171016\_g1(-1.9038)
- egu:105053765

- Down regulated genes

c168470\_g1(-2.7871)

- egu:105045448

- Down regulated genes

c171016\_g1(-1.9038)
- egu:105053765

- Down regulated genes

c168470\_g1(-2.7871)

- egu:105045448

- Down regulated genes

c171016\_g1(-1.9038)
- egu:105053765

- Down regulated genes

c168470\_g1(-2.7871)

- egu:105045448

- Down regulated genes

c171016\_g1(-1.9038)
- egu:105053765

- Down regulated genes

c168470\_g1(-2.7871)

- egu:105045448

- Down regulated genes

c171016\_g1(-1.9038)
- egu:105053765

- Down regulated genes

c168470\_g1(-2.7871)

- egu:105045448

- Down regulated genes

c171016\_g1(-1.9038)
- egu:105053765

- Down regulated genes

c168470\_g1(-2.7871)

- egu:105045448

- Down regulated genes

c171016\_g1(-1.9038)
- egu:105053765

- Down regulated genes

c168470\_g1(-2.7871)

- egu:105045448

- Down regulated genes

c171016\_g1(-1.9038)
- egu:105053765

- Down regulated genes

c168470\_g1(-2.7871)

- egu:105045448

- Down regulated genes

c171016\_g1(-1.9038)
- egu:105053765

- Down regulated genes

c168470\_g1(-2.7871)

- egu:105045448

- Down regulated genes

c171016\_g1(-1.9038)
- egu:105053765

- Down regulated genes

c168470\_g1(-2.7871)

- egu:105045448

- Down regulated genes

c171016\_g1(-1.9038)
- egu:105053765

- Down regulated genes

c168470\_g1(-2.7871)

- egu:105045448

- Down regulated genes

c171016\_g1(-1.9038)
- egu:105053765

- Down regulated genes

c168470\_g1(-2.7871)

- egu:105045448

- Down regulated genes

c171016\_g1(-1.9038)
- egu:105053765

- Down regulated genes

c168470\_g1(-2.7871)

- egu:105045448

- Down regulated genes

c171016\_g1(-1.9038)
- egu:105053765

- Down regulated genes

c168470\_g1(-2.7871)

- egu:105045448

- Down regulated genes

c171016\_g1(-1.9038)
- egu:105053765

- Down regulated genes

c168470\_g1(-2.7871)

- egu:105045448

- Down regulated genes

c171016\_g1(-1.9038)
- egu:105053765

- Down regulated genes

c168470\_g1(-2.7871)

- egu:105045448

- Down regulated genes

c171016\_g1(-1.9038)
- egu:105053765

- Down regulated genes

c168470\_g1(-2.7871)

- egu:105045448

- Down regulated genes

c171016\_g1(-1.9038)
- egu:105053765

- Down regulated genes

c168470\_g1(-2.7871)

- egu:105045448

- Down regulated genes

c171016\_g1(-1.9038)
- egu:105053765

- Down regulated genes

c168470\_g1(-2.7871)

- egu:105045448

- Down regulated genes

c171016\_g1(-1.9038)
- egu:105053765

- Down regulated genes

c168470\_g1(-2.7871)

- egu:105045448

- Down regulated genes

c171016\_g1(-1.9038)
- egu:105053765

- Down regulated genes

c168470\_g1(-2.7871)

- egu:105045448

- Down regulated genes

c171016\_g1(-1.9038)
- egu:105053765

- Down regulated genes

c168470\_g1(-2.7871)

- egu:105045448

- Down regulated genes

c171016\_g1(-1.9038)
- egu:105053765

- Down regulated genes

c168470\_g1(-2.7871)

- egu:105045448

- Down regulated genes

c171016\_g1(-1.9038)
- egu:105053765

- Down regulated genes

c168470\_g1(-2.7871)

- egu:105045448

- Down regulated genes

c171016\_g1(-1.9038)
- egu:105053765

- Down regulated genes

c168470\_g1(-2.7871)

- egu:105045448

- Down regulated genes

c171016\_g1(-1.9038)
- egu:105053765

- Down regulated genes

c168470\_g1(-2.7871)

- egu:105045448

- Down regulated genes

c171016\_g1(-1.9038)
- egu:105053765

- Down regulated genes

c168470\_g1(-2.7871)

- egu:105045448

- Down regulated genes

c171016\_g1(-1.9038)
- egu:105053765

- Down regulated genes

c168470\_g1(-2.7871)

- egu:105045448

- Down regulated genes

c171016\_g1(-1.9038)
- egu:105053765

- Down regulated genes

c168470\_g1(-2.7871)

- egu:105045448

- Down regulated genes

c171016\_g1(-1.9038)
- egu:105053765

- Down regulated genes

c168470\_g1(-2.7871)

- egu:105045448

- Down regulated genes

c171016\_g1(-1.9038)
- egu:105053765

- Down regulated genes

c168470\_g1(-2.7871)

- egu:105045448

- Down regulated genes

c171016\_g1(-1.9038)
- egu:105053765

- Down regulated genes

c168470\_g1(-2.7871)

- egu:105045448

- Down regulated genes

c171016\_g1(-1.9038)
- egu:105053765

- Down regulated genes

c168470\_g1(-2.7871)

- egu:105045448

- Down regulated genes

c171016\_g1(-1.9038)
- egu:105053765

- Down regulated genes

c168470\_g1(-2.7871)

- egu:105045448

- Down regulated genes

c171016\_g1(-1.9038)
- egu:105053765

- Down regulated genes

c168470\_g1(-2.7871)

- egu:105045448

- Down regulated genes

c171016\_g1(-1.9038)
- egu:105053765

- Down regulated genes

c168470\_g1(-2.7871)

- egu:105045448

- Down regulated genes

c171016\_g1(-1.9038)
- egu:105053765

- Down regulated genes

c168470\_g1(-2.7871)

- egu:105045448

- Down regulated genes

c171016\_g1(-1.9038)
- egu:105053765

- Down regulated genes

c168470\_g1(-2.7871)

- egu:105045448

- Down regulated genes

c171016\_g1(-1.9038)
- egu:105053765

- Down regulated genes

c168470\_g1(-2.7871)

- egu:105045448

- Down regulated genes

c171016\_g1(-1.9038)
- egu:105053765

- Down regulated genes

c168470\_g1(-2.7871)

- egu:105045448

- Down regulated genes

c171016\_g1(-1.9038)
- egu:105053765

- Down regulated genes

c168470\_g1(-2.7871)

- egu:105045448

- Down regulated genes

c171016\_g1(-1.9038)
- egu:105053765

- Down regulated genes

c168470\_g1(-2.7871)

- egu:105059896

- Down regulated genes

c127525\_g1(-1.98)
- egu:105037657

- Down regulated genes

c165472\_g1(-1.1637)

- egu:105044629

- Down regulated genes

c156209\_g1(-1.376)
- egu:105052647

- Down regulated genes

c155934\_g1(-3.5798)
- egu:105042952

- Down regulated genes

c101133\_g1(-4.2291)

- egu:105059896

- Down regulated genes

c127525\_g1(-1.98)
- egu:105037657

- Down regulated genes

c165472\_g1(-1.1637)

- egu:105059896

- Down regulated genes

c127525\_g1(-1.98)
- egu:105037657

- Down regulated genes

c165472\_g1(-1.1637)

- egu:105032439

- Down regulated genes

c171265\_g1(-2.6191)

- egu:105044629

- Down regulated genes

c156209\_g1(-1.376)
- egu:105052647

- Down regulated genes

c155934\_g1(-3.5798)
- egu:105042952

- Down regulated genes

c101133\_g1(-4.2291)

- egu:105054281

- Down regulated genes

c141672\_g1(-1.5073)

- egu:105054281

- Down regulated genes

c141672\_g1(-1.5073)

- egu:105054281

- Down regulated genes

c141672\_g1(-1.5073)

- egu:105054281

- Down regulated genes

c141672\_g1(-1.5073)

- egu:105048107

- Down regulated genes

c159323\_g1(-0.98431)

- egu:105048107

- Down regulated genes

c159323\_g1(-0.98431)

- egu:105048107

- Down regulated genes

c159323\_g1(-0.98431)

- egu:105048107

- Down regulated genes

c159323\_g1(-0.98431)

- egu:105048107

- Down regulated genes

c159323\_g1(-0.98431)

- egu:105048107

- Down regulated genes

c159323\_g1(-0.98431)

- egu:105048107

- Down regulated genes

c159323\_g1(-0.98431)

- egu:105048107

- Down regulated genes

c159323\_g1(-0.98431)

- egu:105048107

- Down regulated genes

c159323\_g1(-0.98431)

- egu:105048107

- Down regulated genes

c159323\_g1(-0.98431)

- egu:105048107

- Down regulated genes

c159323\_g1(-0.98431)

- egu:105043264

- Down regulated genes

c152607\_g1(-0.57758)

- egu:105043264

- Down regulated genes

c152607\_g1(-0.57758)

- egu:105043264

- Down regulated genes

c152607\_g1(-0.57758)

- egu:105043264

- Down regulated genes

c152607\_g1(-0.57758)

- egu:105043264

- Down regulated genes

c152607\_g1(-0.57758)

- egu:105043264

- Down regulated genes

c152607\_g1(-0.57758)

- egu:105043264

- Down regulated genes

c152607\_g1(-0.57758)

- egu:105043264

- Down regulated genes

c152607\_g1(-0.57758)

- egu:105043264

- Down regulated genes

c152607\_g1(-0.57758)

- egu:105043264

- Down regulated genes

c152607\_g1(-0.57758)

- egu:105043264

- Down regulated genes

c152607\_g1(-0.57758)

- egu:105043264

- Down regulated genes

c152607\_g1(-0.57758)

- egu:105043264

- Down regulated genes

c152607\_g1(-0.57758)

- egu:105043264

- Down regulated genes

c152607\_g1(-0.57758)

- egu:105043264

- Down regulated genes

c152607\_g1(-0.57758)

- egu:105043264

- Down regulated genes

c152607\_g1(-0.57758)

- egu:105043264

- Down regulated genes

c152607\_g1(-0.57758)

- egu:105043264

- Down regulated genes

c152607\_g1(-0.57758)

- egu:105043264

- Down regulated genes

c152607\_g1(-0.57758)

- egu:105043264

- Down regulated genes

c152607\_g1(-0.57758)

- egu:105043264

- Down regulated genes

c152607\_g1(-0.57758)

- egu:105054281

- Down regulated genes

c141672\_g1(-1.5073)

- egu:105035984

- Down regulated genes

c167553\_g1(-0.99851)

- egu:105039425

- Down regulated genes

c159912\_g1(-2.087)
- egu:105036971

- Down regulated genes

c159912\_g2(-2.2265)

- egu:105045599

- Down regulated genes

c165075\_g1(-0.65473)

- egu:105049267

- Down regulated genes

c173904\_g1(-0.61455)

- egu:105035984

- Down regulated genes

c167553\_g1(-0.99851)

- egu:105037896

- Down regulated genes

c162518\_g1(-0.75828)

- egu:105048201

- Down regulated genes

c171050\_g1(-0.65915)
- egu:105034341

- Down regulated genes

c131571\_g1(-0.85538)

- egu:105052855

- Down regulated genes

c161205\_g1(-0.78676)

- egu:105054529

- Down regulated genes

c167947\_g1(-2.0036)

- egu:105051026

- Down regulated genes

c167743\_g1(-0.97445)

- egu:105061098

- Down regulated genes

c164952\_g6(-0.86221)

- egu:105061098

- Down regulated genes

c164952\_g6(-0.86221)

- egu:105060927

- Down regulated genes

c173971\_g3(-0.86974)

- egu:105047162

- Down regulated genes

c154629\_g1(-0.79402)

- egu:105041933

- Down regulated genes

c165685\_g1(-0.89504)

- egu:105041933

- Down regulated genes

c165685\_g1(-0.89504)

- egu:105041933

- Down regulated genes

c165685\_g1(-0.89504)

- egu:105041933

- Down regulated genes

c165685\_g1(-0.89504)

- egu:105041933

- Down regulated genes

c165685\_g1(-0.89504)

- egu:105041933

- Down regulated genes

c165685\_g1(-0.89504)

- egu:105041933

- Down regulated genes

c165685\_g1(-0.89504)

- egu:105041933

- Down regulated genes

c165685\_g1(-0.89504)

- egu:105041933

- Down regulated genes

c165685\_g1(-0.89504)

- egu:105041933

- Down regulated genes

c165685\_g1(-0.89504)

- egu:105041933

- Down regulated genes

c165685\_g1(-0.89504)

- egu:105041933

- Down regulated genes

c165685\_g1(-0.89504)

- egu:105057764

- Down regulated genes

c156235\_g1(-2.2959)

- egu:105037930

- Down regulated genes

c71670\_g1(-0.96871)

- egu:105037930

- Down regulated genes

c71670\_g1(-0.96871)

- egu:105037930

- Down regulated genes

c71670\_g1(-0.96871)

- egu:105037930

- Down regulated genes

c71670\_g1(-0.96871)

- egu:105037930

- Down regulated genes

c71670\_g1(-0.96871)

- egu:105037930

- Down regulated genes

c71670\_g1(-0.96871)

- egu:105037930

- Down regulated genes

c71670\_g1(-0.96871)

- egu:105037930

- Down regulated genes

c71670\_g1(-0.96871)

- egu:105037930

- Down regulated genes

c71670\_g1(-0.96871)

- egu:105037930

- Down regulated genes

c71670\_g1(-0.96871)

- egu:105037930

- Down regulated genes

c71670\_g1(-0.96871)

- egu:105037930

- Down regulated genes

c71670\_g1(-0.96871)

- egu:105037930

- Down regulated genes

c71670\_g1(-0.96871)

- egu:105037930

- Down regulated genes

c71670\_g1(-0.96871)

- egu:105037930

- Down regulated genes

c71670\_g1(-0.96871)

- egu:105037930

- Down regulated genes

c71670\_g1(-0.96871)

- egu:105037930

- Down regulated genes

c71670\_g1(-0.96871)

- egu:105037930

- Down regulated genes

c71670\_g1(-0.96871)

- egu:105037930

- Down regulated genes

c71670\_g1(-0.96871)

- egu:105037930

- Down regulated genes

c71670\_g1(-0.96871)

- egu:105037930

- Down regulated genes

c71670\_g1(-0.96871)

- egu:105037930

- Down regulated genes

c71670\_g1(-0.96871)

- egu:105037930

- Down regulated genes

c71670\_g1(-0.96871)

- egu:105037930

- Down regulated genes

c71670\_g1(-0.96871)

- egu:105037930

- Down regulated genes

c71670\_g1(-0.96871)

- egu:105037930

- Down regulated genes

c71670\_g1(-0.96871)

- egu:105037930

- Down regulated genes

c71670\_g1(-0.96871)

- egu:105037930

- Down regulated genes

c71670\_g1(-0.96871)

- egu:105037930

- Down regulated genes

c71670\_g1(-0.96871)

- egu:105037930

- Down regulated genes

c71670\_g1(-0.96871)

- egu:105037930

- Down regulated genes

c71670\_g1(-0.96871)

- egu:105037930

- Down regulated genes

c71670\_g1(-0.96871)

- egu:105037930

- Down regulated genes

c71670\_g1(-0.96871)

- egu:105037930

- Down regulated genes

c71670\_g1(-0.96871)

- egu:105037930

- Down regulated genes

c71670\_g1(-0.96871)

- egu:105037930

- Down regulated genes

c71670\_g1(-0.96871)

- egu:105037930

- Down regulated genes

c71670\_g1(-0.96871)

- egu:105037930

- Down regulated genes

c71670\_g1(-0.96871)

- egu:105037930

- Down regulated genes

c71670\_g1(-0.96871)

- egu:105037930

- Down regulated genes

c71670\_g1(-0.96871)

- egu:105037930

- Down regulated genes

c71670\_g1(-0.96871)

- egu:105037930

- Down regulated genes

c71670\_g1(-0.96871)

- egu:105049221

- Down regulated genes

c71809\_g1(-0.98861)

- egu:105040768

- Down regulated genes

c168519\_g1(-1.0519)
- egu:105044798

- Down regulated genes

c165450\_g1(-0.88869)

- egu:105057582

- Down regulated genes

c169028\_g1(-2.0429)
- egu:105040656

- Down regulated genes

c133188\_g1(-1.0414)

- egu:105035938

- Down regulated genes

c123480\_g1(-1.2045)

- egu:105036097

- Down regulated genes

c185147\_g2(-0.93846)

- egu:105036097

- Down regulated genes

c185147\_g2(-0.93846)

- egu:105036097

- Down regulated genes

c185147\_g2(-0.93846)

- egu:105045229

- Down regulated genes

c157598\_g1(-1.0974)

- egu:105045229

- Down regulated genes

c157598\_g1(-1.0974)

- egu:105044579

- Down regulated genes

c155686\_g1(-0.57873)

- egu:105044579

- Down regulated genes

c155686\_g1(-0.57873)

- egu:105058937

- Down regulated genes

c134136\_g1(-0.64172)

- egu:105058937

- Down regulated genes

c134136\_g1(-0.64172)

- egu:105059611

- Down regulated genes

c198353\_g1(-0.63625)
- egu:105045658

- Down regulated genes

c43883\_g1(-0.68594)

- egu:105048493

- Down regulated genes

c170305\_g2(-0.65115)

- egu:105044431

- Down regulated genes

c134778\_g1(-1.5005)

- egu:105052214

- Down regulated genes

c117604\_g1(-0.96857)

- egu:105044215

- Down regulated genes

c148702\_g1(-1.9471)
- egu:105041077

- Down regulated genes

c166224\_g1(-1.1149) c150017\_g1(-1.3389)
- egu:105039895

- Down regulated genes

c134603\_g2(-1.9882)
- egu:105048315

- Down regulated genes

c164323\_g1(-0.74079)
- egu:105036065

- Down regulated genes

c163278\_g2(-3.3645)
- egu:105047165

- Down regulated genes

c164323\_g2(-0.67365)

- egu:105044215

- Down regulated genes

c148702\_g1(-1.9471)
- egu:105041077

- Down regulated genes

c166224\_g1(-1.1149) c150017\_g1(-1.3389)
- egu:105039895

- Down regulated genes

c134603\_g2(-1.9882)
- egu:105048315

- Down regulated genes

c164323\_g1(-0.74079)
- egu:105036065

- Down regulated genes

c163278\_g2(-3.3645)
- egu:105047165

- Down regulated genes

c164323\_g2(-0.67365)

- egu:105044215

- Down regulated genes

c148702\_g1(-1.9471)
- egu:105041077

- Down regulated genes

c166224\_g1(-1.1149) c150017\_g1(-1.3389)
- egu:105039895

- Down regulated genes

c134603\_g2(-1.9882)
- egu:105048315

- Down regulated genes

c164323\_g1(-0.74079)
- egu:105036065

- Down regulated genes

c163278\_g2(-3.3645)
- egu:105047165

- Down regulated genes

c164323\_g2(-0.67365)

- egu:105044215

- Down regulated genes

c148702\_g1(-1.9471)
- egu:105041077

- Down regulated genes

c166224\_g1(-1.1149) c150017\_g1(-1.3389)
- egu:105039895

- Down regulated genes

c134603\_g2(-1.9882)
- egu:105048315

- Down regulated genes

c164323\_g1(-0.74079)
- egu:105036065

- Down regulated genes

c163278\_g2(-3.3645)
- egu:105047165

- Down regulated genes

c164323\_g2(-0.67365)

- egu:105044215

- Down regulated genes

c148702\_g1(-1.9471)
- egu:105041077

- Down regulated genes

c166224\_g1(-1.1149) c150017\_g1(-1.3389)
- egu:105039895

- Down regulated genes

c134603\_g2(-1.9882)
- egu:105048315

- Down regulated genes

c164323\_g1(-0.74079)
- egu:105036065

- Down regulated genes

c163278\_g2(-3.3645)
- egu:105047165

- Down regulated genes

c164323\_g2(-0.67365)

- egu:105044215

- Down regulated genes

c148702\_g1(-1.9471)
- egu:105041077

- Down regulated genes

c166224\_g1(-1.1149) c150017\_g1(-1.3389)
- egu:105039895

- Down regulated genes

c134603\_g2(-1.9882)
- egu:105048315

- Down regulated genes

c164323\_g1(-0.74079)
- egu:105036065

- Down regulated genes

c163278\_g2(-3.3645)
- egu:105047165

- Down regulated genes

c164323\_g2(-0.67365)

- egu:105044215

- Down regulated genes

c148702\_g1(-1.9471)
- egu:105041077

- Down regulated genes

c166224\_g1(-1.1149) c150017\_g1(-1.3389)
- egu:105039895

- Down regulated genes

c134603\_g2(-1.9882)
- egu:105048315

- Down regulated genes

c164323\_g1(-0.74079)
- egu:105036065

- Down regulated genes

c163278\_g2(-3.3645)
- egu:105047165

- Down regulated genes

c164323\_g2(-0.67365)

- egu:105044215

- Down regulated genes

c148702\_g1(-1.9471)
- egu:105041077

- Down regulated genes

c166224\_g1(-1.1149) c150017\_g1(-1.3389)
- egu:105039895

- Down regulated genes

c134603\_g2(-1.9882)
- egu:105048315

- Down regulated genes

c164323\_g1(-0.74079)
- egu:105036065

- Down regulated genes

c163278\_g2(-3.3645)
- egu:105047165

- Down regulated genes

c164323\_g2(-0.67365)

- egu:105044215

- Down regulated genes

c148702\_g1(-1.9471)
- egu:105041077

- Down regulated genes

c166224\_g1(-1.1149) c150017\_g1(-1.3389)
- egu:105039895

- Down regulated genes

c134603\_g2(-1.9882)
- egu:105048315

- Down regulated genes

c164323\_g1(-0.74079)
- egu:105036065

- Down regulated genes

c163278\_g2(-3.3645)
- egu:105047165

- Down regulated genes

c164323\_g2(-0.67365)

- egu:105044215

- Down regulated genes

c148702\_g1(-1.9471)
- egu:105041077

- Down regulated genes

c166224\_g1(-1.1149) c150017\_g1(-1.3389)
- egu:105039895

- Down regulated genes

c134603\_g2(-1.9882)
- egu:105048315

- Down regulated genes

c164323\_g1(-0.74079)
- egu:105036065

- Down regulated genes

c163278\_g2(-3.3645)
- egu:105047165

- Down regulated genes

c164323\_g2(-0.67365)

- egu:105044215

- Down regulated genes

c148702\_g1(-1.9471)
- egu:105041077

- Down regulated genes

c166224\_g1(-1.1149) c150017\_g1(-1.3389)
- egu:105039895

- Down regulated genes

c134603\_g2(-1.9882)
- egu:105048315

- Down regulated genes

c164323\_g1(-0.74079)
- egu:105036065

- Down regulated genes

c163278\_g2(-3.3645)
- egu:105047165

- Down regulated genes

c164323\_g2(-0.67365)

- egu:105044215

- Down regulated genes

c148702\_g1(-1.9471)
- egu:105041077

- Down regulated genes

c166224\_g1(-1.1149) c150017\_g1(-1.3389)
- egu:105039895

- Down regulated genes

c134603\_g2(-1.9882)
- egu:105048315

- Down regulated genes

c164323\_g1(-0.74079)
- egu:105036065

- Down regulated genes

c163278\_g2(-3.3645)
- egu:105047165

- Down regulated genes

c164323\_g2(-0.67365)

- egu:105038852

- Down regulated genes

c137214\_g2(-0.95999)

- egu:105058894

- Down regulated genes

c159032\_g1(-0.69317)

- egu:105058894

- Down regulated genes

c159032\_g1(-0.69317)

- egu:105038832

- Down regulated genes

c166072\_g1(-1.2661)

- egu:105038832

- Down regulated genes

c166072\_g1(-1.2661)

- egu:105038832

- Down regulated genes

c166072\_g1(-1.2661)

- egu:105038832

- Down regulated genes

c166072\_g1(-1.2661)

- egu:105038832

- Down regulated genes

c166072\_g1(-1.2661)

- egu:105038832

- Down regulated genes

c166072\_g1(-1.2661)

- egu:105038832

- Down regulated genes

c166072\_g1(-1.2661)

- egu:105038832

- Down regulated genes

c166072\_g1(-1.2661)

- egu:105038832

- Down regulated genes

c166072\_g1(-1.2661)

- egu:105038832

- Down regulated genes

c166072\_g1(-1.2661)

- egu:105038832

- Down regulated genes

c166072\_g1(-1.2661)

- egu:105038832

- Down regulated genes

c166072\_g1(-1.2661)

- egu:105038832

- Down regulated genes

c166072\_g1(-1.2661)

- egu:105038832

- Down regulated genes

c166072\_g1(-1.2661)

- egu:105038832

- Down regulated genes

c166072\_g1(-1.2661)

- egu:105038832

- Down regulated genes

c166072\_g1(-1.2661)

- egu:105038832

- Down regulated genes

c166072\_g1(-1.2661)

- egu:105038832

- Down regulated genes

c166072\_g1(-1.2661)

- egu:105038832

- Down regulated genes

c166072\_g1(-1.2661)

- egu:105038832

- Down regulated genes

c166072\_g1(-1.2661)

- egu:105038832

- Down regulated genes

c166072\_g1(-1.2661)

- egu:105038832

- Down regulated genes

c166072\_g1(-1.2661)

- egu:105038832

- Down regulated genes

c166072\_g1(-1.2661)

- egu:105038832

- Down regulated genes

c166072\_g1(-1.2661)

- egu:105038832

- Down regulated genes

c166072\_g1(-1.2661)

- egu:105038832

- Down regulated genes

c166072\_g1(-1.2661)

- egu:105038832

- Down regulated genes

c166072\_g1(-1.2661)

- egu:105038832

- Down regulated genes

c166072\_g1(-1.2661)

- egu:105038832

- Down regulated genes

c166072\_g1(-1.2661)

- egu:105038832

- Down regulated genes

c166072\_g1(-1.2661)

- egu:105038832

- Down regulated genes

c166072\_g1(-1.2661)

- egu:105038832

- Down regulated genes

c166072\_g1(-1.2661)

- egu:105038832

- Down regulated genes

c166072\_g1(-1.2661)

- egu:105038832

- Down regulated genes

c166072\_g1(-1.2661)

- egu:105038832

- Down regulated genes

c166072\_g1(-1.2661)

- egu:105038832

- Down regulated genes

c166072\_g1(-1.2661)

- egu:105038832

- Down regulated genes

c166072\_g1(-1.2661)

- egu:105038832

- Down regulated genes

c166072\_g1(-1.2661)

- egu:105038832

- Down regulated genes

c166072\_g1(-1.2661)

- egu:105038832

- Down regulated genes

c166072\_g1(-1.2661)

- egu:105038832

- Down regulated genes

c166072\_g1(-1.2661)

- egu:105038832

- Down regulated genes

c166072\_g1(-1.2661)

- egu:105058545

- Down regulated genes

c166557\_g2(-1.4097) c166557\_g1(-1.5029)

- egu:105058545

- Down regulated genes

c166557\_g2(-1.4097) c166557\_g1(-1.5029)

- egu:105058545

- Down regulated genes

c166557\_g2(-1.4097) c166557\_g1(-1.5029)

- egu:105058545

- Down regulated genes

c166557\_g2(-1.4097) c166557\_g1(-1.5029)

- egu:105058545

- Down regulated genes

c166557\_g2(-1.4097) c166557\_g1(-1.5029)

- egu:105058545

- Down regulated genes

c166557\_g2(-1.4097) c166557\_g1(-1.5029)

- egu:105058545

- Down regulated genes

c166557\_g2(-1.4097) c166557\_g1(-1.5029)

- egu:105058545

- Down regulated genes

c166557\_g2(-1.4097) c166557\_g1(-1.5029)

- egu:105058545

- Down regulated genes

c166557\_g2(-1.4097) c166557\_g1(-1.5029)

- egu:105058545

- Down regulated genes

c166557\_g2(-1.4097) c166557\_g1(-1.5029)

- egu:105058545

- Down regulated genes

c166557\_g2(-1.4097) c166557\_g1(-1.5029)

- egu:105042425

- Down regulated genes

c166887\_g5(-1.2344)
- egu:105042390

- Down regulated genes

c173060\_g2(-1.0904)
- egu:105034542

- Down regulated genes

c174706\_g1(-1.4093)

- egu:105043222

- Down regulated genes

c155524\_g1(-2.4289)

- egu:105043222

- Down regulated genes

c155524\_g1(-2.4289)

- egu:105043222

- Down regulated genes

c155524\_g1(-2.4289)

- egu:105061098

- Down regulated genes

c164952\_g6(-0.86221)

- egu:105061098

- Down regulated genes

c164952\_g6(-0.86221)

- egu:105061098

- Down regulated genes

c164952\_g6(-0.86221)

- egu:105061098

- Down regulated genes

c164952\_g6(-0.86221)

- egu:105061098

- Down regulated genes

c164952\_g6(-0.86221)

- egu:105061098

- Down regulated genes

c164952\_g6(-0.86221)

- egu:105061098

- Down regulated genes

c164952\_g6(-0.86221)

- egu:105061098

- Down regulated genes

c164952\_g6(-0.86221)

- egu:105061098

- Down regulated genes

c164952\_g6(-0.86221)

- egu:105061098

- Down regulated genes

c164952\_g6(-0.86221)

- egu:105061098

- Down regulated genes

c164952\_g6(-0.86221)

- egu:105061098

- Down regulated genes

c164952\_g6(-0.86221)

- egu:105061098

- Down regulated genes

c164952\_g6(-0.86221)

- egu:105061098

- Down regulated genes

c164952\_g6(-0.86221)

- egu:105061098

- Down regulated genes

c164952\_g6(-0.86221)

- egu:105061098

- Down regulated genes

c164952\_g6(-0.86221)

- egu:105061098

- Down regulated genes

c164952\_g6(-0.86221)

- egu:105061098

- Down regulated genes

c164952\_g6(-0.86221)

- egu:105061098

- Down regulated genes

c164952\_g6(-0.86221)

- egu:105061098

- Down regulated genes

c164952\_g6(-0.86221)

- egu:105061098

- Down regulated genes

c164952\_g6(-0.86221)

- egu:105054147

- Down regulated genes

c125057\_g1(-0.74404)
- egu:105046827

- Down regulated genes

c166358\_g1(-1.1126)

- egu:105036454

- Down regulated genes

c164056\_g1(-0.67092)

- egu:105032472

- Down regulated genes

c171401\_g2(-0.65414)

- egu:105032472

- Down regulated genes

c171401\_g2(-0.65414)

- egu:105032472

- Down regulated genes

c171401\_g2(-0.65414)

- egu:105032472

- Down regulated genes

c171401\_g2(-0.65414)

- egu:105032472

- Down regulated genes

c171401\_g2(-0.65414)

- egu:105032472

- Down regulated genes

c171401\_g2(-0.65414)

- egu:105032472

- Down regulated genes

c171401\_g2(-0.65414)

- egu:105032472

- Down regulated genes

c171401\_g2(-0.65414)

- egu:105032472

- Down regulated genes

c171401\_g2(-0.65414)

- egu:105032472

- Down regulated genes

c171401\_g2(-0.65414)

- egu:105032472

- Down regulated genes

c171401\_g2(-0.65414)

- egu:105032472

- Down regulated genes

c171401\_g2(-0.65414)

- egu:105046147

- Down regulated genes

c122896\_g1(-0.73511)
- egu:105032472

- Down regulated genes

c171401\_g2(-0.65414)

- egu:105046147

- Down regulated genes

c122896\_g1(-0.73511)
- egu:105032472

- Down regulated genes

c171401\_g2(-0.65414)

- egu:105046147

- Down regulated genes

c122896\_g1(-0.73511)
- egu:105032472

- Down regulated genes

c171401\_g2(-0.65414)

- egu:105046147

- Down regulated genes

c122896\_g1(-0.73511)
- egu:105032472

- Down regulated genes

c171401\_g2(-0.65414)

- egu:105046147

- Down regulated genes

c122896\_g1(-0.73511)
- egu:105032472

- Down regulated genes

c171401\_g2(-0.65414)

- egu:105046147

- Down regulated genes

c122896\_g1(-0.73511)
- egu:105032472

- Down regulated genes

c171401\_g2(-0.65414)

- egu:105046147

- Down regulated genes

c122896\_g1(-0.73511)
- egu:105032472

- Down regulated genes

c171401\_g2(-0.65414)

- egu:105046147

- Down regulated genes

c122896\_g1(-0.73511)
- egu:105032472

- Down regulated genes

c171401\_g2(-0.65414)

- egu:105046147

- Down regulated genes

c122896\_g1(-0.73511)
- egu:105032472

- Down regulated genes

c171401\_g2(-0.65414)

- egu:105046147

- Down regulated genes

c122896\_g1(-0.73511)
- egu:105032472

- Down regulated genes

c171401\_g2(-0.65414)

- egu:105046147

- Down regulated genes

c122896\_g1(-0.73511)
- egu:105032472

- Down regulated genes

c171401\_g2(-0.65414)

- egu:105046147

- Down regulated genes

c122896\_g1(-0.73511)
- egu:105032472

- Down regulated genes

c171401\_g2(-0.65414)

- egu:105046147

- Down regulated genes

c122896\_g1(-0.73511)
- egu:105032472

- Down regulated genes

c171401\_g2(-0.65414)

- egu:105046147

- Down regulated genes

c122896\_g1(-0.73511)
- egu:105032472

- Down regulated genes

c171401\_g2(-0.65414)

- egu:105046147

- Down regulated genes

c122896\_g1(-0.73511)
- egu:105032472

- Down regulated genes

c171401\_g2(-0.65414)

- egu:105046147

- Down regulated genes

c122896\_g1(-0.73511)
- egu:105032472

- Down regulated genes

c171401\_g2(-0.65414)

- egu:105046147

- Down regulated genes

c122896\_g1(-0.73511)
- egu:105032472

- Down regulated genes

c171401\_g2(-0.65414)

- egu:105046147

- Down regulated genes

c122896\_g1(-0.73511)
- egu:105032472

- Down regulated genes

c171401\_g2(-0.65414)

- egu:105046147

- Down regulated genes

c122896\_g1(-0.73511)
- egu:105032472

- Down regulated genes

c171401\_g2(-0.65414)

- egu:105046147

- Down regulated genes

c122896\_g1(-0.73511)
- egu:105032472

- Down regulated genes

c171401\_g2(-0.65414)

- egu:105046147

- Down regulated genes

c122896\_g1(-0.73511)
- egu:105032472

- Down regulated genes

c171401\_g2(-0.65414)

- egu:105046147

- Down regulated genes

c122896\_g1(-0.73511)
- egu:105032472

- Down regulated genes

c171401\_g2(-0.65414)

- egu:105046147

- Down regulated genes

c122896\_g1(-0.73511)
- egu:105032472

- Down regulated genes

c171401\_g2(-0.65414)

- egu:105046147

- Down regulated genes

c122896\_g1(-0.73511)
- egu:105032472

- Down regulated genes

c171401\_g2(-0.65414)

- egu:105059048

- Down regulated genes

c167963\_g1(-1.7891)

- egu:105055982

- Down regulated genes

c158576\_g4(-3.3247)

- egu:105059048

- Down regulated genes

c167963\_g1(-1.7891)
- egu:105043957

- Down regulated genes

c162118\_g1(-0.71227)
- egu:105038022

- Down regulated genes

c163169\_g1(-0.78154)

- egu:105059048

- Down regulated genes

c167963\_g1(-1.7891)
- egu:105043957

- Down regulated genes

c162118\_g1(-0.71227)
- egu:105038022

- Down regulated genes

c163169\_g1(-0.78154)

- egu:105059048

- Down regulated genes

c167963\_g1(-1.7891)
- egu:105043957

- Down regulated genes

c162118\_g1(-0.71227)
- egu:105038022

- Down regulated genes

c163169\_g1(-0.78154)

- egu:105059048

- Down regulated genes

c167963\_g1(-1.7891)
- egu:105043957

- Down regulated genes

c162118\_g1(-0.71227)
- egu:105038022

- Down regulated genes

c163169\_g1(-0.78154)

- egu:105059048

- Down regulated genes

c167963\_g1(-1.7891)
- egu:105043957

- Down regulated genes

c162118\_g1(-0.71227)
- egu:105038022

- Down regulated genes

c163169\_g1(-0.78154)

- egu:105059048

- Down regulated genes

c167963\_g1(-1.7891)
- egu:105043957

- Down regulated genes

c162118\_g1(-0.71227)
- egu:105038022

- Down regulated genes

c163169\_g1(-0.78154)

- egu:105059048

- Down regulated genes

c167963\_g1(-1.7891)
- egu:105043957

- Down regulated genes

c162118\_g1(-0.71227)
- egu:105038022

- Down regulated genes

c163169\_g1(-0.78154)

- egu:105059048

- Down regulated genes

c167963\_g1(-1.7891)
- egu:105043957

- Down regulated genes

c162118\_g1(-0.71227)
- egu:105038022

- Down regulated genes

c163169\_g1(-0.78154)

- egu:105059048

- Down regulated genes

c167963\_g1(-1.7891)
- egu:105043957

- Down regulated genes

c162118\_g1(-0.71227)
- egu:105038022

- Down regulated genes

c163169\_g1(-0.78154)

- egu:105059048

- Down regulated genes

c167963\_g1(-1.7891)
- egu:105043957

- Down regulated genes

c162118\_g1(-0.71227)
- egu:105038022

- Down regulated genes

c163169\_g1(-0.78154)

- egu:105059048

- Down regulated genes

c167963\_g1(-1.7891)
- egu:105043957

- Down regulated genes

c162118\_g1(-0.71227)
- egu:105038022

- Down regulated genes

c163169\_g1(-0.78154)

Close
