# Supplementary material for: Key anti-freeze genes and pathways of Lanzhou lily (Lilium davidii, var. unicolor) during the seedling stage
Source: PLoS One. 2024 Mar 21;19(3):e0299259. doi: 10.1371/journal.pone.0299259 (PMC10956819; doi:10.1371/journal.pone.0299259)
Supplement: S2 File — (ZIP) [file pone.0299259.s005.zip › S2 Zip/src/egu03008.html]

egu03008


- egu:105054641

- Down regulated genes

c150398\_g4(-0.71873)
- egu:105054640

- Down regulated genes

c113253\_g1(-0.61237)

Close
