# Supplementary material for: Key anti-freeze genes and pathways of Lanzhou lily (Lilium davidii, var. unicolor) during the seedling stage
Source: PLoS One. 2024 Mar 21;19(3):e0299259. doi: 10.1371/journal.pone.0299259 (PMC10956819; doi:10.1371/journal.pone.0299259)
Supplement: S2 File — (ZIP) [file pone.0299259.s005.zip › S2 Zip/src/egu03010.html]

egu03010


- egu:105058445

- Down regulated genes

c146729\_g1(-0.54812)

- egu:105032412

- Down regulated genes

c155204\_g1(-1.7703) c178004\_g1(-1.9413)

- egu:105044731

- Down regulated genes

c173687\_g6(-0.73164)

- egu:105044673

- Down regulated genes

c168022\_g1(-1.0354)

- egu:105036502

- Down regulated genes

c132904\_g1(-0.72422)

- egu:105059802

- Down regulated genes

c159746\_g1(-0.54813)

- egu:105041287

- Down regulated genes

c160112\_g1(-0.60949)

- egu:105052800

- Down regulated genes

c163280\_g1(-1.1565)

- egu:105043313

- Down regulated genes

c143884\_g1(-0.966)

- egu:105034395

- Down regulated genes

c145214\_g1(-0.90912)

- egu:105041074

- Down regulated genes

c160468\_g1(-0.78228)

- egu:105040137

- Down regulated genes

c134374\_g1(-1.1277)

- egu:105034390

- Down regulated genes

c157065\_g1(-0.96196)

- egu:105052661

- Down regulated genes

c27497\_g1(-0.80257)

- egu:105056818

- Down regulated genes

c143129\_g1(-1.0905)

- egu:105035316

- Down regulated genes

c151905\_g1(-1.1024)

- egu:105059074

- Down regulated genes

c156427\_g1(-1.222)

- egu:105048206

- Down regulated genes

c94348\_g1(-1.2506)

- egu:105046043

- Down regulated genes

c116569\_g1(-0.90342)

- egu:105053938

- Down regulated genes

c106647\_g1(-1.0519)

- egu:105040763

- Down regulated genes

c150484\_g1(-0.94925)

- egu:105048988

- Down regulated genes

c141240\_g1(-1.158)

- egu:105059189

- Down regulated genes

c224200\_g1(-1.1925)

- egu:105048529

- Down regulated genes

c141711\_g1(-0.90312)

- egu:105033340

- Down regulated genes

c48496\_g1(-1.333)

- egu:105045120

- Down regulated genes

c172832\_g1(-1.1098)

- egu:105061575

- Down regulated genes

c159369\_g1(-1.1953)

- egu:105060039

- Down regulated genes

c161109\_g1(-1.0631)

- egu:105032797

- Down regulated genes

c158737\_g2(-1.0996)

- egu:105032842

- Down regulated genes

c237068\_g1(-0.63182)

- egu:105041319

- Down regulated genes

c138659\_g1(-0.96968)

- egu:105034754

- Down regulated genes

c132540\_g1(-1.1793)

- egu:105047611

- Down regulated genes

c142600\_g1(-0.80375)

- egu:105042443

- Down regulated genes

c140525\_g1(-0.67096)

- egu:105057725

- Down regulated genes

c156664\_g1(-0.61675)

Close
