# Supplementary material for: Key anti-freeze genes and pathways of Lanzhou lily (Lilium davidii, var. unicolor) during the seedling stage
Source: PLoS One. 2024 Mar 21;19(3):e0299259. doi: 10.1371/journal.pone.0299259 (PMC10956819; doi:10.1371/journal.pone.0299259)
Supplement: S2 File — (ZIP) [file pone.0299259.s005.zip › S2 Zip/src/egu03015.html]

egu03015


- egu:105052667

- Down regulated genes

c158179\_g1(-0.73939)

- egu:105035877

- Down regulated genes

c121960\_g1(-0.99644)
- egu:105050147

- Down regulated genes

c168902\_g1(-0.82295)

- egu:105039096

- Down regulated genes

c116624\_g1(-1.0674)

- egu:105035877

- Down regulated genes

c121960\_g1(-0.99644)
- egu:105050147

- Down regulated genes

c168902\_g1(-0.82295)

- egu:105046347

- Down regulated genes

c153337\_g3(-0.73045)

Close
