# Supplementary material for: Key anti-freeze genes and pathways of Lanzhou lily (Lilium davidii, var. unicolor) during the seedling stage
Source: PLoS One. 2024 Mar 21;19(3):e0299259. doi: 10.1371/journal.pone.0299259 (PMC10956819; doi:10.1371/journal.pone.0299259)
Supplement: S2 File — (ZIP) [file pone.0299259.s005.zip › S2 Zip/src/egu03018.html]

egu03018


- egu:105039219

- Down regulated genes

c163642\_g1(-0.73792)

- egu:105035219

- Down regulated genes

c167990\_g1(-0.71128)

- egu:105040019

- Down regulated genes

c159391\_g2(-0.77447)

- egu:105060207

- Down regulated genes

c166194\_g5(-1.7775)

- egu:105035219

- Down regulated genes

c167990\_g1(-0.71128)

- egu:105050147

- Down regulated genes

c168902\_g1(-0.82295)
- egu:105035877

- Down regulated genes

c121960\_g1(-0.99644)

- egu:105039219

- Down regulated genes

c163642\_g1(-0.73792)

Close
