# Supplementary material for: Key anti-freeze genes and pathways of Lanzhou lily (Lilium davidii, var. unicolor) during the seedling stage
Source: PLoS One. 2024 Mar 21;19(3):e0299259. doi: 10.1371/journal.pone.0299259 (PMC10956819; doi:10.1371/journal.pone.0299259)
Supplement: S2 File — (ZIP) [file pone.0299259.s005.zip › S2 Zip/src/egu03020.html]

egu03020


- egu:105055141

- Down regulated genes

c173864\_g1(-0.58137)

- egu:105034397

- Down regulated genes

c171631\_g8(-3.183)

Close
