# Supplementary material for: Key anti-freeze genes and pathways of Lanzhou lily (Lilium davidii, var. unicolor) during the seedling stage
Source: PLoS One. 2024 Mar 21;19(3):e0299259. doi: 10.1371/journal.pone.0299259 (PMC10956819; doi:10.1371/journal.pone.0299259)
Supplement: S2 File — (ZIP) [file pone.0299259.s005.zip › S2 Zip/src/egu03040.html]

egu03040


- egu:105054037

- Down regulated genes

c156221\_g2(-1.0235) c156221\_g1(-0.99164)

- egu:105053459

- Down regulated genes

c83498\_g1(-1.1934)

- egu:105036282

- Down regulated genes

c162358\_g1(-1.1604)

- egu:105056954

- Down regulated genes

c171346\_g1(-2.124)

- egu:105045262

- Down regulated genes

c169453\_g6(-0.61885)

- egu:105053459

- Down regulated genes

c83498\_g1(-1.1934)

- egu:105038221

- Down regulated genes

c155072\_g1(-0.59401)

Close
