# Supplementary material for: Key anti-freeze genes and pathways of Lanzhou lily (Lilium davidii, var. unicolor) during the seedling stage
Source: PLoS One. 2024 Mar 21;19(3):e0299259. doi: 10.1371/journal.pone.0299259 (PMC10956819; doi:10.1371/journal.pone.0299259)
Supplement: S2 File — (ZIP) [file pone.0299259.s005.zip › S2 Zip/src/egu03060.html]

egu03060


- egu:105044437

- Down regulated genes

c123568\_g1(-0.64573)

- egu:105055789

- Down regulated genes

c132523\_g1(-0.56086)

- egu:105038252

- Down regulated genes

c170040\_g1(-0.71861)

- egu:105038252

- Down regulated genes

c170040\_g1(-0.71861)

Close
