# Supplementary material for: Key anti-freeze genes and pathways of Lanzhou lily (Lilium davidii, var. unicolor) during the seedling stage
Source: PLoS One. 2024 Mar 21;19(3):e0299259. doi: 10.1371/journal.pone.0299259 (PMC10956819; doi:10.1371/journal.pone.0299259)
Supplement: S2 File — (ZIP) [file pone.0299259.s005.zip › S2 Zip/src/egu04070.html]

egu04070


- egu:105058765

- Down regulated genes

c166373\_g4(-0.90377)

- egu:105055982

- Down regulated genes

c158576\_g4(-3.3247)

- egu:105049214

- Down regulated genes

c161796\_g1(-0.83749)

- egu:105049214

- Down regulated genes

c161796\_g1(-0.83749)

- egu:105049214

- Down regulated genes

c161796\_g1(-0.83749)

Close
