# Supplementary material for: Key anti-freeze genes and pathways of Lanzhou lily (Lilium davidii, var. unicolor) during the seedling stage
Source: PLoS One. 2024 Mar 21;19(3):e0299259. doi: 10.1371/journal.pone.0299259 (PMC10956819; doi:10.1371/journal.pone.0299259)
Supplement: S2 File — (ZIP) [file pone.0299259.s005.zip › S2 Zip/src/egu04075.html]

egu04075


- egu:105055597

- Down regulated genes

c142840\_g2(-1.4764)

- egu:105040625

- Down regulated genes

c166932\_g3(-0.64787)

- egu:105033977

- Down regulated genes

c169534\_g1(-1.7398)

- egu:105040857

- Down regulated genes

c141042\_g1(-0.52464)
- egu:105042180

- Down regulated genes

c153105\_g1(-0.95872)

- egu:105052996

- Down regulated genes

c164642\_g1(-0.94868)

- egu:105039360

- Down regulated genes

c141225\_g1(-0.63187) c156359\_g1(-0.55785)

- egu:105057151

- Down regulated genes

c164451\_g1(-0.57392) c156282\_g1(-0.54075)

- egu:105038391

- Down regulated genes

c78828\_g1(-Inf)

- egu:105045549

- Down regulated genes

c163360\_g1(-1.2188)

- egu:105041587

- Down regulated genes

c163658\_g1(-1.3261)

- egu:105032733

- Down regulated genes

c173216\_g1(-1.2544)

- egu:105052748

- Down regulated genes

c146802\_g3(-1.4711)

- egu:105050174

- Down regulated genes

c155948\_g3(-2.0641) c155948\_g1(-0.6894)

- egu:105033626

- Down regulated genes

c173362\_g3(-0.88636)

- egu:105040857

- Down regulated genes

c141042\_g1(-0.52464)
- egu:105042180

- Down regulated genes

c153105\_g1(-0.95872)

- egu:105036306

- Down regulated genes

c167725\_g1(-1.6861) c167725\_g2(-1.2779)
- egu:105043524

- Down regulated genes

c153395\_g1(-1.8222)
- egu:105035706

- Down regulated genes

c133669\_g1(-3.5186)
- egu:105034824

- Down regulated genes

c164908\_g1(-3.9691)
- egu:105042455

- Down regulated genes

c157547\_g1(-Inf) c93769\_g1(-7.2334)
- egu:105042452

- Down regulated genes

c116329\_g1(-3.6503)

- egu:105046249

- Down regulated genes

c146374\_g1(-0.70877)
- egu:105044079

- Down regulated genes

c140543\_g1(-1.2914)

- egu:105060907

- Down regulated genes

c146567\_g1(-0.72927) c173307\_g1(-0.77662)

Close
