# Supplementary material for: Key anti-freeze genes and pathways of Lanzhou lily (Lilium davidii, var. unicolor) during the seedling stage
Source: PLoS One. 2024 Mar 21;19(3):e0299259. doi: 10.1371/journal.pone.0299259 (PMC10956819; doi:10.1371/journal.pone.0299259)
Supplement: S2 File — (ZIP) [file pone.0299259.s005.zip › S2 Zip/src/egu04120.html]

egu04120


- egu:105059434

- Down regulated genes

c133199\_g1(-0.87924)

- egu:105055105

- Down regulated genes

c156645\_g1(-1.7748)

- egu:105055105

- Down regulated genes

c156645\_g1(-1.7748)

- egu:105057710

- Down regulated genes

c163535\_g1(-0.82329)
- egu:105053631

- Down regulated genes

c127313\_g1(-1.8573)

- egu:105040742

- Down regulated genes

c151484\_g1(-0.53575) c27396\_g2(-0.70526)

- egu:105047918

- Down regulated genes

c145598\_g2(-0.56451)
- egu:105055896

- Down regulated genes

c167530\_g4(-1.2987)

Close
