# Supplementary material for: Key anti-freeze genes and pathways of Lanzhou lily (Lilium davidii, var. unicolor) during the seedling stage
Source: PLoS One. 2024 Mar 21;19(3):e0299259. doi: 10.1371/journal.pone.0299259 (PMC10956819; doi:10.1371/journal.pone.0299259)
Supplement: S2 File — (ZIP) [file pone.0299259.s005.zip › S2 Zip/src/egu04141.html]

egu04141


- egu:105042748

- Down regulated genes

c159704\_g1(-0.60344)

- egu:105034003

- Down regulated genes

c141023\_g1(-0.60325)

- egu:105056954

- Down regulated genes

c171346\_g1(-2.124)

- egu:105040742

- Down regulated genes

c151484\_g1(-0.53575) c27396\_g2(-0.70526)

- egu:105056954

- Down regulated genes

c171346\_g1(-2.124)

- egu:105040742

- Down regulated genes

c151484\_g1(-0.53575) c27396\_g2(-0.70526)

- egu:105055105

- Down regulated genes

c156645\_g1(-1.7748)

- egu:105042852

- Down regulated genes

c132613\_g1(-1.1973) c152923\_g1(-0.80798)

- egu:105050014

- Down regulated genes

c168421\_g1(-0.60634)

- egu:105050569

- Down regulated genes

c168095\_g1(-1.287) c172850\_g2(-1.4914)

- egu:105056346

- Down regulated genes

c167026\_g8(-2.8791)

Close
