# Supplementary material for: Key anti-freeze genes and pathways of Lanzhou lily (Lilium davidii, var. unicolor) during the seedling stage
Source: PLoS One. 2024 Mar 21;19(3):e0299259. doi: 10.1371/journal.pone.0299259 (PMC10956819; doi:10.1371/journal.pone.0299259)
Supplement: S1 File — (ZIP) [file pone.0299259.s004.zip › S1 Zip/src/egu03030.html]

egu03030


- egu:105060234

- Up regulated genes

c31927\_g1(2.7264)

- egu:105045532

- Up regulated genes

c146039\_g1(6.3533)

- egu:105045532

- Up regulated genes

c146039\_g1(6.3533)

Close
