# Supplementary material for: Key anti-freeze genes and pathways of Lanzhou lily (Lilium davidii, var. unicolor) during the seedling stage
Source: PLoS One. 2024 Mar 21;19(3):e0299259. doi: 10.1371/journal.pone.0299259 (PMC10956819; doi:10.1371/journal.pone.0299259)
Supplement: S1 File — (ZIP) [file pone.0299259.s004.zip › S1 Zip/src/egu03040.html]

egu03040


- egu:105050579

- Up regulated genes

c173699\_g2(2.1336)

- egu:105040405

- Up regulated genes

c160522\_g1(4.9414)

- egu:105061246

- Up regulated genes

c161316\_g2(1.1024)

- egu:105045690

- Up regulated genes

c163181\_g5(1.7337)

- egu:105041295

- Up regulated genes

c172537\_g3(Inf)
- egu:105048742

- Up regulated genes

c172537\_g1(4.0252)

Close
