# Supplementary material for: Key anti-freeze genes and pathways of Lanzhou lily (Lilium davidii, var. unicolor) during the seedling stage
Source: PLoS One. 2024 Mar 21;19(3):e0299259. doi: 10.1371/journal.pone.0299259 (PMC10956819; doi:10.1371/journal.pone.0299259)
Supplement: S1 File — (ZIP) [file pone.0299259.s004.zip › S1 Zip/src/egu04070.html]

egu04070


- egu:105057333

- Up regulated genes

c101051\_g1(4.9356) c166855\_g1(3.1027) c145934\_g1(8.2812)
- egu:105044995

- Up regulated genes

c168165\_g3(3.3946)

- egu:105056668

- Up regulated genes

c152208\_g2(0.80405)

- egu:105038590

- Up regulated genes

c166846\_g1(1.2816)

- egu:105056668

- Up regulated genes

c152208\_g2(0.80405)

- egu:105054063

- Up regulated genes

c148900\_g2(0.60242)

- egu:105054063

- Up regulated genes

c148900\_g2(0.60242)

Close
