# Supplementary material for: Key anti-freeze genes and pathways of Lanzhou lily (Lilium davidii, var. unicolor) during the seedling stage
Source: PLoS One. 2024 Mar 21;19(3):e0299259. doi: 10.1371/journal.pone.0299259 (PMC10956819; doi:10.1371/journal.pone.0299259)
Supplement: S1 File — (ZIP) [file pone.0299259.s004.zip › S1 Zip/src/egu04075.html]

egu04075


- egu:105059593

- Up regulated genes

c173231\_g1(5.7325)

- egu:105042113

- Up regulated genes

c157302\_g2(0.88049)
- egu:105057423

- Up regulated genes

c169341\_g1(1.2947)

- egu:105052243

- Up regulated genes

c87233\_g1(3.9282)

- egu:105056896

- Up regulated genes

c96913\_g1(2.0305)
- egu:105043050

- Up regulated genes

c159509\_g4(2.551)

- egu:105043791

- Up regulated genes

c147612\_g1(1.9534)

- egu:105038245

- Up regulated genes

c165097\_g1(1.9954)

- egu:105056702

- Up regulated genes

c164987\_g1(Inf)

- egu:105035319

- Up regulated genes

c149381\_g1(0.71133)

- egu:105058413

- Up regulated genes

c114134\_g1(3.1027)

- egu:105036306

- Up regulated genes

c167964\_g1(3.142)
- egu:105034824

- Up regulated genes

c171320\_g6(3.4922) c157031\_g1(3.4513)
- egu:105052902

- Up regulated genes

c164663\_g1(3.8916) c199038\_g1(Inf)

Close
