# Supplementary material for: Key anti-freeze genes and pathways of Lanzhou lily (Lilium davidii, var. unicolor) during the seedling stage
Source: PLoS One. 2024 Mar 21;19(3):e0299259. doi: 10.1371/journal.pone.0299259 (PMC10956819; doi:10.1371/journal.pone.0299259)
Supplement: S1 File — (ZIP) [file pone.0299259.s004.zip › S1 Zip/src/egu04141.html]

egu04141


- egu:105053227

- Up regulated genes

c42777\_g1(0.87089)

- egu:105045690

- Up regulated genes

c163181\_g5(1.7337)

- egu:105045690

- Up regulated genes

c163181\_g5(1.7337)

- egu:105053788

- Up regulated genes

c163791\_g1(0.67924)

- egu:105053689

- Up regulated genes

c159312\_g1(0.68776)

- egu:105042707

- Up regulated genes

c141977\_g1(3.65)

Close
