# Supplementary material for: Key anti-freeze genes and pathways of Lanzhou lily (Lilium davidii, var. unicolor) during the seedling stage
Source: PLoS One. 2024 Mar 21;19(3):e0299259. doi: 10.1371/journal.pone.0299259 (PMC10956819; doi:10.1371/journal.pone.0299259)
Supplement: S1 File — (ZIP) [file pone.0299259.s004.zip › S1 Zip/src/egu04144.html]

egu04144


- egu:105045690

- Up regulated genes

c163181\_g5(1.7337)

- egu:105045676

- Up regulated genes

c161639\_g1(1.7522)

- egu:105033970

- Up regulated genes

c165623\_g2(2.8532)

- egu:105033970

- Up regulated genes

c165623\_g2(2.8532)

- egu:105046398

- Up regulated genes

c173778\_g2(3.9182)

- egu:105033970

- Up regulated genes

c165623\_g2(2.8532)

- egu:105044044

- Up regulated genes

c142610\_g1(3.0068) c142610\_g2(2.6414)

- egu:105047696

- Up regulated genes

c158968\_g2(1.7055) c158968\_g1(1.9214)

- egu:105058367

- Up regulated genes

c173338\_g6(2.0462)
- egu:105041064

- Up regulated genes

c163801\_g1(5.2385)
- egu:105038113

- Up regulated genes

c171004\_g1(2.0584)

Close
