# Supplementary material for: Key anti-freeze genes and pathways of Lanzhou lily (Lilium davidii, var. unicolor) during the seedling stage
Source: PLoS One. 2024 Mar 21;19(3):e0299259. doi: 10.1371/journal.pone.0299259 (PMC10956819; doi:10.1371/journal.pone.0299259)
Supplement: S1 File — (ZIP) [file pone.0299259.s004.zip › S1 Zip/src/egu04626.html]

egu04626


- egu:105050781

- Up regulated genes

c147877\_g2(1.7705)

- egu:105044758

- Up regulated genes

c152643\_g2(5.3429) c166605\_g3(2.7526) c152643\_g1(2.9803)

- egu:105041258

- Up regulated genes

c162064\_g1(2.4987)
- egu:105042206

- Up regulated genes

c164545\_g2(1.5735)
- egu:105057333

- Up regulated genes

c166855\_g1(3.1027) c145934\_g1(8.2812) c101051\_g1(4.9356)
- egu:105037682

- Up regulated genes

c144017\_g1(Inf) c144017\_g2(Inf) c159000\_g1(2.9702) c140553\_g1(9.9651) c170503\_g1(Inf)
- egu:105038625

- Up regulated genes

c140013\_g1(3.7728)
- egu:105039372

- Up regulated genes

c154815\_g1(Inf) c173122\_g1(7.8456) c168962\_g1(Inf) c163915\_g1(Inf)
- egu:105046031

- Up regulated genes

c156536\_g2(2.9685) c156536\_g1(1.2021)
- egu:105034665

- Up regulated genes

c152058\_g1(2.5917) c152058\_g2(2.1536)
- egu:105047887

- Up regulated genes

c153028\_g1(0.7024)
- egu:105044995

- Up regulated genes

c168165\_g3(3.3946)
- egu:105052456

- Up regulated genes

c147918\_g2(1.0107)
- egu:105045551

- Up regulated genes

c153815\_g1(2.4485) c147831\_g1(0.68106)
- egu:105057654

- Up regulated genes

c113196\_g1(1.5527)

- egu:105051034

- Up regulated genes

c173897\_g7(1.528) c153491\_g1(1.2884)

- egu:105060351

- Up regulated genes

c166423\_g2(1.3977)
- egu:105042006

- Up regulated genes

c145559\_g1(2.0297)

- egu:105054415

- Up regulated genes

c156798\_g2(1.9126)
- egu:105052035

- Up regulated genes

c163371\_g2(2.0605)

Close
