# Supplementary material for: Key anti-freeze genes and pathways of Lanzhou lily (Lilium davidii, var. unicolor) during the seedling stage
Source: PLoS One. 2024 Mar 21;19(3):e0299259. doi: 10.1371/journal.pone.0299259 (PMC10956819; doi:10.1371/journal.pone.0299259)
Supplement: S1 File — (ZIP) [file pone.0299259.s004.zip › S1 Zip/src/egu04712.html]

egu04712


- egu:105041113

- Up regulated genes

c174283\_g3(3.9233)

- egu:105054824

- Up regulated genes

c153783\_g1(0.7296)

- egu:105036385

- Up regulated genes

c156146\_g2(2.1536) c171563\_g1(2.3117)
- egu:105047639

- Up regulated genes

c156146\_g1(2.0683)

- egu:105045005

- Up regulated genes

c151010\_g1(3.2906)

- egu:105036385

- Up regulated genes

c156146\_g2(2.1536) c171563\_g1(2.3117)
- egu:105047639

- Up regulated genes

c156146\_g1(2.0683)

- egu:105041005

- Up regulated genes

c131089\_g1(Inf) c144654\_g1(6.9885)

- egu:105058499

- Up regulated genes

c167910\_g3(0.74901)

- egu:105059424

- Up regulated genes

c152959\_g1(4.2291)

- egu:105054824

- Up regulated genes

c153783\_g1(0.7296)

- egu:105045005

- Up regulated genes

c151010\_g1(3.2906)

Close
