# Supplementary material for: Key anti-freeze genes and pathways of Lanzhou lily (Lilium davidii, var. unicolor) during the seedling stage
Source: PLoS One. 2024 Mar 21;19(3):e0299259. doi: 10.1371/journal.pone.0299259 (PMC10956819; doi:10.1371/journal.pone.0299259)
Supplement: S2 File — (ZIP) [file pone.0299259.s005.zip › S2 Zip/src/egu04144.html]

egu04144


- egu:105056954

- Down regulated genes

c171346\_g1(-2.124)

- egu:105061464

- Down regulated genes

c164929\_g1(-0.58258)

- egu:105038113

- Down regulated genes

c168008\_g1(-1.0886)

Close
