# Supplementary material for: Key anti-freeze genes and pathways of Lanzhou lily (Lilium davidii, var. unicolor) during the seedling stage
Source: PLoS One. 2024 Mar 21;19(3):e0299259. doi: 10.1371/journal.pone.0299259 (PMC10956819; doi:10.1371/journal.pone.0299259)
Supplement: S2 File — (ZIP) [file pone.0299259.s005.zip › S2 Zip/src/egu04145.html]

egu04145


- egu:105054774

- Down regulated genes

c158225\_g2(-0.94779)
- egu:105046462

- Down regulated genes

c105637\_g1(-0.56386)

- egu:105059836

- Down regulated genes

c141394\_g1(-2.0277)
- egu:105045457

- Down regulated genes

c141394\_g3(-1.3738)

- egu:105051928

- Down regulated genes

c158106\_g1(-0.60077)
- egu:105054293

- Down regulated genes

c150055\_g1(-0.56168)

- egu:105051928

- Down regulated genes

c158106\_g1(-0.60077)
- egu:105054293

- Down regulated genes

c150055\_g1(-0.56168)

- egu:105043351

- Down regulated genes

c141160\_g2(-2.1455)

- egu:105051928

- Down regulated genes

c158106\_g1(-0.60077)
- egu:105054293

- Down regulated genes

c150055\_g1(-0.56168)

Close
