# Supplementary material for: Key anti-freeze genes and pathways of Lanzhou lily (Lilium davidii, var. unicolor) during the seedling stage
Source: PLoS One. 2024 Mar 21;19(3):e0299259. doi: 10.1371/journal.pone.0299259 (PMC10956819; doi:10.1371/journal.pone.0299259)
Supplement: S2 File — (ZIP) [file pone.0299259.s005.zip › S2 Zip/src/egu04146.html]

egu04146


- egu:105035642

- Down regulated genes

c163366\_g1(-1.7043)
- egu:105039221

- Down regulated genes

c169731\_g1(-1.0084)

- egu:105061386

- Down regulated genes

c103250\_g1(-2.8827)

- egu:105059069

- Down regulated genes

c151165\_g1(-0.6154)
- egu:105060838

- Down regulated genes

c152778\_g1(-0.75869)

- egu:105058041

- Down regulated genes

c147579\_g1(-1.4187)
- egu:105044749

- Down regulated genes

c160079\_g1(-1.1364)
- egu:105047481

- Down regulated genes

c156939\_g1(-0.80103)

- egu:105052909

- Down regulated genes

c140758\_g1(-0.77014)

Close
