# Supplementary material for: Key anti-freeze genes and pathways of Lanzhou lily (Lilium davidii, var. unicolor) during the seedling stage
Source: PLoS One. 2024 Mar 21;19(3):e0299259. doi: 10.1371/journal.pone.0299259 (PMC10956819; doi:10.1371/journal.pone.0299259)
Supplement: S2 File — (ZIP) [file pone.0299259.s005.zip › S2 Zip/src/egu04626.html]

egu04626


- egu:105057983

- Down regulated genes

c164943\_g1(-0.8467)
- egu:105046801

- Down regulated genes

c143148\_g1(-0.63596)

- egu:105057238

- Down regulated genes

c27081\_g1(-1.2137)

- egu:105052035

- Down regulated genes

c163371\_g1(-0.96906)

- egu:105050014

- Down regulated genes

c168421\_g1(-0.60634)

Close
